# A guide to bullvalene stereodynamics†

**DOI:** 10.1039/d4sc03700f

**Published:** 2024-08-23

**Authors:** Robert A. Ives, William Maturi, Matthew T. Gill, Conor Rankine, Paul R. McGonigal

**Affiliations:** a Department of Chemistry, University of York Heslington York YO10 5DD UK paul.mcgonigal@york.ac.uk; b Department of Chemistry, Durham University Lower Mountjoy, Stockton Road Durham DH1 3LE UK

## Abstract

Here, we analyze the stereodynamic properties of bullvalenes using principal moments of inertia and exit vector plots to draw comparisons with commonly used ring systems in medicinal chemistry. To aid analyses, we first classify (i) the four elementary rearrangement steps available to substituted bullvalenes, which (ii) can be described by applying positional descriptors (α, β, γ, and δ) to the substituents. We also (iii) derive an intuitive equation to calculate the number of isomers for a given bullvalene system. Using DFT-modelled structures for di-, tri-, and tetrasubstituted bullvalenes, generated using a newly developed computational tool (*bullviso*), we show that their 3D shapes and the exit vectors available from the bullvalene scaffold make them comparable to other bioisosteres currently used to replace planar aromatic ring systems in drug discovery. Unlike conventional ring systems, the shapeshifting valence isomerism of bullvalenes gives rise to numerous shapes and substituent relationships attainable as a concentration-independent dynamic covalent library from a single compound. We visualize this property by applying population weightings to the principal moments of inertia and exit vector analyses to reflect the relative thermodynamic stabilities of the available isomers.

## Abstract 2

Population-weighted principal moments of inertia and exit vectors analyses of shapeshifting bullvalenes reveals the diversity in their 3D structures.

## Graphical abstract

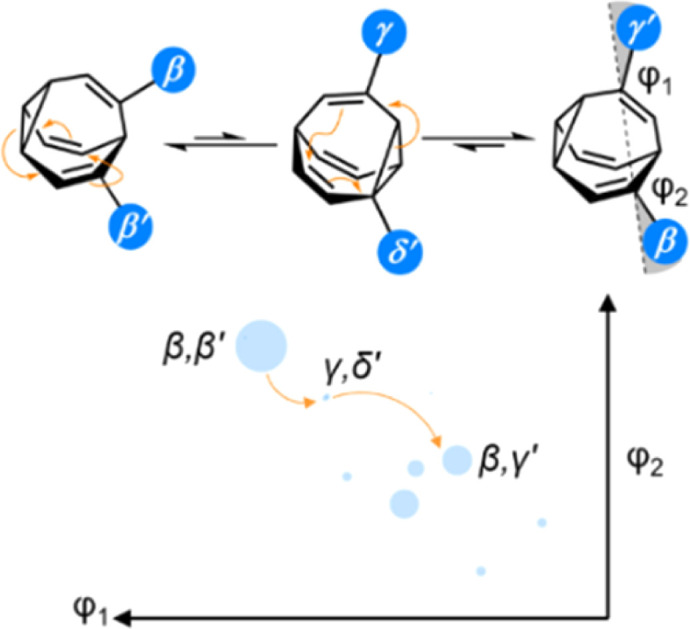

## Introduction

**Fig. 1:**
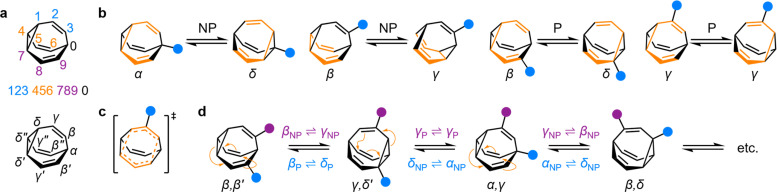
(a) The BV isomer barcode labelling system, top, and relative positional labels, bottom. (b) The possible exchange processes following one Cope rearrangement step, enumerated for each BV position. Full isomerization requires sequential steps that include (P = participating) and exclude (NP = non-participating) the substituent in the rearranging 1,5-hexadiene motif (shown in orange).^1*b*^ (c) Illustration of the higher symmetry in the transition state for γ_P_ ⇌ γ_P_ Cope rearrangement relative to the ground state. (d) The partial isomer network of a heterodisubstituted BV showing the positional exchange arising from three sequential Cope rearrangement steps.

The C_10_H_10_ cage bullvalene (BV, Fig. 1a) fluctuates between 1 209 600 degenerate isomers through rapid and reversible strain-promoted Cope rearrangements.^1^ There are now several useful synthetic methods available to prepare substituted derivatives of BV,^1*c*,2^ which fluctuate between nondegenerate constitutional isomers (Fig. 1) with distinct shapes. This ‘shapeshifting’ property of substituted BVs and other fluxional molecules has presented opportunities for their inclusion as dynamic structural units in functional molecules^3^ and materials, such as chemical sensors,^2*d*,4^ fluorophores,^5^ metal complexes,^6^ components of electromechanical systems,^7^ rigid-rod polymers,^8^ and antibiotics.^9^

In general, building blocks with rigid three-dimensional (3D) structures are key components of materials, such as metal–organic frameworks^10^ and other porous networks.^11^ In the context of medicinal chemistry research—particularly for fragment-based drug discovery^12^ (FBDD) libraries—it has been acknowledged that drug candidates based on flat or rod-like molecules offer limited shape diversity.^13^ Consequently, there is a desire for a greater number of diverse 3D fragments to be included within these libraries to cover more chemical space and, ultimately, to provide better candidates for drug development.^14^ One way that this objective can be achieved is by including fragments that possess diverse aliphatic ring systems, particularly as the core ring system is considered the key factor in shape diversity.^13^

The BV ring system has the seemingly contradictory characteristics of, on one hand, being highly dynamic through its reversible Cope rearrangements, while on the other hand, being a rigidly 3D structure. Its tricyclic hydrocarbon skeleton is a shape-persistent structure with substituents projecting outwards at well-defined angles. Therefore, designing effective materials based on BV derivatives requires understanding of their stereodynamics, *i.e.*, their overall 3D shapes, the relative orientations that are accessible to appended substituents, and the relative energies of the isomers at equilibrium.

Here, we quantify the 3D shape diversity accessible from BVs and demonstrate that their fluxional behavior enables them to reversibly access diverse areas of chemical space. We provide a concise guide to the rearrangement processes of substituted BVs and apply computational modelling to categorize and quantify their stereodynamics. To do so, we perform population-weighted principal moment of inertia (PMI)^15^ and exit vector (EV)^16^ analyses. Much of the analysis can be automated using a new computational tool we have developed, *bullviso*, that generates all the isomers of a given substituted BV, and the input files needed to compute relative energy levels of each isomer. To demonstrate its utility, we apply *bullviso* to examine di-, tri- and tetramethyl BVs. The analysis illustrates that shapeshifting networks of BV derivatives dynamically sample many different areas of chemical space from a single starting compound. They do so by positioning their substituents at a range of angles, extending beyond those typically found in *cis*-disubstituted rings, while maintaining a higher degree of sphericity than most common ring systems found in biologically active molecules.

## Results and discussion

**Fig. 2:**
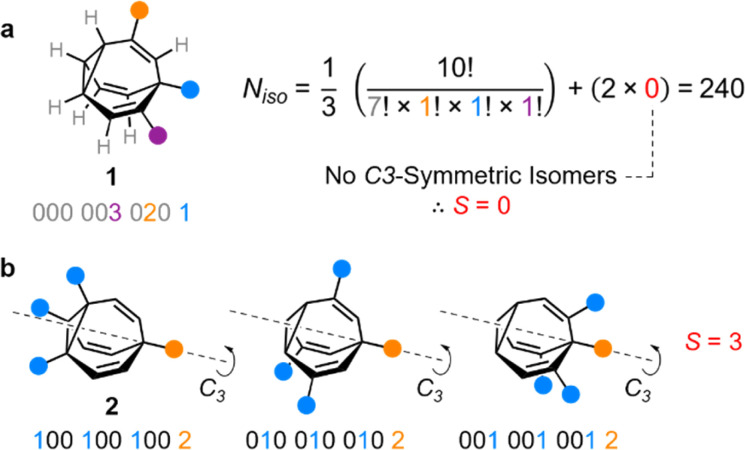
Structural formulae and isomer barcodes of (a) one of the 240 unique isomers of BV 1, showing the calculation of *N*_iso_, and (b) the three *C*_3_-symmetric isomers of BV 2 that are accounted for by a correction factor of *S* = 3.

Given the large number of possible BV isomers, a method for naming them is essential for any discussion involving their interconversion. Bode and coworkers developed an elegant barcode labelling system that can be parsed by computer algorithms to construct a full network map for interconversion of all the non-degenerate isomers of a substituted BV – a task that is otherwise impractical to do by hand. Each digit of the barcode represents one of the carbon atoms in the BV structure (Fig. 1a) and each type of substituent is given a numeral, enabling each isomer to be described with a unique numeric code (see examples in Fig. 2).^17^ Importantly, this system enables isomer information to be coded unambiguously. But while this naming system is ideally suited to comprehensively describing the positions of all substituents in any given isomer, its high level of detail is not always needed. Indeed, perhaps because the length of the barcodes and their unfamiliar appearance compared to typical nomenclature for organic structures, there has been a tendency in the literature to name bullvalene isomers with individual labels (such as numbers or letters, *e.g.*, isomer A, isomer B, *etc.*) that lack structural information. Therefore, we suggest that Greek letter locants be used as relative positional labels for succinctly discussing distinct positions in the BV structure and their relationships to one another through Cope rearrangement steps. The threefold rotational symmetry of the parent BV scaffold reduces its number of chemically inequivalent positions to four, which are labelled as α–δ (Fig. 1a) starting from α as the apical position, *i.e.*, the unique sp^3^-C, to δ as the cyclopropyl position. The olefin positions, which are typically the energetically favored sites for any non-hydrogen substituents,^2^ are labelled as β and γ. Prime and double prime labels can be used distinguish the same locants on different ‘arms’ of the BV.§§The use of Greek letter locants to label relative positions and prime symbols to label identical rings in multiple ring systems follows IUPAC conventions.^18^^18^ We find that this labelling system is a useful shorthand that is complementary to the more detailed barcode system. Where appropriate, both systems are used below.

### Elementary types of positional exchange

Each Cope rearrangement occurs on one of the three 1,5-hexadiene ‘faces’, involving two arms of the BV. The positions of the BV that are part of the 1,5-hexadiene motif undergoing a given Cope rearrangement can be referred to as participating sites (P), while the other four positions are non-participating sites (NP). Whether a substituent is located on a P or a NP site determines its resulting position following isomerization (Fig. 1b). The α position is never part of the 1,5-hexadiene motif, so it is always considered to be a NP site. On the other hand, for each of the other sites (β, γ, and δ), there exist two potential positional outcomes following a single rearrangement step.

Applying the α–δ and P/NP labels, it becomes clear that there are just four elementary types of positional exchange that occur during any Cope rearrangement step, which are illustrated in Fig. 1b using a monosubstituted BV as a model. Firstly, the sole possible outcome for the α position is migration to a newly formed δ position on the NP arm. A substituent at a β position will migrate to a γ position when on a NP arm, whereas P rearrangement exchanges the β position with a δ site. Finally, a γ substituent remains at a γ position on a P arm following the Cope rearrangement on account of symmetry in the transition state (Fig. 1c). As the Cope rearrangement is reversible, the reciprocal of each of these exchange processes must also occur. Overall, therefore, the elementary types of positional exchange that govern the outcome of any BV rearrangement are (i) α_NP_ ⇌ δ_NP_, (ii) β_NP_ ⇌ γ_NP_, (iii) β_P_ ⇌ δ_P_, and (iv) γ_P_ ⇌ γ_P_. These elementary types of positional exchange apply equally to every BV substituent in every isomer, regardless of the total number of substituents or their relative positions. For example, the functional groups of a heterodisubstituted BV migrate relative to one another during sequential Cope rearrangement steps, allowing them to switch from being on separate arms to occupying the same arm and back again (Fig. 1d).

### Calculating the number of unique bullvalene permutations

Deriving the total number of unique nondegenerate isomers for a given substitution pattern is one of the most important considerations for the construction of BV interconversion networks. Bode reported a MATLAB code to calculate the number of unique BV isomers.^17^ However, taking account of the symmetry present in BVs, it is possible to perform a simple ‘back-of-the-envelope’ calculation to determine the number of isomers for a given BV system using eqn (1):1
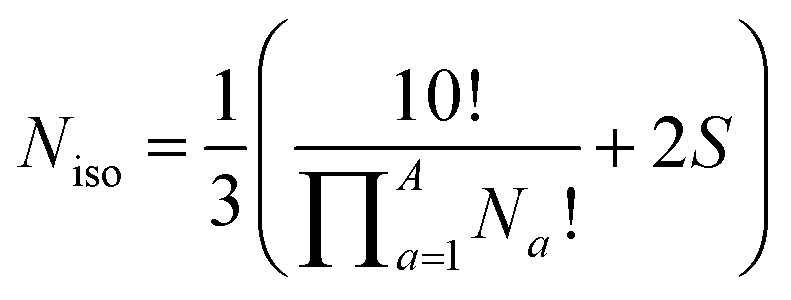
where *N*_iso_ is the number of unique nondegenerate isomers (enantiomers are considered to be distinct from one another), *N*_*a*_ is the number of occurrences of a given type of substituent, 
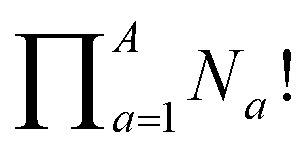
 the product of multiplying together the factorial of this term for each unique type of substituent (including hydrogen substituents), and *S* is a correction factor that accounts for the *C*_3_ symmetry of BV and has a value of 0, 1, 3, or 6. In the same manner as the isomer barcode system, eqn (1) treats each substituent as a number within a group of ten numbers.

Combinatorics is used to derive the total number of ways in which these substituents can be ordered. The 1/3 multiplier adjusts for the fact that the parent BV has *C*_3_ symmetry, offsetting triple counting in the 
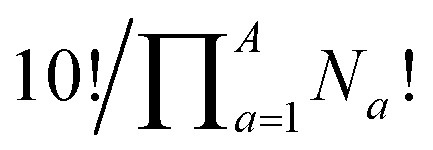
 term that arises for structures related by rotation. A further correction factor, 2*S*, readjusts for the BV isomers that have three identically substituted arms and so are represented just once each in the 
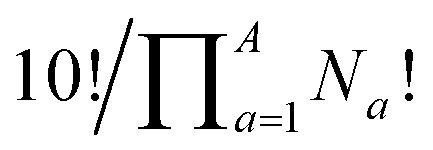
 term. *S* is the number of ways that the substituents can be arranged around the BV core to retain its *C*_3_ symmetry, or in other words, where all three arms of the BV possess identical substitution patterns.

Taking heterotrisubstituted BV 1 as a worked example (Fig. 2a), there are seven hydrogen substituents and three distinct non-hydrogen substituents (colored circles). Therefore, the product operation in the denominator of the equation is 7! × 1! × 1! × 1!. The substituents on 1 cannot be arranged in any pattern that gives *C*_3_ symmetry, hence *S* = 0 and applying eqn (1) gives *N*_iso_ = 240.

Taking structure 2 (Fig. 2b) as a second worked example, there are six hydrogens, a substituent type that occurs three times (blue circles), and another substituent type that occurs just once (orange circle), so the product operation is 6! × 3! × 1!. However, three of the substituents of 2 are the same and thus there are three different ways in which they can be arranged such that the BV possesses *C*_3_ symmetry, which are shown in Fig. 2b. These *C*_3_-symmetric isomers arise when the unique substituent is at the α position and the three identical substituents occupy the same position on each of the three BV arms, *i.e.*, either all β, all γ, or all δ. Therefore, *S* = 3 for 2 and applying eqn (1) gives *N*_iso_ = 282.

There are 42 possible variations of substituted BVs having different numbers and identities of substituents. Based on all of these possible substituent patterns, a comprehensive reference table is provided in the ESI (Table S1)† where this method and eqn (1) have been applied to generate *N*_iso_ and *S*, as well as the numbers of achiral and chiral isomers.

### Generating bullvalene isomers using *bullviso*

We have developed a Python3 code, *bullviso*,^19^ which interfaces with RDKit^20^ to generate the Cartesian coordinates of substituted BVs. It is publicly available under the GNU Public License (GPLv3) on GitLab. The *bullviso* code generates exhaustively all possible isomer barcodes for a substituted BV and filters out the non-unique isomer barcodes according to the protocol outlined by Bode.¶¶A Fortran code to generate input geometries of BV isomers is mentioned in ref. 2a but it has not been made publicly available.^17^ It also outputs the connectivity between isomers, which can be used to generate interconversion network diagrams.^21^ Cartesian coordinates sampling the constitutional isomers of the substituted BV are generated by grafting given substituents (supplied as SMILES strings) onto a BV to produce a unique structure corresponding to each isomer barcode. The *bullviso* code generates up to *N* configurational and conformational isomers according to the experimental-torsion distance geometry with ‘basic knowledge’ embedding approach (ETKDGv3)^22^ implemented in RDKit. These configurational and conformational isomers are then (pre-)optimized using the Universal Force Field^23^ and the *M* lowest-energy isomers are outputted. Cartesian coordinates can be written in *xyz* file format or, alternatively, as pre-prepared inputs for computational chemistry packages, *e.g.*, Gaussian^24^ or Orca,^25^ to enable subsequent optimization and analysis at higher levels of theory.

### Principal moments of inertia analysis

**Fig. 3:**
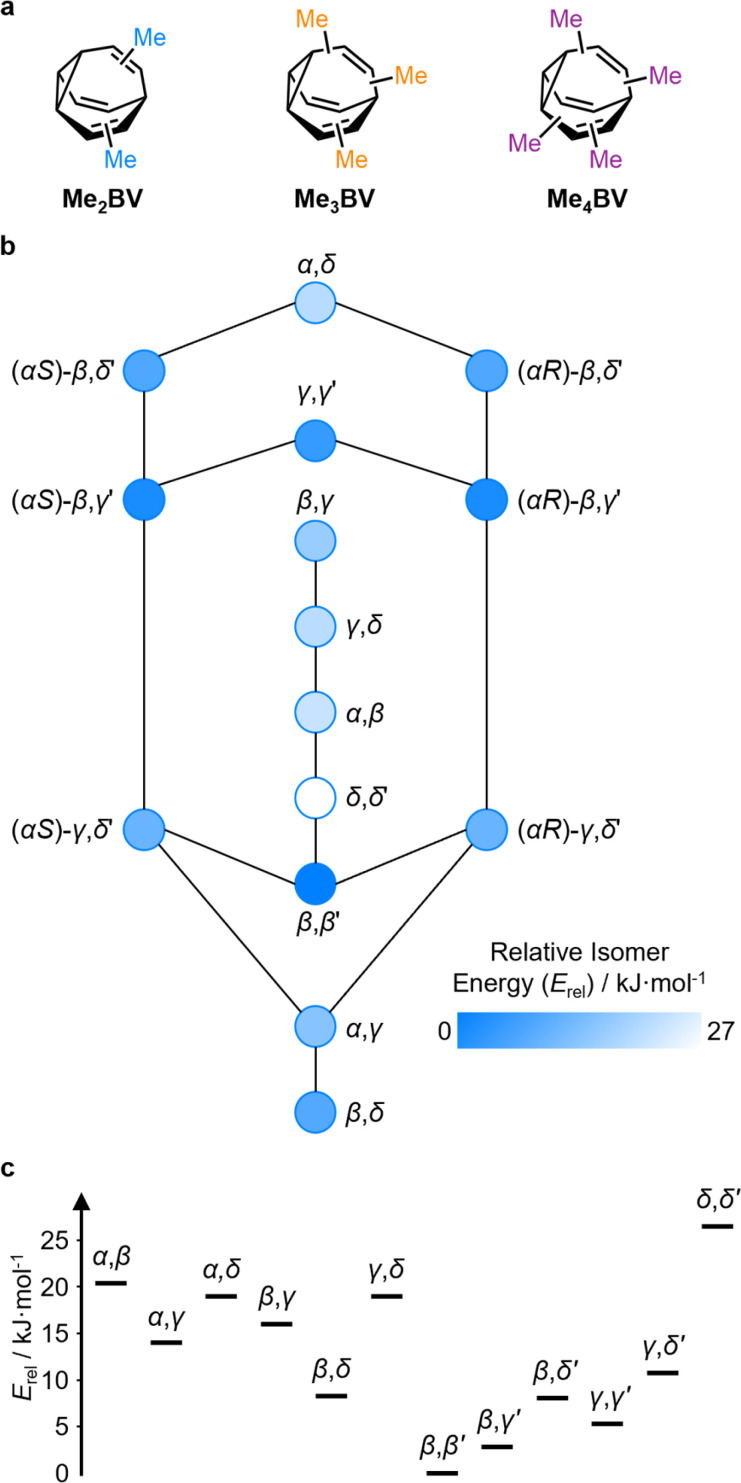
(a) Structural formulae of methyl-substituted BVs. (b) The population-weighted isomer interconversion network calculated for Me_2_BV (PBE0-D3/def2-SV(P)). The diagram has a mirror plane with achiral isomers down the middle and enantiomeric pairs of chiral structures on either side. Chiral structures are labelled with an *R*/*S* descriptor according to the stereogenic α position. (c) A graph of the relative energies of Me_2_BV isomers. Pairs of enantiomers are isoenergetic, so are represented just once.

The PMI analysis developed by Schwartz^15^ has been used as a straightforward and quantitative method to assess the shape diversity of potential pharmaceutical building blocks.^12,13^ Typical PMI plots are constructed (i) for the lowest energy conformer of any given compound to compare either the inherent shapes of an array of molecules^26^ or (ii) for a range of conformers to gain insight into conformational diversity of a limited number of molecules.^14^ We selected di-, tri-, and tetrasubstituted BVs (Fig. 3a) as targets to examine shape diversity present in dynamic BV networks arising from rapid constitutional isomerism. To enable us to focus our analysis on the dynamic shape that is inherent to the functionalized BV scaffold itself rather than any potential conformational processes in the attached substituents, we chose to investigate the methyl-substituted derivatives, *i.e.*, dimethyl- (Me_2_BV), trimethyl- (Me_3_BV), and tetramethylbullvalene (Me_4_BV).

**Fig. 4:**
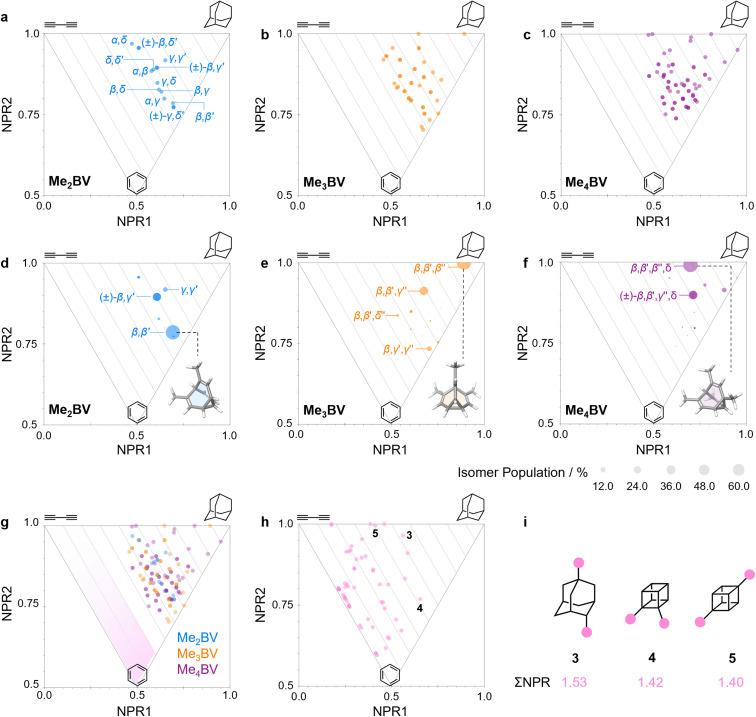
(a–c) PMI plots for the shapeshifting networks of (a) Me_2_BV, (b) Me_3_BV, and (c) Me_4_BV. Substituent positional labels are given for Me_2_BV. For clarity, these labels are not shown on the plots for Me_3_BV and Me_4_BV. See Tables S6 and S7† for labelled data. (d–e) Population-weighted PMI plots for the shapeshifting networks of (d) Me_2_BV, (e) Me_3_BV, and (f) Me_4_BV where the data points are scaled by calculated Boltzmann distributions at 298 K (PBE0-D3/def2-SV(P)). The modelled structure of the lowest-energy isomer for each BV is shown inset. (g) An overlay of the PMI plots of Me_2_BV (blue), Me_3_BV (orange), and Me_4_BV (purple) showing that none of the isomers have ΣNPR values close to the rod–disc axis. (h) A PMI plot for common ring systems. See Table S8† for compound identities. (i) Structural formulae and ΣNPR values for 1,4-dimethyladamantane (3), 1,2-dimethylcubane (4), and 1,4-dimethylcubane (5).

We first generated all possible isomers of the methyl-substituted BVs using *bullviso*, then optimized their geometries by performing density functional theory (DFT) calculations. The PBE0 (ref. 27) functional with Grimme's D3 dispersion correction^28^ and the def2-SV(P)^29^ basis set were deemed suitable for modelling the energetics of BV systems. Using this level of theory, we constructed isomer interconversion networks and predicted relative isomer populations for Me_2_BV (Fig. 3), Me_3_BV (Fig. S1†), and Me_4_BV (Fig. S2†), which have 15, 42, and 72 unique isomers, respectively.^21^ For each of these isomers, we calculated the three principal moments of inertia (*I*_1_, *I*_2_, and *I*_3_ in ascending order) using a KNIME^30^ Vernalis^31^ chemoinformatic protocol, then used these values to calculate normal PMI ratios, NPR1 (*I*_1_/*I*_3_) and NPR2 (*I*_2_/*I*_3_). The resulting PMI plots (Fig. 4a–g) follow the standard layout (Fig. 4) where the vertices are defined by NPR values associated with rod-like shape [NPR1 = 0, NPR2 = 1], disc-like shape [NPR1 = 0.5, NPR2 = 0.5], and spherical shape [NPR1 = 1, NPR2 = 1]. The diagrams are also labelled with representative structures for each vertex, *i.e.*, butadiyne (rod-like), benzene (disc-like), and adamantane (sphere-like). To guide the eye, parallel lines on the PMI plot correspond to increments of 0.1 in ∑NPR values (∑NPR = NPR1 + NPR2) between the limits of 1.0 and 2.0. The points that lie furthest from the rod–disc axis, *i.e.*, toward the top right of the diagram, are associated with greater sphericity.

The PMI plot of Me_2_BV (Fig. 4a) shows the structural diversity in the population of constitutional isomers, which fall in the range 1.44 ≤ ∑NPR ≤ 1.57. For ease of reference, isomers in Fig. 4a are labelled using the α–δ locants, however, the full isomer barcodes are also given in the ESI (Tables S2–S4).† Darker colored points indicate overlap of enantiomers on the diagram as they give identical PMI coordinates, *e.g.*, for (±)-β,γ′-Me_2_BV.

As the unique isomers of a substituted BV are non-degenerate, they are present in varying concentrations at equilibrium. To visualize how this property influences which molecular shapes are most prevalent, we made PMI plots with the data points scaled by the Boltzmann distribution at 298 K. A population-weighted PMI plot (Fig. 4d) shows that the shapeshifting network of Me_2_BV consists predominantly (∼93%) of the four lowest-energy isomers, which include two achiral isomers, β,β′-Me_2_BV and γ,γ′-Me_2_BV, and the enantiomeric pair of (±)-β,γ′-Me_2_BV. The β,δ-Me_2_BV, (±)-β,δ′-Me_2_BV, and (±)-γ,δ′-Me_2_BV isomers are also present in ∼0.5–2% each (Table S2†), leaving ∼0.3% of the remaining six isomers combined.

The introduction of more substituents to the BV scaffold (*i.e.*, in Me_3_BV and Me_4_BV) increases structural variety and overall sphericity (Fig. 4b and c). Several structures extend beyond ΣNPR > 1.6, reaching maxima of 1.89 and 1.90 for Me_3_BV and Me_4_BV, respectively. In both cases, the PMI distributions are markedly broader than that of Me_2_BV, spanning 1.3 ≤ ΣNPR ≤ 1.9, which reflects the change in shape that can occur when several substituents are located close to one another around the BV scaffold (giving rod-like shape) or are spread around the BV evenly to maintain sphericity. Like Me_2_BV, a subset of the Me_3_BV and Me_4_BV constitutional isomers are most prevalent in the network at equilibrium. The 14 most stable isomers of Me_3_BV are within ∼15 kJ mol^−1^ of one another, so they are each present in amounts ranging from 0.1% to 60% (Table S3†). Similarly, there are 12 isomers of Me_4_BV present in proportions of 0.1% to 52%.||||A consequence of there being more possible isomers in the networks of highly substituted BVs is that, statistically, any individual isomer is expected to be present in lower concentrations. However, certain BV systems, *e.g.*, hexasubstituted BVs, are biased toward a relatively small number of isomers, which act as energetic sinks that also slow the rate of exchange.^32^^32^ The energetically preferred isomers are those with most of their substituents at β and γ positions, and in which substituents do not neighbor one another directly. Therefore, the population-weighted PMI plots (Fig. 4e and f) show that the most spherical isomers are present in higher populations.

To contextualize the PMI data of the functionalized BVs, it is useful to compare the Me_2_BV isomers (Fig. 4a) to a small representative library of rings prominent in pharmaceuticals (Fig. S3†).^33^ PMI analysis of the dimethyl derivatives of these pharmaceutical building blocks (Fig. 4h) shows the relative lack of 3D diversity in most currently used ring systems.^32^ The majority of compounds are close to the rod–disc axis (ΣNPR ≤ 1.3) with the notable exception of a few structures such as substituted adamantanes and cubanes 3–5 (Fig. 4i), which are increasingly popular as more spherical replacements for flat ring systems.^34^ The PMI ratios of the Me_2_BV isomers (1.44 ≤ ∑NPR ≤ 1.57) and 3–5 (1.40 ≤ ∑NPR ≤ 1.53) are nearly identical, suggesting that BVs could be similarly useful as 3D scaffolds in medicinal chemistry. BVs have the additional, unique property of spontaneously sampling different structures through their Cope rearrangements and are now readily accessible by short synthetic routes.^1*c*,2^

### Exit vector analysis

**Fig. 5:**
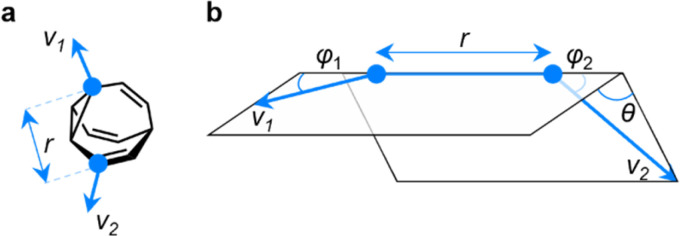
(a) The vectors *v*_1_ and *v*_2_ for two substituent attachment points on a BV (shown for (α*S*)-γ,δ′-Me_2_BV) which are defined by (b) the geometric parameters *r*, *φ*_1_, *φ*_2_, and *θ*.

EV plots are used to analyze the relationship between two substituents attached to a central scaffold.^16^ They are useful in FBDD and bioisostere studies to show the geometries that are available when elaborating a structure outward starting from different ring systems. The relative orientations (Fig. 5a) of bonds emanating from the scaffold are defined as the exit vectors, *v*_1_ and *v*_2_. They are described (Fig. 5b) by four geometric parameters; the distance between the functionalized carbon atoms of the scaffold (*r*), the dihedral angle of the vectors (*θ*), and the plane angles of each vector (*φ*_1_ and *φ*_2_).^16^ Like PMI plots, the EV plot for a single molecule possesses only one data point if a compound is conformationally rigid, whereas multiple points are plotted to show the effects of conformational flexibility or to compare multiple molecules with different covalent structures on a single diagram.

**Fig. 6:**
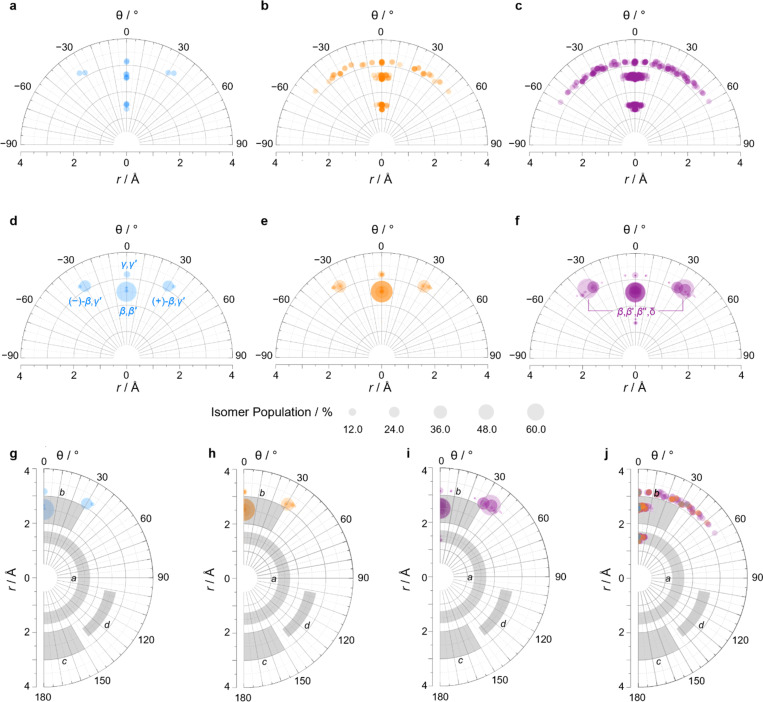
(a–c) Distance–dihedral angle EV plots and (d–i) Boltzmann population-weighted distance–dihedral angle EV plots (298 K, PBE0-D3/def2-SV(P)) for the isomers of (a, d and g) Me_2_BV, (b, e and h) Me_3_BV, and (c, f and i) Me_4_BV. (j) Overlaid distance *versus* dihedral angle EV plot of all three methyl-substituted BVs. (g–j) Plots include characteristic areas of EV plots in grey that correspond to those found in disubstituted cycloalkanes,^16^ a = *cis*-1,2-disubstituted cyclopropanes, b = *cis*-1,3-disubstituted aliphatic rings and *cis*-1,4-disubstituted cyclohexanes, c = *trans*-1,4-disubstituted cyclohexanes, d = *trans*-1,3-disubstituted cyclopentanes and cyclohexanes.

We constructed EV plots (Fig. 6) for the C–Me bonds of the methyl-substituted BVs to quantify the stereodynamics of the BV scaffold, *i.e.*, the relative orientations and spacings of its substituents. Typically, EV plots span *θ* values of 0° to 180°, where all dihedral angles are defined as being positive. For BV isomer networks, however, it is also beneficial to extend the EV plots to include negative values of *θ*, allowing pairs of enantiomers that are present in the dynamic equilibrium to be shown on the same diagram (*e.g.*, (±)-β,γ′-Me_2_BV, Fig. 6d), as well as revealing enantiotopic relationships between substituents at equivalent positions on different arms (*e.g.*, the β′ and β′′ positions of β,β′,β′′,δ-Me_4_BV, Fig. 6f). As the cage-like structure of BV imposes dihedral angles between −60° and +60°, plotting *θ* from −90° to +90° (Fig. 6a–f) gives an informative representation of the data.

An EV plot of the Me_2_BV isomers reveals C–Me dihedral angles clustered in two regions of either *θ* ∼ 0° or *θ* ∼ ±30°. The points in the former region span distances of *r* ∼ 1.3–3.1 Å (Fig. 6a). The majority of coplanar EVs (*θ* ∼ 0°) arise from isomers that are either functionalized (i) at two different positions on the same arm of the BV or (ii) at the same type of position on BV on different arms, *e.g.*, β,β′-Me_2_BV, accounting for nine of the 15 possible substitution patterns. The four points at *θ* ∼ ±30° correspond to two of the three enantiomer pairs of the isomers with differently substituted arms ((±)-β,γ′-Me_2_BV and (±)-β,δ′-Me_2_BV). The final enantiomer pair, (±)-γ,δ′-Me_2_BV, has coplanar EVs of *θ* = ±0.5°. Boltzmann population-weighted EV analysis of Me_2_BV (Fig. 6d) reveals that the most populated isomers β,β′-Me_2_BV (*r* = 2.5 Å, *θ* = 0°, *p* = 53%) and (±)-β,γ′-Me_2_BV (*r* = 3.1 Å, *θ* = 30°, *p* = 17% for each enantiomer) exhibit substantial changes in the dihedral angles between the C–Me EVs.

Standard EV plots of Me_2_BV, Me_3_BV, and Me_4_BV spanning *θ* values of 0° to 180° (Fig. 6g–j) are also shown to aid comparison to the four regions, a–d, determined by Grygorenko *et al.* that are characteristic of EVs found in common disubstituted cycloalkanes.^16^ Note that these four regions were determined by plotting the EVs found for ∼2900 cycloalkanes in the Cambridge Structural Database, so they represent the span of angles that are obtained using a variety of functional groups. The majority of data points for the BV isomers fall within the a or b region. EVs in region *a* are characteristic of *cis*-1,2-disubstituted cyclopropyl compounds while region b is associated with *cis*-1,3-disubstituted and *cis*-1,4-disubstituted aliphatic rings.^16^ The presence of EVs in these regions for BV is expected, therefore, as the structure of BV contains these motifs.

**Fig. 7:**
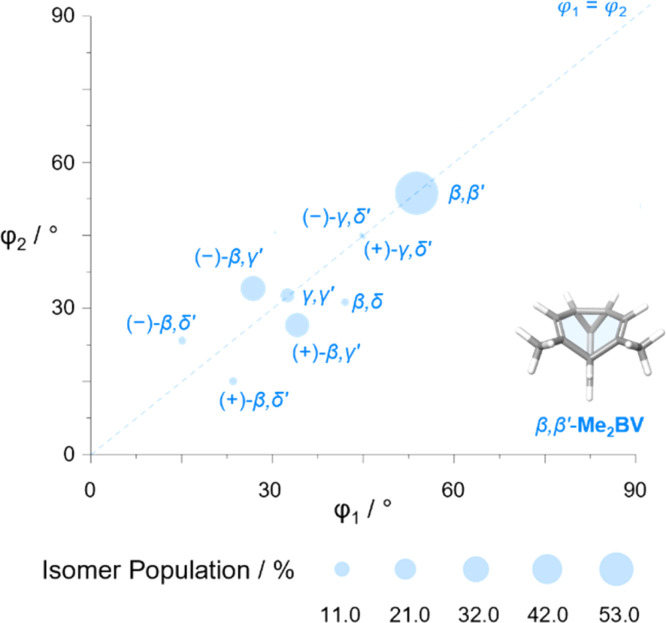
The plane angles subtended by C–Me EVs in the nine lowest energy isomers of Me_2_BV. Data points are scaled according to the Boltzmann population at 298 K (PBE0-D3/def2-SV(P)).

The diversity of EVs arising from the shapeshifting isomerization becomes even more apparent when considering the plane angles subtended by the C–Me EVs, *φ*_1_ and *φ*_2_ (Fig. 5), of the Me_2_BV isomers. There is a spread of possible plane angles spanning from 15° to 60° found in the most energetically accessible isomers (Fig. 7), extending to 67° for higher-energy isomers (Fig. S4†). Therefore, sequential Cope rearrangements between isomers significantly alter the relative orientations of substituents in space, granting BV its unique stereodynamic properties.

As the cage-like geometry of the BV scaffold is well defined and insensitive to the addition of more substituents, Me_3_BV and Me_4_BV would be expected to exhibit similar EVs between pairs of their C–Me bonds as those found for Me_2_BV. This generalization applies when considering the plane angles (Fig. S7–S14†) and distances between substituents. It is also the case for the dihedral angles, but only when considering the most energetically favorable isomers (Fig. 6e and f).

A wider spread of dihedral angles (Fig. 6b and c) that extends beyond region b to angles between −60° and +60° is apparent when the complete isomer networks of Me_3_BV and Me_4_BV are taken into account (Fig. 6j), including isomers that lie at higher energy. Significant deviations from the 0° and ±30° dihedral angles arise to minimize unfavorable steric interactions when substituents are close to one another in space. For the di-, tri-, and tetrasubstituted BVs investigated here, the isomers that bear methyl groups at neighboring positions are relatively high in energy, so are not very prevalent. The most significant (albeit still low) populations of such isomers are present for Me_4_BV because of the increased likelihood of substituents being close to one another in the tetrasubstituted system, such as the ∼0.1% of β,β′,γ,δ′-Me_4_BV, which has *θ* = 51° between its β and δ′ methyl groups. But more highly substituted BVs, or those bearing bulkier substituents, will likely have larger isomer populations with varied dihedral angles.

Of course, shape differentiation between the orientations of functional groups attached to BV diminishes if conformationally flexible linking groups are used, so elaborating BVs with short rigid groups^2*c*,8*b*,35^ or fused rings^36^ may be advantageous. Each of the molecular shapes described by the PMI (Fig. 4a–c) and EV plots (Fig. 6a–c and S4–S14†) correspond to accessible states for the methyl-substituted BVs, demonstrating that a single BV derivative can cover a significant region of chemical space on its own. Heterosubstituted systems with varied functional groups possess still greater structural diversity. Although realizing the full extent of this shape diversity in some BVs may require higher-energy isomers to be invoked, it is important to note that, in the contexts of drug discovery and materials chemistry, noncovalent bonding interactions with biomolecular targets, confinement effects, or forces imposed by the surrounding medium may compensate for the moderately low energy differences between isomers to amplify certain BV structures within the shapeshifting network.^2*d*,4,6,37^ Therefore, isomers that have otherwise low populations should not be dismissed, as they may become more significant components of the network in the right environment.

## Conclusions

In summary, the appealing complexity of dynamic BV networks is built upon four types of positional exchange between different sites around the BV scaffold. Isomers in these networks tend to position functional groups with dihedral angles of either 0° or 30° and at a range of plane angles between 15° and 60°, originating from vertices of the BV that are spaced apart by 1.3–3.1 Å. The BV scaffold itself has quasi-spherical overall structure. Functionalized derivatives generally retain this characteristic, particularly because the most energetically favored isomers that emerge are typically those that space substituents out from one another around the scaffold. PMI analysis shows that they have a similar degree of sphericity as adamantane and cubane building blocks, while they orient functional groups at vectors that are characteristic of *cis*-disubstituted cycloalkanes. Greater shape diversity emerges with the inclusion of more substituents, not just because it gives rise to greater numbers of unique isomers, but also because interactions between neighboring groups reduces the predisposition toward them being coplanar with one another, giving more varied EVs. Currently, the most accessible syntheses of BVs^1*c*,2*a*–*c*^ produce structures with two or three functional groups attached, implying it remains advantageous to develop new synthetic approaches that give efficient access to higher-order multifunctional BVs. The analysis described here, which is accelerated using *bullviso*, can be exploited to prescreen computationally the diversity and accessibility of molecular shapes in complex BV libraries.

## Data availability

Coordinates of optimized geometries, PMIs and EVs are available in the ESI.† The Python code for *bullviso* is publicly available under the GNU Public License (GPLv3) on GitLab.^19^

## Author contributions

Conceptualization: RAI, WM, PRM. Methodology: RAI, WM, CR. Software: CR. Writing: RAI, WM, MTG, CR, PRM. Funding acquisition: PRM.

## Conflicts of interest

There are no conflicts to declare.

## Supplementary Material

SC-OLF-D4SC03700F-s001

## References

[cit1] von Eggers Doering W., Roth W. R. (1963). Tetrahedron.

[cit2] Yahiaoui O., Pašteka L. F., Judeel B., Fallon T. (2018). Angew. Chem., Int. Ed..

[cit3] Sanchez A., Gurajapu A., Guo W., Kong W.-Y., Laconsay C. J., Settineri N. S., Tantillo D. J., Maimone T. J. (2023). J. Am. Chem. Soc..

[cit4] Lippert A. R., Keleshian V. L., Bode J. W. (2009). Org. Biomol. Chem..

[cit5] Dohmen C., Ihmels H., Paululat T. (2022). Eur. J. Org Chem..

[cit6] Birvé A. P., Patel H. D., Price J. R., Bloch W. M., Fallon T. (2022). Angew. Chem., Int. Ed..

[cit7] Reimers J. R., Li T., Birvé A. P., Yang L., Aragonès A. C., Fallon T., Kosov D. S., Darwish N. (2023). Nat. Commun..

[cit8] Tantillo D. J., Hoffmann R. (2006). Acc. Chem. Res..

[cit9] Ottonello A., Wyllie J. A., Yahiaoui O., Sun S., Koelln R. A., Homer J. A., Johnson R. M., Murray E., Williams P., Bolla J. R., Robinson C. V., Fallon T., Soares da Costa T. P., Moses J. E. (2023). Proc. Natl. Acad. Sci. U.S.A..

[cit10] Baumann A. E., Burns D. A., Liu B., Thoi V. S. (2019). Commun. Chem..

[cit11] Lin R.-B., Chen B. (2022). Chem.

[cit12] Erlanson D. A., Fesik S. W., Hubbard R. E., Jahnke W., Jhoti H. (2016). Nat. Rev. Drug Discovery.

[cit13] Hung A. W., Ramek A., Wang Y., Kaya T., Wilson J. A., Clemons P. A., Young D. W. (2011). Proc. Natl. Acad. Sci. U.S.A..

[cit14] Downes T. D., Jones S. P., Klein H. F., Wheldon M. C., Atobe M., Bond P. S., Firth J. D., Chan N. S., Waddelove L., Hubbard R. E., Blakemore D. C., De Fusco C., Roughley S. D., Vidler L. R., Whatton M. A., Woolford A. J. A., Wrigley G. L., O'Brien P. (2020). Chem.–Eur. J..

[cit15] Sauer W. H. B., Schwarz M. K. (2003). J. Chem. Inf. Comput. Sci..

[cit16] Grygorenko O. O., Demenko D., Volochnyuk D. M., V Komarov I. (2018). New J. Chem..

[cit17] He M., Bode J. W. (2013). Org. Biomol. Chem..

[cit18] Favre H. A. and Powell W. H., Nomenclature of Organic Chemistry: IUPAC Recommendations and Preferred Names 2013, IUPAC Blue book, RSC Publishing, 2014

[cit19] bullviso, 2024, https://gitlab.com/conorrankine/bullviso

[cit20] (a) RDKit: Open-Source Cheminformatics, https://rdkit.org

[cit21] Gimarc B. M., Brant A. R. (1994). J. Chem. Inf. Comput. Sci..

[cit22] Riniker S., Landrum G. A. (2015). J. Chem. Inf. Model..

[cit23] Rappe A. K., Casewit C. J., Colwell K. S., Goddard W. A., Skiff W. M. (1992). J. Am. Chem. Soc..

[cit24] Frisch M. J. , Trucks G. W., Schlegel H. B., Scuseria G. E., Robb M. A., Cheeseman J. R., Scalmani G., Barone V., Petersson G. A., Nakatsuji H., Li X., Caricato M., Marenich A. V., Bloino J., Janesko B. G., Gomperts R., Mennucci B., Hratchian H. P., Ortiz J. V., Izmaylov A. F., Sonnenberg J. L., Williams-Young D., Ding F., Lipparini F., Egidi F., Goings J., Peng B., Petrone A., Henderson T., Ranasinghe D., Zakrzewski V. G., Gao J., Rega N., Zheng G., Liang W., Hada M., Ehara M., Toyota K., Fukuda R., Hasegawa J., Ishida M., Nakajima T., Honda Y., Kitao O., Nakai H., Vreven T., Throssell K., Montgomery Jr J. A., Peralta J. E., Ogliaro F., Bearpark M. J., Heyd J. J., Brothers E. N., Kudin K. N., Staroverov V. N., Keith T. A., Kobayashi R., Normand J., Raghavachari K., Rendell A. P., Burant J. C., Iyengar S. S., Tomasi J., Cossi M., Millam J. M., Klene M., Adamo C., Cammi R., Ochterski J. W., Martin R. L., Morokuma K., Farkas O., Foresman J. B. and Fox D. J., Gaussian, Gaussian, Inc., Wallingford CT, 2016

[cit25] Neese F. (2012). Wiley Interdiscip. Rev. Comput. Mol. Sci..

[cit26] Jones S. P., Firth J. D., Wheldon M. C., Atobe M., Hubbard R. E., Blakemore D. C., De Fusco C., Lucas S. C. C., Roughley S. D., Vidler L. R., Whatton M. A., Woolford A. J. A., Wrigley G. L., O'Brien P. (2022). RSC Med. Chem..

[cit27] Adamo C., Barone V. (1999). J. Chem. Phys..

[cit28] Grimme S., Antony J., Ehrlich S., Krieg H. (2010). J. Chem. Phys..

[cit29] Weigend F., Ahlrichs R. (2005). Phys. Chem. Chem. Phys..

[cit30] Berthold M. R. , Cebron N., Dill F., Gabriel T. R., Kötter T., Meinl T., Ohl P., Sieb C., Thiel K. and Wiswedel B., KNIME: The Konstanz Information Miner, Springer, 2007

[cit31] Roughley S. D. (2018). Curr. Med. Chem..

[cit32] Rebsamen K., Röttele H., Schröder G. (1993). Chem. Ber..

[cit33] Taylor R. D., MacCoss M., Lawson A. D. G. (2014). J. Med. Chem..

[cit34] Chalmers B. A., Xing H., Houston S., Clark C., Ghassabian S., Kuo A., Cao B., Reitsma A., Murray C. E. P., Stok J. E., Boyle G. M., Pierce C. J., Littler S. W., Winkler D. A., Bernhardt P. V., Pasay C., De Voss J. J., McCarthy J., Parsons P. G., Walter G. H., Smith M. T., Cooper H. M., Nilsson S. K., Tsanaktsidis J., Savage G. P., Williams C. M. (2016). Angew. Chem., Int. Ed..

[cit35] Bismillah A. N., Sturala J., Chapin B. M., Yufit D. S., Hodgkinson P., McGonigal P. R. (2018). Chem. Sci..

[cit36] Schröder G., Witt W. (1979). Angew Chem. Int. Ed. Engl..

[cit37] Bismillah A. N., Johnson T. G., Hussein B. A., Turley A. T., Saha P. K., Wong H. C., Aguilar J. A., Yufit D. S., McGonigal P. R. (2023). Nat. Chem..

