# Supplementary material for: A guide to bullvalene stereodynamics
Source: Chem Sci. 2024 Aug 23;15(36):14608–17. doi: 10.1039/d4sc03700f (PMC11358867; doi:10.1039/d4sc03700f)
Supplement: SC-015-D4SC03700F-s001 [file SC-015-D4SC03700F-s001.pdf]

Supplementary Information (SI) for Chemical Science.
This journal is © The Royal Society of Chemistry 2024

A guide to bullvalene stereodynamics

Robert A. Ives,[a,b] William Maturi,[a,b] Matthew T. Gill,[a] Conor Rankine,*[a] and Paul R. McGonigal*[a]

[a] *Department of Chemistry, University of York, Heslington, York, YO10 5DD (UK)*

[b] *Department of Chemistry, Durham University, Lower Mountjoy, Stockton Road, Durham, DH1 3LE (UK)*

E-mail: conor.rankine@york.ac.uk; paul.mcgonigal@york.ac.uk

Table of Contents

1. General Calculation Details and Nomenclature S2
2. Isomer Populations S4
3. Principal Moments of Inertia Analysis S9
4. Exit Vector Analysis S13
5. Supplemental Exit Vector Plots S23
6. DFT-Optimized Geometries S29
7. References S188

Supporting Information

1. General Calculation Details and Nomenclature

Isomer Generation

All initial sets of Cartesian coordinates sampling the constitutional isomers of **Me_2BV**, **Me_3BV**, and **Me_4BV** were generated *via* the in-house-developed *bullviso*[19] code. *bullviso* is publicly available under the GNU Public License (GPLv3) on GitLab. All constitutional isomers were subsequently (pre-)optimized at the GFN2-xTB[S1] (extended tight binding; xTB) level of theory using *xtb* (v6.4.1).[S2] An SCF convergence criterion of $1.0{\times}10^{-6}$ a.u. was used with convergence criteria of $5.0{\times}10^{-6}$ and $1.0{\times}10^{-3}$ a.u. for the energy change and gradient, respectively, in all geometry optimizations. All constitutional isomers verified at the GFN2-xTB level of theory were progressed to DFT geometry optimization.

DFT Geometry Optimization

All DFT geometry optimizations and energy evaluations of the constitutional isomers of **Me_2BV**, **Me_3BV**, and **Me_4BV** were carried out at the PBE0-D3 level of theory (*i.e.* with the PBE0[28] density functional of Adamo and Barone coupled with the D3[28] dispersion correction of Grimme *et al.*) using ORCA (v5.0.2).[25] All calculations were carried out under the resolution-of-identity (RI) approximation for the Coulomb integrals (RIJONX). A tightened SCF convergence criterion of $1.0{\times}10^{-9}$ a.u. was used in all calculations; convergence criteria of $2.0{\times}10^{-7}$ and $3.0{\times}10^{-5}$ a.u. were used for the energy change and gradient, respectively, in all geometry optimizations. The def2-SV(P)[29] basis set of Weigend and Ahlrichs was coupled with the def2/J[S3] auxilliary basis set; the two were used together throughout. The proper convergence of all geometry optimizations to real minima was verified *via* vibrational frequency inspection. Enantiomers were optimized independently. As exact mirror-image geometries were not generated by the optimization, the energies, PMIs, and EVs are not identical.

Numbers of Unique BV Permutations

Varying the number of different substituents, there are 42 possible bullvalene systems, which are listed in Table S1 below. The total number of unique isomers, N_{iso}, is calculated according to Equation 1. We further divide N_{iso} into the number of achiral and chiral isomers. To do so, we counted the number of isomers where at least two of the arms are identically substituted. The presence of these two identical arms gives a symmetrical (achiral) structure with a mirror plane bisecting the molecule parallel to the third arm. Each of the possible systems with at least two identical arms are determined, and combinatorics performed with the remaining four substituents to determine all the ways in which they may be organized. For example, in the case of 0000000112, there are four systems with two identical arms, ("000 000", "001 001", "010 010", and "100 100"). In the first system, the remaining substituents are 0112, so there are ($\frac{4!}{\prod N_A!} = \frac{4!}{2!}$) 12 different isomers possible that have two 000 arms. For the three remaining

cases, the four substituents left over are 0002 so there are ($\frac{4!}{\prod N_A!} = \frac{4!}{3!}$) four different isomers possible for each of the 001, 010, and 100 cases. Summing these together makes 24 achiral isomers of the 120 total possible unique isomers of 0000000112.

Table S1. All possible BV systems, including the numbers of unique substituents R^x, the total number of isomers N_{iso}, the symmetry term *S*, and the number of achiral and chiral isomers.

Occurrences of Each Substituent Type										Substituent System	*S*	N_{iso}	Achiral	Chiral
R^0	R^1	R^2	R^3	R^4	R^5	R^6	R^7	R^8	R^9					
10	0	0	0	0	0	0	0	0	0	0000000000	1	1	1	0
9	1	0	0	0	0	0	0	0	0	0000000001	1	4	4	0
8	1	1	0	0	0	0	0	0	0	0000000012	0	30	12	18
8	2	0	0	0	0	0	0	0	0	0000000011	0	15	9	6
7	1	1	1	0	0	0	0	0	0	0000000123	0	240	24	216
7	2	1	0	0	0	0	0	0	0	0000000112	0	120	24	96
7	3	0	0	0	0	0	0	0	0	0000000111	3	42	16	26
6	1	1	1	1	0	0	0	0	0	0000001234	0	1680	24	1656
6	2	1	1	0	0	0	0	0	0	0000001123	0	840	48	792
6	2	2	0	0	0	0	0	0	0	0000001122	0	420	48	372
6	3	1	0	0	0	0	0	0	0	0000001112	3	282	40	242
6	4	0	0	0	0	0	0	0	0	0000001111	3	72	22	50
5	1	1	1	1	1	0	0	0	0	0000012345	0	10080	0	10080
5	2	1	1	1	0	0	0	0	0	0000011234	0	5040	72	4968
5	2	2	1	0	0	0	0	0	0	0000011223	0	2520	96	2424
5	3	1	1	0	0	0	0	0	0	0000011123	0	1680	72	1608
5	3	2	0	0	0	0	0	0	0	0000011122	0	840	96	744
5	4	1	0	0	0	0	0	0	0	0000011112	0	420	48	372
5	5	0	0	0	0	0	0	0	0	0000011111	0	84	24	60
4	1	1	1	1	1	1	0	0	0	0000123456	0	50400	0	50400
4	2	1	1	1	1	0	0	0	0	0000112345	0	25200	72	25128
4	2	2	1	1	0	0	0	0	0	0000112234	0	12600	144	12456
4	2	2	2	0	0	0	0	0	0	0000112233	0	6300	168	6132
4	3	1	1	1	0	0	0	0	0	0000111234	0	8400	72	8328
4	3	2	1	0	0	0	0	0	0	0000111223	0	4200	120	4080
4	3	3	0	0	0	0	0	0	0	0000111222	6	1404	96	1308
4	4	1	1	0	0	0	0	0	0	0000111123	0	2100	72	2028
4	4	2	0	0	0	0	0	0	0	0000111122	0	1050	48	1002
3	1	1	1	1	1	1	1	0	0	0001234567	0	201600	0	201600
3	2	1	1	1	1	1	0	0	0	0001123456	0	100800	0	100800
3	2	2	1	1	1	0	0	0	0	0001122345	0	50400	144	50256
3	2	2	2	1	0	0	0	0	0	0001122334	0	25200	240	24960
3	3	1	1	1	1	0	0	0	0	0001112345	0	33600	0	33600
3	3	2	1	1	0	0	0	0	0	0001112234	0	16800	144	16656
3	3	2	2	0	0	0	0	0	0	0001112233	0	8400	168	8232
3	3	3	1	0	0	0	0	0	0	0001112223	6	5604	144	5460
2	1	1	1	1	1	1	1	1	0	0012345678	0	604800	0	604800
2	2	1	1	1	1	1	1	0	0	0011234567	0	302400	0	302400
2	2	2	1	1	1	1	0	0	0	0011223456	0	151200	144	151056
2	2	2	2	1	1	0	0	0	0	0011223345	0	75600	288	75312
2	2	2	2	2	0	0	0	0	0	0011223344	0	37800	360	37140
1	1	1	1	1	1	1	1	1	1	0123456789	0	1209600	0	1209600

Stereochemical Assignment and Priority Rules

M and *P* stereochemical descriptors have been used previously by Fallon[S4] to distinguish BV enantiomers from one another. The assignment requires a viewing direction to be assigned to the BV C_{3v} axis, which has been as running in the direction from the cyclopropyl to the α position. We prefer to use of point chirality of the α position to assign an *R* or *S* stereochemical descriptor as this can be done using the established Cahn-Ingold-Prelog (CIP) priority rules without needing to define a direction for an additional axis. The α position of BV is the highest priority sp^3-hybridized carbon and only unique position on the BV structure.

It is also useful to assign priority to the arms of the BV to determine which should be labelled without a prime symbol, with a single prime symbol ('), or with a double prime symbol ("). Priority can also be assigned to the three arms using the CIP priority rules. Substituents on the highest priority bridge are given with no prime symbol, substituents on the second priority bridge are given with a single prime symbol and substituents the lowest priority bridge are given with a double prime symbol. The positional labels for each of the isomers for **Me_2BV**, **Me_3BV** and **Me_4BV** are presented in Tables S5, S6, and S7 respectively, including stereochemical descriptors for the chiral isomers.

2. Isomer Populations

Table S2. Energy (*E*), Relative energies (E_{rel}), and Boltzmann distribution population percentage (*P* / %) for **Me_2BV**.

Isomer	Positional Label	*E* / Hartree	E_{rel} / kJ·mol^{-1}	*P* / %
000 000 001 1	α,β	−464.735969	20.59	0.013
000 000 010 1	α,γ	−464.738464	14.04	0.183
000 000 011 0	β,γ	−464.737758	15.89	0.087
000 000 100 1	α,δ	−464.736512	19.16	0.023
000 000 101 0	β,δ	−464.740612	8.40	1.777
000 000 110 0	γ,δ	−464.736491	19.22	0.023
000 001 001 0	β,β'	−464.74381	0.00	52.646
000 001 010 0	(α*R*)-β,γ'	−464.742721	2.86	16.607
000 001 100 0	(α*R*)-β,δ'	−464.740664	8.26	1.880
000 010 001 0	(α*S*)-β,γ'	−464.742780	2.70	17.714
000 010 010 0	γ,γ'	−464.741728	5.47	5.794
000 010 100 0	(α*R*)-γ,δ'	−464.739697	10.80	0.675
000 100 001 0	(α*S*)-β,δ'	−464.740682	8.21	1.918
000 100 010 0	(α*S*)-γ,δ'	−464.739675	10.86	0.659
000 100 100 0	δ,δ'	−464.733729	26.47	0.001

Table S3. Energy (*E*), Relative energies (E_{rel}), and Boltzmann distribution population percentage (*P* / %) for **Me_3BV**.

Isomer	Positional Label	*E* / Hartree	E_{rel} / kJ·mol^{-1}	*P* / %
000 000 011 1	α,β,γ	−503.968652	43.80	0.000
000 000 101 1	α,β,δ	−503.974248	29.11	0.000
000 000 110 1	α,γ,δ	−503.973677	30.61	0.000
000 000 111 0	β,γ,δ	−503.970754	38.29	0.000
000 001 001 1	α,β,β'	−503.972914	32.61	0.000

000 001 010 1	(α*R*)-α,β,γ'	−503.976285	23.76	0.004
000 001 011 0	(α*S*)-β,β',γ	−503.979301	15.85	0.101
000 001 100 1	(α*R*)-α,β,δ'	−503.974363	28.81	0.001
000 001 101 0	(α*S*)-β,β',δ	−503.982174	8.3	2.118
000 001 110 0	(α*R*)-β,γ',δ'	−503.978096	19.01	0.028
000 010 001 1	(α*S*)-α,β,γ'	−503.976318	23.68	0.004
000 010 010 1	α,γ,γ'	−503.978773	17.23	0.058
000 010 011 0	(α*S*)-β,γ,γ'	−503.978373	18.28	0.038
000 010 100 1	(α*R*)-α,γ,δ'	−503.976846	22.29	0.007
000 010 101 0	(α*S*)-β,γ',δ	−503.981157	10.97	0.721
000 010 110 0	(α*S*)-γ,γ',δ	−503.976996	21.9	0.009
000 011 001 0	(α*R*)-β,β',γ	−503.979298	15.85	0.101
000 011 010 0	(α*R*)-β,γ,γ'	−503.978334	18.38	0.036
000 011 100 0	(α*R*)-β,γ,δ'	−503.976206	23.97	0.004
000 100 001 1	(α*S*)-α,β,δ'	−503.974408	28.69	0.001
000 100 010 1	(α*S*)-α,γ,δ'	−503.976816	22.37	0.007
000 100 011 0	(α*S*)-β,γ,δ'	−503.976229	23.91	0.004
000 100 100 1	α,δ,δ'	−503.971059	37.48	0.000
000 100 101 0	(α*S*)-β,δ,δ'	−503.975111	26.85	0.001
000 100 110 0	(α*S*)-γ,δ,δ'	−503.969699	41.05	0.000
000 101 001 0	(α*R*)-β,β',δ	−503.98216	8.34	2.084
000 101 010 0	(α*R*)-β,γ',δ	−503.981121	11.07	0.693
000 101 100 0	(α*R*)-β,δ,δ'	−503.975151	26.74	0.001
000 110 001 0	(α*S*)-β,γ',δ'	−503.978071	19.07	0.027
000 110 010 0	(α*R*)-γ,γ',δ	−503.976989	21.91	0.009
000 110 100 0	(α*R*)-γ,δ,δ'	−503.969765	40.88	0.000
001 001 001 0	β,β',β''	−503.985336	0	60.266
001 001 010 0	β,β',γ''	−503.984343	2.61	21.028
001 001 100 0	β,β',δ''	−503.982227	8.16	2.241
001 010 010 0	β,γ',γ''	−503.98326	5.45	6.687
001 010 100 0	(α*R*)-β,γ',δ''	−503.981171	10.93	0.733
001 100 010 0	(α*S*)-β,γ',δ''	−503.981155	10.98	0.718
001 100 100 0	β,δ',δ''	−503.975147	26.75	0.001
010 010 010 0	γ,γ',γ''	−503.982133	8.41	2.026
010 010 100 0	γ,γ',δ''	−503.980126	13.68	0.242
010 100 100 0	γ,δ',δ''	−503.974214	29.2	0.000
100 100 100 0	δ,δ',δ''	−503.965015	53.35	0.000

Table S4. Energy (*E*), Relative energies (E_{rel}), and Boltzmann distribution population percentage (*P* / %) for **Me_4BV**.

Isomer	Positional Label	*E* / Hartree	E_{rel} / kJ·mol^{-1}	*P* / %
000 000 111 1	α,β,γ,δ	−543.201058	59.60	0.000
000 001 011 1	(α*S*)-α,β,β',γ	−543.205509	47.92	0.000
000 001 101 1	(α*S*)-α,β,β',δ	−543.211306	32.70	0.000
000 001 110 1	(α*R*)-α,β,γ',δ'	−543.211751	31.53	0.000
000 001 111 0	(α*S*)-β,β',γ,δ	−543.212477	29.63	0.000
000 010 011 1	(α*S*)-α,β,γ,γ'	−543.209119	38.44	0.000
000 010 101 1	(α*S*)-α,β,γ',δ	−543.21468	23.84	0.003
000 010 110 1	(α*S*)-α,γ,γ',δ	−543.214078	25.42	0.002
000 010 111 0	(α*S*)-β,γ,γ',δ	−543.211407	32.43	0.000
000 011 001 1	(α*R*)-α,β,β',γ	−543.205524	47.88	0.000
000 011 010 1	(α*R*)-α,β,γ,γ'	−543.209146	38.37	0.000
000 011 011 0	β,β',γ,γ'	−543.215144	22.62	0.006
000 011 100 1	(α*R*)-α,β,γ,δ'	−543.207151	43.61	0.000
000 011 101 0	(α*R*)-β,β',γ,δ'	−543.217729	15.84	0.086
000 011 110 0	(α*R*)-β,γ,γ',δ'	−543.21377	26.23	0.001
000 100 011 1	(α*S*)-α,β,γ,δ'	−543.207204	43.47	0.000
000 100 101 1	(α*S*)-α,β,δ,δ'	−543.209013	38.72	0.000
000 100 110 1	(α*S*)-α,γ,δ,δ'	−543.206971	44.08	0.000

000 100 111 0	(α*S*)-β,γ,δ,δ'	−543.203636	52.84	0.000
000 101 001 1	(α*R*)-α,β,β',δ	−543.211278	32.77	0.000
000 101 010 1	(α*R*)-α,β,γ',δ	−543.214731	23.71	0.004
000 101 011 0	(α*S*)-β,β',γ,δ'	−543.217733	15.83	0.087
000 101 100 1	(α*R*)-α,β,δ,δ'	−543.209013	38.72	0.000
000 101 101 0	β,β',δ,δ'	−543.216658	18.65	0.028
000 101 110 0	(α*R*)-β,γ',δ,δ'	−543.211289	32.74	0.000
000 110 001 1	(α*S*)-α,β,γ',δ'	−543.211756	31.52	0.000
000 110 010 1	(α*R*)-α,γ,γ',δ	−543.21409	25.39	0.002
000 110 011 0	(α*S*)-β,γ,γ',δ'	−543.213744	26.30	0.001
000 110 100 1	(α*R*)-α,γ,δ,δ'	−543.206983	44.05	0.000
000 110 101 0	(α*S*)-β,γ',δ,δ'	−543.211245	32.86	0.000
000 110 110 0	γ,γ',δ,δ'	−543.205433	48.12	0.000
000 111 001 0	(α*R*)-β,β',γ,δ	−543.212485	29.60	0.000
000 111 010 0	(α*R*)-β,γ,γ',δ	−543.211438	32.35	0.000
000 111 100 0	(α*R*)-β,γ,δ,δ'	−543.203646	52.81	0.000
001 001 001 1	α,β,β',β''	−543.207757	42.02	0.000
001 001 010 1	α,β,β',γ''	−543.213382	27.25	0.001
001 001 011 0	β,β',β'',γ	−543.220959	7.36	2.645
001 001 100 1	α,β,β',δ''	−543.211517	32.15	0.000
001 001 101 0	β,β',β'',δ	−543.223761	0.00	51.514
001 001 110 0	β,β',γ'',δ''	−543.219802	10.39	0.779
001 010 010 1	α,β,γ',γ''	−543.216707	18.52	0.029
001 010 011 0	(α*R*)-β,β',γ,γ''	−543.220027	9.80	0.988
001 010 100 1	(α*R*)-α,β,γ',δ''	−543.214856	23.38	0.004
001 010 101 0	(α*R*)-β,β',γ'',δ	−543.222765	2.61	17.974
001 010 110 0	(α*S*)-β,γ',γ'',δ'	−543.218583	13.59	0.214
001 011 010 0	(α*S*)-β,β',γ,γ''	−543.219986	9.91	0.946
001 011 100 0	(α*S*)-β,β',γ,δ''	−543.217838	15.55	0.097
001 100 010 1	(α*S*)-α,β,γ',δ''	−543.21479	23.55	0.004
001 100 011 0	(α*R*)-β,β',γ,δ''	−543.217797	15.66	0.093
001 100 100 1	α,β,δ',δ''	−543.208991	38.78	0.000
001 100 101 0	(α*R*)-β,β',δ,δ''	−543.216676	18.60	0.028
001 100 110 0	(α*S*)-β,γ',δ',δ''	−543.211294	32.73	0.000
001 101 010 0	(α*S*)-β,β',γ'',δ	−543.222754	2.64	17.758
001 101 100 0	(α*S*)-β,β',δ'',δ	−543.216628	18.73	0.027
001 110 010 0	(α*R*)-β,γ',γ'',δ'	−543.2186	13.55	0.218
001 110 100 0	(α*R*)-β,γ',δ',δ''	−543.211265	32.81	0.000
010 010 010 1	α,γ,γ',γ''	−543.219091	12.26	0.366
010 010 011 0	β,γ,γ',γ''	−543.218926	12.69	0.308
010 010 100 1	α,γ,γ',δ''	−543.217194	17.24	0.049
010 010 101 0	β,γ',γ'',δ	−543.221662	5.51	5.579
010 010 110 0	γ,γ',γ'',δ	−543.217491	16.46	0.067
010 011 100 0	(α*S*)-β,γ,γ',δ''	−543.21686	18.12	0.034
010 100 011 0	(α*R*)-β,γ,γ',δ''	−543.216835	18.18	0.034
010 100 100 1	α,γ,δ',δ''	−543.211495	32.20	0.000
010 100 101 0	(α*R*)-β,γ',δ,δ''	−543.21566	21.27	0.010
010 100 110 0	(α*R*)-γ,γ',δ,δ''	−543.210233	35.52	0.000
010 101 100 0	(α*S*)-β,γ',δ,δ''	−543.21567	21.24	0.010
010 110 100 0	(α*S*)-γ,γ',δ,δ''	−543.210282	35.39	0.000
011 100 100 0	β,γ,δ',δ''	−543.210698	34.30	0.000
100 100 100 1	α,δ,δ',δ''	−543.202482	55.87	0.000
100 100 101 0	β,δ,δ',δ''	−543.206421	45.52	0.000
100 100 110 0	γ,δ,δ',δ''	−543.199442	63.85	0.000

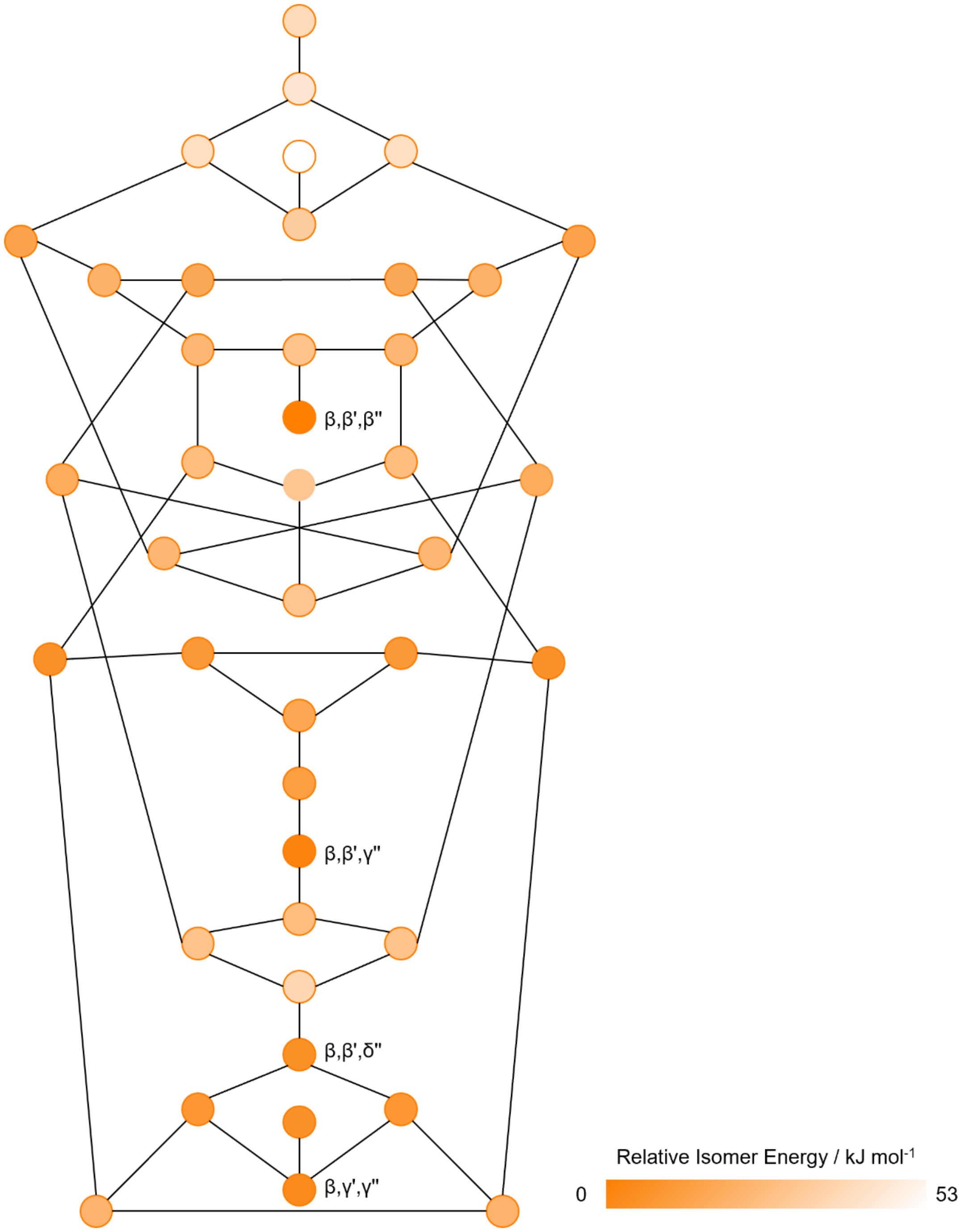

Figure S1. The energy-weighted isomer interconversion network calculated for **Me_3BV** (PBE0-D3/ def2-SV(P)).[21]

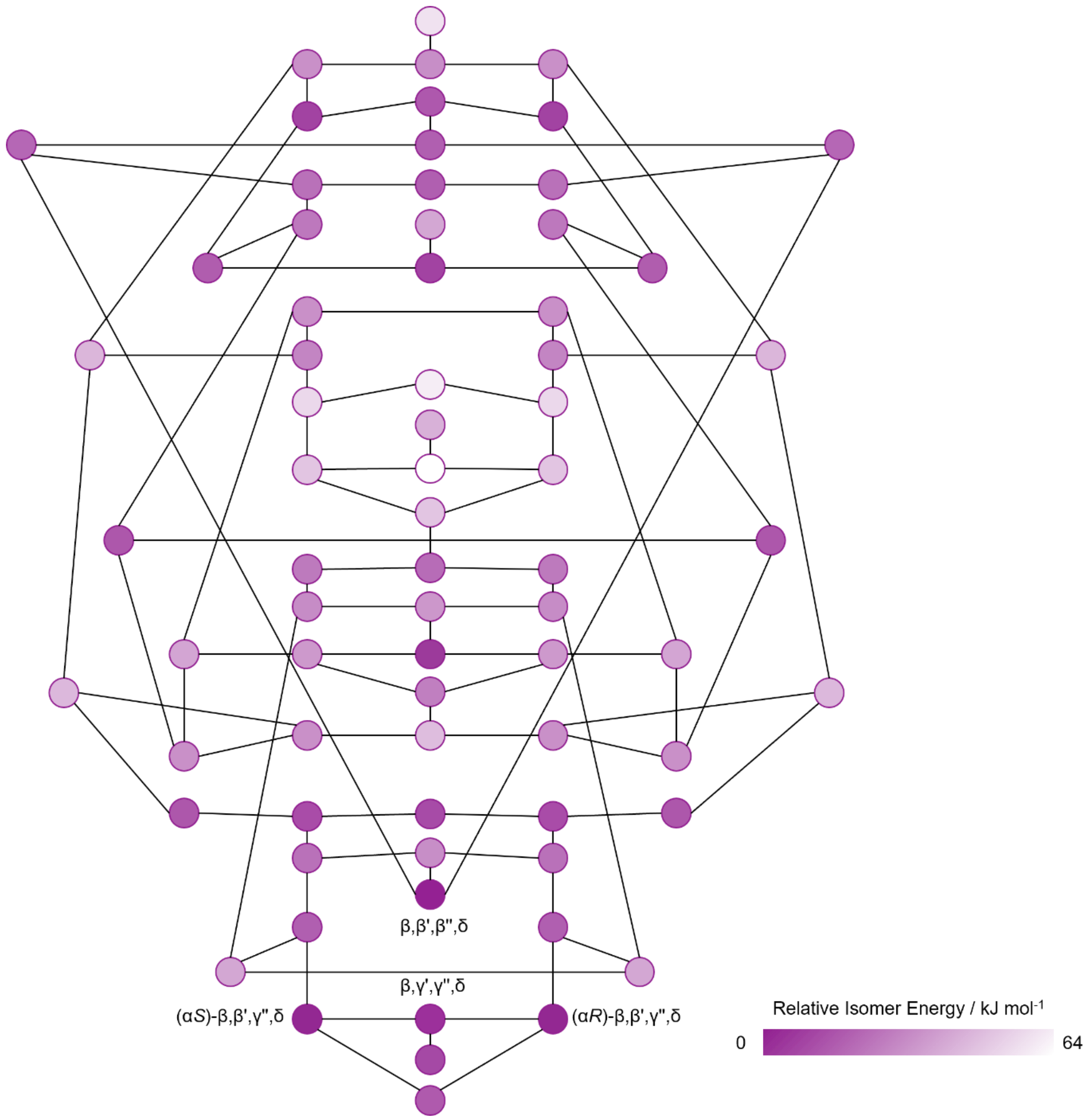

Figure S2. The energy-weighted isomer interconversion network calculated for **Me_4BV** (PBE0-D3/ def2-SV(P)).[21]

3. Principal Moments of Inertia Analysis

PMI Generation

3−D structures of compounds were generated using Vernalis KNIME nodes v1.34.1 and RDKit v4.5.0 in KNIME v4.4.1, based upon the Vernalis example workflow (https://hub.knime.com/vernalis/spaces/Public/latest/PMI%20Plotting%20Example~zuyv2AxB_dFVs0Ym, accessed November 2022). DFT optimised (ωB97M−V functional, def2−SV(P) basis set) were imported into the KNIME workflow *via* an SDF reader. A maximum of 30 conformers were generated for each molecule, using experimental torsions and 'basic knowledge', i.e., flat aromatic rings etc. The geometry of each was optimised using MMFF94 force field with 1000 iterations. Prior to calculation, explicit hydrogens were added. Post calculation, explicit hydrogens were kept and the lowest energy conformer of each molecule was selected. Normalised principal moments of inertia (PMI) values (NPR1 and NPR2) were then derived and plotted on a 2D PMI plot in Microsoft Excel 2023.

Table S5. PMI generation of normalised principal moments of inertia (NPR1 and NPR2) for **Me_2BV**.

Isomer	NPR1	NPR2
000 000 001 1	0.579451568	0.884053394
000 000 010 1	0.648490572	0.798908463
000 000 011 0	0.633008369	0.820297516
000 000 100 1	0.473739491	0.968402290
000 000 101 0	0.619009668	0.826640516
000 000 110 0	0.611689113	0.847342749
000 001 001 0	0.694590202	0.785185086
000 001 010 0	0.609014942	0.894473552
000 001 100 0	0.510598953	0.955380037
000 010 001 0	0.609010310	0.894471363
000 010 010 0	0.654156352	0.917353765
000 010 100 0	0.698308763	0.772691736
000 100 001 0	0.510591262	0.955397691
000 100 010 0	0.698304702	0.772702604
000 100 100 0	0.590581046	0.888824493

Table S6. PMI generation of normalised principal moments of inertia (NPR1 and NPR2) for **Me_3BV**.

Isomer	NPR1	NPR2
000 000 011 1	0.662971310	0.712488254
000 000 101 1	0.516496035	0.846370614
000 000 110 1	0.528833382	0.832863467
000 000 111 0	0.670738372	0.703398101
000 001 001 1	0.652346910	0.839580720
000 001 010 1	0.625540139	0.890769526
000 001 011 0	0.669654028	0.773255566
000 001 100 1	0.473135465	0.930891427
000 001 101 0	0.607636684	0.848361464
000 001 110 0	0.546430483	0.919287752
000 010 001 1	0.625540963	0.890759877
000 010 010 1	0.746792262	0.793448936
000 010 011 0	0.647718778	0.955680574
000 010 100 1	0.593820861	0.800836680

000 010 101 0	0.711872896	0.819256412
000 010 110 0	0.674476176	0.866517628
000 011 001 0	0.669658721	0.773251710
000 011 010 0	0.647723374	0.955691226
000 011 100 0	0.570785225	0.822385486
000 100 001 1	0.473128508	0.930888432
000 100 010 1	0.593814445	0.800843681
000 100 011 0	0.570770626	0.822386315
000 100 100 1	0.457564827	0.919742134
000 100 101 0	0.546856878	0.869661457
000 100 110 0	0.677790124	0.753007295
000 101 001 0	0.607639268	0.848363075
000 101 010 0	0.711872514	0.819246991
000 101 100 0	0.546848992	0.869664043
000 110 001 0	0.546438566	0.919289540
000 110 010 0	0.674467592	0.866524433
000 110 100 0	0.677798780	0.753011319
001 001 001 0	0.892975057	0.999995568
001 001 010 0	0.676531071	0.912325595
001 001 100 0	0.536304976	0.836139627
001 010 010 0	0.706239380	0.733588598
001 010 100 0	0.605649669	0.794098332
001 100 010 0	0.605648288	0.794101934
001 100 100 0	0.507145821	0.956196177
010 010 010 0	0.753482798	0.753485031
010 010 100 0	0.768961471	0.857654514
010 100 100 0	0.723964861	0.880883726
100 100 100 0	0.646586940	0.999996888

Table S7. PMI generation of normalised principal moments of inertia (NPR1 and NPR2) for **Me_4BV**.

Isomer	NPR1	NPR2
000 000 111 1	0.563051215	0.751696738
000 001 011 1	0.674686669	0.775069595
000 001 101 1	0.555183858	0.873884216
000 001 110 1	0.524845860	0.906436938
000 001 111 0	0.612853060	0.786923960
000 010 011 1	0.687754380	0.824019790
000 010 101 1	0.630662518	0.821485164
000 010 110 1	0.656495218	0.800477250
000 010 111 0	0.710933870	0.808541740
000 011 001 1	0.674685300	0.775066330
000 011 010 1	0.687750916	0.824010651
000 011 011 0	0.650410620	0.846078320
000 011 100 1	0.555926360	0.784486640
000 011 101 0	0.684549860	0.772347480
000 011 110 0	0.601847940	0.905901900
000 100 011 1	0.555921690	0.784492220
000 100 101 1	0.474779760	0.869858100
000 100 110 1	0.546817392	0.804077475
000 100 111 0	0.600965544	0.768555240
000 101 001 1	0.555190288	0.873874502
000 101 010 1	0.684566608	0.772346810
000 101 011 0	0.684562483	0.772343273
000 101 100 1	0.474779337	0.869866035
000 101 101 0	0.587400463	0.823499121
000 101 110 0	0.618288850	0.841957250
000 110 001 1	0.524854429	0.906432500
000 110 010 1	0.656492730	0.800476170

000 110 011 0	0.601839460	0.905906190
000 110 100 1	0.546813110	0.804079130
000 110 101 0	0.618282800	0.841953650
000 110 110 0	0.731480740	0.790645070
000 111 001 0	0.612864740	0.786922310
000 111 010 0	0.710928400	0.808529240
000 111 100 0	0.600962510	0.768545750
001 001 001 1	0.809209990	0.999993550
001 001 010 1	0.697889580	0.979044680
001 001 011 0	0.777979461	0.928502653
001 001 100 1	0.517980060	0.882348820
001 001 101 0	0.700049140	0.989860770
001 001 110 0	0.587054020	0.950107760
001 010 010 1	0.727553751	0.831528016
001 010 011 0	0.723262451	0.795699963
001 010 100 1	0.579461478	0.852400313
001 010 101 0	0.714882263	0.897453794
001 010 110 0	0.657390165	0.798637152
001 011 010 0	0.723267062	0.795699518
001 011 100 0	0.620258594	0.739152709
001 100 010 1	0.579467520	0.852404580
001 100 011 0	0.620257270	0.739153250
001 100 100 1	0.471633060	0.997681370
001 100 101 0	0.551907470	0.868170480
001 100 110 0	0.575908430	0.834141260
001 101 010 0	0.714899330	0.897457410
001 101 100 0	0.551914366	0.868172360
001 110 010 0	0.657386370	0.798639360
001 110 100 0	0.575907431	0.834135051
010 010 010 1	0.950846377	0.950854008
010 010 011 0	0.720698637	0.844085604
010 010 100 1	0.745382690	0.839053390
010 010 101 0	0.881862400	0.912984280
010 010 110 0	0.799755990	0.861150800
010 011 100 0	0.663252290	0.841042480
010 100 011 0	0.663252420	0.841039070
010 100 100 1	0.594341440	0.883316360
010 100 101 0	0.682183630	0.857493310
010 100 110 0	0.775420660	0.874045650
010 101 100 0	0.682188570	0.857492320
010 110 100 0	0.775414600	0.874038310
011 100 100 0	0.579943440	0.901650508
100 100 100 1	0.492922242	0.999997408
100 100 101 0	0.555813522	0.939994105
100 100 110 0	0.710702857	0.812443988

Table S8. PMI generation of normalised principal moments of inertia (NPR1 and NPR2) for 3D and common ring systems

Compound	NPR1	NPR2
1,4−dimethyladamantane	0.560813614	0.965283098
1,2−dimethylcubane	0.654554766	0.768061524
1,4−dimethylcubane	0.386724171	0.999990450
p−xylene	0.216879256	0.797921538
o−xylene	0.416028907	0.600580363
m−xylene	0.336329337	0.679090911
1,4−dimethylpiperazine	0.248330953	0.816390622
(1*S*,2*S*)−1,2−dimethylcyclobutane	0.479827723	0.650785997
(1*R*,3*R*)−1,3−dimethylcyclobutane	0.279549544	0.919459774

(1*R*,2*S*)−1,2−dimethylcyclobutane	0.553180758	0.671158082
(1*S*,3*S*)−1,3−dimethylcyclobutane	0.255843764	0.914094127
1*R*,4*R*,6*R*)−2,6−dimethylbicyclo[2.2.1]hept−2−ene	0.482384578	0.813203336
1,3−dimethylazetidine	0.268972148	0.915622214
1,2−dimethylazetidine	0.487557647	0.646360335
2,5−dimethylpyridine	0.216330750	0.798840434
2,3−dimethylpyridine	0.422075200	0.594999842
2,4−dimethylpyridine	0.328569016	0.687151837
3,4−dimethylpyridine	0.414936199	0.601949465
1,4−dimethylpiperidine	0.259618894	0.813581832
2,6−dimethyl−2−azaspiro[3.3]heptane	0.174720498	0.998096293
2,5−dimethyloctahydrocyclopenta[*c*]pyrrole	0.285180132	0.931848441
3,6−dimethyl−3−azabicyclo[3.1.0]hexane	0.412421426	0.995728438
1,5−dimethylazocane	0.437109773	0.813556375
2,6−dimethyl−2,6−diazaspiro[3.3]heptane	0.177391885	0.999617791
1,6−dimethyl−1,6−diazaspiro[3.3]heptane	0.309926511	0.891222204
(*R*)−1,7−dimethyl−1,7−diazaspiro[4.4]nonane	0.359787851	0.901014064
2,5−dimethyl−2,5−diazaspiro[3.4]octane	0.338226173	0.895946912
(1*S*,4*S*)−2,5−dimethyl−2,5−diazabicyclo[2.2.1]heptane	0.587353006	0.910167407
(1*S*,3*S*)−1,3−dimethylcyclohexane	0.453685408	0.758132605
(1*R*,2*R*)−1,2−dimethylcyclopropane	0.299772204	0.862461341
(1*s*,4*s*)−1,4−dimethylcyclohexane	0.400440242	0.856168260

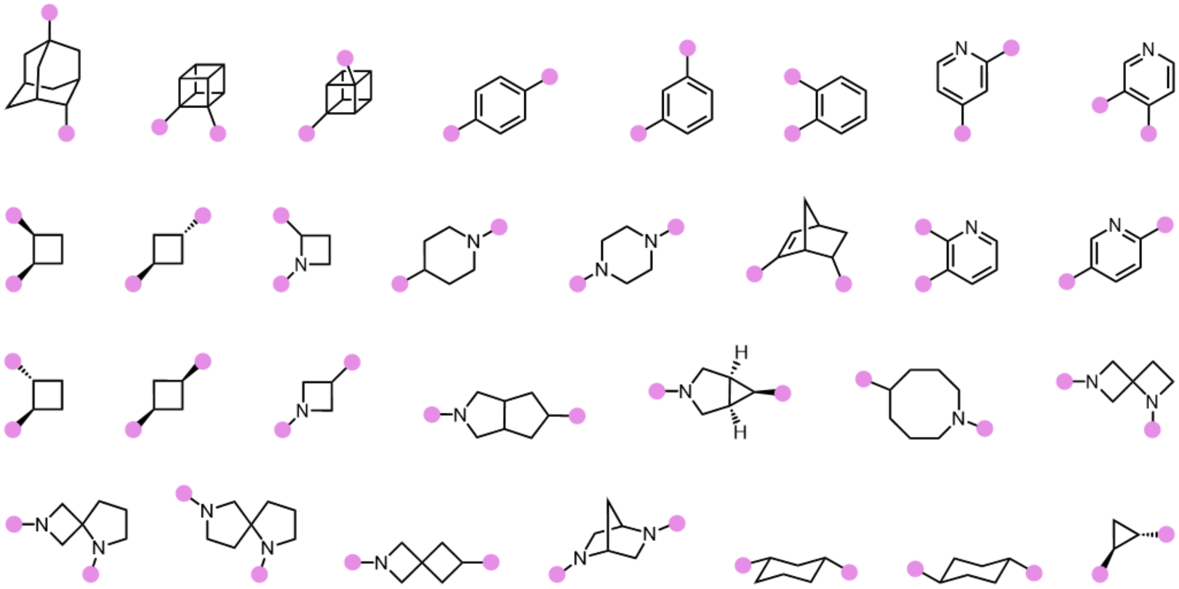

Figure S3: The commonly used ring systems in medicinal chemistry that are compared to the methylated bullvalenes in Figure 4 of the main text. See Section 6 of the SI for cartesian coordinates of their calculated geometries.

4. Exit Vector Analysis

Table S9. Isomer codes, Energy (E) and Relative energies (E_{rel}), Dipole moment (μ) and Geometric parameters r, φ_1, φ_2, and θ for **Me_2BV**. v_1 and v_2 correspond to positions to which the vectors are appended.

Isomer	E / Hartree	E_{rel} / kJ·mol^{-1}	v_1	v_2	r / Å	φ_1 / °	φ_2 / °	θ / °	μ / D
000 000 001 1	−464.735969	20.59	β	α	1.537	61.0	67.4	−0.2	0.311
000 000 010 1	−464.738464	14.04	γ	α	2.554	30.4	45.6	0.2	0.755
000 000 011 0	−464.737758	15.89	γ	β	1.353	56.0	55.1	−0.1	0.477
000 000 100 1	−464.736512	19.16	δ	α	3.122	12.4	16.9	−0.1	0.609
000 000 101 0	−464.740612	8.40	δ	β	2.544	42.0	31.3	−0.2	0.302
000 000 110 0	−464.736491	19.22	δ	γ	1.494	63.8	63.0	−0.3	0.727
000 001 001 0	−464.743810	0.00	β'	β	2.509	53.8	53.7	−0.2	0.126
000 001 010 0	−464.742721	2.86	β	γ'	3.14	34.1	26.6	30.0	0.399
000 001 100 0	−464.740664	8.26	β	δ'	3.237	15.1	23.4	33.4	0.258
000 010 001 0	−464.742780	2.70	γ'	β	3.139	26.8	34.1	−30.0	0.399
000 010 010 0	−464.741728	5.47	γ'	γ	3.166	32.5	32.7	0.2	0.774
000 010 100 0	−464.739697	10.80	γ	δ'	2.662	45.0	44.7	−0.5	0.654
000 100 001 0	−464.740682	8.21	δ'	β	3.236	23.5	15.1	−33.3	0.257
000 100 010 0	−464.739675	10.86	δ'	γ	2.662	44.7	45.1	0.2	0.656
000 100 100 0	−464.733729	26.47	δ'	δ	1.542	61.3	61.4	0.0	0.566

Table S10. Isomer codes, Energy (E) and Relative energies (E_{rel}), Dipole moment (μ) and Geometric parameters r, φ_1, φ_2, and θ for **Me_3BV**. v_1 and v_2 correspond to positions to which the vectors are appended.

Isomer	E / Hartree	E_{rel} / kJ·mol^{-1}	v_1	v_2	r / Å	φ_1 / °	φ_2 / °	θ / °	μ / D
000 000 011 1	−503.968652	43.80	γ	β	1.357	59.0	59.3	−0.4	0.544
000 000 011 1	−503.968652	43.80	γ	α	2.544	28.3	40.4	0.4	0.544
000 000 011 1	−503.968652	43.80	β	α	1.542	63.3	67.1	0.7	0.544
000 000 101 1	−503.974248	29.11	δ	β	2.561	42.6	34.6	−0.2	0.365
000 000 101 1	−503.974248	29.11	δ	α	3.114	13.2	12.2	0.1	0.365
000 000 101 1	−503.974248	29.11	β	α	1.535	61.1	67.2	0.2	0.365
000 000 110 1	−503.973677	30.61	δ	γ	1.492	63.6	63.1	0.1	0.833
000 000 110 1	−503.973677	30.61	δ	α	3.12	8.8	17.8	0.1	0.833
000 000 110 1	−503.973677	30.61	γ	α	2.569	33.7	46.2	−0.2	0.833
000 000 111 0	−503.970754	38.29	δ	γ	1.505	63.8	65.9	0.2	0.549
000 000 111 0	−503.970754	38.29	δ	β	2.534	37.6	25.7	0.0	0.549
000 000 111 0	−503.970754	38.29	γ	β	1.356	58.7	54.9	−0.1	0.549
000 001 001 1	−503.972914	32.61	β'	β	2.492	52.6	52.4	−0.2	0.209
000 001 001 1	−503.972914	32.61	β'	α	1.539	61.1	68.6	−7.8	0.209
000 001 001 1	−503.972914	32.61	β	α	1.539	61.1	68.6	7.6	0.209
000 001 010 1	−503.976285	23.76	β	γ'	3.143	32.1	27.4	23.2	0.457
000 001 010 1	−503.976285	23.76	β	α	1.536	60.9	67.4	0.9	0.457
000 001 010 1	−503.976285	23.76	γ'	α	2.563	30.8	47.7	5.9	0.457
000 001 011 0	−503.979301	15.85	β'	γ	3.132	35.3	25.4	39.4	0.318
000 001 011 0	−503.979301	15.85	β'	β	2.524	54.2	56.9	−2.6	0.318
000 001 011 0	−503.979301	15.85	γ	β	1.353	56.0	55.2	−0.3	0.318
000 001 100 1	−503.974363	28.81	β	δ'	3.258	16.7	23.6	−21.0	0.316
000 001 100 1	−503.974363	28.81	β	α	1.536	61.0	67.3	−0.6	0.316
000 001 100 1	−503.974363	28.81	δ'	α	3.134	12.2	19.4	−13.9	0.316
000 001 101 0	−503.982174	8.30	β'	δ	3.221	15.5	23.6	−34.0	0.179
000 001 101 0	−503.982174	8.30	β'	β	2.504	53.4	53.8	−0.2	0.179
000 001 101 0	−503.982174	8.30	δ	β	2.54	41.8	31.1	0.4	0.179
000 001 110 0	−503.978096	19.01	β	δ'	3.248	15.1	24.2	42.6	0.471
000 001 110 0	−503.978096	19.01	β	γ'	3.15	34.1	28.4	23.0	0.471
000 001 110 0	−503.978096	19.01	δ'	γ'	1.493	63.7	63.0	0.2	0.471
000 010 001 1	−503.976318	23.68	γ'	β	3.144	27.3	32.3	23.0	0.457

000 010 001 1	−503.976318	23.68	γ'	α	2.563	30.8	47.6	5.6	0.457
000 010 001 1	−503.976318	23.68	β	α	1.536	60.9	67.5	0.6	0.457
000 010 010 1	−503.978773	17.23	γ'	γ	3.151	32.7	32.8	0.0	0.892
000 010 010 1	−503.978773	17.23	γ'	α	2.55	30.1	45.6	0.8	0.892
000 010 010 1	−503.978773	17.23	γ	α	2.55	30.1	45.6	−0.6	0.892
000 010 011 0	−503.978373	18.28	γ'	γ	3.18	32.4	34.7	7.1	0.560
000 010 011 0	−503.978373	18.28	γ'	β	3.161	26.2	36.1	−36.2	0.560
000 010 011 0	−503.978373	18.28	γ	β	1.352	55.9	55.1	0.1	0.560
000 010 100 1	−503.976846	22.29	γ	δ'	2.658	44.8	44.6	−0.8	0.762
000 010 100 1	−503.976846	22.29	γ	α	2.55	30.3	45.4	0.8	0.762
000 010 100 1	−503.976846	22.29	δ'	α	3.11	12.1	16.8	−0.5	0.762
000 010 101 0	−503.981157	10.97	γ'	δ	2.655	44.6	44.7	−0.9	0.408
000 010 101 0	−503.981157	10.97	γ'	β	3.124	26.6	34.2	−29.8	0.408
000 010 101 0	−503.981157	10.97	δ	β	2.54	41.9	31.0	0.4	0.408
000 010 110 0	−503.976996	21.90	γ'	δ	2.673	45.3	46.5	3.2	0.828
000 010 110 0	−503.976996	21.90	γ'	γ	3.158	33.5	31.5	−6.8	0.828
000 010 110 0	−503.976996	21.90	δ	γ	1.494	63.8	62.9	0.4	0.828
000 011 001 0	−503.979298	15.85	γ	β	1.352	56.0	55.1	0.2	0.310
000 011 001 0	−503.979298	15.85	γ	β'	3.134	25.2	35.0	−39.6	0.310
000 011 001 0	−503.979298	15.85	β	β'	2.527	56.8	53.9	2.4	0.310
000 011 010 0	−503.978334	18.38	γ	β	1.352	55.9	55.1	0.3	0.560
000 011 010 0	−503.978334	18.38	γ	γ'	3.18	34.7	32.3	7.0	0.560
000 011 010 0	−503.978334	18.38	β	γ'	3.161	36.0	26.2	−36.4	0.560
000 011 100 0	−503.976206	23.97	γ	β	1.352	56.1	55.0	0.3	0.456
000 011 100 0	−503.976206	23.97	γ	δ'	2.679	48.6	45.3	2.7	0.456
000 011 100 0	−503.976206	23.97	β	δ'	3.241	13.7	24.3	−50.8	0.456
000 100 001 1	−503.974408	28.69	δ'	β	3.258	23.6	16.6	−20.7	0.315
000 100 001 1	−503.974408	28.69	δ'	α	3.134	12.2	19.5	−13.5	0.315
000 100 001 1	−503.974408	28.69	β	α	1.536	61.0	67.3	−0.9	0.315
000 100 010 1	−503.976816	22.37	δ'	γ	2.657	44.7	44.9	−0.8	0.763
000 100 010 1	−503.976816	22.37	δ'	α	3.11	12.1	16.8	0.2	0.763
000 100 010 1	−503.976816	22.37	γ	α	2.55	30.2	45.4	0.5	0.763
000 100 011 0	−503.976229	23.91	δ'	γ	2.68	45.3	48.6	−2.7	0.457
000 100 011 0	−503.976229	23.91	δ'	β	3.241	24.3	14.0	51.1	0.457
000 100 011 0	−503.976229	23.91	γ	β	1.352	56.1	55.0	0.0	0.457
000 100 100 1	−503.971059	37.48	δ'	δ	1.54	61.4	61.4	−0.1	0.670
000 100 100 1	−503.971059	37.48	δ'	α	3.132	15.3	16.7	12.5	0.670
000 100 100 1	−503.971059	37.48	δ	α	3.133	15.4	16.7	−12.3	0.670
000 100 101 0	−503.975111	26.85	δ'	δ	1.54	61.2	61.5	−0.1	0.324
000 100 101 0	−503.975111	26.85	δ'	β	3.228	20.1	16.1	−25.8	0.324
000 100 101 0	−503.975111	26.85	δ	β	2.554	44.6	31.5	−5.3	0.324
000 100 110 0	−503.969699	41.05	δ'	δ	1.548	61.1	63.4	−2.7	0.750
000 100 110 0	−503.969699	41.05	δ'	γ	2.645	40.2	42.5	3.6	0.750
000 100 110 0	−503.969699	41.05	δ	γ	1.498	65.7	62.8	6.7	0.750
000 101 001 0	−503.982160	8.34	δ	β	2.54	41.8	31.2	−0.2	0.183
000 101 001 0	−503.982160	8.34	δ	β'	3.22	23.7	15.7	34.1	0.183
000 101 001 0	−503.982160	8.34	β	β'	2.504	53.7	53.6	0.0	0.183
000 101 010 0	−503.981121	11.07	δ	β	2.539	41.9	31.0	−0.4	0.407
000 101 010 0	−503.981121	11.07	δ	γ'	2.655	44.8	44.6	1.1	0.407
000 101 010 0	−503.981121	11.07	β	γ'	3.124	34.3	26.5	29.8	0.407
000 101 100 0	−503.975151	26.74	δ	β	2.554	44.6	31.6	5.0	0.323
000 101 100 0	−503.975151	26.74	δ	δ'	1.539	61.5	61.2	−0.1	0.323
000 101 100 0	−503.975151	26.74	β	δ'	3.229	15.9	20.2	25.3	0.323
000 110 001 0	−503.978071	19.07	δ'	γ'	1.493	63.7	63.0	0.3	0.470
000 110 001 0	−503.978071	19.07	δ'	β	3.249	24.2	15.0	42.2	0.470
000 110 001 0	−503.978071	19.07	γ'	β	3.151	28.3	34.0	22.9	0.470
000 110 010 0	−503.976989	21.91	δ	γ	1.493	63.8	62.9	0.3	0.830
000 110 010 0	−503.976989	21.91	δ	γ'	2.673	46.5	45.4	3.5	0.830
000 110 010 0	−503.976989	21.91	γ	γ'	3.156	31.6	33.7	−6.8	0.830
000 110 100 0	−503.969765	40.88	δ	γ	1.498	65.7	62.9	6.6	0.750

000 110 100 0	−503.969765	40.88	δ	δ'	1.548	63.4	61.1	−2.6	0.750
000 110 100 0	−503.969765	40.88	γ	δ'	2.645	42.5	40.1	3.6	0.750
001 001 001 0	−503.985336	0.00	β''	β'	2.513	53.6	53.5	−0.2	0.248
001 001 001 0	−503.985336	0.00	β''	β	2.515	53.3	53.3	0.0	0.248
001 001 001 0	−503.985336	0.00	β'	β	2.513	53.5	53.6	0.1	0.248
001 001 010 0	−503.984343	2.61	β	β'	2.504	54.2	54.1	0.1	0.144
001 001 010 0	−503.984343	2.61	β	γ''	3.143	33.8	26.5	30.4	0.144
001 001 010 0	−503.984343	2.61	β'	γ''	3.142	33.9	26.6	−30.3	0.144
001 001 100 0	−503.982227	8.16	β	β'	2.51	53.8	54.0	0.4	0.051
001 001 100 0	−503.982227	8.16	β	δ'	3.236	14.7	23.2	−34.2	0.051
001 001 100 0	−503.982227	8.16	β'	δ'	3.237	14.3	23.3	33.3	0.051
001 010 010 0	−503.983260	5.45	β	γ'	3.137	34.5	27.1	30.6	0.505
001 010 010 0	−503.983260	5.45	β	γ''	3.136	34.7	27.0	−30.3	0.505
001 010 010 0	−503.983260	5.45	γ'	γ''	3.166	32.6	32.7	−0.2	0.505
001 010 100 0	−503.981171	10.93	β	γ'	3.143	34.1	26.4	29.9	0.380
001 010 100 0	−503.981171	10.93	β	δ''	3.233	15.3	23.5	−35.1	0.380
001 010 100 0	−503.981171	10.93	γ'	δ''	2.663	44.9	44.7	0.0	0.380
001 100 010 0	−503.981155	10.98	β	δ''	3.233	15.3	23.5	35.4	0.380
001 100 010 0	−503.981155	10.98	β	γ'	3.143	34.1	26.3	−30.0	0.380
001 100 010 0	−503.981155	10.98	δ''	γ'	2.663	44.8	45.0	−0.2	0.380
001 100 100 0	−503.975147	26.75	β	δ'	3.248	15.3	26.2	25.3	0.296
001 100 100 0	−503.975147	26.75	β	δ''	3.247	15.3	26.2	−25.5	0.296
001 100 100 0	−503.975147	26.75	δ'	δ''	1.544	61.4	61.4	0.1	0.296
010 010 010 0	−503.982133	8.41	γ''	γ'	3.166	33.1	33.0	0.1	0.876
010 010 010 0	−503.982133	8.41	γ''	γ	3.165	33.2	33.0	0.0	0.876
010 010 010 0	−503.982133	8.41	γ'	γ	3.163	33.3	33.3	0.0	0.876
010 010 100 0	−503.980126	13.68	γ	γ'	3.172	32.2	32.4	−0.1	0.761
010 010 100 0	−503.980126	13.68	γ	δ''	2.663	45.2	44.9	−0.4	0.761
010 010 100 0	−503.980126	13.68	γ'	δ''	2.663	45.1	44.9	0.1	0.761
010 100 100 0	−503.974214	29.20	γ	δ'	2.668	45.4	45.8	6.2	0.696
010 100 100 0	−503.974214	29.20	γ	δ''	2.669	45.2	45.7	−6.6	0.696
010 100 100 0	−503.974214	29.20	δ'	δ''	1.545	61.4	61.4	0.1	0.696
100 100 100 0	−503.965015	53.35	δ''	δ'	1.541	61.0	61.0	0.1	0.609
100 100 100 0	−503.965015	53.35	δ''	δ	1.541	61.0	61.0	0.0	0.609
100 100 100 0	−503.965015	53.35	δ'	δ	1.541	61.1	61.0	0.1	0.609

Table S11. Isomer codes, Energy (*E*) and Relative energies (E_{rel}), Dipole moment (*μ)* and Geometric parameters *r*, $φ_1$, $φ_2$, and θ for **Me_4BV**. v_1 and v_2 correspond to positions to which the vectors are appended.

Isomer	*E* / Hartree	E_{rel} / kJ·mol^{-1}	v_1	v_2	*r* / Å	$φ_1$ / °	$φ_2$ / °	θ / °	*μ* / D
000 000 111 1	−543.201058	59.60	δ	γ	1.505	63.3	67.6	−0.5	0.626
000 000 111 1	−543.201058	59.60	δ	β	2.558	37.8	29.0	−0.2	0.626
000 000 111 1	−543.201058	59.60	δ	α	3.097	8.0	11.0	−0.5	0.626
000 000 111 1	−543.201058	59.60	γ	β	1.363	58.6	57.4	0.3	0.626
000 000 111 1	−543.201058	59.60	γ	α	2.559	28.2	39.9	0.7	0.626
000 000 111 1	−543.201058	59.60	β	α	1.546	65.8	66.4	0.1	0.626
000 001 011 1	−543.205509	47.92	β'	γ	3.118	35.5	27.6	−27.2	0.440
000 001 011 1	−543.205509	47.92	β'	β	2.500	52.7	56.4	0.3	0.440
000 001 011 1	−543.205509	47.92	β'	α	1.538	60.8	69.2	8.6	0.440
000 001 011 1	−543.205509	47.92	γ	β	1.357	58.5	59.5	3.4	0.440
000 001 011 1	−543.205509	47.92	γ	α	2.552	28.1	42.2	−10.2	0.440
000 001 011 1	−543.205509	47.92	β	α	1.544	63.6	68.4	−10.7	0.440
000 001 101 1	−543.211306	32.70	β'	δ	3.231	19.4	24.2	22.7	0.250
000 001 101 1	−543.211306	32.70	β'	β	2.489	52.2	52.3	0.0	0.250
000 001 101 1	−543.211306	32.70	β'	α	1.538	61.0	68.6	−7.3	0.250
000 001 101 1	−543.211306	32.70	δ	β	2.559	42.3	35.2	−2.5	0.250
000 001 101 1	−543.211306	32.70	δ	α	3.127	13.1	14.8	21.5	0.250
000 001 101 1	−543.211306	32.70	β	α	1.537	61.2	68.5	7.3	0.250
000 001 110 1	−543.211751	31.53	β	δ'	3.272	16.1	24.2	−28.7	0.522

000 001 110 1	−543.211751	31.53	β	γ'	3.158	31.5	28.7	−16.5	0.522
000 001 110 1	−543.211751	31.53	β	α	1.534	60.7	67.4	−2.1	0.522
000 001 110 1	−543.211751	31.53	δ'	γ'	1.490	63.4	63.1	−0.5	0.522
000 001 110 1	−543.211751	31.53	δ'	α	3.132	8.7	20.3	−13.6	0.522
000 001 110 1	−543.211751	31.53	γ'	α	2.578	34.0	48.3	−5.0	0.522
000 001 111 0	−543.212477	29.63	β'	δ	3.225	15.3	24.8	−47.3	0.386
000 001 111 0	−543.212477	29.63	β'	γ	3.150	35.3	26.3	−30.9	0.386
000 001 111 0	−543.212477	29.63	β'	β	2.515	53.8	58.5	3.0	0.386
000 001 111 0	−543.212477	29.63	δ	γ	1.503	63.7	65.9	−0.6	0.386
000 001 111 0	−543.212477	29.63	δ	β	2.531	37.5	25.7	1.6	0.386
000 001 111 0	−543.212477	29.63	γ	β	1.356	58.6	55.0	1.2	0.386
000 010 011 1	−543.209119	38.44	γ'	γ	3.156	32.9	33.1	5.5	0.603
000 010 011 1	−543.209119	38.44	γ'	β	3.161	27.1	32.1	−29.4	0.603
000 010 011 1	−543.209119	38.44	γ'	α	2.559	30.4	48.5	−6.4	0.603
000 010 011 1	−543.209119	38.44	γ	β	1.357	58.8	59.3	0.7	0.603
000 010 011 1	−543.209119	38.44	γ	α	2.540	28.0	40.4	−2.5	0.603
000 010 011 1	−543.209119	38.44	β	α	1.541	63.1	67.3	−2.3	0.603
000 010 101 1	−543.214680	23.84	γ'	δ	2.642	44.5	44.5	−0.9	0.475
000 010 101 1	−543.214680	23.84	γ'	β	3.126	27.4	32.7	−22.7	0.475
000 010 101 1	−543.214680	23.84	γ'	α	2.560	30.7	47.4	−5.5	0.475
000 010 101 1	−543.214680	23.84	δ	β	2.557	42.5	34.4	0.0	0.475
000 010 101 1	−543.214680	23.84	δ	α	3.103	13.0	12.2	−0.8	0.475
000 010 101 1	−543.214680	23.84	β	α	1.535	61.0	67.3	−0.2	0.475
000 010 110 1	−543.214078	25.42	γ'	δ	2.668	45.3	46.4	−3.4	0.951
000 010 110 1	−543.214078	25.42	γ'	γ	3.145	33.5	31.5	6.7	0.951
000 010 110 1	−543.214078	25.42	γ'	α	2.542	29.8	45.3	−1.2	0.951
000 010 110 1	−543.214078	25.42	δ	γ	1.492	63.7	63.0	−0.4	0.951
000 010 110 1	−543.214078	25.42	δ	α	3.109	8.6	17.7	−0.1	0.951
000 010 110 1	−543.214078	25.42	γ	α	2.566	33.4	46.2	0.9	0.951
000 010 111 0	−543.211407	32.43	γ'	δ	2.667	44.5	47.5	−3.2	0.601
000 010 111 0	−543.211407	32.43	γ'	γ	3.185	32.6	32.1	−0.5	0.601
000 010 111 0	−543.211407	32.43	γ'	β	3.139	25.8	36.8	39.0	0.601
000 010 111 0	−543.211407	32.43	δ	γ	1.504	63.9	65.8	−1.3	0.601
000 010 111 0	−543.211407	32.43	δ	β	2.530	37.6	25.5	−1.4	0.601
000 010 111 0	−543.211407	32.43	γ	β	1.356	58.5	54.9	0.4	0.601
000 011 001 1	−543.205524	47.88	γ	β	1.357	58.5	59.5	−3.7	0.438
000 011 001 1	−543.205524	47.88	γ	β'	3.118	27.6	35.3	27.4	0.438
000 011 001 1	−543.205524	47.88	γ	α	2.551	28.0	42.3	10.3	0.438
000 011 001 1	−543.205524	47.88	β	β'	2.498	56.7	52.6	0.2	0.438
000 011 001 1	−543.205524	47.88	β	α	1.544	63.6	68.4	11.2	0.438
000 011 001 1	−543.205524	47.88	β'	α	1.537	60.8	69.1	−8.4	0.438
000 011 010 1	−543.209146	38.37	γ	β	1.357	58.9	59.3	0.0	0.612
000 011 010 1	−543.209146	38.37	γ	γ'	3.156	33.5	33.0	−5.0	0.612
000 011 010 1	−543.209146	38.37	γ	α	2.540	28.1	40.5	1.3	0.612
000 011 010 1	−543.209146	38.37	β	γ'	3.156	33.3	27.3	28.4	0.612
000 011 010 1	−543.209146	38.37	β	α	1.541	63.1	67.3	0.8	0.612
000 011 010 1	−543.209146	38.37	γ'	α	2.558	30.4	48.4	6.7	0.612
000 011 011 0	−543.215144	22.62	γ'	β'	1.351	55.7	55.1	−0.5	0.400
000 011 011 0	−543.215144	22.62	γ'	γ	3.197	34.2	34.1	0.0	0.400
000 011 011 0	−543.215144	22.62	γ'	β	3.154	24.8	37.2	45.4	0.400
000 011 011 0	−543.215144	22.62	β'	γ	3.153	37.3	24.7	−45.2	0.400
000 011 011 0	−543.215144	22.62	β'	β	2.541	57.1	57.0	0.1	0.400
000 011 011 0	−543.215144	22.62	γ	β	1.351	55.7	55.1	0.5	0.400
000 011 100 1	−543.207151	43.61	γ	β	1.357	58.9	59.0	−1.8	0.516
000 011 100 1	−543.207151	43.61	γ	δ'	2.675	46.9	45.1	−2.1	0.516
000 011 100 1	−543.207151	43.61	γ	α	2.540	28.1	40.3	1.1	0.516
000 011 100 1	−543.207151	43.61	β	δ'	3.262	13.6	24.2	28.6	0.516
000 011 100 1	−543.207151	43.61	β	α	1.541	63.3	67.1	2.8	0.516
000 011 100 1	−543.207151	43.61	δ'	α	3.117	11.7	20.3	16.9	0.516
000 011 101 0	−543.217729	15.84	γ	β	1.352	56.0	55.1	0.0	0.299

000 011 101 0	−543.217729	15.84	γ	δ'	2.673	48.3	45.3	3.0	0.299
000 011 101 0	−543.217729	15.84	γ	β'	3.117	25.4	35.2	39.3	0.299
000 011 101 0	−543.217729	15.84	β	δ'	3.226	14.1	24.3	−51.0	0.299
000 011 101 0	−543.217729	15.84	β	β'	2.520	56.3	54.1	−3.1	0.299
000 011 101 0	−543.217729	15.84	δ'	β'	2.531	41.5	30.7	−0.6	0.299
000 011 110 0	−543.213770	26.23	γ	β	1.351	55.9	54.9	0.2	0.590
000 011 110 0	−543.213770	26.23	γ	δ'	2.691	48.9	47.0	−1.0	0.590
000 011 110 0	−543.213770	26.23	γ	γ'	3.175	35.3	30.7	13.6	0.590
000 011 110 0	−543.213770	26.23	β	δ'	3.255	13.5	25.0	−59.4	0.590
000 011 110 0	−543.213770	26.23	β	γ'	3.176	35.6	27.4	−28.9	0.590
000 011 110 0	−543.213770	26.23	δ'	γ'	1.491	63.7	62.7	−1.7	0.590
000 100 011 1	−543.207204	43.47	δ'	γ	2.674	45.0	46.6	−1.9	0.520
000 100 011 1	−543.207204	43.47	δ'	β	3.256	24.2	15.4	31.5	0.520
000 100 011 1	−543.207204	43.47	δ'	α	3.114	11.8	20.0	17.3	0.520
000 100 011 1	−543.207204	43.47	γ	β	1.357	59.1	59.2	0.1	0.520
000 100 011 1	−543.207204	43.47	γ	α	2.541	28.3	40.3	0.3	0.520
000 100 011 1	−543.207204	43.47	β	α	1.541	63.2	67.1	0.3	0.520
000 100 101 1	−543.209013	38.72	δ'	δ	1.538	61.0	61.5	0.2	0.385
000 100 101 1	−543.209013	38.72	δ'	β	3.249	20.2	17.7	14.0	0.385
000 100 101 1	−543.209013	38.72	δ'	α	3.145	15.2	19.1	0.6	0.385
000 100 101 1	−543.209013	38.72	δ	β	2.570	45.1	34.9	4.7	0.385
000 100 101 1	−543.209013	38.72	δ	α	3.126	16.1	12.2	12.2	0.385
000 100 101 1	−543.209013	38.72	β	α	1.533	61.1	67.1	0.0	0.385
000 100 110 1	−543.206971	44.08	δ'	δ	1.547	61.0	63.3	2.7	0.861
000 100 110 1	−543.206971	44.08	δ'	γ	2.641	40.0	42.4	−3.4	0.861
000 100 110 1	−543.206971	44.08	δ'	α	3.118	16.0	16.4	17.5	0.861
000 100 110 1	−543.206971	44.08	δ	γ	1.497	65.6	63.0	−6.6	0.861
000 100 110 1	−543.206971	44.08	δ	α	3.133	12.1	17.6	−20.8	0.861
000 100 110 1	−543.206971	44.08	γ	α	2.566	34.5	45.9	3.1	0.861
000 100 111 0	−543.203636	52.84	δ'	δ	1.546	60.6	64.4	−3.6	0.538
000 100 111 0	−543.203636	52.84	δ'	γ	2.665	40.3	46.5	3.3	0.538
000 100 111 0	−543.203636	52.84	δ'	β	3.224	19.5	13.5	45.5	0.538
000 100 111 0	−543.203636	52.84	δ	γ	1.509	65.8	65.6	9.3	0.538
000 100 111 0	−543.203636	52.84	δ	β	2.545	40.4	26.1	5.9	0.538
000 100 111 0	−543.203636	52.84	γ	β	1.356	59.6	55.0	−4.1	0.538
000 101 001 1	−543.211278	32.77	δ	β	2.559	42.4	35.1	3.0	0.261
000 101 001 1	−543.211278	32.77	δ	β'	3.230	24.1	19.7	−23.3	0.261
000 101 001 1	−543.211278	32.77	δ	α	3.126	13.1	14.7	−21.4	0.261
000 101 001 1	−543.211278	32.77	β	β'	2.485	52.6	52.5	0.2	0.261
000 101 001 1	−543.211278	32.77	β	α	1.537	61.2	68.4	−7.6	0.261
000 101 001 1	−543.211278	32.77	β'	α	1.538	61.1	68.5	7.8	0.261
000 101 010 1	−543.214731	23.71	δ	β	2.557	42.4	34.4	0.1	0.474
000 101 010 1	−543.214731	23.71	δ	γ'	2.642	44.5	44.5	−0.8	0.474
000 101 010 1	−543.214731	23.71	δ	α	3.103	12.9	12.3	−1.3	0.474
000 101 010 1	−543.214731	23.71	β	γ'	3.126	32.6	27.4	−22.7	0.474
000 101 010 1	−543.214731	23.71	β	α	1.535	61.0	67.4	−0.4	0.474
000 101 010 1	−543.214731	23.71	γ'	α	2.560	30.7	47.4	−5.4	0.474
000 101 011 0	−543.217733	15.83	δ'	β'	2.531	41.5	30.7	0.9	0.296
000 101 011 0	−543.217733	15.83	δ'	γ	2.673	45.2	48.3	−3.1	0.296
000 101 011 0	−543.217733	15.83	δ'	β	3.225	24.2	14.4	51.2	0.296
000 101 011 0	−543.217733	15.83	β'	γ	3.119	35.0	25.2	−39.5	0.296
000 101 011 0	−543.217733	15.83	β'	β	2.520	54.0	56.5	2.6	0.296
000 101 011 0	−543.217733	15.83	γ	β	1.352	56.0	55.1	0.4	0.296
000 101 100 1	−543.209013	38.72	δ	β	2.570	45.1	34.8	−4.4	0.384
000 101 100 1	−543.209013	38.72	δ	δ'	1.538	61.5	61.0	−0.1	0.384
000 101 100 1	−543.209013	38.72	δ	α	3.126	16.1	12.2	−12.6	0.384
000 101 100 1	−543.209013	38.72	β	δ'	3.249	17.5	20.3	−13.7	0.384
000 101 100 1	−543.209013	38.72	β	α	1.534	61.1	67.1	−0.3	0.384
000 101 100 1	−543.209013	38.72	δ'	α	3.145	15.2	19.2	−0.6	0.384
000 101 101 0	−543.216658	18.65	δ'	β'	2.551	44.4	31.5	−5.3	0.173

000 101 101 0	−543.216658	18.65	δ'	δ	1.538	61.3	61.2	0.0	0.173
000 101 101 0	−543.216658	18.65	δ'	β	3.213	20.2	16.3	26.0	0.173
000 101 101 0	−543.216658	18.65	β'	δ	3.214	16.2	20.2	−25.5	0.173
000 101 101 0	−543.216658	18.65	β'	β	2.481	53.7	53.9	−0.1	0.173
000 101 101 0	−543.216658	18.65	δ	β	2.551	44.4	31.5	5.6	0.173
000 101 110 0	−543.211289	32.74	δ	β	2.551	45.2	31.3	6.0	0.490
000 101 110 0	−543.211289	32.74	δ	δ'	1.546	61.2	63.1	2.6	0.490
000 101 110 0	−543.211289	32.74	δ	γ'	2.638	40.1	42.1	−3.3	0.490
000 101 110 0	−543.211289	32.74	β	δ'	3.242	15.8	19.6	36.8	0.490
000 101 110 0	−543.211289	32.74	β	γ'	3.107	34.8	31.2	22.6	0.490
000 101 110 0	−543.211289	32.74	δ'	γ'	1.497	65.7	62.8	−6.3	0.490
000 110 001 1	−543.211756	31.52	δ'	γ'	1.490	63.4	63.1	−0.4	0.525
000 110 001 1	−543.211756	31.52	δ'	β	3.271	24.2	16.4	−29.3	0.525
000 110 001 1	−543.211756	31.52	δ'	α	3.132	8.7	20.3	−13.5	0.525
000 110 001 1	−543.211756	31.52	γ'	β	3.156	28.9	31.9	−16.4	0.525
000 110 001 1	−543.211756	31.52	γ'	α	2.578	34.0	48.2	−5.2	0.525
000 110 001 1	−543.211756	31.52	β	α	1.534	60.7	67.4	−1.7	0.525
000 110 010 1	−543.214090	25.39	δ	γ	1.492	63.7	63.0	0.4	0.951
000 110 010 1	−543.214090	25.39	δ	γ'	2.668	46.3	45.3	3.3	0.951
000 110 010 1	−543.214090	25.39	δ	α	3.109	8.6	17.7	−0.5	0.951
000 110 010 1	−543.214090	25.39	γ	γ'	3.144	31.5	33.5	−6.7	0.951
000 110 010 1	−543.214090	25.39	γ	α	2.566	33.4	46.2	−1.0	0.951
000 110 010 1	−543.214090	25.39	γ'	α	2.542	29.9	45.3	1.4	0.951
000 110 011 0	−543.213744	26.30	δ'	γ'	1.491	63.7	62.7	−1.1	0.593
000 110 011 0	−543.213744	26.30	δ'	γ	2.690	46.9	48.8	−1.0	0.593
000 110 011 0	−543.213744	26.30	δ'	β	3.253	24.9	13.8	−60.0	0.593
000 110 011 0	−543.213744	26.30	γ'	γ	3.173	31.1	35.3	13.7	0.593
000 110 011 0	−543.213744	26.30	γ'	β	3.173	27.8	36.0	−29.3	0.593
000 110 011 0	−543.213744	26.30	γ	β	1.351	55.9	54.8	−0.1	0.593
000 110 100 1	−543.206983	44.05	δ	γ	1.497	65.6	62.9	−7.0	0.859
000 110 100 1	−543.206983	44.05	δ	δ'	1.547	63.3	61.0	2.7	0.859
000 110 100 1	−543.206983	44.05	δ	α	3.133	12.1	17.6	−20.9	0.859
000 110 100 1	−543.206983	44.05	γ	δ'	2.640	42.6	40.0	−3.8	0.859
000 110 100 1	−543.206983	44.05	γ	α	2.566	34.5	45.9	3.7	0.859
000 110 100 1	−543.206983	44.05	δ'	α	3.118	16.0	16.4	17.7	0.859
000 110 101 0	−543.211245	32.86	δ'	γ'	1.497	65.6	62.8	6.2	0.488
000 110 101 0	−543.211245	32.86	δ'	δ	1.546	63.1	61.2	−2.8	0.488
000 110 101 0	−543.211245	32.86	δ'	β	3.243	19.6	15.7	−36.7	0.488
000 110 101 0	−543.211245	32.86	γ'	δ	2.638	42.0	40.1	3.1	0.488
000 110 101 0	−543.211245	32.86	γ'	β	3.109	31.1	34.6	−22.6	0.488
000 110 101 0	−543.211245	32.86	δ	β	2.551	45.3	31.3	−5.6	0.488
000 110 110 0	−543.205433	48.12	δ'	γ'	1.498	66.3	62.6	−8.4	0.907
000 110 110 0	−543.205433	48.12	δ'	δ	1.557	63.0	63.0	−0.2	0.907
000 110 110 0	−543.205433	48.12	δ'	γ	2.655	41.1	43.2	10.3	0.907
000 110 110 0	−543.205433	48.12	γ'	δ	2.655	43.0	41.0	−10.3	0.907
000 110 110 0	−543.205433	48.12	γ'	γ	3.086	36.4	36.6	0.0	0.907
000 110 110 0	−543.205433	48.12	δ	γ	1.498	66.3	62.7	8.8	0.907
000 111 001 0	−543.212485	29.60	δ	γ	1.503	63.7	65.9	0.3	0.386
000 111 001 0	−543.212485	29.60	δ	β	2.531	37.5	25.6	−1.3	0.386
000 111 001 0	−543.212485	29.60	δ	β'	3.224	24.8	15.4	47.1	0.386
000 111 001 0	−543.212485	29.60	γ	β	1.356	58.6	54.9	−0.7	0.386
000 111 001 0	−543.212485	29.60	γ	β'	3.148	26.6	35.3	31.1	0.386
000 111 001 0	−543.212485	29.60	β	β'	2.514	58.4	53.8	−3.2	0.386
000 111 010 0	−543.211438	32.35	δ	γ	1.504	63.9	65.8	1.0	0.605
000 111 010 0	−543.211438	32.35	δ	β	2.530	37.6	25.5	0.9	0.605
000 111 010 0	−543.211438	32.35	δ	γ'	2.666	47.4	44.6	3.7	0.605
000 111 010 0	−543.211438	32.35	γ	β	1.356	58.6	54.9	−0.5	0.605
000 111 010 0	−543.211438	32.35	γ	γ'	3.181	32.3	32.9	0.8	0.605
000 111 010 0	−543.211438	32.35	β	γ'	3.136	37.0	26.2	−38.9	0.605
000 111 100 0	−543.203646	52.81	δ	γ	1.509	65.8	65.6	−9.2	0.540

000 111 100 0	−543.203646	52.81	δ	β	2.545	40.3	26.1	−5.6	0.540
000 111 100 0	−543.203646	52.81	δ	δ'	1.546	64.4	60.4	3.5	0.540
000 111 100 0	−543.203646	52.81	γ	β	1.356	59.6	55.0	4.2	0.540
000 111 100 0	−543.203646	52.81	γ	δ'	2.665	46.5	40.2	−3.3	0.540
000 111 100 0	−543.203646	52.81	β	δ'	3.225	13.4	19.3	−45.1	0.540
001 001 001 1	−543.207757	42.02	β''	β'	2.521	49.2	49.4	0.4	0.205
001 001 001 1	−543.207757	42.02	β''	β	2.516	49.7	49.7	−0.1	0.205
001 001 001 1	−543.207757	42.02	β''	α	1.544	60.9	70.4	0.4	0.205
001 001 001 1	−543.207757	42.02	β'	β	2.521	49.3	49.2	−0.3	0.205
001 001 001 1	−543.207757	42.02	β'	α	1.544	60.9	70.5	0.1	0.205
001 001 001 1	−543.207757	42.02	β	α	1.544	60.9	70.4	−0.5	0.205
001 001 010 1	−543.213382	27.25	β	β'	2.486	53.1	53.1	0.1	0.174
001 001 010 1	−543.213382	27.25	β	γ''	3.162	29.2	26.6	23.4	0.174
001 001 010 1	−543.213382	27.25	β	α	1.538	61.0	68.8	8.5	0.174
001 001 010 1	−543.213382	27.25	β'	γ''	3.161	29.3	26.7	−23.5	0.174
001 001 010 1	−543.213382	27.25	β'	α	1.538	61.0	68.6	−8.3	0.174
001 001 010 1	−543.213382	27.25	γ''	α	2.577	31.3	50.3	−0.2	0.174
001 001 011 0	−543.220959	7.36	β''	β'	2.510	54.1	54.1	−0.2	0.238
001 001 011 0	−543.220959	7.36	β''	γ	3.137	34.9	25.0	39.9	0.238
001 001 011 0	−543.220959	7.36	β''	β	2.531	53.8	56.4	−2.6	0.238
001 001 011 0	−543.220959	7.36	β'	γ	3.136	34.7	25.0	−40.0	0.238
001 001 011 0	−543.220959	7.36	β'	β	2.530	53.7	56.5	2.4	0.238
001 001 011 0	−543.220959	7.36	γ	β	1.352	55.9	55.2	0.0	0.238
001 001 100 1	−543.211517	32.15	β	β'	2.489	53.0	52.7	−0.2	0.137
001 001 100 1	−543.211517	32.15	β	δ''	3.267	14.6	22.9	−14.4	0.137
001 001 100 1	−543.211517	32.15	β	α	1.538	61.1	68.5	−8.7	0.137
001 001 100 1	−543.211517	32.15	β'	δ''	3.266	14.9	22.9	15.0	0.137
001 001 100 1	−543.211517	32.15	β'	α	1.538	61.0	68.5	8.3	0.137
001 001 100 1	−543.211517	32.15	δ''	α	3.150	11.9	22.1	−0.1	0.137
001 001 101 0	−543.223761	0.00	β''	β'	2.514	53.7	53.5	−0.4	0.265
001 001 101 0	−543.223761	0.00	β''	δ	3.220	14.7	23.3	−34.4	0.265
001 001 101 0	−543.223761	0.00	β''	β	2.510	53.0	53.5	−0.5	0.265
001 001 101 0	−543.223761	0.00	β'	δ	3.219	15.1	23.4	35.0	0.265
001 001 101 0	−543.223761	0.00	β'	β	2.508	53.2	53.5	0.2	0.265
001 001 101 0	−543.223761	0.00	δ	β	2.536	41.5	31.0	−0.1	0.265
001 001 110 0	−543.219802	10.39	β	β'	2.509	54.4	54.2	0.2	0.206
001 001 110 0	−543.219802	10.39	β	δ''	3.250	14.1	24.0	42.6	0.206
001 001 110 0	−543.219802	10.39	β	γ''	3.157	33.3	28.1	23.8	0.206
001 001 110 0	−543.219802	10.39	β'	δ''	3.249	14.3	24.0	−42.9	0.206
001 001 110 0	−543.219802	10.39	β'	γ''	3.154	33.5	28.4	−23.8	0.206
001 001 110 0	−543.219802	10.39	δ''	γ''	1.492	63.6	62.8	0.1	0.206
001 010 010 1	−543.216707	18.52	β	γ'	3.140	32.8	27.7	−23.6	0.576
001 010 010 1	−543.216707	18.52	β	γ''	3.140	32.9	27.7	23.4	0.576
001 010 010 1	−543.216707	18.52	β	α	1.535	60.8	67.5	0.0	0.576
001 010 010 1	−543.216707	18.52	γ'	γ''	3.157	32.2	32.2	0.2	0.576
001 010 010 1	−543.216707	18.52	γ'	α	2.559	30.5	47.7	−6.7	0.576
001 010 010 1	−543.216707	18.52	γ''	α	2.559	30.5	47.7	6.6	0.576
001 010 011 0	−543.220027	9.80	β'	γ''	3.142	34.4	27.1	31.7	0.312
001 010 011 0	−543.220027	9.80	β'	γ	3.130	35.7	25.7	−39.7	0.312
001 010 011 0	−543.220027	9.80	β'	β	2.519	54.6	57.5	2.1	0.312
001 010 011 0	−543.220027	9.80	γ''	γ	3.185	32.2	34.3	7.3	0.312
001 010 011 0	−543.220027	9.80	γ''	β	3.168	25.9	35.3	−37.3	0.312
001 010 011 0	−543.220027	9.80	γ	β	1.352	55.8	55.1	0.7	0.312
001 010 100 1	−543.214856	23.38	β	γ'	3.144	32.5	27.2	−23.0	0.445
001 010 100 1	−543.214856	23.38	β	δ''	3.255	16.8	23.5	22.7	0.445
001 010 100 1	−543.214856	23.38	β	α	1.536	60.8	67.4	0.1	0.445
001 010 100 1	−543.214856	23.38	γ'	δ''	2.658	44.6	44.4	0.2	0.445
001 010 100 1	−543.214856	23.38	γ'	α	2.559	30.7	47.5	−6.0	0.445
001 010 100 1	−543.214856	23.38	δ''	α	3.123	12.0	19.4	13.7	0.445
001 010 101 0	−543.222765	2.61	β'	γ''	3.147	33.7	26.1	−30.3	0.144

001 010 101 0	−543.222765	2.61	β'	δ	3.217	15.6	23.7	35.6	0.144
001 010 101 0	−543.222765	2.61	β'	β	2.500	53.8	54.1	0.1	0.144
001 010 101 0	−543.222765	2.61	γ''	δ	2.655	44.7	44.7	0.0	0.144
001 010 101 0	−543.222765	2.61	γ''	β	3.125	26.9	34.1	30.5	0.144
001 010 101 0	−543.222765	2.61	δ	β	2.536	41.6	30.9	−0.5	0.144
001 010 110 0	−543.218583	13.59	β	γ''	3.142	34.7	26.7	−30.8	0.548
001 010 110 0	−543.218583	13.59	β	δ'	3.247	14.8	24.2	43.9	0.548
001 010 110 0	−543.218583	13.59	β	γ'	3.153	34.1	28.4	23.6	0.548
001 010 110 0	−543.218583	13.59	γ''	δ'	2.675	45.1	46.5	−3.5	0.548
001 010 110 0	−543.218583	13.59	γ''	γ'	3.158	33.4	31.8	7.1	0.548
001 010 110 0	−543.218583	13.59	δ'	γ'	1.492	63.7	62.8	0.2	0.548
001 011 010 0	−543.219986	9.91	β'	γ	3.131	35.4	25.6	39.8	0.310
001 011 010 0	−543.219986	9.91	β'	β	2.520	54.3	57.2	−2.3	0.310
001 011 010 0	−543.219986	9.91	β'	γ''	3.139	34.7	27.3	−31.4	0.310
001 011 010 0	−543.219986	9.91	γ	β	1.352	55.8	55.1	−0.3	0.310
001 011 010 0	−543.219986	9.91	γ	γ''	3.186	34.3	32.1	−7.1	0.310
001 011 010 0	−543.219986	9.91	β	γ''	3.166	35.6	25.9	36.7	0.310
001 011 100 0	−543.217838	15.55	β'	γ	3.137	35.1	25.0	−39.6	0.254
001 011 100 0	−543.217838	15.55	β'	β	2.528	54.0	56.9	2.4	0.254
001 011 100 0	−543.217838	15.55	β'	δ''	3.229	14.8	23.0	37.8	0.254
001 011 100 0	−543.217838	15.55	γ	β	1.352	56.0	55.1	0.4	0.254
001 011 100 0	−543.217838	15.55	γ	δ''	2.681	48.5	45.2	2.4	0.254
001 011 100 0	−543.217838	15.55	β	δ''	3.241	13.3	24.0	−51.9	0.254
001 100 010 1	−543.214790	23.55	β	δ''	3.254	17.1	23.7	−23.3	0.444
001 100 010 1	−543.214790	23.55	β	γ'	3.147	32.2	27.0	23.0	0.444
001 100 010 1	−543.214790	23.55	β	α	1.536	60.8	67.4	0.3	0.444
001 100 010 1	−543.214790	23.55	δ''	γ'	2.657	44.3	44.7	0.2	0.444
001 100 010 1	−543.214790	23.55	δ''	α	3.123	11.9	19.4	−14.3	0.444
001 100 010 1	−543.214790	23.55	γ'	α	2.560	30.7	47.5	5.7	0.444
001 100 011 0	−543.217797	15.66	β'	δ''	3.229	14.7	23.0	38.1	0.254
001 100 011 0	−543.217797	15.66	β'	γ	3.135	35.1	25.1	−39.6	0.254
001 100 011 0	−543.217797	15.66	β'	β	2.527	54.1	56.9	2.4	0.254
001 100 011 0	−543.217797	15.66	δ''	γ	2.682	45.3	48.4	2.5	0.254
001 100 011 0	−543.217797	15.66	δ''	β	3.241	24.1	13.3	−52.2	0.254
001 100 011 0	−543.217797	15.66	γ	β	1.352	56.0	55.0	0.3	0.254
001 100 100 1	−543.208991	38.78	β	δ'	3.268	16.9	26.1	13.7	0.359
001 100 100 1	−543.208991	38.78	β	δ''	3.268	17.1	26.2	−14.0	0.359
001 100 100 1	−543.208991	38.78	β	α	1.535	61.0	67.2	0.1	0.359
001 100 100 1	−543.208991	38.78	δ'	δ''	1.541	61.3	61.3	0.1	0.359
001 100 100 1	−543.208991	38.78	δ'	α	3.145	15.3	19.2	25.5	0.359
001 100 100 1	−543.208991	38.78	δ''	α	3.145	15.3	19.2	−25.7	0.359
001 100 101 0	−543.216676	18.60	β'	δ''	3.247	14.5	25.9	25.3	0.115
001 100 101 0	−543.216676	18.60	β'	δ	3.231	15.9	26.3	−26.4	0.115
001 100 101 0	−543.216676	18.60	β'	β	2.507	53.4	53.4	0.2	0.115
001 100 101 0	−543.216676	18.60	δ''	δ	1.541	61.2	61.6	−0.2	0.115
001 100 101 0	−543.216676	18.60	δ''	β	3.228	19.8	15.4	−25.9	0.115
001 100 101 0	−543.216676	18.60	δ	β	2.551	44.4	31.5	−4.3	0.115
001 100 110 0	−543.211294	32.73	β	δ''	3.247	15.4	26.8	−26.1	0.470
001 100 110 0	−543.211294	32.73	β	δ'	3.263	14.9	27.4	33.7	0.470
001 100 110 0	−543.211294	32.73	β	γ'	3.172	33.3	26.2	19.0	0.470
001 100 110 0	−543.211294	32.73	δ''	δ'	1.551	61.2	63.4	−2.7	0.470
001 100 110 0	−543.211294	32.73	δ''	γ'	2.646	40.2	42.6	4.4	0.470
001 100 110 0	−543.211294	32.73	δ'	γ'	1.497	65.6	62.8	7.2	0.470
001 101 010 0	−543.222754	2.64	β'	δ	3.218	15.6	23.7	35.6	0.134
001 101 010 0	−543.222754	2.64	β'	β	2.499	53.8	54.3	0.3	0.134
001 101 010 0	−543.222754	2.64	β'	γ''	3.145	33.8	26.6	−30.6	0.134
001 101 010 0	−543.222754	2.64	δ	β	2.536	41.7	30.9	−0.9	0.134
001 101 010 0	−543.222754	2.64	δ	γ''	2.656	44.7	44.4	0.5	0.134
001 101 010 0	−543.222754	2.64	β	γ''	3.129	33.7	26.4	30.2	0.134
001 101 100 0	−543.216628	18.73	β'	δ	3.231	15.9	26.3	−26.5	0.116

001 101 100 0	−543.216628	18.73	β'	β	2.508	53.5	53.4	0.2	0.116
001 101 100 0	−543.216628	18.73	β'	δ"	3.248	14.5	25.8	25.6	0.116
001 101 100 0	−543.216628	18.73	δ	β	2.551	44.5	31.5	−4.4	0.116
001 101 100 0	−543.216628	18.73	δ	δ"	1.541	61.6	61.3	−0.1	0.116
001 101 100 0	−543.216628	18.73	β	δ"	3.229	15.4	19.9	−26.0	0.116
001 110 010 0	−543.21860	13.55	β	δ'	3.246	15.0	24.2	43.8	0.551
001 110 010 0	−543.21860	13.55	β	γ'	3.150	34.3	28.7	23.5	0.551
001 110 010 0	−543.21860	13.55	β	γ"	3.144	34.4	26.4	−30.7	0.551
001 110 010 0	−543.21860	13.55	δ'	γ'	1.492	63.8	62.8	−0.1	0.551
001 110 010 0	−543.21860	13.55	δ'	γ"	2.674	46.5	45.3	−3.8	0.551
001 110 010 0	−543.21860	13.55	γ'	γ"	3.157	31.6	33.6	6.8	0.551
001 110 100 0	−543.211265	32.81	β	δ'	3.263	14.8	27.5	33.4	0.468
001 110 100 0	−543.211265	32.81	β	γ'	3.175	33.1	25.7	18.7	0.468
001 110 100 0	−543.211265	32.81	β	δ"	3.248	15.6	26.9	−26.2	0.468
001 110 100 0	−543.211265	32.81	δ'	γ'	1.497	65.6	62.8	7.8	0.468
001 110 100 0	−543.211265	32.81	δ'	δ"	1.551	63.3	61.1	−2.8	0.468
001 110 100 0	−543.211265	32.81	γ'	δ"	2.644	42.9	40.2	4.9	0.468
010 010 010 1	−543.219091	12.26	γ"	γ'	3.152	33.1	33.1	−0.1	1.008
010 010 010 1	−543.219091	12.26	γ"	γ	3.152	33.0	33.1	−0.1	1.008
010 010 010 1	−543.219091	12.26	γ"	α	2.547	29.8	45.7	0.1	1.008
010 010 010 1	−543.219091	12.26	γ'	γ	3.152	33.1	33.0	0.0	1.008
010 010 010 1	−543.219091	12.26	γ'	α	2.546	29.8	45.6	0.1	1.008
010 010 010 1	−543.219091	12.26	γ	α	2.546	29.8	45.6	0.0	1.008
010 010 011 0	−543.218926	12.69	γ"	γ'	3.167	33.6	33.4	−0.1	0.633
010 010 011 0	−543.218926	12.69	γ"	γ	3.182	32.5	34.7	7.0	0.633
010 010 011 0	−543.218926	12.69	γ"	β	3.158	26.4	36.7	−36.4	0.633
010 010 011 0	−543.218926	12.69	γ'	γ	3.180	32.8	35.0	−6.9	0.633
010 010 011 0	−543.218926	12.69	γ'	β	3.159	26.5	36.4	36.9	0.633
010 010 011 0	−543.218926	12.69	γ	β	1.351	55.8	55.0	−0.3	0.633
010 010 100 1	−543.217194	17.24	γ	γ'	3.158	32.4	32.5	0.3	0.885
010 010 100 1	−543.217194	17.24	γ	δ"	2.658	45.1	44.7	0.0	0.885
010 010 100 1	−543.217194	17.24	γ	α	2.547	30.0	45.4	−0.6	0.885
010 010 100 1	−543.217194	17.24	γ'	δ"	2.659	44.9	44.6	0.1	0.885
010 010 100 1	−543.217194	17.24	γ'	α	2.547	30.1	45.5	0.2	0.885
010 010 100 1	−543.217194	17.24	δ"	α	3.099	11.9	16.7	0.4	0.885
010 010 101 0	−543.221662	5.51	γ"	γ'	3.170	32.8	32.6	−0.2	0.493
010 010 101 0	−543.221662	5.51	γ"	δ	2.657	44.6	44.8	−0.6	0.493
010 010 101 0	−543.221662	5.51	γ"	β	3.123	26.7	34.5	−30.3	0.493
010 010 101 0	−543.221662	5.51	γ'	δ	2.656	44.7	45.0	0.5	0.493
010 010 101 0	−543.221662	5.51	γ'	β	3.122	26.9	34.8	30.3	0.493
010 010 101 0	−543.221662	5.51	δ	β	2.535	41.7	30.8	0.2	0.493
010 010 110 0	−543.217491	16.46	γ"	γ'	3.178	32.2	32.6	−0.2	0.915
010 010 110 0	−543.217491	16.46	γ"	δ	2.674	45.5	46.5	−4.2	0.915
010 010 110 0	−543.217491	16.46	γ"	γ	3.153	34.3	32.3	6.8	0.915
010 010 110 0	−543.217491	16.46	γ'	δ	2.674	45.4	46.7	3.6	0.915
010 010 110 0	−543.217491	16.46	γ'	γ	3.158	33.8	31.7	−6.9	0.915
010 010 110 0	−543.217491	16.46	δ	γ	1.493	63.9	62.8	0.4	0.915
010 011 100 0	−543.216860	18.12	γ'	γ	3.189	32.0	34.1	7.0	0.522
010 011 100 0	−543.216860	18.12	γ'	β	3.165	25.8	36.0	−36.8	0.522
010 011 100 0	−543.216860	18.12	γ'	δ"	2.665	45.1	44.8	−1.3	0.522
010 011 100 0	−543.216860	18.12	γ	β	1.352	55.9	55.0	0.0	0.522
010 011 100 0	−543.216860	18.12	γ	δ"	2.680	48.7	45.4	−2.0	0.522
010 011 100 0	−543.216860	18.12	β	δ"	3.236	14.3	24.2	52.7	0.522
010 100 011 0	−543.216835	18.18	γ'	δ"	2.665	45.0	44.8	−1.5	0.523
010 100 011 0	−543.216835	18.18	γ'	γ	3.188	31.9	34.1	7.2	0.523
010 100 011 0	−543.216835	18.18	γ'	β	3.163	25.9	36.4	−36.0	0.523
010 100 011 0	−543.216835	18.18	δ"	γ	2.681	45.4	48.7	−2.1	0.523
010 100 011 0	−543.216835	18.18	δ"	β	3.240	24.2	13.8	52.5	0.523
010 100 011 0	−543.216835	18.18	γ	β	1.352	55.9	55.0	−0.4	0.523
010 100 100 1	−543.211495	32.20	γ	δ'	2.662	45.4	45.4	5.8	0.811

010 100 100 1	−543.211495	32.20	γ	δ"	2.663	45.0	45.6	−6.9	0.811
010 100 100 1	−543.211495	32.20	γ	α	2.546	30.2	45.2	0.9	0.811
010 100 100 1	−543.211495	32.20	δ'	δ"	1.544	61.4	61.4	−0.2	0.811
010 100 100 1	−543.211495	32.20	δ'	α	3.122	15.2	16.5	−11.6	0.811
010 100 100 1	−543.211495	32.20	δ"	α	3.121	15.2	16.6	12.2	0.811
010 100 101 0	−543.215660	21.27	γ'	δ"	2.669	45.3	45.7	5.9	0.427
010 100 101 0	−543.215660	21.27	γ'	δ	2.662	44.9	45.8	−6.8	0.427
010 100 101 0	−543.215660	21.27	γ'	β	3.130	26.1	33.6	−30.0	0.427
010 100 101 0	−543.215660	21.27	δ"	δ	1.543	61.1	61.6	0.0	0.427
010 100 101 0	−543.215660	21.27	δ"	β	3.226	20.0	16.0	26.7	0.427
010 100 101 0	−543.215660	21.27	δ	β	2.550	44.4	31.4	5.4	0.427
010 100 110 0	−543.210233	35.52	γ'	δ"	2.671	45.6	46.3	−7.3	0.843
010 100 110 0	−543.210233	35.52	γ'	δ	2.682	45.6	48.1	3.2	0.843
010 100 110 0	−543.210233	35.52	γ'	γ	3.182	32.4	28.1	7.3	0.843
010 100 110 0	−543.210233	35.52	δ"	δ	1.552	61.1	63.4	2.5	0.843
010 100 110 0	−543.210233	35.52	δ"	γ	2.646	40.2	42.8	−4.7	0.843
010 100 110 0	−543.210233	35.52	δ	γ	1.497	65.7	62.8	−7.3	0.843
010 101 100 0	−543.215670	21.24	γ'	δ	2.662	44.9	45.8	−6.9	0.427
010 101 100 0	−543.215670	21.24	γ'	β	3.132	26.0	33.5	−30.3	0.427
010 101 100 0	−543.215670	21.24	γ'	δ"	2.669	45.3	45.6	5.8	0.427
010 101 100 0	−543.215670	21.24	δ	β	2.550	44.5	31.3	5.6	0.427
010 101 100 0	−543.215670	21.24	δ	δ"	1.543	61.6	61.2	0.0	0.427
010 101 100 0	−543.215670	21.24	β	δ"	3.225	16.2	20.1	27.0	0.427
010 110 100 0	−543.210282	35.39	γ'	δ	2.682	45.8	48.2	−3.1	0.841
010 110 100 0	−543.210282	35.39	γ'	γ	3.180	32.7	28.2	−7.4	0.841
010 110 100 0	−543.210282	35.39	γ'	δ"	2.672	45.5	46.2	7.7	0.841
010 110 100 0	−543.210282	35.39	δ	γ	1.497	65.8	62.8	7.4	0.841
010 110 100 0	−543.210282	35.39	δ	δ"	1.552	63.3	61.1	−2.6	0.841
010 110 100 0	−543.210282	35.39	γ	δ"	2.645	42.7	40.2	4.6	0.841
011 100 100 0	−543.210698	34.30	γ	β	1.351	56.1	54.9	0.3	0.474
011 100 100 0	−543.210698	34.30	γ	δ'	2.686	48.9	46.3	8.3	0.474
011 100 100 0	−543.210698	34.30	γ	δ"	2.686	48.8	46.3	−8.8	0.474
011 100 100 0	−543.210698	34.30	β	δ'	3.252	13.8	26.9	−43.0	0.474
011 100 100 0	−543.210698	34.30	β	δ"	3.251	14.1	26.9	43.2	0.474
011 100 100 0	−543.210698	34.30	δ'	δ"	1.548	61.4	61.4	0.0	0.474
100 100 100 1	−543.202482	55.87	δ"	δ'	1.541	60.8	61.0	0.2	0.713
100 100 100 1	−543.202482	55.87	δ"	δ	1.541	61.0	61.0	0.0	0.713
100 100 100 1	−543.202482	55.87	δ"	α	3.147	17.8	16.5	−0.5	0.713
100 100 100 1	−543.202482	55.87	δ'	δ	1.541	60.9	60.9	0.0	0.713
100 100 100 1	−543.202482	55.87	δ'	α	3.148	17.7	16.4	0.0	0.713
100 100 100 1	−543.202482	55.87	δ	α	3.147	17.8	16.4	0.2	0.713
100 100 101 0	−543.206421	45.52	δ"	δ'	1.543	61.0	61.0	0.0	0.347
100 100 101 0	−543.206421	45.52	δ"	δ	1.540	60.8	61.2	0.1	0.347
100 100 101 0	−543.206421	45.52	δ"	β	3.239	22.9	15.8	16.6	0.347
100 100 101 0	−543.206421	45.52	δ'	δ	1.540	60.7	61.1	0.0	0.347
100 100 101 0	−543.206421	45.52	δ'	β	3.239	22.9	15.6	−16.0	0.347
100 100 101 0	−543.206421	45.52	δ	β	2.565	46.9	31.9	0.2	0.347
100 100 110 0	−543.199442	63.85	δ"	δ'	1.540	60.7	60.7	−0.1	0.763
100 100 110 0	−543.199442	63.85	δ"	δ	1.547	61.0	63.5	−2.4	0.763
100 100 110 0	−543.199442	63.85	δ"	γ	2.657	41.2	40.6	−4.9	0.763
100 100 110 0	−543.199442	63.85	δ'	δ	1.548	60.9	63.4	2.4	0.763
100 100 110 0	−543.199442	63.85	δ'	γ	2.657	41.1	40.6	4.7	0.763
100 100 110 0	−543.199442	63.85	δ	γ	1.504	67.5	62.6	−0.1	0.763

5. Supplemental Exit Vector Plots

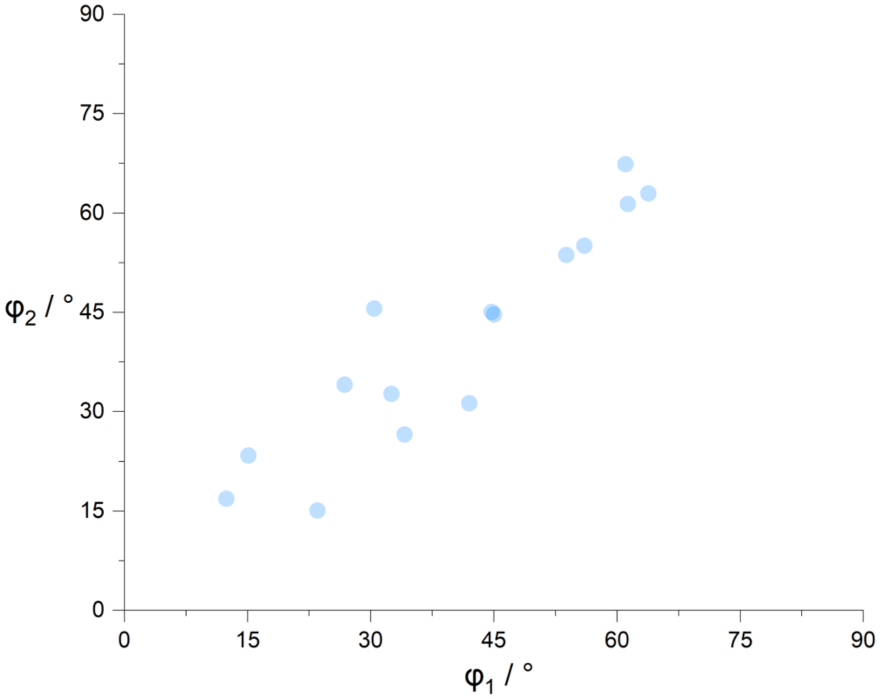

Figure S4. φ_1–φ_2 plot for **Me$_2$BV**.

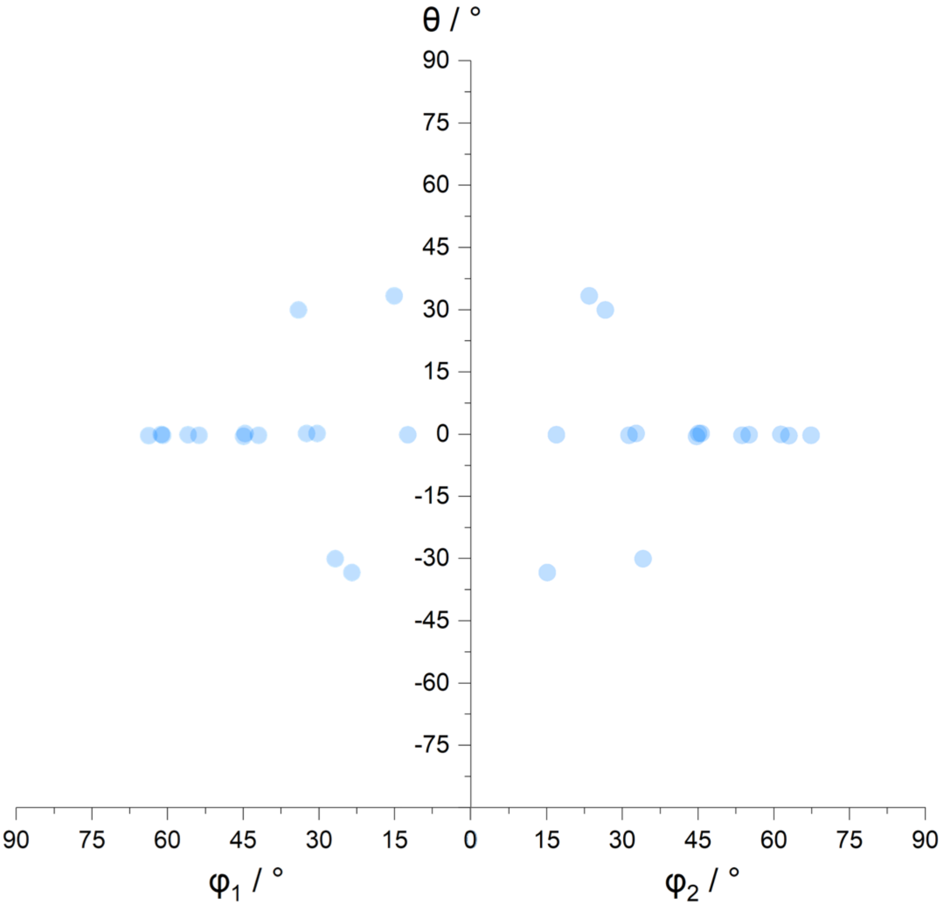

Figure S5. θ–φ_1 / θ–φ_2 plot for **Me$_2$BV**.

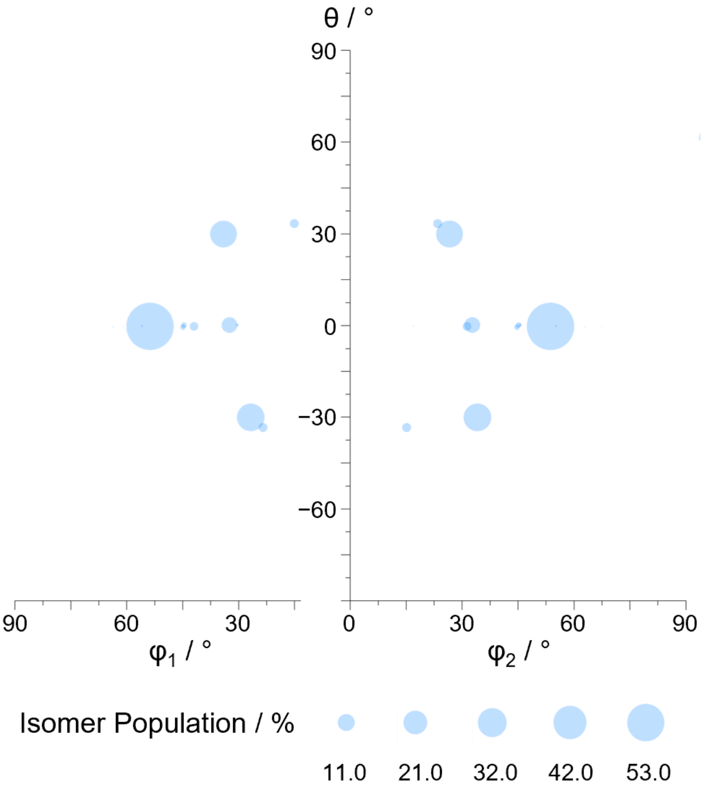

Figure S6. θ–φ_1 / θ–φ_2 plot adjusted by Boltzmann distribution at 298 K for **Me_3BV**.

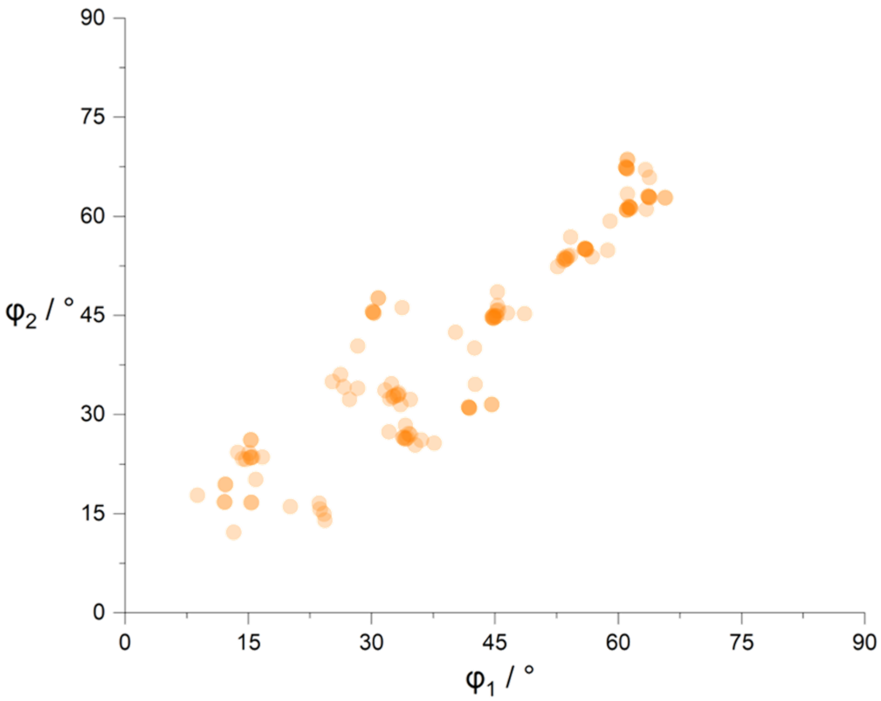

Figure S7. φ_1–φ_2 plot for **Me_3BV**.

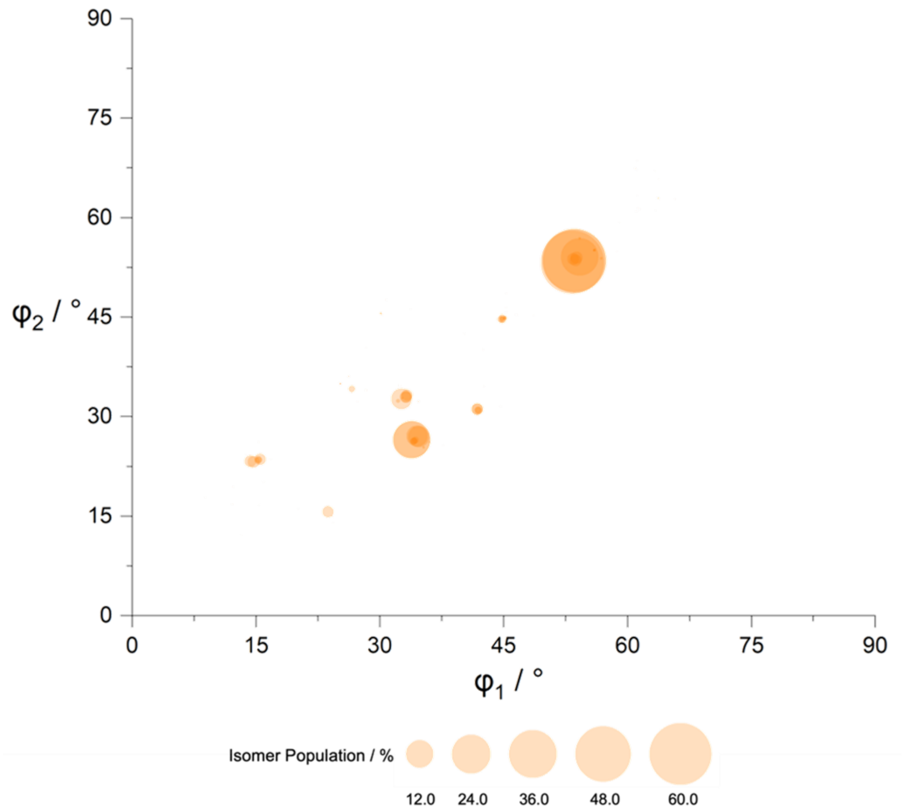

Figure S8. φ_1–φ_2 plot adjusted by Boltzmann distribution at 298 K for **Me$_3$BV**.

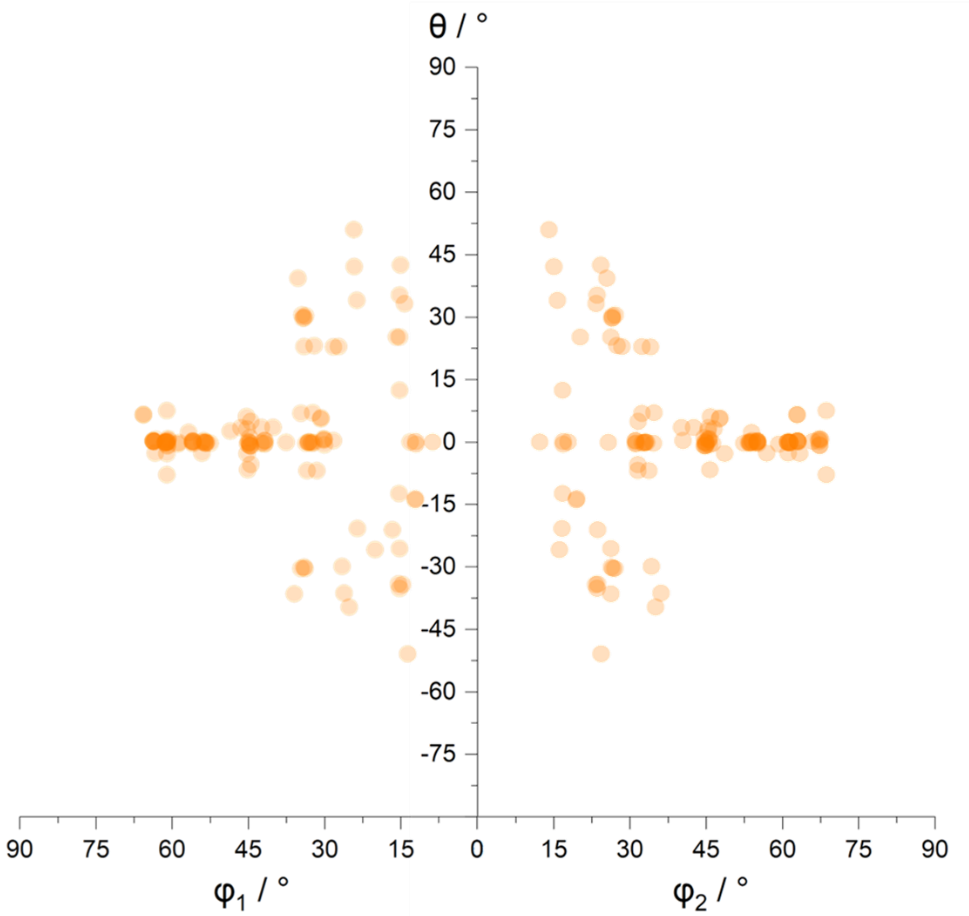

Figure S9. θ–φ_1 / θ–φ_2 plot for **Me$_3$BV**.

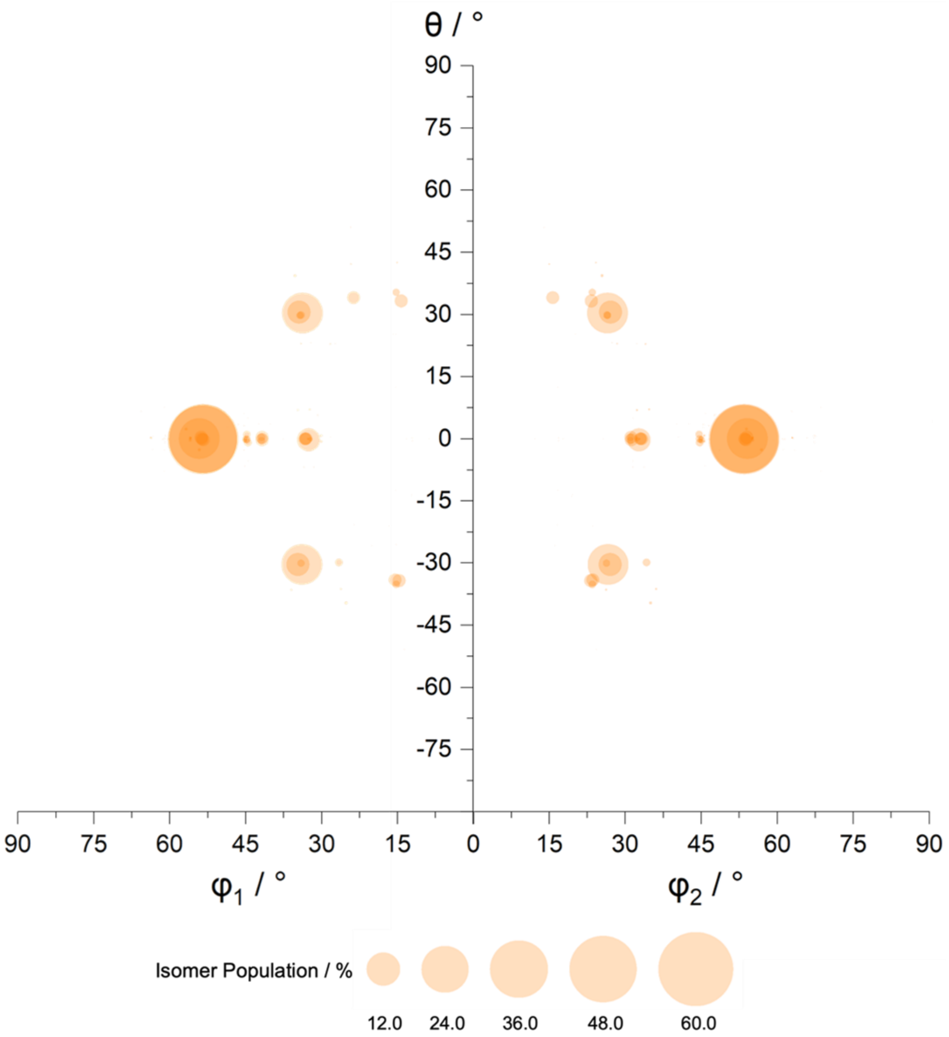

Figure S10. θ–φ_1 / θ–φ_2 plot adjusted by Boltzmann distribution at 298 K for **Me$_3$BV**.

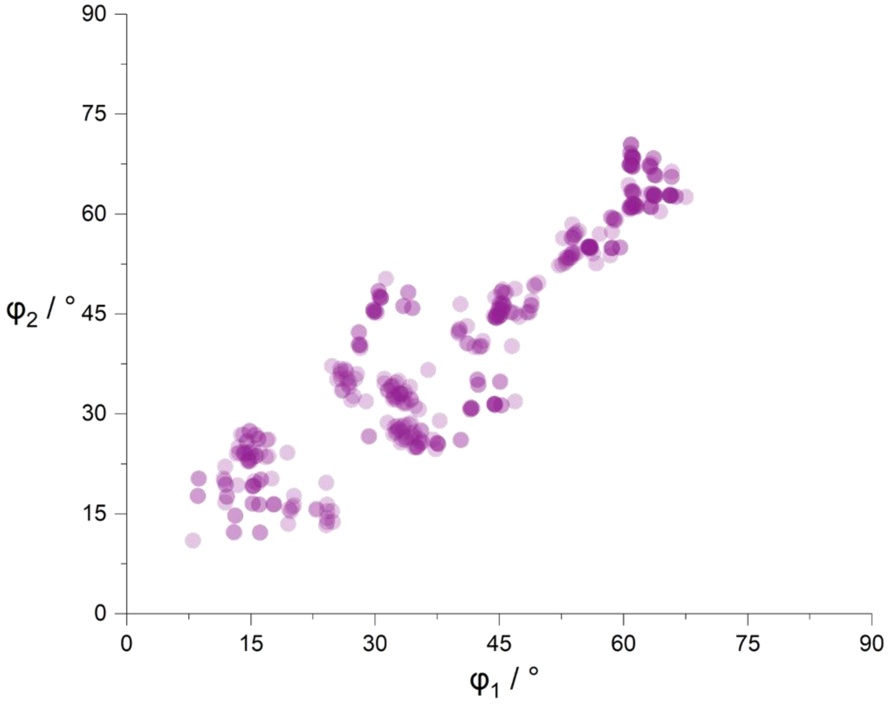

Figure S11. φ_1–φ_2 plot for **Me$_4$BV**.

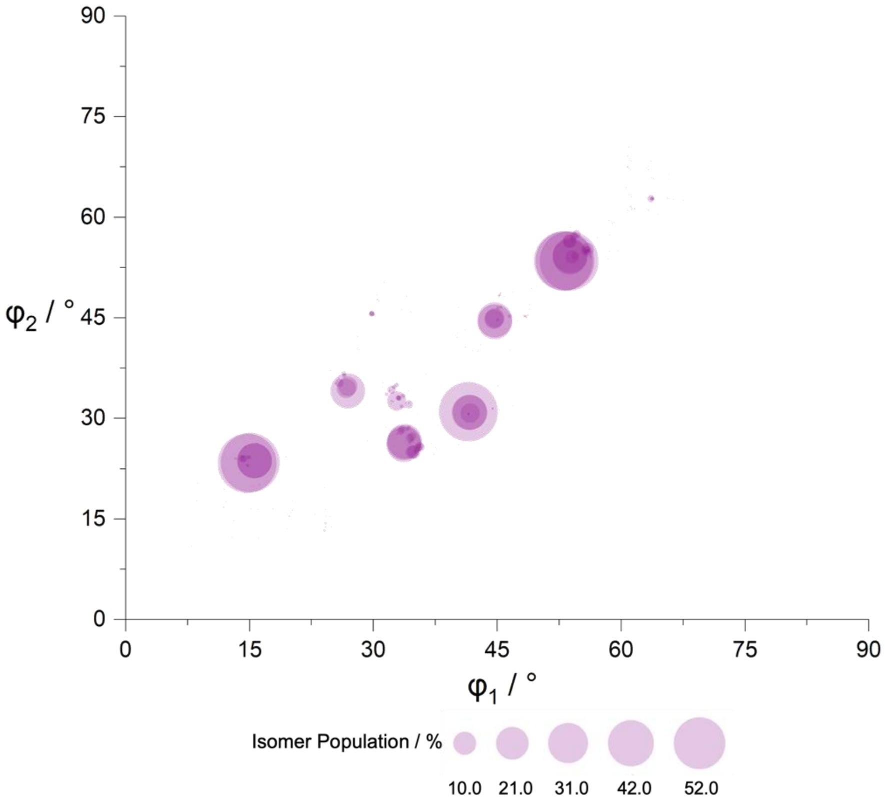

Figure S12. φ_1–φ_2 plot adjusted by Boltzmann distribution at 298 K for **Me_4BV**.

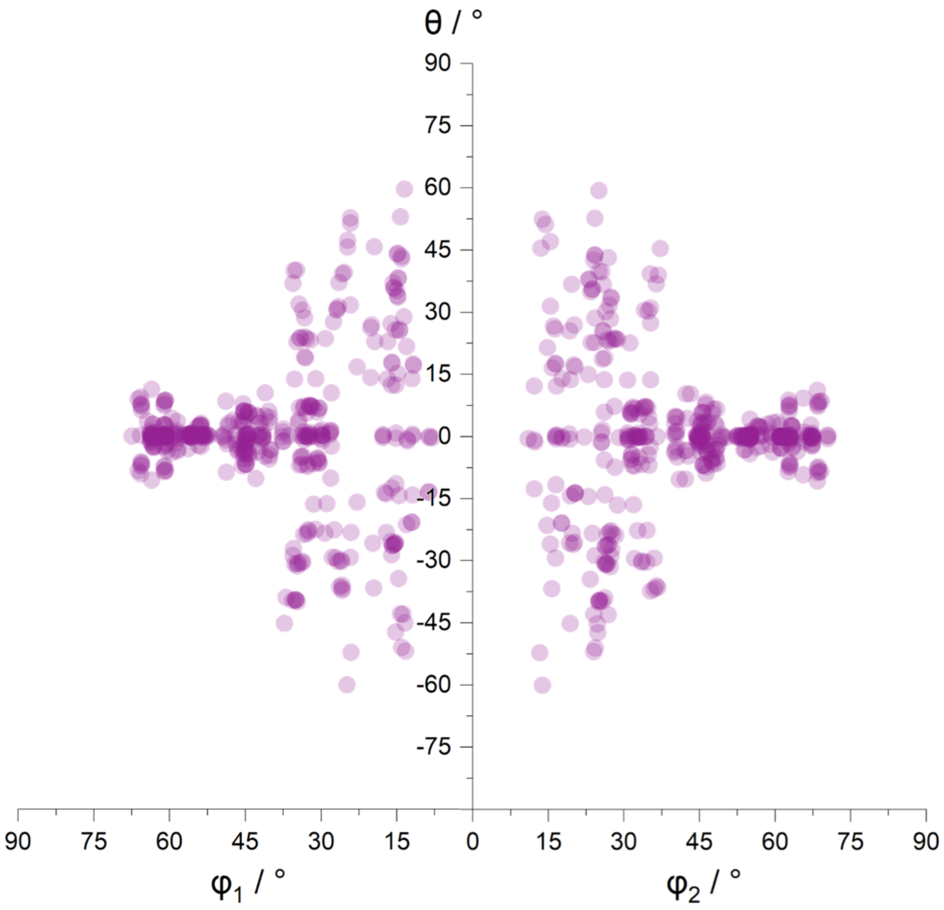

Figure S13. θ–φ_1 / θ–φ_2 plot for **Me_4BV**.

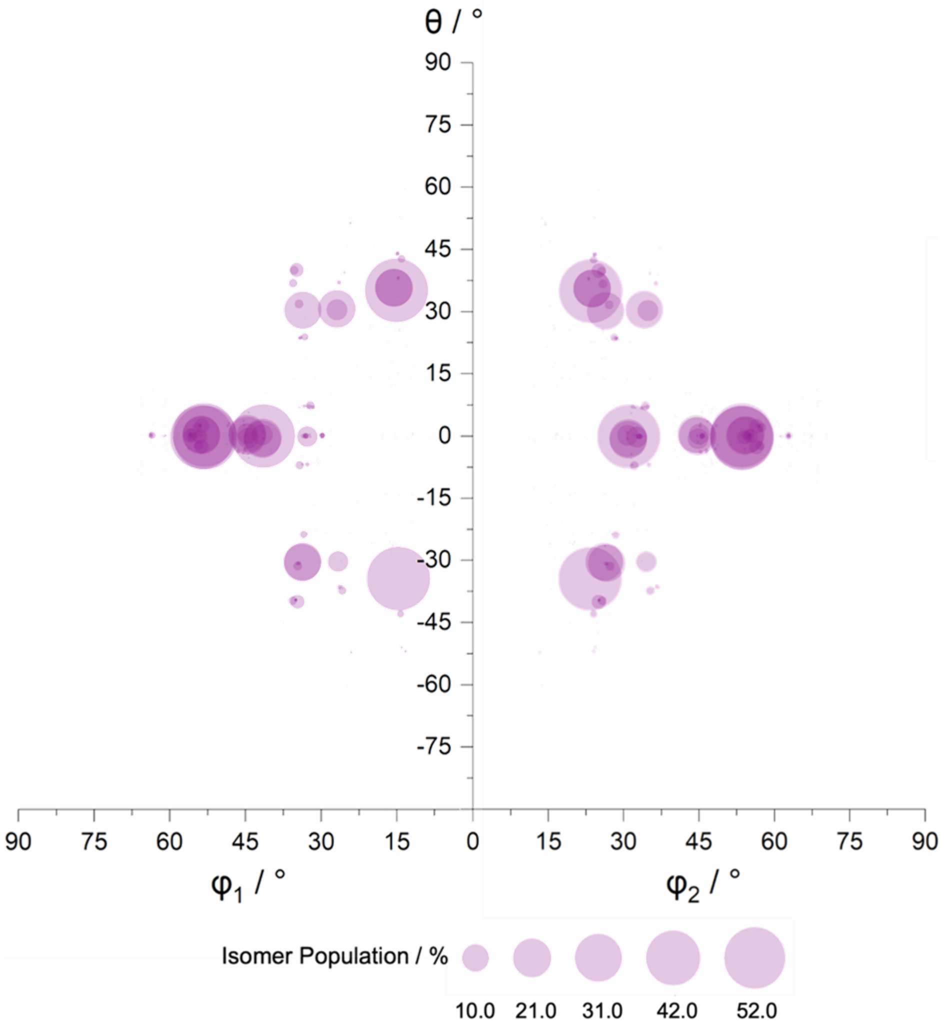

Figure S14. θ–φ_1 / θ–φ_2 plot adjusted by Boltzmann distribution at 298 K for **Me_4BV**.

.

6. DFT-Optimized Geometries

Me_2BV

Me_2BV (000 000 001 1)

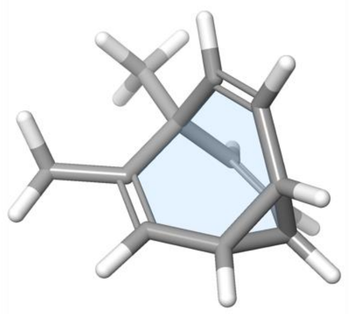

Table S12. Cartesian coordinates for the optimised geometry of **Me_2BV (000 000 001 1).**

	Coordinates / Å		
**Atom**	**x**	**y**	**z**
C	−2.215864	−0.462196	−0.621148
C	−1.524052	0.539611	−1.446268
C	−0.313541	1.082213	−1.225801
C	−2.120326	−0.475070	0.887715
C	−1.328891	0.513308	1.635904
C	−0.155268	1.060878	1.273781
C	−1.466223	−1.572000	0.079428
C	−0.000982	−1.719631	−0.014610
C	0.945474	−0.753635	−0.066298
C	0.574759	0.743544	−0.030046
C	2.395941	−1.147100	−0.161506
C	1.804514	1.680560	−0.099918
H	−3.185123	−0.735988	−1.032138
H	−2.059513	0.863052	−2.336828
H	0.061032	1.803788	−1.947157
H	−3.030305	−0.756849	1.412953
H	−1.747406	0.820986	2.592257
H	0.308076	1.770484	1.954300
H	−1.972004	−2.535135	0.103234
H	0.332984	−2.755760	−0.044598
H	2.529867	−2.234428	−0.179264
H	2.843172	−0.756047	−1.080503
H	2.955943	−0.771235	0.700242
H	1.504716	2.736704	−0.071915
H	2.375508	1.537525	−1.025281
H	2.487513	1.522423	0.743465

Me_2BV (000 000 010 1)

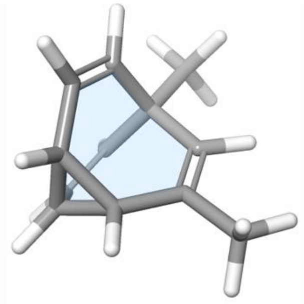

Table S13. Cartesian coordinates for the optimised geometry of **Me_2BV (000 000 010 1).**

Atom	Coordinates / Å		
	x	y	z
C	−1.328770	1.535114	0.378174
C	−0.090042	1.772121	1.138361
C	1.082847	1.127019	1.006298
C	−1.318692	1.158735	−1.087935
C	−0.069485	1.004376	−1.852237
C	1.099462	0.506539	−1.410662
C	−1.763993	0.150851	−0.050852
C	−0.978649	−1.069604	0.267861
C	0.369813	−1.155563	0.299198
C	1.314100	0.002839	0.008305
C	−1.803147	−2.295561	0.576918
C	2.768865	−0.490600	0.144981
H	−2.130480	2.203784	0.684484
H	−0.143361	2.560492	1.886667
H	1.910370	1.420674	1.646114
H	−2.114167	1.594526	−1.688760
H	−0.110538	1.334646	−2.888370
H	1.936135	0.458442	−2.102078
H	−2.838744	−0.022122	−0.013834
H	0.831222	−2.108261	0.546946
H	−1.185412	−3.170354	0.805737
H	−2.435981	−2.550893	−0.279619
H	−2.447816	−2.108850	1.442291
H	3.487626	0.313817	−0.056592
H	2.985304	−1.305911	−0.556979
H	2.973533	−0.866256	1.155584

Me_2BV (000 000 011 0)

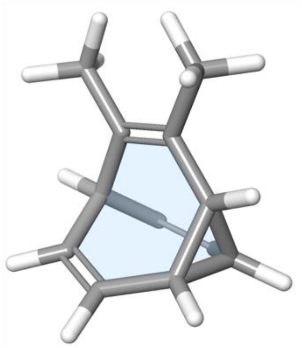

Table S14. Cartesian coordinates for the optimised geometry of **Me_2BV (000 000 011 0).**

	Coordinates / Å		
**Atom**	**x**	**y**	**z**
C	1.654576	−1.009731	0.872568
C	1.635489	0.269092	1.599938
C	1.019002	1.399957	1.218824
C	1.708947	−1.089939	−0.639386
C	1.741680	0.107470	−1.494101
C	1.102786	1.268326	−1.272673
C	0.459316	−1.518169	0.097352
C	−0.837827	−0.777445	0.000936
C	−0.975654	0.575748	−0.052203
C	0.232765	1.508560	−0.063150
C	−2.041280	−1.697138	−0.061577
C	−2.293900	1.307257	−0.086152
H	2.227129	−1.769515	1.400709
H	2.177799	0.285986	2.542963
H	1.074112	2.282261	1.848309
H	2.314472	−1.898144	−1.044029
H	2.345362	0.029927	−2.395859
H	1.201978	2.081016	−1.985179
H	0.317663	−2.596620	0.164116
H	−0.115036	2.548317	−0.133737
H	−2.362106	−1.965252	0.949907
H	−1.798018	−2.620806	−0.599123
H	−2.885916	−1.261275	−0.601428
H	−2.298024	2.110196	0.659705
H	−2.450793	1.753168	−1.073484
H	−3.154523	0.676752	0.146753

Me_2BV (000 000 100 1)

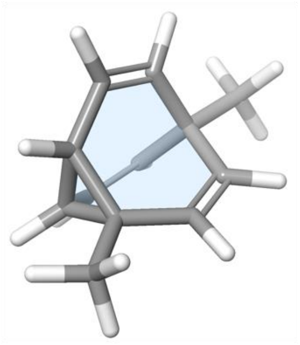

Table S15. Cartesian coordinates for the optimised geometry of **Me_2BV (000 000 100 1).**

Atom	Coordinates / Å		
	x	y	z
C	1.095092	0.517403	−1.256227
C	−0.215283	1.137828	−1.523698
C	−1.376931	0.909456	−0.885088
C	1.232465	−0.923998	−0.819938
C	0.065176	−1.804924	−0.632977
C	−1.150321	−1.468280	−0.165388
C	1.585861	0.196870	0.151082
C	0.717597	0.471532	1.331884
C	−0.621465	0.371487	1.422991
C	−1.519700	−0.061234	0.276202
C	3.062217	0.417039	0.413614
C	−2.984571	−0.055574	0.756142
H	1.848684	0.861829	−1.961915
H	−0.231018	1.859613	−2.338212
H	−2.261138	1.451390	−1.209028
H	2.070254	−1.463018	−1.258220
H	0.217386	−2.845334	−0.914108
H	−1.909107	−2.242335	−0.091004
H	1.238048	0.793868	2.232927
H	−1.092266	0.613854	2.371958
H	3.673497	0.207452	−0.471262
H	3.409456	−0.238804	1.219443
H	3.247913	1.456210	0.706375
H	−3.671074	−0.362878	−0.042948
H	−3.135081	−0.742341	1.598750
H	−3.295693	0.942890	1.088648

Me_2BV (000 000 101 0)

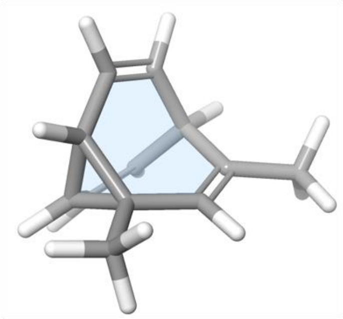

Table S16. Cartesian coordinates for the optimised geometry of **Me_2BV (000 000 101 0).**

	Coordinates / Å		
**Atom**	**x**	**y**	**z**
C	−1.432350	−0.967181	−0.506247
C	−0.429834	−1.446055	−1.476325
C	0.904529	−1.300777	−1.403324
C	−1.173931	−0.946224	0.984835
C	0.098774	−1.403183	1.573751
C	1.330082	−1.266261	1.052128
C	−1.304469	0.367285	0.220916
C	−0.110258	1.240089	0.001678
C	1.171484	0.873918	−0.215315
C	1.591755	−0.583470	−0.267665
C	−2.591674	1.139893	0.433145
C	2.262170	1.885087	−0.418559
H	−2.438552	−1.262263	−0.797775
H	−0.827708	−1.974690	−2.340227
H	1.533051	−1.702806	−2.191541
H	−2.021983	−1.228481	1.605837
H	0.017257	−1.906160	2.535236
H	2.189050	−1.649599	1.593589
H	−0.319631	2.309036	0.022937
H	2.672473	−0.640490	−0.454162
H	−2.811722	1.763719	−0.440057
H	−3.449050	0.476143	0.591066
H	−2.508052	1.788341	1.312113
H	1.888793	2.913203	−0.368303
H	3.031708	1.772777	0.352065
H	2.728088	1.748149	−1.399799

Me_2BV (000 000 110 0)

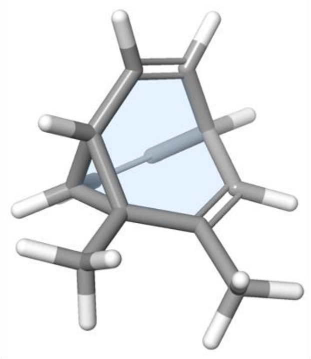

Table S17. Cartesian coordinates for the optimised geometry of **Me_2BV (000 000 110 0).**

	Coordinates / Å		
**Atom**	**x**	**y**	**z**
C	−0.030248	−1.103888	−1.179637
C	1.240034	−0.576096	−1.709609
C	2.159100	0.144722	−1.045705
C	−0.179600	−1.586910	0.245942
C	0.933988	−1.565881	1.211617
C	1.913243	−0.650405	1.301011
C	−0.924957	−0.332143	−0.209443
C	−0.499078	1.034725	0.298302
C	0.771389	1.419645	0.561826
C	1.987459	0.547747	0.393809
C	−2.418597	−0.525618	−0.431473
C	−1.586724	2.062338	0.532530
H	−0.542121	−1.708191	−1.926698
H	1.447515	−0.810158	−2.751875
H	3.060830	0.469936	−1.554382
H	−0.782428	−2.485368	0.367048
H	0.958192	−2.392671	1.918713
H	2.682205	−0.754578	2.059615
H	0.972134	2.424601	0.923359
H	2.867491	1.135574	0.685178
H	−2.796209	0.178955	−1.179570
H	−2.972505	−0.391256	0.503298
H	−2.649657	−1.533133	−0.797057
H	−2.122318	2.275562	−0.397910
H	−1.190636	3.016706	0.897393
H	−2.298504	1.705784	1.283718

Me_2BV (000 001 001 0)

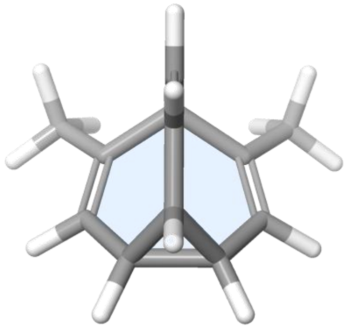

Table S18. Cartesian coordinates for the optimised geometry of **Me_2BV (000 001 001 0).**

Atom	Coordinates / Å x	y	z
C	2.123799	−0.259032	−0.869098
C	1.181220	0.135880	−1.930382
C	−0.134669	0.373852	−1.792368
C	1.724205	−1.182854	0.261535
C	0.366982	−1.751074	0.380315
C	−0.804590	−1.155268	0.073157
C	2.017341	0.274543	0.543495
C	0.965486	1.224531	0.955998
C	−0.318322	1.262329	0.540884
C	−0.861681	0.264462	−0.471124
C	−2.121435	−1.852218	0.251518
C	−1.289088	2.285987	1.052126
H	3.135678	−0.389538	−1.246522
H	1.608327	0.242472	−2.925365
H	−0.721919	0.659691	−2.659280
H	2.489805	−1.885340	0.584273
H	0.334769	−2.768524	0.765756
H	2.964340	0.473926	1.040717
H	1.287580	1.968602	1.682236
H	−1.912017	0.515098	−0.674641
H	−2.642045	−1.931003	−0.708353
H	−2.755027	−1.292962	0.947569
H	−2.002723	−2.865189	0.649974
H	−2.134093	1.794150	1.544828
H	−0.830009	2.965231	1.777966
H	−1.671912	2.892249	0.224789

Me_2BV (000 001 010 0)

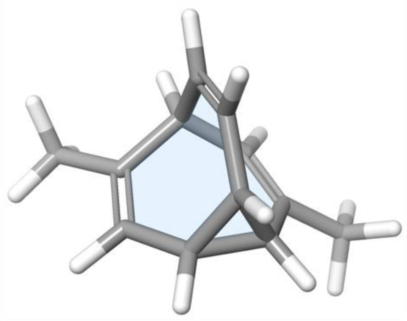

Table S19. Cartesian coordinates for the optimised geometry of **Me_2BV (000 001 010 0).**

Atom	Coordinates / Å x	y	z
C	−0.939825	−1.770774	−0.303864
C	−0.319610	−1.484578	−1.609452
C	0.468668	−0.439560	−1.915496
C	−0.240024	−1.470576	1.005033
C	1.108065	−0.872229	1.065189
C	1.632214	0.058591	0.240356
C	−1.466537	−0.678997	0.604179
C	−1.404144	0.762452	0.246703
C	−0.394888	1.358781	−0.423687
C	0.834090	0.632287	−0.917474
C	3.026642	0.583696	0.415074
C	−2.585514	1.587253	0.693026
H	−1.526046	−2.687096	−0.322381
H	−0.526720	−2.204475	−2.398512
H	0.869626	−0.343763	−2.919448
H	−0.394641	−2.202058	1.795650
H	1.731731	−1.242001	1.877035
H	−2.374614	−0.935530	1.148110
H	−0.434414	2.422897	−0.637522
H	1.453372	1.368363	−1.447627
H	3.537338	0.124852	1.268090
H	3.623734	0.379407	−0.479549
H	3.006461	1.665547	0.581818
H	−2.685599	1.545475	1.782753
H	−2.492477	2.640502	0.407910
H	−3.506890	1.201534	0.244083

Me_2BV (000 001 100 0)

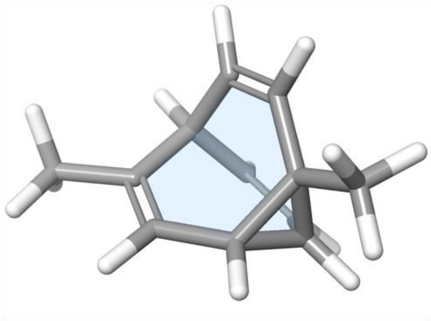

Table S20. Cartesian coordinates for the optimised geometry of **Me_2BV (000 001 100 0).**

Atom	Coordinates / Å		
	x	y	z
C	−1.212618	0.656815	1.061179
C	−0.187810	1.715440	1.130914
C	0.974814	1.765492	0.457921
C	−0.853552	−0.801842	0.882368
C	0.544132	−1.267231	0.765460
C	1.577622	−0.642074	0.163239
C	−1.579213	−0.059891	−0.235084
C	−0.868584	0.265329	−1.505857
C	0.424777	0.597924	−1.665651
C	1.402913	0.701231	−0.522265
C	2.955359	−1.235003	0.126825
C	−3.044620	−0.397045	−0.424129
H	−2.031402	0.851596	1.751213
H	−0.406504	2.536325	1.810918
H	1.647034	2.604350	0.607543
H	−1.453588	−1.499899	1.463380
H	0.738092	−2.230688	1.233673
H	−1.473517	0.224587	−2.410551
H	0.804319	0.805546	−2.661286
H	2.365903	1.017141	−0.945133
H	3.274537	−1.389847	−0.908920
H	3.002993	−2.202180	0.637985
H	3.669662	−0.564678	0.615754
H	−3.538825	−0.631902	0.525178
H	−3.155436	−1.267504	−1.079936
H	−3.576489	0.448009	−0.874739

Me$_2$BV (000 010 001 0)

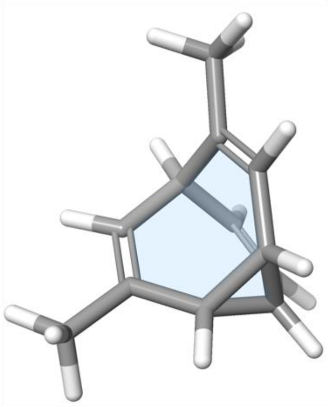

Table S21. Cartesian coordinates for the optimised geometry of **Me$_2$BV (000 010 001 0).**

Atom	Coordinates / Å		
	x	y	z
C	0.567453	−1.397906	1.354658
C	−0.396030	−0.569904	2.101148
C	−1.105139	0.468258	1.625235
C	1.486778	−0.823492	0.296907
C	1.492468	0.618157	−0.065129
C	0.404115	1.416035	−0.115204
C	0.363980	−1.757086	−0.102270
C	−0.809774	−1.304744	−0.874714
C	−1.450088	−0.120475	−0.779779
C	−0.998019	0.949613	0.198610
C	2.846892	1.191804	−0.399198
C	−2.639946	0.212598	−1.630656
H	0.999169	−2.180945	1.974429
H	−0.541829	−0.840325	3.144881
H	−1.790000	0.997476	2.280029
H	2.481766	−1.265529	0.267294
H	0.504699	2.460665	−0.394960
H	0.670761	−2.762777	−0.382480
H	−1.182919	−2.028656	−1.596862
H	−1.657918	1.822382	0.102767
H	3.522044	1.089914	0.456984
H	3.283000	0.659799	−1.251284
H	2.798843	2.254332	−0.659983
H	−2.434087	1.098061	−2.240729
H	−2.904823	−0.604955	−2.309207
H	−3.511395	0.417702	−1.000487

Me_2BV (000 010 010 0)

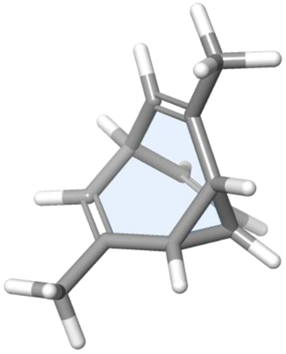

Table S22. Cartesian coordinates for the optimised geometry of **Me_2BV (000 010 010 0).**

Atom	Coordinates / Å		
	x	y	z
C	−0.216106	0.101265	1.766602
C	−0.413594	1.544778	1.546636
C	−0.379730	2.192225	0.369141
C	−0.715134	−0.946888	0.793115
C	−1.446169	−0.601494	−0.454209
C	−1.198728	0.473619	−1.232817
C	0.772457	−0.717641	0.961949
C	1.622262	−0.128630	−0.105959
C	1.243145	0.849926	−0.955677
C	−0.122897	1.488639	−0.938465
C	−2.545729	−1.558612	−0.841442
C	3.013477	−0.701907	−0.210503
H	−0.294704	−0.163761	2.818979
H	−0.606084	2.136591	2.439080
H	−0.541513	3.264849	0.338177
H	−1.091627	−1.857739	1.256944
H	−1.785912	0.647509	−2.129705
H	1.291596	−1.490468	1.527429
H	1.933521	1.220694	−1.707570
H	−0.148977	2.244140	−1.734513
H	−3.053183	−1.261593	−1.765322
H	−3.300028	−1.608313	−0.049090
H	−2.136103	−2.562522	−0.995198
H	3.555756	−0.551778	0.728995
H	3.599052	−0.236463	−1.010345
H	2.964950	−1.776426	−0.416231

Me_2BV (000 010 100 0)

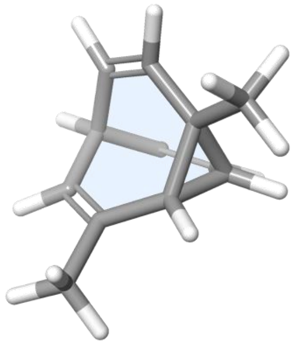

Table S23. Cartesian coordinates for the optimised geometry of **Me_2BV (000 010 100 0).**

Atom	Coordinates / Å		
	x	y	z
C	−0.865117	−0.459545	1.173923
C	−0.855916	0.904833	1.734142
C	−0.348754	2.012084	1.165381
C	0.273477	−1.003372	0.338277
C	1.490150	−0.207628	0.019450
C	1.522243	1.122424	−0.211655
C	−1.084929	−0.748053	−0.308773
C	−1.264435	0.371896	−1.278066
C	−0.679108	1.582525	−1.256878
C	0.300140	2.004821	−0.193912
C	2.775181	−0.996523	−0.036451
C	−1.911744	−1.975308	−0.637846
H	−1.306237	−1.169135	1.871549
H	−1.309191	1.011724	2.717601
H	−0.404697	2.961139	1.688745
H	0.513418	−2.048750	0.530449
H	2.462989	1.616880	−0.435376
H	−1.954128	0.181803	−2.099205
H	−0.908782	2.302806	−2.035781
H	0.620800	3.030064	−0.419258
H	2.968824	−1.472787	0.930479
H	2.705981	−1.777316	−0.801232
H	3.640854	−0.370788	−0.278023
H	−1.638971	−2.366976	−1.623877
H	−1.763320	−2.779652	0.091242
H	−2.978726	−1.727165	−0.644905

Me_2BV (000 100 001 0)

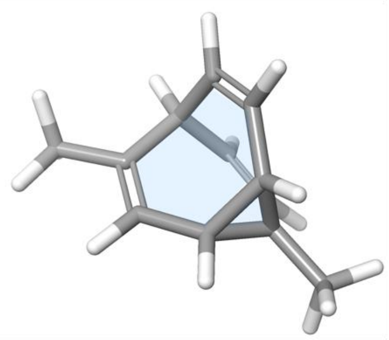

Table S24. Cartesian coordinates for the optimised geometry of **Me_2BV (000 100 001 0).**

	Coordinates / Å		
**Atom**	**x**	**y**	**z**
C	1.229892	0.405406	−1.162303
C	0.287519	−0.344677	−2.013825
C	−0.849081	−0.950429	−1.628695
C	1.575834	−0.014672	0.263255
C	0.927085	−1.209196	0.877959
C	−0.332215	−1.646766	0.700101
C	0.765349	1.250722	0.003125
C	−0.660121	1.384130	0.369204
C	−1.625967	0.444392	0.294195
C	−1.330411	−0.960818	−0.198697
C	3.015781	0.185963	0.691188
C	−3.043507	0.725018	0.697780
H	2.042044	0.830608	−1.748911
H	0.549714	−0.399781	−3.068482
H	−1.459017	−1.466354	−2.363371
H	1.551582	−1.792321	1.553268
H	−0.667470	−2.537447	1.222346
H	1.294176	2.193720	0.129615
H	−0.939890	2.370038	0.736367
H	−2.254529	−1.553444	−0.171024
H	3.464501	1.070102	0.224789
H	3.077564	0.316468	1.777105
H	3.622802	−0.681485	0.409924
H	−3.345230	0.058351	1.512111
H	−3.178886	1.755164	1.043401
H	−3.717520	0.567306	−0.150424

Me_2BV (000 100 010 0)

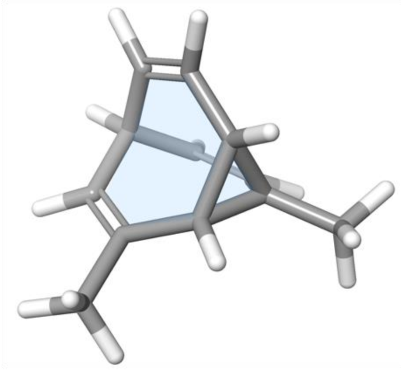

Table S25. Cartesian Coordinates for the optimised geometry of **Me_2BV (000 100 010 0).**

	Coordinates / Å		
**Atom**	**x**	**y**	**z**
C	−0.753022	−0.353741	−1.282780
C	−0.739344	1.061275	−1.698712
C	−0.313317	2.114176	−0.979957
C	−1.083481	−0.799161	0.139310
C	−1.374382	0.210077	1.198956
C	−0.825788	1.428653	1.349940
C	0.329913	−0.956764	−0.414412
C	1.492377	−0.172462	0.084285
C	1.465790	1.126820	0.451218
C	0.223260	1.979864	0.421057
C	−1.897105	−2.071392	0.271639
C	2.791709	−0.935137	0.167037
H	−1.114724	−0.996432	−2.083347
H	−1.114463	1.259864	−2.700672
H	−0.355084	3.110984	−1.407008
H	−2.122323	−0.078934	1.935935
H	−1.139277	2.059148	2.176043
H	0.615836	−1.971174	−0.691064
H	2.370087	1.615474	0.801935
H	0.493758	2.982840	0.775450
H	−1.693494	−2.557297	1.232110
H	−2.968104	−1.848275	0.214592
H	−1.666084	−2.792385	−0.520426
H	3.615836	−0.319405	0.542511
H	3.077221	−1.304256	−0.823675
H	2.684205	−1.792359	0.840037

Me_2BV (000 100 100 0)

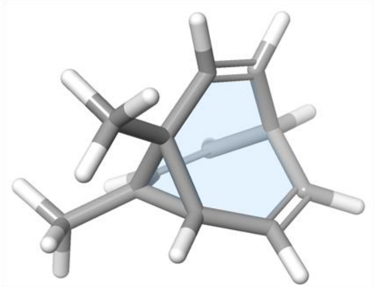

Table S26. Cartesian coordinates for the optimised geometry of **Me_2BV (000 100 100 0).**

	Coordinates / Å		
**Atom**	**x**	**y**	**z**
C	−0.592515	0.102612	1.099236
C	0.614058	0.065916	1.948993
C	1.892511	−0.045429	1.550268
C	−0.744489	−0.736658	−0.164583
C	0.382311	−1.598691	−0.644400
C	1.705188	−1.386002	−0.530942
C	−0.636710	0.809750	−0.250841
C	0.594739	1.449223	−0.814410
C	1.876328	1.069514	−0.667908
C	2.289892	−0.153850	0.103027
C	−2.100840	−1.390695	−0.375270
C	−1.891166	1.617697	−0.543082
H	−1.485140	0.199185	1.715187
H	0.432979	0.138261	3.019739
H	2.687313	−0.059608	2.289200
H	0.088348	−2.520958	−1.144244
H	2.401285	−2.116455	−0.931318
H	0.427295	2.342239	−1.415510
H	2.663641	1.647844	−1.141288
H	3.383431	−0.232687	0.056025
H	−2.086951	−2.427576	−0.019909
H	−2.907027	−0.887152	0.166333
H	−2.356365	−1.395341	−1.440348
H	−2.151790	1.539733	−1.604076
H	−1.731281	2.675590	−0.304556
H	−2.755046	1.293538	0.044678

Me_3BV

Me_3BV (000 000 011 1)

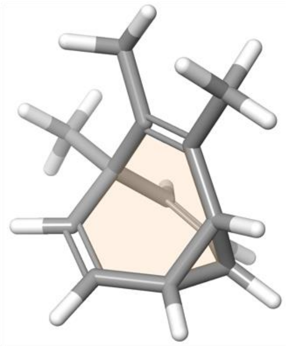

Table S27. Coordinates for the optimised geometry of **Me_3BV (000 000 011 1).**

	Coordinates / Å		
**Atom**	**x**	**y**	**z**
C	1.044945	−1.895300	−0.934283
C	−0.149888	−1.561184	−1.716014
C	−1.151927	−0.744326	−1.352819
C	1.020227	−2.046579	0.569295
C	−0.200288	−1.877038	1.364302
C	−1.194794	−1.002074	1.144731
C	1.735280	−0.875516	−0.060983
C	1.269928	0.539936	0.066488
C	−0.026155	0.970662	0.107866
C	−1.221484	−0.012564	−0.014926
C	2.413595	1.537227	0.140160
C	−0.381061	2.431680	0.287574
C	−2.612816	0.671847	0.026803
H	1.702512	−2.582272	−1.462765
H	−0.213711	−2.022668	−2.699452
H	−1.960418	−0.586398	−2.061983
H	1.663456	−2.827453	0.969885
H	−0.293398	−2.530942	2.229170
H	−2.027362	−0.991531	1.843286
H	2.820907	−0.964994	−0.040529
H	2.504783	1.937555	1.155140
H	3.376149	1.084600	−0.120253
H	2.271062	2.358916	−0.569575
H	−0.905262	2.808599	−0.596156
H	0.479568	3.085180	0.446217
H	−1.016866	2.565402	1.168431
H	−2.788273	1.191619	0.976286
H	−2.736164	1.400173	−0.783800
H	−3.422541	−0.062557	−0.082095

Me_3BV (000 000 101 1)

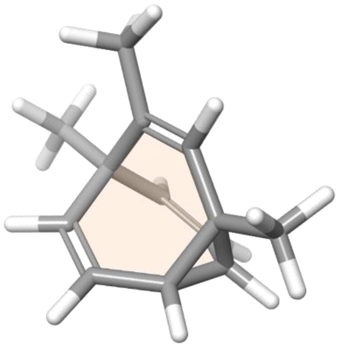

Table S28. Coordinates for the optimised geometry of **Me_3BV (000 000 101 1).**

	Coordinates / Å		
**Atom**	**x**	**y**	**z**
C	1.456745	−0.908781	1.034451
C	0.209793	−0.872438	1.813330
C	−1.025921	−0.587897	1.368116
C	1.505376	−1.383473	−0.398555
C	0.309334	−1.844059	−1.119808
C	−0.945260	−1.375220	−1.008668
C	1.752236	0.084611	−0.080539
C	0.746264	1.122012	−0.458322
C	−0.605229	1.047287	−0.479433
C	−1.354720	−0.237083	−0.079415
C	3.188290	0.559972	−0.189268
C	−1.401479	2.253209	−0.905917
C	−2.894104	−0.106799	−0.174815
H	2.302477	−1.208316	1.650422
H	0.314670	−1.100183	2.872460
H	−1.841194	−0.594376	2.086827
H	2.380926	−1.974073	−0.661248
H	0.474094	−2.656338	−1.825272
H	−1.715063	−1.825548	−1.629849
H	1.186980	2.072648	−0.758265
H	3.903242	−0.220724	0.093592
H	3.415026	0.859940	−1.218205
H	3.357763	1.418894	0.469239
H	−2.002896	2.025918	−1.791444
H	−0.760951	3.103321	−1.165782
H	−2.060351	2.586756	−0.098353
H	−3.393143	−1.040424	0.117526
H	−3.279984	0.679086	0.485719
H	−3.222920	0.122077	−1.195756

Me_3BV (000 000 110 1)

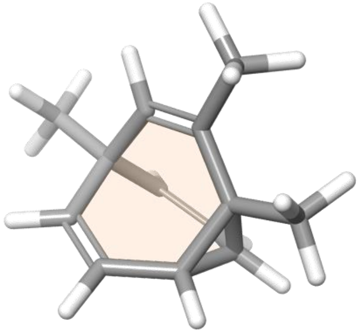

Table S29. Coordinates for the optimised geometry of **Me_3BV (000 000 110 1).**

	Coordinates / Å		
**Atom**	**x**	**y**	**z**
C	0.833112	−1.615286	0.355018
C	−0.327672	−1.693028	1.256424
C	−1.451188	−0.957581	1.209081
C	0.699130	−1.301332	−1.116700
C	−0.602005	−1.050190	−1.756989
C	−1.672562	−0.438844	−1.222595
C	1.388716	−0.301812	−0.190811
C	0.711241	1.011766	0.151086
C	−0.619326	1.203912	0.313207
C	−1.688668	0.133484	0.182207
C	2.905276	−0.258853	−0.319714
C	1.601017	2.225765	0.329064
C	−3.070989	0.766807	0.443155
H	1.566543	−2.382519	0.597347
H	−0.258629	−2.442408	2.042730
H	−2.223782	−1.142320	1.950148
H	1.350874	−1.877159	−1.771637
H	−0.697525	−1.413947	−2.778346
H	−2.567856	−0.336075	−1.829305
H	−0.978317	2.200905	0.558576
H	3.209826	0.425791	−1.117948
H	3.368194	0.054704	0.621590
H	3.321878	−1.242353	−0.567447
H	1.034194	3.131072	0.573794
H	2.153284	2.436986	−0.592056
H	2.311532	2.066159	1.146236
H	−3.875822	0.025558	0.358293
H	−3.131845	1.201694	1.448933
H	−3.288634	1.569106	−0.273342

Me_3BV (000 000 111 0)

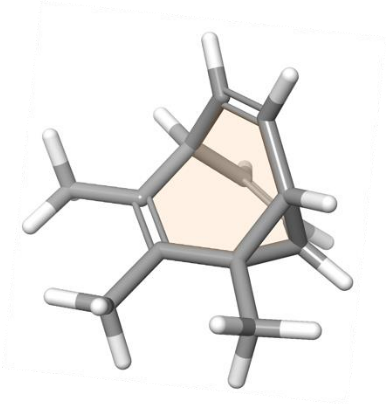

Table S30. Coordinates for the optimised geometry of **Me_3BV (000 000 111 0).**

	Coordinates / Å		
**Atom**	**x**	**y**	**z**
C	−0.158579	1.815982	0.688506
C	1.247305	1.652613	1.084038
C	2.142847	0.810957	0.547346
C	−0.636234	1.687565	−0.739731
C	0.271371	1.379250	−1.854280
C	1.355108	0.589074	−1.813956
C	−1.067335	0.652675	0.295741
C	−0.527759	−0.783481	0.222347
C	0.744546	−1.144277	−0.118086
C	1.792914	−0.133713	−0.567424
C	−2.502706	0.808803	0.789919
C	−1.552123	−1.866346	0.532731
C	1.279874	−2.557521	−0.090463
H	−0.623811	2.622098	1.253747
H	1.586941	2.291729	1.896564
H	3.157427	0.784116	0.931179
H	−1.394093	2.409268	−1.039759
H	0.030004	1.850010	−2.805045
H	1.945589	0.439962	−2.712302
H	2.718635	−0.661805	−0.834235
H	−2.634070	0.338481	1.769768
H	−3.210977	0.374136	0.077433
H	−2.782957	1.862892	0.903653
H	−2.482884	−1.690354	−0.015383
H	−1.767086	−1.889136	1.605655
H	−1.247207	−2.869824	0.228891
H	0.720525	−3.225524	0.568091
H	1.289118	−2.977340	−1.101040
H	2.305616	−2.570288	0.296095

Me_3BV (000 001 001 1)

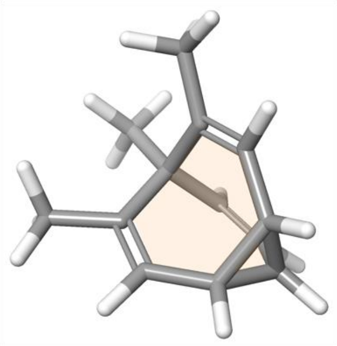

Table S31. Coordinates for the optimised geometry of **Me_3BV (000 001 001 1).**

Atom	Coordinates / Å x	y	z
C	−2.506932	−0.517048	0.009709
C	−1.843724	−0.701972	1.303964
C	−0.545428	−0.505742	1.587319
C	−1.820918	−0.845895	−1.293527
C	−0.445257	−1.366285	−1.360744
C	0.617730	−1.055200	−0.584279
C	−2.115911	0.591074	−0.937047
C	−1.045093	1.555655	−0.635877
C	0.124099	1.349397	0.012243
C	0.514909	−0.038502	0.581300
C	1.946755	−1.701197	−0.881920
C	1.083858	2.502217	0.160835
C	1.832782	0.028386	1.402239
H	−3.569484	−0.745788	0.052476
H	−2.485705	−1.035458	2.117010
H	−0.224232	−0.693297	2.609148
H	−2.459442	−1.278428	−2.061090
H	−0.290955	−2.086952	−2.162546
H	−2.937409	1.049858	−1.483494
H	−1.244660	2.558761	−1.010055
H	2.700743	−0.942691	−1.114069
H	1.897393	−2.373174	−1.746084
H	2.286664	−2.301920	−0.032994
H	0.708541	3.418052	−0.309437
H	2.041002	2.271064	−0.316832
H	1.252690	2.734899	1.216504
H	2.686892	0.353577	0.798173
H	1.748939	0.724246	2.246913
H	2.092154	−0.947636	1.832162

Me$_3$BV (000 001 010 1)

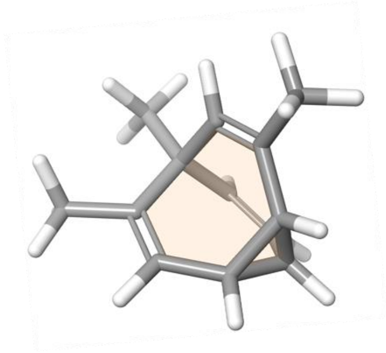

Table S32. Coordinates for the optimised geometry of **Me$_3$BV (000 001 010 1).**

	Coordinates / Å		
**Atom**	**x**	**y**	**z**
C	−1.293467	−1.435462	1.225440
C	−0.330354	−0.760624	2.105917
C	0.625224	0.113637	1.747114
C	−0.921799	−1.885925	−0.168639
C	0.421711	−1.676799	−0.737850
C	1.256647	−0.626396	−0.567651
C	−1.910015	−0.759040	0.022626
C	−1.598037	0.638384	−0.360755
C	−0.392880	1.236952	−0.246137
C	0.866385	0.576648	0.313104
C	2.596389	−0.630378	−1.255040
C	−2.757474	1.416819	−0.933758
C	1.962805	1.668528	0.372907
H	−1.952420	−2.104570	1.774656
H	−0.403539	−1.012711	3.162040
H	1.269826	0.513124	2.525632
H	−1.352153	−2.834925	−0.482367
H	0.759817	−2.493009	−1.374530
H	−2.949219	−1.024218	−0.167184
H	−0.291063	2.267397	−0.578450
H	2.682090	0.218473	−1.940308
H	2.754198	−1.537180	−1.849487
H	3.407469	−0.585213	−0.521833
H	−3.135013	0.923403	−1.835629
H	−3.570943	1.475198	−0.202851
H	−2.482297	2.441123	−1.206320
H	2.900704	1.283855	0.791527
H	1.654416	2.510887	1.006567
H	2.182990	2.082022	−0.618745

Me_3BV (000 001 011 0)

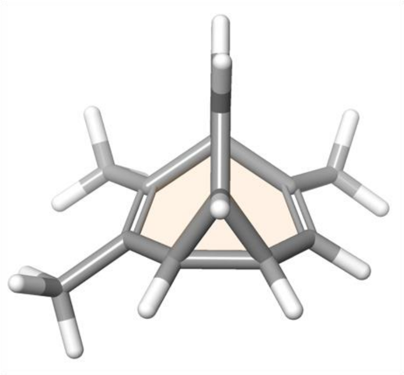

Table S33. Coordinates for the optimised geometry of **Me_3BV (000 001 011 0).**

		Coordinates / Å	
**Atom**	**x**	**y**	**z**
C	0.009651	−2.126014	−0.896654
C	−0.345681	−1.244219	−2.017825
C	−0.634033	0.065202	−1.945194
C	−0.609582	−1.979783	0.477025
C	−1.615014	−0.951943	0.794868
C	−1.670292	0.310295	0.325268
C	0.850825	−1.651412	0.266802
C	1.403160	−0.265382	0.363356
C	0.758239	0.871181	−0.015373
C	−0.632080	0.839008	−0.648215
C	−2.750538	1.266703	0.736619
C	2.811714	−0.226183	0.922040
C	1.303359	2.267074	0.154443
H	0.193457	−3.147897	−1.221470
H	−0.373131	−1.711761	−2.999729
H	−0.881184	0.611281	−2.850038
H	−0.806943	−2.913672	0.999792
H	−2.387777	−1.271238	1.491643
H	1.533035	−2.399700	0.669559
H	−0.914318	1.866341	−0.919064
H	−3.455482	0.815493	1.442632
H	−2.313910	2.147115	1.219227
H	−3.320330	1.594892	−0.138720
H	3.391046	0.628479	0.563097
H	2.781644	−0.205067	2.015918
H	3.378080	−1.112090	0.612679
H	1.570184	2.687491	−0.820394
H	0.546558	2.913258	0.613580
H	2.179342	2.322550	0.804128

Me$_3$BV (000 001 100 1)

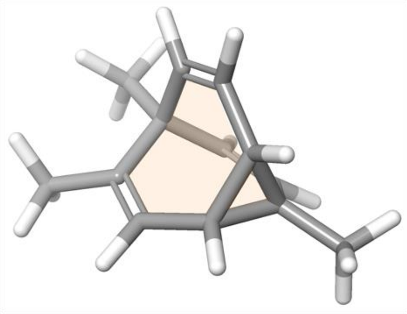

Table S34. Coordinates for the optimised geometry of **Me$_3$BV (000 001 100 1).**

**Atom**	**Coordinates / Å** x	y	z
C	−1.351602	−0.010190	−1.504190
C	−0.321529	0.995108	−1.807225
C	0.734633	1.339341	−1.050636
C	−1.027096	−1.287138	−0.766320
C	0.335093	−1.609099	−0.298442
C	1.288509	−0.777163	0.179629
C	−1.905847	−0.235498	−0.103837
C	−1.385985	0.547473	1.048987
C	−0.129937	0.976893	1.264262
C	1.040425	0.736961	0.316961
C	2.621285	−1.342891	0.593520
C	−3.384286	−0.555494	−0.010836
C	2.254497	1.507140	0.890005
H	−2.059358	−0.114763	−2.324281
H	−0.432106	1.502294	−2.763867
H	1.413783	2.095320	−1.435977
H	−1.537371	−2.175394	−1.134685
H	0.578592	−2.668661	−0.363314
H	−2.116898	0.794994	1.817839
H	0.067448	1.528514	2.179947
H	2.674050	−2.427637	0.448341
H	3.428992	−0.902688	0.000777
H	2.808931	−1.154059	1.654943
H	−3.735454	−1.134331	−0.872378
H	−3.592903	−1.142633	0.890198
H	−3.973401	0.367150	0.031101
H	2.042517	2.581274	0.976356
H	2.525307	1.156089	1.893121
H	3.139714	1.409091	0.249998

Me_3BV (000 001 101 0)

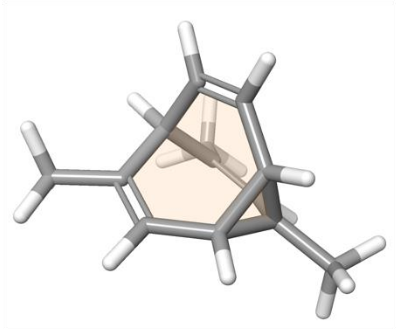

Table S35. Coordinates for the optimised geometry of **Me_3BV (000 001 101 0).**

Atom	Coordinates / Å x	y	z
C	−1.404385	−1.323841	−0.639221
C	−0.606923	−1.070860	−1.852005
C	0.515558	−0.338389	−1.944962
C	−0.771309	−1.487781	0.724345
C	0.686772	−1.406510	0.940093
C	1.572704	−0.608915	0.308632
C	−1.634719	−0.265953	0.434327
C	−1.013290	1.087075	0.314771
C	0.191122	1.416131	−0.198512
C	1.136776	0.372097	−0.766457
C	3.037237	−0.632505	0.634332
C	−3.009329	−0.250048	1.073897
C	0.674399	2.836943	−0.237758
H	−2.239736	−1.990253	−0.845467
H	−0.975740	−1.537327	−2.763236
H	1.010134	−0.233626	−2.905387
H	−1.220335	−2.254965	1.352574
H	1.067502	−2.081972	1.704150
H	−1.623869	1.903070	0.699714
H	2.029883	0.884474	−1.150236
H	3.364735	0.353384	0.979982
H	3.274605	−1.356621	1.420643
H	3.618827	−0.902335	−0.253106
H	−3.440075	−1.254711	1.149502
H	−3.698932	0.362728	0.483113
H	−2.955364	0.164552	2.086458
H	0.861800	3.145377	−1.271394
H	1.604929	2.936472	0.330538
H	−0.052979	3.534308	0.190669

Me_3BV (000 001 110 0)

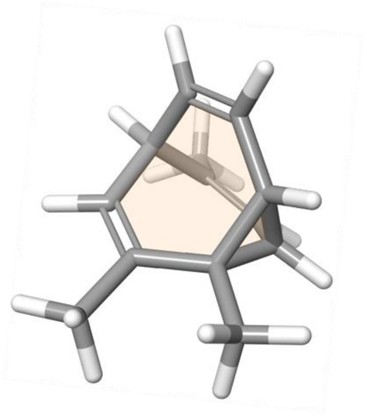

Table S36. Coordinates for the optimised geometry of **Me_3BV (000 001 110 0).**

**Atom**	**Coordinates / Å** x	y	z
C	−0.517303	−1.522595	−0.857428
C	0.361231	−2.203574	0.108686
C	1.217383	−1.629123	0.969125
C	−0.109903	−0.251874	−1.567267
C	1.194425	0.401838	−1.347783
C	1.899932	0.473010	−0.201123
C	−1.254904	−0.218062	−0.556509
C	−1.067455	0.474595	0.780257
C	0.080319	0.512135	1.495180
C	1.376523	−0.136254	1.083192
C	3.228416	1.165177	−0.122347
C	−2.643768	−0.117058	−1.171782
C	−2.264253	1.190775	1.370399
H	−1.051168	−2.243969	−1.473749
H	0.309243	−3.290461	0.107183
H	1.818734	−2.248942	1.626269
H	−0.396498	−0.199971	−2.616468
H	1.620674	0.867463	−2.234658
H	0.112888	1.040457	2.444267
H	2.101501	0.049453	1.887219
H	3.535416	1.581741	−1.087343
H	3.185083	1.989130	0.597272
H	4.003859	0.462013	0.198376
H	−2.702922	−0.639366	−2.133984
H	−2.912189	0.927627	−1.359583
H	−3.394520	−0.570670	−0.516583
H	−2.038567	1.660966	2.334153
H	−3.086159	0.489081	1.544060
H	−2.606017	1.986457	0.700971

Me_3BV (000 010 001 1)

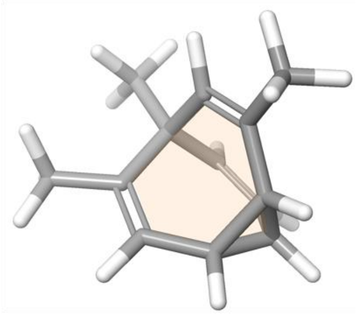

Table S37. Coordinates for the optimised geometry of **Me_3BV (000 010 001 1).**

	Coordinates / Å		
**Atom**	**x**	**y**	**z**
C	1.421743	−0.078832	−1.791019
C	0.535168	1.086310	−1.912094
C	−0.461804	1.438991	−1.082717
C	1.914344	−0.592250	−0.457531
C	1.548489	0.040889	0.831806
C	0.350889	0.589951	1.128636
C	0.928580	−1.406358	−1.262839
C	−0.464793	−1.620310	−0.832153
C	−1.295001	−0.759251	−0.200676
C	−0.841587	0.664702	0.176315
C	2.638639	0.038354	1.875911
C	−2.692597	−1.201208	0.144034
C	−1.942961	1.484919	0.891951
H	2.138202	−0.135738	−2.607726
H	0.709657	1.720587	−2.779011
H	−1.036874	2.328720	−1.325345
H	2.935053	−0.972258	−0.463718
H	0.204171	1.011234	2.120406
H	1.341240	−2.285786	−1.753300
H	−0.849054	−2.611588	−1.067920
H	2.937684	−0.989412	2.107502
H	2.324967	0.511739	2.812248
H	3.515574	0.581041	1.507348
H	−2.892160	−2.230208	−0.175172
H	−2.853487	−1.166579	1.225884
H	−3.432640	−0.565100	−0.351125
H	−2.260079	1.013213	1.829908
H	−1.588934	2.492675	1.147474
H	−2.832429	1.611555	0.262919

Me_3BV (000 010 010 1)

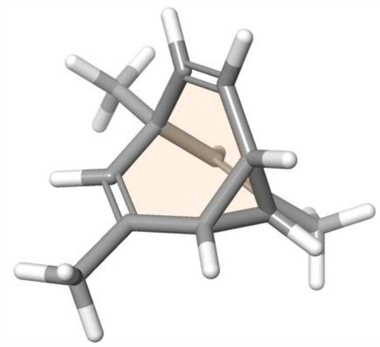

Table S38. Coordinates for the optimised geometry of **Me_3BV (000 010 010 1).**

	Coordinates / Å		
**Atom**	**x**	**y**	**z**
C	0.095046	−1.028287	1.759368
C	−0.400880	0.296580	2.162581
C	−0.639443	1.352005	1.364895
C	1.114657	−1.213600	0.656708
C	1.696139	−0.075095	−0.097328
C	1.040070	1.050148	−0.455002
C	−0.308652	−1.685783	0.457626
C	−1.238666	−1.048718	−0.507832
C	−1.305513	0.271999	−0.783091
C	−0.421388	1.328632	−0.138596
C	3.148892	−0.233936	−0.474370
C	−2.171346	−1.998927	−1.218531
C	−0.799467	2.710225	−0.712437
H	0.198982	−1.699860	2.609131
H	−0.591518	0.422632	3.226524
H	−1.006918	2.269714	1.815459
H	1.837867	−2.008742	0.833689
H	1.574297	1.818502	−1.008167
H	−0.442679	−2.765312	0.514704
H	−2.031448	0.622293	−1.512522
H	3.761945	−0.352219	0.425356
H	3.535515	0.627802	−1.028590
H	3.280910	−1.120069	−1.104195
H	−2.797036	−2.528173	−0.492072
H	−1.596612	−2.738181	−1.786443
H	−2.838153	−1.486659	−1.920085
H	−0.185812	3.509064	−0.276893
H	−0.659428	2.746784	−1.800306
H	−1.849362	2.957181	−0.509582

Me_3BV (000 010 011 0)

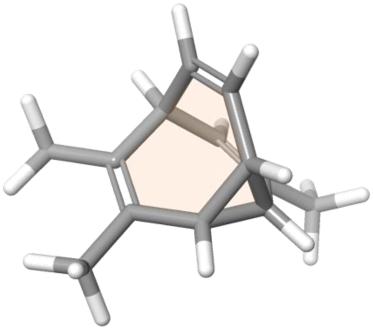

Table S39. Coordinates for the optimised geometry of **Me_3BV (000 010 011 0).**

Atom	Coordinates / Å		
	x	y	z
C	0.852672	−1.162445	1.482374
C	0.226591	−0.153868	2.349262
C	−0.389147	0.971144	1.952511
C	1.598980	−0.806311	0.213324
C	1.760820	0.589423	−0.260469
C	0.833139	1.561276	−0.141882
C	0.280520	−1.542743	0.134630
C	−0.961476	−0.937559	−0.437222
C	−1.359400	0.352996	−0.273433
C	−0.516098	1.359070	0.502583
C	3.079127	0.898015	−0.924413
C	−1.766197	−1.913413	−1.272483
C	−2.646460	0.933075	−0.802947
H	1.277935	−1.982802	2.057186
H	0.270299	−0.353032	3.417891
H	−0.820639	1.639411	2.690602
H	2.477042	−1.419062	0.013384
H	1.033734	2.558094	−0.523533
H	0.366936	−2.604567	−0.095307
H	−1.016643	2.337069	0.482312
H	3.904565	0.717868	−0.227663
H	3.146454	1.939101	−1.257430
H	3.219188	0.258487	−1.802287
H	−2.435138	−2.494899	−0.630362
H	−2.354794	−1.425438	−2.053633
H	−1.105550	−2.611549	−1.799251
H	−2.436603	1.625826	−1.624127
H	−3.354952	0.182390	−1.159282
H	−3.164904	1.484445	−0.010334

Me_3BV (000 010 100 1)

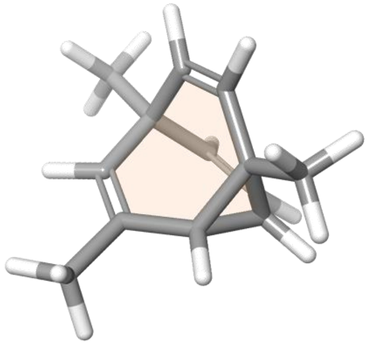

Table S40. Coordinates for the optimised geometry of **Me_3BV (000 010 100 1).**

Atom	Coordinates / Å		
	x	y	z
C	−0.633454	−0.945759	−1.383887
C	0.633645	−0.443710	−1.941524
C	1.585988	0.259847	−1.304973
C	−1.427979	−0.176168	−0.353573
C	−0.995365	1.144669	0.172937
C	0.279653	1.529804	0.398371
C	−0.758002	−1.489033	0.035209
C	0.420837	−1.495471	0.945734
C	1.413748	−0.591690	1.026892
C	1.497331	0.651411	0.159610
C	−2.117504	2.111809	0.464597
C	−1.676069	−2.681391	0.218802
C	2.766798	1.440181	0.540496
H	−1.227807	−1.448841	−2.144431
H	0.804337	−0.672358	−2.991818
H	2.468374	0.560446	−1.862942
H	−2.507840	−0.222810	−0.491762
H	0.468105	2.528187	0.785088
H	0.483037	−2.341037	1.629618
H	2.202147	−0.756805	1.756239
H	−2.798597	1.686200	1.209149
H	−1.756418	3.069351	0.854425
H	−2.685454	2.318188	−0.448741
H	−2.531368	−2.652178	−0.465457
H	−2.069789	−2.707058	1.240771
H	−1.133541	−3.614464	0.031337
H	3.674062	0.842160	0.386796
H	2.750947	1.744721	1.594714
H	2.870177	2.351800	−0.061677

Me$_3$BV (000 010 101 0)

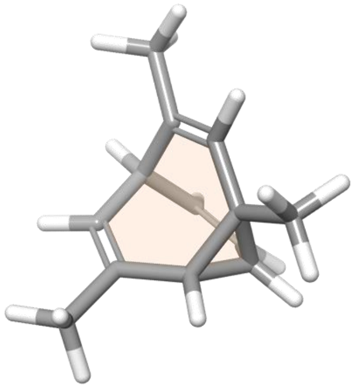

Table S41. Coordinates for the optimised geometry of **Me$_3$BV (000 010 101 0).**

Atom	Coordinates / Å		
	x	y	z
C	0.387476	1.227810	−1.271711
C	−0.078925	0.200006	−2.218797
C	−0.478704	−1.048733	−1.926113
C	1.205465	0.894005	−0.043488
C	1.607162	−0.494124	0.305727
C	0.864644	−1.601914	0.098307
C	−0.220001	1.412284	0.115159
C	−1.310471	0.510245	0.593408
C	−1.482768	−0.804885	0.343019
C	−0.511630	−1.587426	−0.518901
C	2.967853	−0.627095	0.943911
C	−0.360941	2.842830	0.598623
C	−2.642979	−1.575080	0.902987
H	0.684249	2.137181	−1.791055
H	−0.095461	0.497355	−3.265346
H	−0.802492	−1.711072	−2.722493
H	1.994485	1.611511	0.179934
H	1.243761	−2.576974	0.389988
H	−2.055893	0.992861	1.224485
H	−0.848847	−2.630875	−0.581259
H	3.216695	−1.665323	1.187749
H	3.007287	−0.049065	1.873239
H	3.741536	−0.250192	0.266681
H	−1.306981	3.272484	0.251490
H	−0.342490	2.879971	1.693323
H	0.448485	3.483599	0.231413
H	−3.245423	−1.996342	0.091653
H	−2.284748	−2.395048	1.533854
H	−3.300341	−0.947993	1.514213

Me_3BV (000 010 110 0)

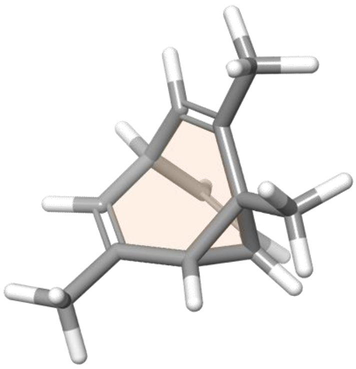

Table S42. Coordinates for the optimised geometry of **Me_3BV (000 010 110 0).**

Atom	Coordinates / Å x	y	z
C	0.011345	−0.418306	1.588999
C	0.374610	0.926341	2.066916
C	0.715190	1.986813	1.317098
C	0.648254	−1.054373	0.374043
C	1.693841	−0.380033	−0.438445
C	1.761836	0.944117	−0.686602
C	−0.832197	−0.673905	0.339562
C	−1.304494	0.488381	−0.514091
C	−0.616097	1.630150	−0.742322
C	0.747254	1.934782	−0.184636
C	2.752370	−1.291874	−1.008360
C	−1.792758	−1.850190	0.452181
C	−2.663618	0.367101	−1.171072
H	−0.156704	−1.094000	2.426114
H	0.369939	1.059424	3.146864
H	0.968069	2.927904	1.794406
H	0.855984	−2.118681	0.484197
H	2.568543	1.349029	−1.290352
H	−1.041966	2.407402	−1.371030
H	1.043036	2.928310	−0.546204
H	3.283145	−1.806664	−0.200549
H	2.292627	−2.044498	−1.657618
H	3.496108	−0.750515	−1.602586
H	−1.388041	−2.648923	1.084921
H	−2.740899	−1.537878	0.901812
H	−1.986859	−2.290629	−0.531189
H	−3.448170	0.254694	−0.416175
H	−2.689325	−0.493032	−1.847515
H	−2.921024	1.249051	−1.768366

Me_3BV (000 011 001 0)

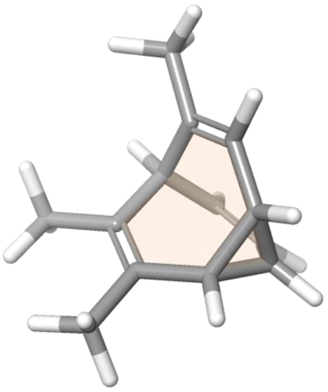

Table S43. Coordinates for the optimised geometry of **Me_3BV (000 011 001 0).**

	Coordinates / Å		
**Atom**	**x**	**y**	**z**
C	−1.080534	−1.974620	−0.507281
C	−0.299342	−1.651149	−1.709759
C	0.618610	−0.678504	−1.829471
C	−1.583249	−0.907325	0.438555
C	−1.347763	0.553084	0.221232
C	−0.203059	1.106099	−0.263494
C	−0.503266	−1.864688	0.887890
C	0.880196	−1.432764	1.148364
C	1.585435	−0.500302	0.477560
C	0.986663	0.251431	−0.697794
C	−2.547964	1.406795	0.579543
C	0.042132	2.586947	−0.410062
C	2.994441	−0.145543	0.851331
H	−1.755867	−2.811790	−0.670390
H	−0.494991	−2.268683	−2.583749
H	1.130095	−0.536894	−2.776233
H	−2.561159	−1.110300	0.874504
H	−0.824342	−2.635455	1.585898
H	1.364568	−1.935877	1.983151
H	1.761848	0.916253	−1.104650
H	−2.598736	2.336189	0.006338
H	−2.534491	1.644588	1.647810
H	−3.482046	0.877536	0.358902
H	1.013387	2.852157	0.022871
H	−0.694709	3.208935	0.102491
H	0.049633	2.863614	−1.469142
H	3.352727	−0.723810	1.709368
H	3.061506	0.915290	1.113665
H	3.670275	−0.341215	0.012551

Me_3BV (000 011 010 0)

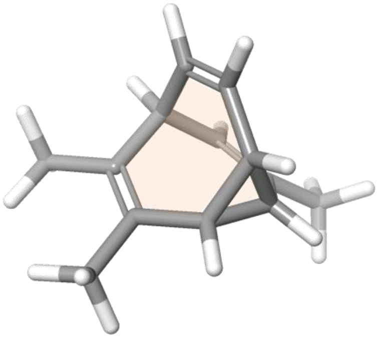

Table S44. Coordinates for the optimised geometry of **Me_3BV (000 011 010 0)**

	Coordinates / Å		
**Atom**	**x**	**y**	**z**
C	1.077945	−0.378306	1.723565
C	0.501495	0.913465	2.123234
C	−0.215354	1.743617	1.349172
C	0.376918	−1.320053	0.769589
C	−0.953123	−1.031290	0.150376
C	−1.398794	0.197195	−0.227651
C	1.654055	−0.630154	0.345536
C	1.685137	0.405320	−0.715395
C	0.725990	1.330132	−0.924948
C	−0.527886	1.441764	−0.093085
C	−1.794169	−2.275468	−0.055028
C	−2.767346	0.482593	−0.792467
C	2.900364	0.380187	−1.607905
H	1.609750	−0.855768	2.544210
H	0.677697	1.213649	3.153956
H	−0.595123	2.672860	1.761275
H	0.493678	−2.372716	1.026645
H	2.535306	−1.270135	0.329615
H	0.827573	2.050036	−1.731771
H	−1.079518	2.308664	−0.482475
H	−2.353055	−2.505963	0.857357
H	−2.493586	−2.187133	−0.890357
H	−1.161331	−3.137506	−0.295320
H	−2.690382	0.733839	−1.855121
H	−3.220843	1.331948	−0.269050
H	−3.470347	−0.346133	−0.685393
H	3.808513	0.528628	−1.014198
H	2.973526	−0.585094	−2.119919
H	2.872908	1.161821	−2.374448

Me_3BV (000 011 100 0)

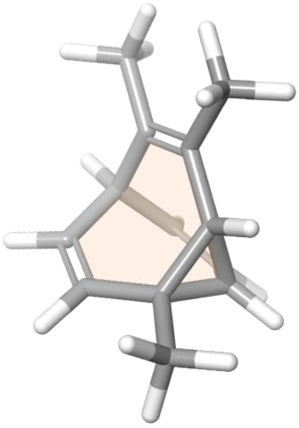

Table S45. Coordinates for the optimised geometry of **Me_3BV (000 011 100 0)**

	Coordinates / Å		
**Atom**	**x**	**y**	**z**
C	1.442882	−0.916452	−0.713809
C	0.715363	−2.130025	−0.310213
C	−0.354522	−2.197251	0.497789
C	0.746430	0.402280	−0.957655
C	−0.730130	0.601377	−0.810281
C	−1.530260	−0.021615	0.096673
C	1.698024	0.260256	0.223843
C	1.169702	0.247057	1.615139
C	0.013244	−0.283147	2.046665
C	−0.966399	−0.980267	1.138993
C	−1.288428	1.619623	−1.785236
C	−3.026988	0.140792	0.181598
C	3.042723	0.946791	0.088925
H	2.243174	−1.152539	−1.413035
H	1.089634	−3.064346	−0.723544
H	−0.808342	−3.159217	0.712955
H	1.141398	0.947579	−1.815121
H	1.794573	0.726979	2.366826
H	−0.247771	−0.213294	3.098027
H	−1.783669	−1.337435	1.780504
H	−2.182208	2.125901	−1.411487
H	−0.560120	2.416742	−1.973952
H	−1.522177	1.137383	−2.739554
H	−3.509100	−0.841401	0.244204
H	−3.292997	0.712958	1.076089
H	−3.466592	0.635773	−0.686928
H	2.972102	1.992369	0.408127
H	3.792957	0.443696	0.708714
H	3.407495	0.939431	−0.944256

Me_3BV (000 100 001 1)

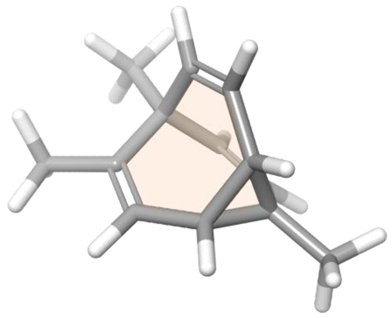

Table S46. Coordinates for the optimised geometry of **Me_3BV (000 100 001 1)**

	Coordinates / Å		
**Atom**	**x**	**y**	**z**
C	1.458608	0.940753	−1.037770
C	0.344913	0.650733	−1.953782
C	−0.801105	0.007589	−1.671983
C	1.922502	−0.030438	0.039511
C	1.224352	−1.328868	0.236921
C	−0.086548	−1.597316	0.103208
C	1.228787	1.270593	0.417819
C	−0.118975	1.319742	1.017196
C	−1.198459	0.551079	0.746002
C	−1.141659	−0.574727	−0.303909
C	3.416207	−0.093496	0.287901
C	−2.484180	0.795682	1.490806
C	−2.479452	−1.337940	−0.456693
H	2.236631	1.522367	−1.528494
H	0.470767	1.016167	−2.971252
H	−1.531597	−0.106402	−2.468614
H	1.857736	−2.163262	0.535657
H	−0.419466	−2.612307	0.304915
H	1.867511	2.055237	0.819616
H	−0.230389	2.093915	1.775098
H	3.620130	−0.409106	1.316941
H	3.900254	0.877870	0.136986
H	3.887646	−0.808560	−0.395118
H	−2.776418	−0.092037	2.060051
H	−2.397451	1.619898	2.207522
H	−3.288359	1.060090	0.797336
H	−2.402654	−2.133292	−1.210134
H	−3.293374	−0.675953	−0.776279
H	−2.785958	−1.818011	0.480539

Me_3BV (000 100 010 1)

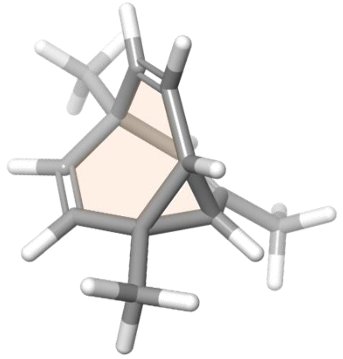

Table S47. Coordinates for the optimised geometry of **Me_3BV (000 100 010 1).**

Atom	Coordinates / Å		
	x	y	z
C	−1.227199	0.652353	−1.131058
C	−0.266324	0.052821	−2.072244
C	0.826968	−0.670864	−1.775360
C	−1.663330	−0.033522	0.158849
C	−1.087923	−1.352789	0.542521
C	0.161374	−1.806687	0.336338
C	−0.806346	1.226360	0.202541
C	0.606111	1.233407	0.665027
C	1.523761	0.271230	0.426371
C	1.248464	−1.003605	−0.354864
C	−3.119220	0.132357	0.547615
C	1.006567	2.447698	1.467993
C	2.537336	−1.849257	−0.404489
H	−1.993926	1.218633	−1.656634
H	−0.475146	0.226483	−3.126172
H	1.439521	−1.043030	−2.591794
H	−1.768524	−2.032786	1.053337
H	0.410897	−2.804904	0.685743
H	−1.328848	2.140307	0.483725
H	2.530207	0.393848	0.818627
H	−3.733851	−0.635823	0.065710
H	−3.516447	1.109283	0.250249
H	−3.237792	0.042640	1.632966
H	2.051808	2.410955	1.792578
H	0.383422	2.529379	2.364881
H	0.876414	3.355896	0.870009
H	2.881978	−2.118138	0.602138
H	2.384654	−2.784402	−0.958178
H	3.355393	−1.307843	−0.896427

Me_3BV (000 100 011 0)

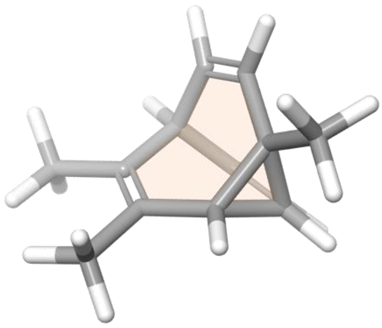

Table S48. Coordinates for the optimised geometry of **Me_3BV (000 100 011 0).**

	Coordinates / Å		
**Atom**	**x**	**y**	**z**
C	1.306395	−0.250054	1.289237
C	0.459425	−1.358338	1.757512
C	−0.584899	−1.902656	1.113194
C	1.722833	−0.069398	−0.167773
C	1.241018	−1.011362	−1.214464
C	0.046323	−1.622352	−1.278676
C	0.756265	0.908071	0.489446
C	−0.682818	1.037129	0.097056
C	−1.519011	0.008789	−0.209855
C	−1.034353	−1.435925	−0.245753
C	3.131607	0.437183	−0.405421
C	−1.153052	2.477661	0.044087
C	−2.984266	0.155956	−0.534124
H	2.052129	0.017030	2.036007
H	0.712177	−1.765666	2.734280
H	−1.137594	−2.715912	1.572290
H	1.939828	−1.224612	−2.021936
H	−0.171469	−2.285054	−2.110398
H	1.182886	1.870466	0.772977
H	−1.865938	−2.088304	−0.545593
H	3.839448	−0.398532	−0.430653
H	3.189991	0.971554	−1.359962
H	3.460088	1.126008	0.380836
H	−1.470154	2.806328	1.038743
H	−0.345587	3.137745	−0.293162
H	−1.971425	2.635825	−0.663088
H	−3.155167	−0.036801	−1.598026
H	−3.572209	−0.564437	0.045886
H	−3.392471	1.139656	−0.292668

Me$_3$BV (000 100 100 1)

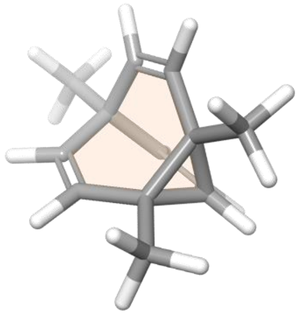

Table S49. Coordinates for the optimised geometry of **Me$_3$BV (000 100 100 1).**

Atom	Coordinates / Å		
	x	y	z
C	−0.900221	−0.403725	1.197529
C	0.405409	−0.565921	1.862111
C	1.627122	−0.354315	1.343456
C	−1.136588	−0.761410	−0.263821
C	−0.005976	−1.234916	−1.120402
C	1.294803	−0.898002	−1.062374
C	−1.192620	0.715193	0.206633
C	−0.116320	1.672976	−0.193933
C	1.205517	1.454939	−0.312715
C	1.863510	0.108710	−0.081674
C	−2.438466	−1.482818	−0.574839
C	−2.547556	1.392034	0.341105
C	3.380771	0.242090	−0.319603
H	−1.707649	−0.653101	1.884074
H	0.360311	−0.897647	2.897919
H	2.494009	−0.523051	1.976307
H	−0.274805	−1.956126	−1.891694
H	1.977117	−1.354767	−1.774224
H	−0.450982	2.686654	−0.412484
H	1.839013	2.284643	−0.614691
H	−3.210634	−1.317213	0.182247
H	−2.275995	−2.565862	−0.622227
H	−2.832138	−1.151305	−1.541718
H	−2.938583	1.653924	−0.647958
H	−3.289717	0.766815	0.846226
H	−2.461070	2.311449	0.931717
H	3.897238	−0.714573	−0.170038
H	3.601157	0.576038	−1.341504
H	3.833342	0.969288	0.366454

Me_3BV (000 100 101 0)

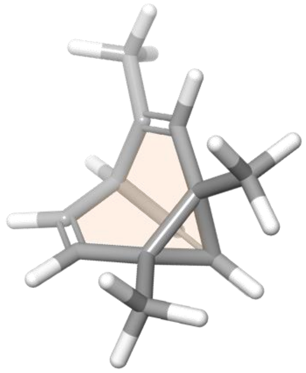

Table S50. Coordinates for the optimised geometry of **Me_3BV (000 100 101 0).**

Atom	Coordinates / Å		
	x	y	z
C	−1.176109	0.476915	0.937458
C	−0.272843	0.298757	2.089474
C	0.940786	−0.276623	2.093954
C	−1.333878	−0.562319	−0.166384
C	−0.504292	−1.807035	−0.163971
C	0.751657	−1.972237	0.284846
C	−0.675389	0.810851	−0.462211
C	0.794382	0.901601	−0.746556
C	1.813672	0.213944	−0.189992
C	1.578718	−0.853361	0.858153
C	−2.753178	−0.815992	−0.649284
C	−1.473992	1.856965	−1.225024
C	3.241632	0.447111	−0.590218
H	−2.089563	0.981327	1.248309
H	−0.642713	0.684504	3.037523
H	1.503341	−0.337239	3.020125
H	−0.984556	−2.693713	−0.576134
H	1.219947	−2.949161	0.215886
H	1.061785	1.635715	−1.506214
H	2.543445	−1.282276	1.160274
H	−3.179030	−1.692636	−0.147518
H	−3.430178	0.018622	−0.444510
H	−2.757242	−1.001674	−1.728745
H	−2.503100	1.954591	−0.866959
H	−1.012429	2.845560	−1.118904
H	−1.506104	1.604438	−2.290352
H	3.677269	−0.472086	−0.995173
H	3.831717	0.760446	0.277139
H	3.336247	1.225004	−1.354994

Me_3BV (000 100 110 0)

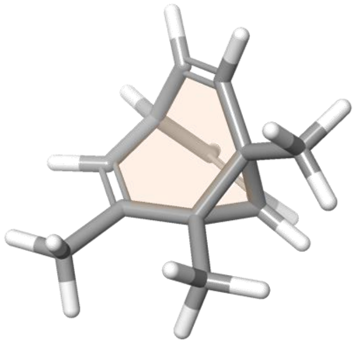

Table S51. Coordinates for the optimised geometry of **Me_3BV (000 100 110 0).**

	Coordinates / Å		
**Atom**	**x**	**y**	**z**
C	0.817761	−0.008212	−1.203452
C	0.378934	1.253895	−1.823668
C	−0.323731	2.243094	−1.250534
C	1.271575	−0.125506	0.245879
C	1.230332	1.067063	1.148611
C	0.370507	2.099272	1.130951
C	−0.043581	−0.811874	−0.232408
C	−1.387416	−0.274190	0.249956
C	−1.718254	1.028426	0.402951
C	−0.784888	2.183492	0.175907
C	2.498983	−0.988685	0.499845
C	−0.016854	−2.318093	−0.484246
C	−2.452748	−1.286445	0.622910
H	1.392332	−0.590448	−1.922726
H	0.664994	1.387947	−2.865163
H	−0.581725	3.125877	−1.826478
H	2.007566	1.107860	1.910904
H	0.479156	2.900826	1.854748
H	−2.708195	1.301190	0.758850
H	−1.333200	3.109741	0.391055
H	2.375974	−1.553758	1.430015
H	3.396498	−0.365910	0.592043
H	2.699646	−1.698333	−0.307919
H	−0.842665	−2.619233	−1.137372
H	0.894925	−2.650556	−0.989660
H	−0.086539	−2.867761	0.460039
H	−3.345091	−0.817557	1.052980
H	−2.073242	−1.986162	1.374374
H	−2.781056	−1.845958	−0.258392

Me_3BV (000 101 001 0)

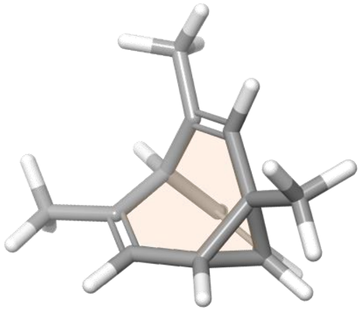

Table S52. Coordinates for the optimised geometry of **Me_3BV (000 101 001 0).**

	Coordinates / Å		
**Atom**	**x**	**y**	**z**
C	1.411826	−0.599992	−1.334245
C	0.417308	0.079498	−2.182784
C	−0.771952	0.573395	−1.799579
C	1.699958	−0.177226	0.102047
C	0.939088	0.942925	0.732514
C	−0.355918	1.281877	0.558100
C	1.013961	−1.506177	−0.190783
C	−0.394806	−1.775594	0.158417
C	−1.440220	−0.925660	0.091366
C	−1.271437	0.510010	−0.376177
C	3.148165	−0.254117	0.544357
C	−0.975215	2.443325	1.280119
C	−2.828311	−1.339673	0.482891
H	2.266483	−0.937644	−1.917345
H	0.685797	0.181770	−3.232267
H	−1.415564	1.051425	−2.531185
H	1.519219	1.554258	1.422843
H	1.626263	−2.398597	−0.074423
H	−0.586615	−2.788641	0.507605
H	−2.258275	0.993329	−0.373163
H	3.676346	0.670367	0.286626
H	3.680943	−1.084381	0.067472
H	3.209866	−0.399677	1.628372
H	−1.347729	3.181210	0.562198
H	−0.262044	2.950377	1.938322
H	−1.812978	2.102706	1.897278
H	−2.871004	−2.382308	0.814644
H	−3.193220	−0.714015	1.303926
H	−3.509935	−1.232769	−0.367147

Me_3BV (000 101 010 0)

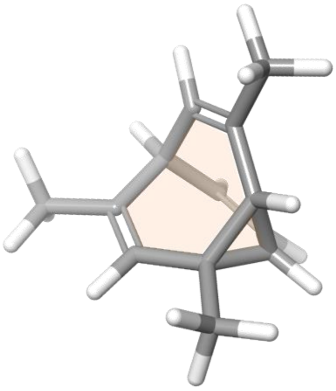

Table S53. Coordinates for the optimised geometry of **Me_3BV (000 101 010 0).**

Atom	Coordinates / Å		
	x	y	z
C	1.333432	−0.055614	−1.222199
C	0.249547	−0.251122	−2.200899
C	−1.071432	−0.252936	−1.956264
C	1.279970	−0.616228	0.195307
C	0.080257	−1.369028	0.670176
C	−1.217260	−1.162951	0.360451
C	1.209831	0.887962	−0.045996
C	−0.010761	1.695223	0.216107
C	−1.279451	1.298500	−0.017541
C	−1.647450	−0.052705	−0.577993
C	2.587059	−1.142800	0.755936
C	−2.314558	−2.016516	0.926327
C	0.238721	3.067238	0.792104
H	2.308347	−0.065103	−1.706141
H	0.567232	−0.409066	−3.229531
H	−1.769887	−0.410951	−2.771705
H	0.298840	−2.188385	1.354067
H	2.118837	1.443860	0.181972
H	−2.110036	1.960586	0.208998
H	−2.740887	−0.073477	−0.679736
H	2.587640	−1.078103	1.849508
H	3.450526	−0.576267	0.389791
H	2.732109	−2.190033	0.468954
H	−2.864114	−2.511805	0.119271
H	−3.015653	−1.401705	1.500039
H	−1.931373	−2.794993	1.594238
H	0.851079	3.660263	0.104630
H	0.768164	2.985336	1.747251
H	−0.688730	3.620820	0.972878

Me_3BV (000 101 100 0)

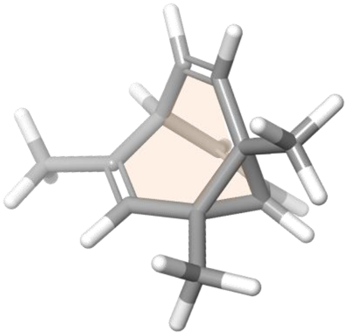

Table S54. Coordinates for the optimised geometry of **Me_3BV (000 101 100 0).**

	Coordinates / Å		
**Atom**	**x**	**y**	**z**
C	−1.047832	−0.244686	1.153984
C	−0.023434	0.195582	2.119150
C	1.167078	0.755035	1.847728
C	−0.702287	−0.895436	−0.179596
C	0.727060	−1.051271	−0.606210
C	1.784932	−0.254050	−0.347495
C	−1.358542	0.509532	−0.133642
C	−0.567319	1.719868	−0.516508
C	0.727011	1.983667	−0.269004
C	1.643622	1.027962	0.445870
C	−1.555191	−2.089466	−0.580656
C	3.163231	−0.575306	−0.848200
C	−2.830060	0.645360	−0.491612
H	−1.905968	−0.662087	1.678234
H	−0.271825	0.041227	3.167424
H	1.830337	1.028803	2.662021
H	0.924877	−1.942174	−1.201751
H	−1.114931	2.486871	−1.062912
H	1.158337	2.917311	−0.616416
H	2.624965	1.514423	0.525036
H	−2.533503	−2.100139	−0.091172
H	−1.058554	−3.026741	−0.304088
H	−1.715930	−2.091554	−1.664115
H	3.856950	−0.678970	−0.007550
H	3.190025	−1.509793	−1.418256
H	3.525141	0.224107	−1.502914
H	−3.456829	−0.118089	−0.021349
H	−3.218562	1.615072	−0.159423
H	−2.962797	0.574942	−1.576579

Me_3BV (000 110 001 0)

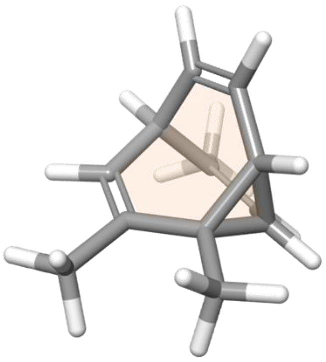

Table S55. Coordinates for the optimised geometry of **Me_3BV (000 110 001 0).**

	Coordinates / Å		
**Atom**	**x**	**y**	**z**
C	−0.597030	1.709096	−0.208836
C	0.405697	1.783960	−1.284878
C	1.353779	0.877437	−1.572460
C	−1.311134	0.418924	0.193569
C	−0.968705	−0.889827	−0.493284
C	0.258717	−1.253943	−0.930048
C	−0.301065	1.077059	1.131877
C	1.010301	0.488544	1.464675
C	1.852424	−0.169875	0.643122
C	1.503040	−0.412692	−0.810699
C	−2.767392	0.598864	0.599327
C	−2.093189	−1.880911	−0.709610
C	3.169583	−0.710268	1.115603
H	−1.192629	2.619492	−0.167809
H	0.370585	2.681398	−1.899207
H	2.041973	1.058208	−2.391818
H	0.401559	−2.219170	−1.408467
H	−0.717313	1.603319	1.989456
H	1.315175	0.622791	2.501049
H	2.319897	−0.973519	−1.284785
H	−3.073149	−0.176404	1.309370
H	−3.423328	0.565327	−0.276632
H	−2.938342	1.564911	1.088885
H	−2.876080	−1.444382	−1.337770
H	−2.528116	−2.185434	0.247548
H	−1.756039	−2.795105	−1.211021
H	3.347148	−0.498661	2.175172
H	3.203926	−1.796453	0.982755
H	3.989705	−0.262686	0.544917

Me_3BV (000 110 010 0)

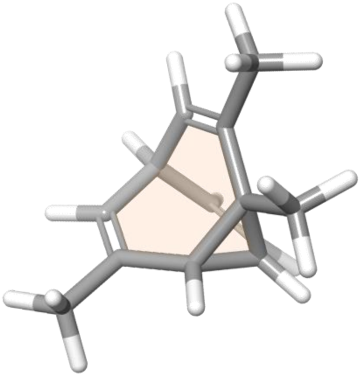

Table S56. Coordinates for the optimised geometry of **Me_3BV (000 110 010 0).**

Atom	Coordinates / Å		
	x	y	z
C	−0.250843	−0.197686	−1.611839
C	−0.329475	1.244033	−1.901158
C	−0.343386	2.248404	−1.010165
C	0.617087	−0.792855	−0.502578
C	1.423174	0.115704	0.407061
C	1.045165	1.343018	0.831829
C	−0.908316	−0.826705	−0.404190
C	−1.689690	−0.046681	0.590365
C	−1.422585	1.216285	0.981709
C	−0.252738	2.012403	0.471397
C	1.260747	−2.128933	−0.848414
C	2.768876	−0.388292	0.885208
C	−2.877516	−0.765569	1.181137
H	−0.321408	−0.780592	−2.528791
H	−0.390003	1.510296	−2.954468
H	−0.409832	3.275471	−1.353881
H	1.695882	1.918686	1.484464
H	−1.369021	−1.794921	−0.600011
H	−2.054580	1.709575	1.714164
H	−0.273614	2.991967	0.966711
H	1.432938	−2.724061	0.054278
H	0.625104	−2.729550	−1.509737
H	2.211728	−1.980335	−1.370175
H	3.278298	0.330079	1.537348
H	2.652193	−1.312140	1.460492
H	3.434602	−0.574555	0.036597
H	−2.551260	−1.678605	1.690317
H	−3.417383	−0.153198	1.911115
H	−3.584143	−1.041243	0.391215

Me$_3$BV (000 110 100 0)

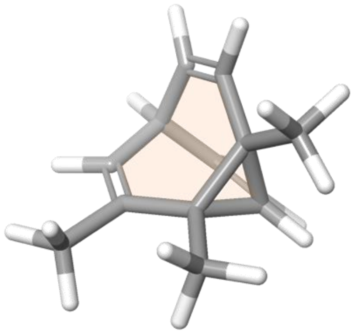

Table S57. Coordinates for the optimised geometry of **Me$_3$BV (000 110 100 0).**

	Coordinates / Å		
**Atom**	**x**	**y**	**z**
C	0.342934	−0.525560	1.312738
C	−0.901053	−0.228746	2.043867
C	−2.087760	0.122645	1.525229
C	0.802115	0.254411	0.083304
C	−0.085281	1.329137	−0.537310
C	−1.437740	1.350778	−0.529836
C	0.384063	−1.242852	−0.030417
C	−0.886828	−1.634766	−0.716047
C	−2.079254	−1.016632	−0.682960
C	−2.310269	0.273604	0.049203
C	2.293149	0.585544	0.083310
C	0.601197	2.472853	−1.257970
C	1.469158	−2.296607	−0.198579
H	1.125045	−0.805470	2.017322
H	−0.837662	−0.308765	3.127422
H	−2.929275	0.313543	2.183248
H	−1.972450	2.167197	−1.007949
H	−0.840113	−2.547713	−1.308664
H	−2.916307	−1.435249	−1.232757
H	−3.357025	0.565867	−0.104936
H	2.692703	0.562920	−0.935764
H	2.885715	−0.112435	0.682483
H	2.473287	1.574267	0.518283
H	1.173152	3.086162	−0.554939
H	−0.109422	3.140712	−1.758049
H	1.272584	2.091706	−2.034046
H	2.298960	−2.174517	0.503467
H	1.063371	−3.299892	−0.023540
H	1.873003	−2.262146	−1.216113

Me_3BV (001 001 001 0)

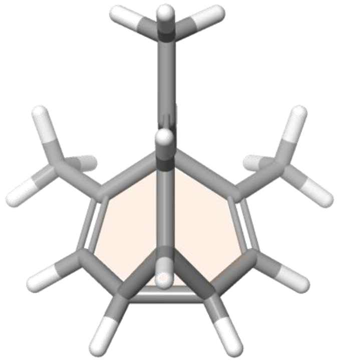

Table S58. Coordinates for the optimised geometry of **Me_3BV (001 001 001 0).**

Atom	Coordinates / Å x	y	z
C	1.845625	−1.409149	−0.263178
C	2.049900	0.018704	0.043118
C	1.104023	0.963334	0.225107
C	0.728184	−1.893358	−1.160271
C	−0.234281	−0.971076	−1.790644
C	−0.753968	0.158230	−1.266504
C	0.699924	−2.206691	0.319233
C	−0.292048	−1.611566	1.233634
C	−0.800957	−0.362755	1.193493
C	−0.381333	0.641731	0.128626
C	1.455973	2.389189	0.533794
C	−1.731109	1.008163	−2.024811
C	−1.811713	0.114496	2.194902
H	2.787791	−1.944832	−0.358629
H	3.092631	0.319408	0.126716
H	0.978951	−2.728638	−1.810785
H	−0.545224	−1.256946	−2.793784
H	0.933207	−3.235841	0.584138
H	−0.637227	−2.277011	2.022769
H	−0.934786	1.573113	0.315305
H	1.061738	3.053493	−0.242070
H	1.028509	2.685132	1.497317
H	2.538321	2.545985	0.587673
H	−1.951574	0.600425	−3.016834
H	−2.676286	1.079763	−1.476910
H	−1.329345	2.017394	−2.161633
H	−1.423047	0.978562	2.743550
H	−2.736762	0.409290	1.688875
H	−2.065120	−0.658550	2.927808

Me_3BV (001 001 010 0)

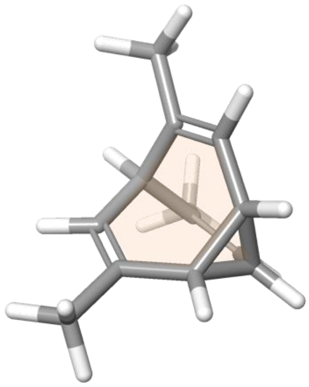

Table S59. Coordinates for the optimised geometry of **Me_3BV (001 001 010 0).**

	Coordinates / Å		
**Atom**	**x**	**y**	**z**
C	0.983444	−0.791337	−1.611009
C	−0.049304	−1.662970	−1.020755
C	−0.876081	−1.376745	0.006065
C	0.800159	0.704539	−1.747025
C	−0.423875	1.394089	−1.298720
C	−1.180203	1.105375	−0.219621
C	1.827913	0.152786	−0.782845
C	1.684910	0.269220	0.690396
C	0.521492	0.188449	1.369859
C	−0.824627	−0.035621	0.719491
C	−1.894330	−2.359024	0.505470
C	−2.414676	1.887870	0.119330
C	2.970286	0.495449	1.446308
H	1.499697	−1.275817	−2.437307
H	−0.137150	−2.642239	−1.487649
H	1.203232	1.143758	−2.657312
H	−0.733489	2.224791	−1.930186
H	2.861720	0.249558	−1.111644
H	0.513521	0.285830	2.451585
H	−1.561256	−0.051734	1.534931
H	−1.706802	−2.598264	1.557228
H	−2.901480	−1.938864	0.417115
H	−1.874679	−3.297645	−0.058072
H	−2.608103	2.688348	−0.602333
H	−2.312839	2.348037	1.107496
H	−3.289736	1.229944	0.128995
H	2.812138	0.574354	2.527071
H	3.447403	1.422619	1.111664
H	3.662718	−0.334756	1.271476

Me_3BV (001 001 100 0)

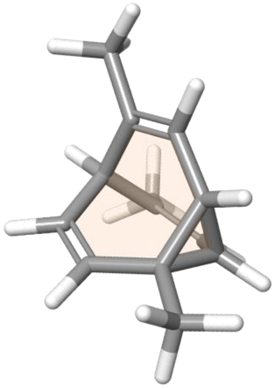

Table S60. Coordinates for the optimised geometry of **Me_3BV (001 001 100 0).**

	Coordinates / Å		
**Atom**	**x**	**y**	**z**
C	−1.097997	−0.932190	−1.039753
C	0.296701	−1.416289	−1.037949
C	1.334453	−0.945026	−0.316203
C	−1.447058	0.530365	−0.883402
C	−0.417273	1.575245	−0.718146
C	0.754845	1.483520	−0.056585
C	−1.817596	−0.457060	0.217613
C	−1.105197	−0.426909	1.526033
C	0.189027	−0.142158	1.751801
C	1.168673	0.208517	0.658586
C	2.711493	−1.530651	−0.426903
C	1.718122	2.631551	0.018044
C	−3.284994	−0.819180	0.328979
H	−1.703324	−1.477303	−1.761757
H	0.485665	−2.248260	−1.714045
H	−2.265866	0.879733	−1.509783
H	−0.652454	2.520429	−1.204259
H	−1.708903	−0.664499	2.400702
H	0.569937	−0.159907	2.768210
H	2.133964	0.386079	1.152677
H	3.037247	−1.920454	0.542913
H	2.754351	−2.353639	−1.147893
H	3.423570	−0.765481	−0.752728
H	2.680569	2.347678	−0.419922
H	1.354287	3.512606	−0.520788
H	1.881303	2.922918	1.060677
H	−3.781003	−0.833125	−0.647942
H	−3.400968	−1.813474	0.773954
H	−3.811573	−0.093036	0.957869

Me_3BV (001 010 010 0)

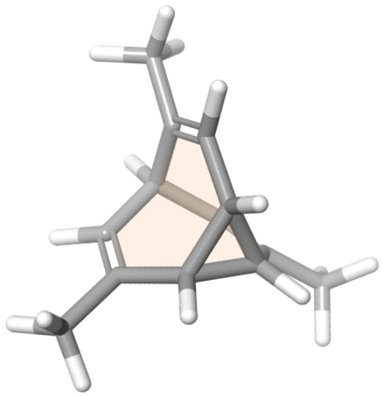

Table S61. Coordinates for the optimised geometry of **Me_3BV (001 010 010 0).**

	Coordinates / Å		
**Atom**	**x**	**y**	**z**
C	0.191368	−0.188613	1.753246
C	1.529301	−0.071483	1.144258
C	1.824647	0.085196	−0.162510
C	−0.952397	−0.917978	1.081113
C	−0.812692	−1.576853	−0.242263
C	−0.068088	−1.114602	−1.268639
C	−1.007670	0.585927	1.248936
C	−0.926817	1.528323	0.104247
C	−0.158986	1.358598	−0.992652
C	0.734055	0.162493	−1.214378
C	3.237875	0.191978	−0.653974
C	−1.579449	−2.865428	−0.404614
C	−1.786273	2.761955	0.223353
H	0.248340	−0.307068	2.833518
H	2.355593	−0.119986	1.851042
H	−1.598340	−1.476033	1.757738
H	−0.020835	−1.666673	−2.202675
H	−1.686881	0.932996	2.026565
H	−0.159444	2.104683	−1.781825
H	1.201197	0.289701	−2.200467
H	3.456387	−0.616467	−1.359154
H	3.391485	1.149493	−1.162085
H	3.965231	0.127652	0.161997
H	−1.248606	−3.598473	0.338659
H	−2.650968	−2.687715	−0.264840
H	−1.443050	−3.311785	−1.395296
H	−2.840920	2.480620	0.311901
H	−1.690613	3.424143	−0.643623
H	−1.503449	3.335397	1.112422

Me_3BV (001 010 100 0)

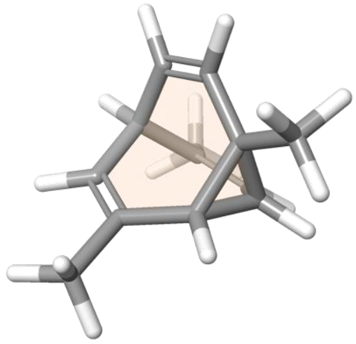

Table S62. Coordinates for the optimised geometry of **Me_3BV (001 010 100 0).**

Atom	Coordinates / Å x	y	z
C	−0.410280	0.934131	1.183543
C	0.848615	0.292055	1.610256
C	1.644194	−0.525386	0.890380
C	−1.402399	0.256380	0.265127
C	−1.187822	−1.111297	−0.277113
C	0.003618	−1.637481	−0.631121
C	−0.633751	1.480862	−0.222686
C	0.428801	1.347057	−1.259185
C	1.293534	0.331280	−1.424633
C	1.311238	−0.890585	−0.543289
C	2.901759	−1.115998	1.456261
C	−2.435270	−1.947875	−0.420105
C	−1.423240	2.771037	−0.323640
H	−0.852376	1.508065	1.996127
H	1.146132	0.517920	2.632723
H	−2.446636	0.429608	0.523651
H	0.061435	−2.652368	−1.013487
H	0.513495	2.174815	−1.961757
H	2.022190	0.373898	−2.228062
H	2.091867	−1.558124	−0.932366
H	3.074621	−0.805759	2.492050
H	3.768093	−0.801188	0.865520
H	2.847496	−2.209361	1.441010
H	−2.916274	−2.079356	0.554899
H	−2.227704	−2.943880	−0.825307
H	−3.144409	−1.456195	−1.094413
H	−2.193491	2.844570	0.452174
H	−1.924152	2.837670	−1.295608
H	−0.759282	3.635506	−0.214324

Me$_3$BV (001 100 010 0)

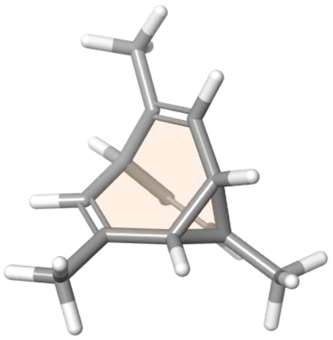

Table S63. Coordinates for the optimised geometry of **Me$_3$BV (001 100 010 0).**

	Coordinates / Å		
**Atom**	**x**	**y**	**z**
C	−0.372178	1.137189	−0.935377
C	1.044484	0.820631	−1.203745
C	1.839194	−0.040610	−0.535855
C	−0.951981	1.243058	0.471376
C	−0.091888	0.984097	1.660674
C	0.909788	0.094820	1.773687
C	−1.363441	0.101553	−0.453721
C	−0.988215	−1.317334	−0.215877
C	0.197831	−1.745685	0.265303
C	1.327439	−0.825647	0.656394
C	3.269979	−0.270489	−0.923827
C	−1.983668	2.331628	0.691991
C	−2.052395	−2.329217	−0.562358
H	−0.761693	1.841996	−1.667954
H	1.474092	1.356097	−2.048542
H	−0.300995	1.592651	2.539254
H	1.456333	0.019907	2.708529
H	−2.356045	0.188844	−0.894617
H	0.382231	−2.807497	0.399823
H	2.138963	−1.456286	1.043815
H	3.428988	−1.322933	−1.180144
H	3.565412	0.331235	−1.789643
H	3.934811	−0.008926	−0.094249
H	−1.493255	3.278215	0.944111
H	−2.657252	2.059470	1.511924
H	−2.598083	2.503678	−0.198780
H	−2.955306	−2.145300	0.029457
H	−2.311541	−2.257089	−1.623925
H	−1.731607	−3.358056	−0.367721

Me_3BV (001 100 100 0)

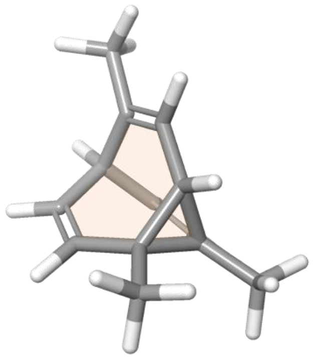

Table S64. Coordinates for the optimised geometry of **Me_3BV (001 100 100 0).**

	Coordinates / Å		
**Atom**	**x**	**y**	**z**
C	−0.358259	−0.485098	−1.046226
C	1.097950	−0.280308	−1.189980
C	1.961127	0.225432	−0.285677
C	−1.267788	0.485046	−0.302754
C	−0.693037	1.678828	0.392025
C	0.506208	1.801751	0.986229
C	−1.012349	−0.942191	0.251620
C	−0.189145	−1.136600	1.485602
C	0.912507	−0.468384	1.868001
C	1.499117	0.675204	1.085149
C	3.425237	0.373787	−0.578358
C	−2.604798	0.788436	−0.960208
C	−2.107871	−1.988106	0.118273
H	−0.768640	−0.913291	−1.959516
H	1.499543	−0.583463	−2.155498
H	−1.327189	2.564438	0.408589
H	0.782010	2.746745	1.443762
H	−0.522908	−1.929357	2.154081
H	1.405555	−0.737211	2.797008
H	2.362441	1.049595	1.651224
H	4.015722	−0.210467	0.134816
H	3.682958	0.028859	−1.585135
H	3.723125	1.424405	−0.500199
H	−3.382235	0.906327	−0.197705
H	−2.925943	0.008057	−1.656403
H	−2.546594	1.717459	−1.539132
H	−2.565935	−2.003451	−0.875082
H	−2.897154	−1.804027	0.855067
H	−1.703655	−2.992415	0.290308

Me_3BV (010 010 010 0)

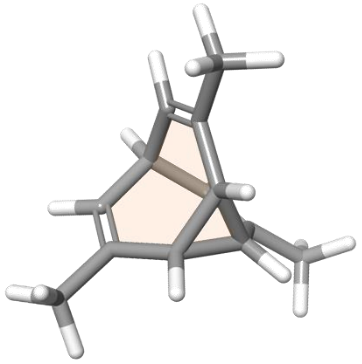

Table S65. Coordinates for the optimised geometry of **Me_3BV (010 010 010 0).**

**Atom**	**Coordinates / Å** x	y	z
C	−0.454083	−0.865670	1.064591
C	−0.739123	−1.638583	−0.170879
C	−0.479584	−1.221985	−1.427987
C	0.771248	0.010695	1.222289
C	1.790895	0.170904	0.154731
C	1.532525	0.217090	−1.169031
C	−0.604778	0.639289	1.146509
C	−1.050275	1.468802	−0.001735
C	−0.727041	1.249300	−1.293468
C	0.146580	0.109927	−1.749830
C	−1.369146	−2.991594	0.046035
C	3.215456	0.287346	0.636068
C	−1.932980	2.639248	0.352537
H	−0.748722	−1.401999	1.965865
H	−0.721799	−1.854532	−2.276779
H	1.212475	0.000662	2.218268
H	2.342419	0.337018	−1.882418
H	−0.989917	1.006754	2.096979
H	−1.098649	1.908959	−2.071922
H	0.238057	0.178528	−2.841842
H	−0.711915	−3.621359	0.654898
H	−1.558988	−3.520485	−0.893927
H	−2.326898	−2.883609	0.565960
H	3.322574	1.156926	1.293052
H	3.499440	−0.609375	1.196884
H	3.927501	0.403504	−0.187821
H	−1.406706	3.317367	1.032584
H	−2.844825	2.288813	0.847516
H	−2.233742	3.218058	−0.527129

Me$_3$BV (010 010 100 0)

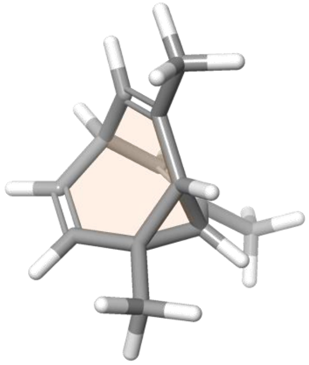

Table S66. Coordinates for the optimised geometry of **Me$_3$BV (010 010 100 0).**

	Coordinates / Å		
**Atom**	**x**	**y**	**z**
C	0.648989	−0.937184	−0.374519
C	1.700900	0.112422	−0.327820
C	1.546506	1.356278	0.172429
C	−0.811132	−0.627910	−0.622545
C	−1.319258	0.752135	−0.840845
C	−0.853594	1.864654	−0.235269
C	−0.370168	−1.153386	0.740961
C	−0.385303	−0.265063	1.937749
C	−0.115331	1.050666	1.989064
C	0.261459	1.857254	0.776685
C	3.036592	−0.291102	−0.901815
C	−2.449127	0.870853	−1.833658
C	−0.727967	−2.593914	1.051066
H	1.005622	−1.842400	−0.865392
H	2.374887	2.058427	0.161876
H	−1.320625	−1.349669	−1.260542
H	−1.287856	2.834250	−0.460304
H	−0.646730	−0.743112	2.880668
H	−0.166195	1.572615	2.939343
H	0.425593	2.892873	1.101795
H	3.436161	−1.153825	−0.358332
H	3.778501	0.512159	−0.841417
H	2.927803	−0.564375	−1.956697
H	−2.791351	1.903747	−1.957413
H	−2.129320	0.506804	−2.815738
H	−3.306170	0.274296	−1.503636
H	−0.018469	−3.017897	1.770062
H	−0.709612	−3.225246	0.155714
H	−1.734805	−2.654353	1.478531

Me_3BV (010 100 100 0)

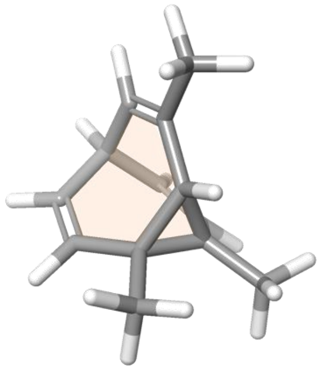

Table S67. Coordinates for the optimised geometry of **Me_3BV (010 100 100 0).**

Atom	Coordinates / Å x	y	z
C	−0.127415	0.906429	0.159351
C	1.307753	1.012170	−0.220964
C	2.107753	−0.012776	−0.581596
C	−1.106254	−0.048445	−0.514680
C	−0.637783	−1.005708	−1.564654
C	0.554680	−1.617280	−1.664373
C	−0.697875	−0.252013	0.969793
C	0.167643	−1.407194	1.363105
C	1.203167	−1.940535	0.692903
C	1.651354	−1.444317	−0.652348
C	1.877420	2.409679	−0.186037
C	−2.484238	0.515353	−0.825533
C	−1.690160	0.119523	2.060971
H	−0.534257	1.887173	0.405764
H	3.146288	0.168828	−0.842394
H	−1.356325	−1.229476	−2.352290
H	0.745507	−2.288175	−2.496056
H	−0.071007	−1.870175	2.319891
H	1.739445	−2.783628	1.116946
H	2.505147	−2.055963	−0.971102
H	2.933975	2.439343	−0.472620
H	1.795495	2.825930	0.823605
H	1.327976	3.058979	−0.875872
H	−2.532431	0.861221	−1.864616
H	−3.248539	−0.256339	−0.683820
H	−2.746860	1.373546	−0.199989
H	−2.171767	1.086874	1.890502
H	−2.473200	−0.642829	2.134568
H	−1.185495	0.189806	3.031546

Me_3BV (100 100 100 0)

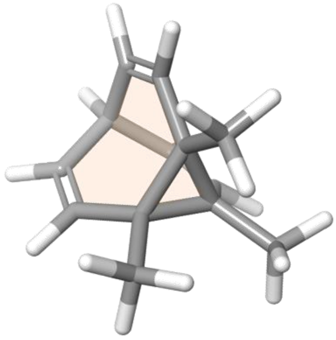

Table S68. Coordinates for the optimised geometry of **Me_3BV (100 100 100 0).**

	Coordinates / Å		
**Atom**	**x**	**y**	**z**
C	−0.221887	0.090733	0.977020
C	1.176190	0.062588	1.529955
C	2.346745	−0.051032	0.881333
C	−0.591514	−0.770542	−0.261562
C	0.445781	−1.639344	−0.917572
C	1.759916	−1.418408	−1.085075
C	−0.510854	0.775521	−0.386823
C	0.605172	1.415782	−1.165090
C	1.887975	1.036160	−1.283936
C	2.451304	−0.177175	−0.608335
C	−1.230474	0.235344	2.112412
C	−1.954949	−1.452774	−0.315228
C	−1.796854	1.577538	−0.560743
H	1.249550	0.146603	2.614210
H	3.273575	−0.053595	1.446560
H	0.077147	−2.585223	−1.314399
H	2.367923	−2.163862	−1.588194
H	0.332990	2.318660	−1.711695
H	2.565557	1.624268	−1.895094
H	3.513700	−0.253962	−0.871986
H	−1.038986	1.162037	2.666089
H	−2.275045	0.264805	1.803087
H	−1.131213	−0.605218	2.809255
H	−2.771575	−0.892219	0.139286
H	−2.239881	−1.636104	−1.357911
H	−1.909551	−2.418901	0.201030
H	−2.663235	1.184658	−0.029034
H	−2.069997	1.619604	−1.621689
H	−1.647514	2.604059	−0.205870

Me_4BV

Me_4BV (000 000 111 1)

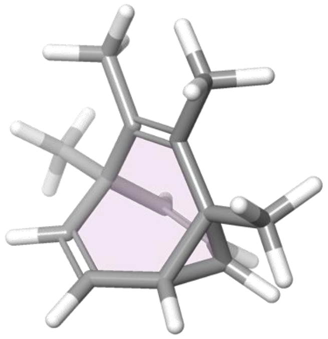

Table S69. Coordinates for the optimised geometry of **Me_4BV (000 000 111 1).**

	Coordinates / Å		
**Atom**	**x**	**y**	**z**
C	−1.513155	−1.366682	−0.612927
C	−0.320131	−1.880062	−1.289367
C	0.960517	−1.632154	−0.981616
C	−1.587018	−1.128044	0.873574
C	−0.455838	−1.373367	1.770920
C	0.847084	−1.213129	1.495208
C	−1.632814	0.057580	−0.080743
C	−0.449469	1.035806	−0.146735
C	0.883505	0.715800	−0.114640
C	1.390649	−0.724951	0.156028
C	−3.021770	0.657631	−0.288000
C	−0.858536	2.504470	−0.217232
C	1.970665	1.745860	−0.356749
C	2.933577	−0.858839	0.267535
H	−2.413284	−1.798568	−1.047827
H	−0.507496	−2.535952	−2.137545
H	1.731965	−2.086153	−1.597689
H	−2.530529	−1.405055	1.340441
H	−0.714338	−1.716667	2.770753
H	1.559992	−1.423739	2.288481
H	−3.303900	1.291895	0.558413
H	−3.064694	1.240048	−1.214107
H	−3.794837	−0.116461	−0.364140
H	−0.082384	3.195198	0.120706
H	−1.145673	2.770787	−1.239065
H	−1.694834	2.716736	0.455138
H	2.701308	1.375354	−1.082225
H	1.613282	2.677145	−0.801089
H	2.483935	1.988277	0.578662
H	3.441240	−0.620629	−0.674687
H	3.345319	−0.206070	1.046970
H	3.227662	−1.886066	0.523551

Me$_4$BV (000 001 011 1)

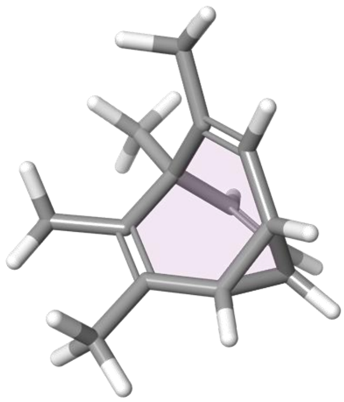

Table S70. Coordinates for the optimised geometry of **Me$_4$BV (000 001 011 1).**

Atom	Coordinates / Å x	y	z
C	−0.900441	−1.815609	−1.496571
C	−0.222111	−0.801937	−2.302963
C	0.477677	0.237823	−1.827493
C	−0.316391	−2.308087	−0.174761
C	0.979542	−1.845152	0.332778
C	1.499905	−0.604206	0.279920
C	−1.560951	−1.448990	−0.175243
C	−1.623347	−0.074837	0.374719
C	−0.605314	0.821592	0.359649
C	0.736545	0.567653	−0.365387
C	2.891440	−0.379060	0.829342
C	−2.969058	0.255862	1.001803
C	−0.755307	2.144789	1.066740
C	1.603923	1.836256	−0.383924
H	−1.430424	−2.580842	−2.071966
H	−0.311298	−0.910605	−3.389638
H	0.924331	0.923027	−2.558472
H	−0.509733	−3.367248	0.021998
H	1.596906	−2.628818	0.794301
H	−2.474137	−2.022203	0.010788
H	3.601872	−0.045406	0.042250
H	2.913083	0.391224	1.631793
H	3.293356	−1.313368	1.256032
H	−2.946973	0.184371	2.111070
H	−3.323643	1.269260	0.740245
H	−3.741829	−0.448799	0.651068
H	0.070123	2.303775	1.789006
H	−1.692319	2.208098	1.637740
H	−0.732599	3.005997	0.368366
H	2.524408	1.652606	−0.961478
H	1.897062	2.181471	0.618004
H	1.068042	2.658196	−0.885717

Me_4BV (000 001 101 1)

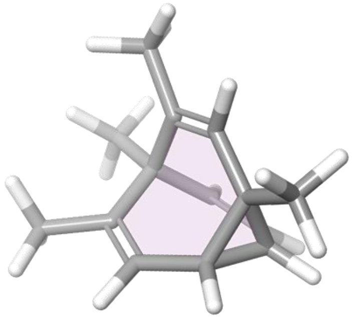

Table S71. Coordinates for the optimised geometry of **Me_4BV (000 001 101 1).**

Atom	Coordinates / Å		
	x	y	z
C	1.500635	−1.060132	−1.324825
C	0.461769	−0.465112	−2.170839
C	−0.650013	0.172730	−1.771755
C	1.178589	−1.724857	−0.010690
C	−0.190657	−1.820615	0.522033
C	−1.204435	−0.931110	0.429629
C	1.963291	−0.422708	−0.024964
C	1.336079	0.833190	0.474553
C	0.052181	1.251806	0.390601
C	−1.045413	0.417929	−0.311995
C	−2.516193	−1.254407	1.098234
C	3.444874	−0.529747	0.280997
C	−0.322576	2.566557	1.027563
C	−2.392420	1.189103	−0.394781
H	2.266617	−1.554086	−1.919404
H	0.619505	−0.562364	−3.243340
H	−1.314088	0.549196	−2.546149
H	1.749115	−2.627422	0.201183
H	−0.379591	−2.743341	1.068951
H	2.031051	1.493099	0.993677
H	−3.324653	−1.310156	0.363119
H	−2.762125	−0.498720	1.850689
H	−2.491604	−2.219976	1.615774
H	3.991793	0.298660	−0.182399
H	3.877051	−1.463800	−0.095109
H	3.615586	−0.497816	1.362660
H	−1.088814	2.418541	1.794851
H	−0.692677	3.271375	0.276984
H	0.529239	3.048100	1.520771
H	−3.159768	0.607832	−0.922140
H	−2.795700	1.436564	0.593282
H	−2.286647	2.131686	−0.947162

Me$_4$BV (000 001 110 1)

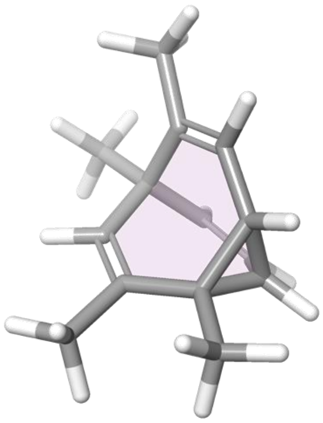

Table S72. Coordinates for the optimised geometry of **Me$_4$BV (000 001 110 1).**

Atom	Coordinates / Å		
	x	y	z
C	−0.948008	−1.272415	−1.289278
C	0.015930	−0.575685	−2.150304
C	0.989277	0.267592	−1.771008
C	−0.582798	−1.763138	0.089770
C	0.757190	−1.577253	0.673225
C	1.610478	−0.540292	0.524215
C	−1.601512	−0.639326	−0.065039
C	−1.236442	0.767926	0.353115
C	−0.013967	1.337843	0.245427
C	1.237235	0.683163	−0.327925
C	2.947434	−0.578453	1.215738
C	−3.047804	−1.054789	0.167580
C	−2.334669	1.619886	0.957879
C	2.346390	1.764153	−0.358243
H	−1.572271	−1.949517	−1.869798
H	−0.067961	−0.789157	−3.214248
H	1.633831	0.683734	−2.540675
H	−0.985739	−2.740701	0.350272
H	1.080437	−2.408727	1.297970
H	0.112791	2.358367	0.600679
H	3.761614	−0.527349	0.486338
H	3.042434	0.252885	1.920954
H	3.090990	−1.501009	1.789197
H	−3.234276	−2.084930	−0.158412
H	−3.733344	−0.413767	−0.396178
H	−3.301359	−1.007371	1.231612
H	−1.987659	2.622281	1.232714
H	−3.154929	1.753256	0.245475
H	−2.722582	1.157399	1.870967
H	2.570088	2.149730	0.643848
H	2.048812	2.626219	−0.970287
H	3.280390	1.379443	−0.785576

Me_4BV (000 001 111 0)

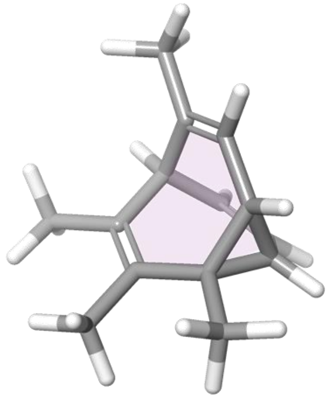

Table S73. Coordinates for the optimised geometry of **Me_4BV (000 001 111 0).**

	Coordinates / Å		
**Atom**	**x**	**y**	**z**
C	−0.642888	−1.412711	−1.408857
C	0.113063	−0.531381	−2.308044
C	0.979287	0.427875	−1.949892
C	−0.080612	−1.908133	−0.097524
C	1.264327	−1.562926	0.387643
C	1.922643	−0.400731	0.223647
C	−1.259259	−0.938242	−0.096312
C	−1.082518	0.515567	0.359276
C	0.050606	1.263874	0.221429
C	1.288589	0.758767	−0.512443
C	3.301808	−0.179150	0.770322
C	−2.589745	−1.597997	0.255092
C	−2.319384	1.143707	0.986615
C	0.236112	2.659436	0.771988
H	−1.219025	−2.145690	−1.970966
H	−0.050531	−0.687005	−3.372334
H	1.479268	1.012654	−2.715429
H	−0.312397	−2.947170	0.132293
H	1.767274	−2.358027	0.935184
H	2.026521	1.572223	−0.559104
H	3.999034	0.052029	−0.041489
H	3.300030	0.656588	1.477584
H	3.684318	−1.059798	1.296771
H	−2.782249	−1.544988	1.331535
H	−3.415217	−1.126795	−0.287865
H	−2.603690	−2.660004	−0.017924
H	−2.481950	0.740757	1.991148
H	−3.206608	0.950971	0.375542
H	−2.277138	2.232243	1.062492
H	0.099269	3.399780	−0.022205
H	−0.435065	2.896700	1.600306
H	1.246128	2.777575	1.181521

Me_4BV (000 010 011 1)

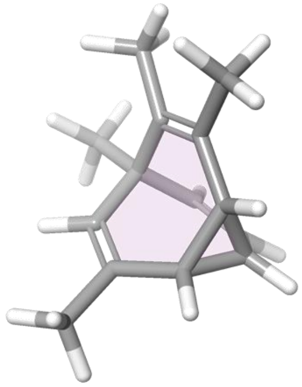

Table S74. Coordinates for the optimised geometry of **Me_4BV (000 010 011 1).**

Atom	Coordinates / Å		
	x	y	z
C	1.318800	0.014529	−1.856230
C	0.502639	−1.194586	−1.989066
C	−0.357433	−1.691438	−1.086830
C	1.940481	0.447768	−0.549111
C	1.779470	−0.308361	0.710250
C	0.666181	−0.968425	1.089098
C	0.814875	1.251887	−1.152693
C	−0.540703	1.356860	−0.534089
C	−1.228181	0.355984	0.090659
C	−0.621727	−1.059817	0.273616
C	2.982260	−0.300135	1.621812
C	−1.136761	2.749908	−0.643066
C	−2.626733	0.559929	0.633602
C	−1.543423	−2.047388	1.037841
H	1.925336	0.194905	−2.741572
H	0.606119	−1.732872	−2.929102
H	−0.904273	−2.593877	−1.346955
H	2.926843	0.900428	−0.642589
H	0.666732	−1.471579	2.053266
H	1.131867	2.180359	−1.626805
H	2.814801	−0.866881	2.543754
H	3.234929	0.727170	1.904993
H	3.844950	−0.742613	1.112547
H	−0.388140	3.498403	−0.923766
H	−1.922682	2.768435	−1.405002
H	−1.544049	3.087582	0.315611
H	−2.632870	0.453079	1.722790
H	−3.055578	1.538867	0.408360
H	−3.317381	−0.167683	0.195879
H	−1.062849	−3.027467	1.162173
H	−1.787741	−1.685617	2.043949
H	−2.485761	−2.227356	0.506676

Me_4BV (000 010 101 1)

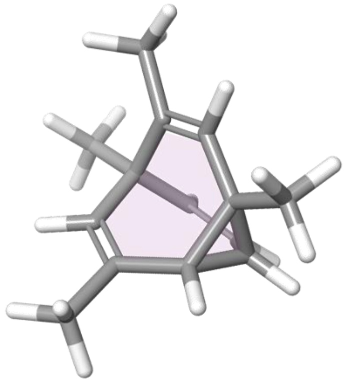

Table S75. Coordinates for the optimised geometry of **Me_4BV (000 010 101 1).**

	Coordinates / Å		
**Atom**	**x**	**y**	**z**
C	−1.324469	−0.643752	−1.406022
C	−0.145683	−0.270666	−2.199154
C	1.003369	0.262688	−1.752963
C	−1.779260	0.143864	−0.200964
C	−1.078976	1.358479	0.280710
C	0.253880	1.568701	0.248844
C	−1.217191	−1.258756	−0.017533
C	0.115836	−1.467830	0.619108
C	1.236199	−0.713460	0.540851
C	1.283976	0.580600	−0.289516
C	−1.978367	2.425730	0.856941
C	−2.224825	−2.343774	0.312201
C	2.469786	−1.149290	1.287964
C	2.660331	1.289027	−0.240981
H	−2.124805	−1.021891	−2.039371
H	−0.223456	−0.457453	−3.268527
H	1.785897	0.469038	−2.478350
H	−2.860973	0.234019	−0.105785
H	0.642181	2.502902	0.647653
H	0.174749	−2.366773	1.232493
H	−1.421365	3.300727	1.208161
H	−2.540345	2.026803	1.708095
H	−2.691118	2.768436	0.099367
H	−2.396930	−2.389963	1.393114
H	−1.859504	−3.320188	−0.024388
H	−3.192794	−2.168064	−0.170309
H	2.766203	−0.391958	2.020155
H	2.312724	−2.082118	1.840850
H	3.298823	−1.325895	0.595846
H	3.462048	0.653336	−0.636024
H	2.935585	1.579592	0.780136
H	2.658473	2.207887	−0.842600

Me$_4$BV (000 010 110 1)

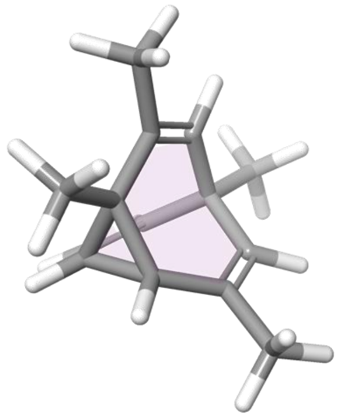

Table S76. Coordinates for the optimised geometry of **Me$_4$BV (000 010 110 1).**

	Coordinates / Å		
**Atom**	**x**	**y**	**z**
C	−0.619108	0.484198	1.669556
C	0.529407	−0.298743	2.147948
C	1.496325	−0.864597	1.407707
C	−0.539492	1.402110	0.473002
C	0.701265	1.593025	−0.317847
C	1.629515	0.647627	−0.573442
C	−1.405044	0.145893	0.404601
C	−0.986327	−1.024046	−0.462584
C	0.283883	−1.434915	−0.684378
C	1.530207	−0.792016	−0.105471
C	0.904654	2.987708	−0.859283
C	−2.903288	0.402035	0.501855
C	−2.085767	−1.810747	−1.147628
C	2.770007	−1.563018	−0.606563
H	−1.200765	0.869165	2.505644
H	0.596208	−0.419664	3.227486
H	2.286307	−1.410844	1.915242
H	−1.083042	2.339311	0.592641
H	2.503015	0.911390	−1.164230
H	0.451554	−2.293504	−1.330633
H	0.954830	3.709040	−0.036773
H	1.827878	3.082452	−1.440614
H	0.071016	3.263668	−1.513738
H	−3.130272	1.259139	1.146877
H	−3.420159	−0.463123	0.929672
H	−3.325714	0.624888	−0.483320
H	−2.659919	−1.165217	−1.819743
H	−2.761421	−2.252639	−0.408518
H	−1.697300	−2.635646	−1.755427
H	2.840219	−1.546001	−1.701684
H	3.698978	−1.131180	−0.212832
H	2.742348	−2.615750	−0.297523

Me_4BV (000 010 111 0)

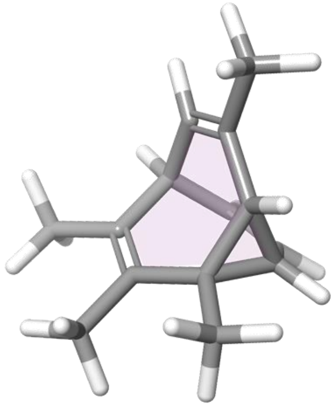

Table S77. Coordinates for the optimised geometry of **Me_4BV (000 010 111 0).**

	Coordinates / Å		
**Atom**	**x**	**y**	**z**
C	0.598862	−0.743330	−1.651858
C	0.725804	0.614842	−2.194589
C	0.681909	1.763082	−1.504739
C	1.137715	−1.152126	−0.300746
C	1.840843	−0.215142	0.606339
C	1.563087	1.096658	0.736556
C	−0.370368	−1.123947	−0.533950
C	−1.240618	−0.065020	0.155176
C	−0.901951	1.242357	0.351649
C	0.465910	1.797398	−0.019883
C	2.949516	−0.822179	1.429610
C	−1.008508	−2.497365	−0.725456
C	−2.575866	−0.576025	0.678441
C	−1.811919	2.293131	0.944771
H	0.739468	−1.482304	−2.439370
H	0.875137	0.683152	−3.270193
H	0.787778	2.710588	−2.022647
H	1.589673	−2.143742	−0.280241
H	2.130852	1.709659	1.430392
H	0.522302	2.853329	0.278908
H	2.555293	−1.626855	2.059245
H	3.721941	−1.239420	0.774967
H	3.431000	−0.091209	2.087771
H	−1.176758	−2.988030	0.238480
H	−1.957052	−2.417776	−1.266299
H	−0.366454	−3.168584	−1.308389
H	−3.266849	−0.752226	−0.151844
H	−2.443991	−1.506987	1.238501
H	−3.064520	0.096424	1.386534
H	−1.697725	3.243337	0.410162
H	−2.873508	2.048952	0.865779
H	−1.561002	2.459359	1.996921

Me_4BV (000 011 001 1)

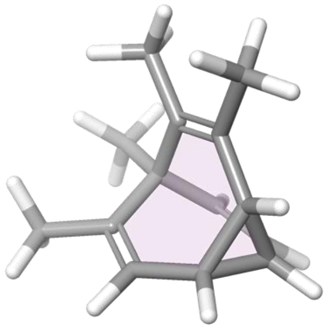

Table S78. Coordinates for the optimised geometry of **Me_4BV (000 011 001 1).**

Atom	Coordinates / Å x	y	z
C	0.560252	−2.150210	−1.189479
C	−0.263269	−1.306757	−2.054694
C	−0.841276	−0.150198	−1.700624
C	1.482280	−1.532693	−0.148664
C	1.666173	−0.076112	0.047370
C	0.672713	0.847103	0.036514
C	0.258436	−2.300214	0.299821
C	−0.902087	−1.666581	0.934506
C	−1.414554	−0.442958	0.702121
C	−0.790763	0.511920	−0.332181
C	3.114895	0.311819	0.297524
C	0.971432	2.281309	0.391864
C	−2.663591	−0.026450	1.447163
C	−1.631992	1.790639	−0.471308
H	0.953524	−3.053094	−1.666798
H	−0.403058	−1.659949	−3.082511
H	−1.425262	0.376958	−2.465127
H	2.410176	−2.098277	−0.021161
H	0.481353	−3.297046	0.693116
H	−1.417170	−2.293497	1.676033
H	3.407105	1.236331	−0.232852
H	3.792464	−0.482805	−0.058575
H	3.331471	0.460929	1.378221
H	2.004247	2.417791	0.742334
H	0.309610	2.631112	1.208966
H	0.813847	2.973561	−0.459865
H	−2.982631	−0.822111	2.141402
H	−3.513760	0.169756	0.758856
H	−2.512364	0.898490	2.046846
H	−2.650702	1.534435	−0.804928
H	−1.717855	2.364947	0.462585
H	−1.198274	2.450828	−1.239882

Me$_4$BV (000 011 010 1)

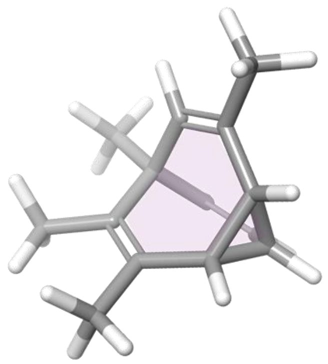

Table S79. Coordinates for the optimised geometry of **Me$_4$BV (000 011 010 1).**

Atom	Coordinates / Å x	y	z
C	0.991853	−0.185290	−2.041306
C	0.347343	1.130014	−2.058758
C	−0.278335	1.736272	−1.038173
C	0.433287	−1.344329	−1.250994
C	−0.807895	−1.259370	−0.424235
C	−1.236521	−0.176379	0.288576
C	1.744099	−0.716823	−0.843666
C	1.895178	0.041721	0.415474
C	0.962627	0.851274	0.957319
C	−0.411599	1.135432	0.354968
C	−1.607669	−2.550914	−0.419531
C	−2.545902	−0.182399	1.048717
C	3.213446	−0.148867	1.125094
C	−1.048816	2.237552	1.242742
H	1.418037	−0.442062	−3.009131
H	0.376759	1.657358	−3.010162
H	−0.724027	2.710640	−1.220002
H	0.534306	−2.303879	−1.757340
H	2.627721	−1.305782	−1.086332
H	1.188359	1.339245	1.902684
H	−2.498860	−2.448320	−1.047077
H	−1.902040	−2.836216	0.595811
H	−1.028502	−3.396476	−0.805616
H	−3.185363	0.641481	0.716562
H	−3.141236	−1.087059	0.906841
H	−2.363847	−0.086887	2.123667
H	3.357493	−1.204678	1.377880
H	3.277507	0.426651	2.054554
H	4.039008	0.169985	0.479995
H	−0.418783	3.136919	1.276792
H	−2.027667	2.556712	0.865234
H	−1.179959	1.904475	2.279412

Me_4BV (000 011 011 0)

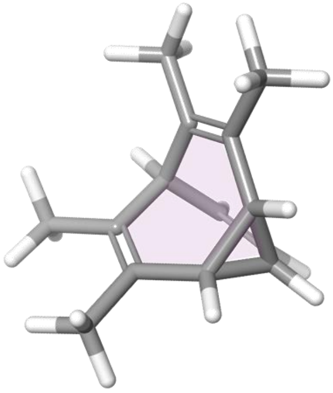

Table S80. Coordinates for the optimised geometry of **Me_4BV (000 011 011 0).**

	Coordinates / Å		
**Atom**	**x**	**y**	**z**
C	−0.146131	−1.569668	−1.639697
C	−0.306067	−0.341803	−2.425949
C	−0.299755	0.911830	−1.949234
C	0.778307	−1.652178	−0.445695
C	1.622132	−0.514261	0.020086
C	1.242086	0.789951	0.036075
C	−0.717183	−1.720033	−0.247460
C	−1.508684	−0.656315	0.435094
C	−1.255186	0.676643	0.367104
C	−0.120335	1.232374	−0.485973
C	3.003487	−0.943026	0.472763
C	2.077143	1.924793	0.573249
C	−2.680991	−1.200949	1.226273
C	−2.019419	1.738922	1.116272
H	−0.188055	−2.461609	−2.261281
H	−0.442040	−0.474161	−3.497014
H	−0.428025	1.746295	−2.631264
H	1.280228	−2.612703	−0.328835
H	−1.083909	−2.719971	−0.015458
H	−0.163373	2.330082	−0.434909
H	3.756077	−0.159175	0.354557
H	2.974802	−1.256784	1.520946
H	3.364960	−1.786356	−0.126809
H	2.429575	2.554454	−0.249905
H	2.943926	1.599027	1.152088
H	1.478840	2.544960	1.250325
H	−3.109392	−2.080108	0.731394
H	−2.352946	−1.498537	2.227165
H	−3.505095	−0.488635	1.317084
H	−1.323301	2.417822	1.621762
H	−2.628349	2.324961	0.420553
H	−2.673329	1.344158	1.896691

Me_4BV (000 011 100 1)

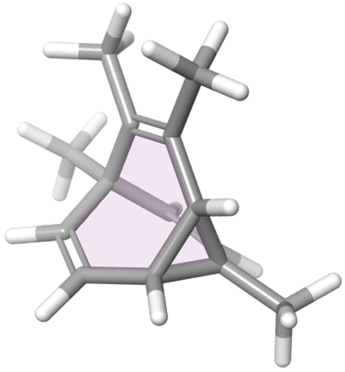

Table S81. Coordinates for the optimised geometry of **Me_4BV (000 011 100 1).**

	Coordinates / Å		
**Atom**	**x**	**y**	**z**
C	1.655939	−0.377197	1.172492
C	0.588814	−1.101774	1.870172
C	−0.656460	−1.338428	1.429909
C	1.374814	0.818000	0.296295
C	0.004260	1.364598	0.048792
C	−1.158403	0.652139	−0.025731
C	1.891938	−0.468136	−0.328699
C	0.998750	−1.284043	−1.186158
C	−0.321882	−1.484557	−1.047418
C	−1.175236	−0.895196	0.069692
C	0.001403	2.872268	−0.142442
C	−2.503904	1.324594	−0.198030
C	3.338302	−0.461580	−0.782385
C	−2.583398	−1.526127	−0.090236
H	2.547552	−0.296925	1.791975
H	0.843554	−1.471597	2.861542
H	−1.335757	−1.876525	2.085725
H	2.113438	1.611704	0.409372
H	1.473122	−1.769220	−2.038205
H	−0.818571	−2.102740	−1.791158
H	−0.301333	3.371142	0.783627
H	−0.659433	3.179095	−0.959335
H	0.992725	3.249137	−0.417258
H	−2.469177	2.414642	−0.138291
H	−2.937852	1.067844	−1.169342
H	−3.190989	1.016641	0.596386
H	3.419366	−0.055951	−1.796817
H	3.744101	−1.479157	−0.781218
H	3.972588	0.148740	−0.129836
H	−3.269004	−1.212107	0.705868
H	−3.042148	−1.266371	−1.051974
H	−2.537120	−2.622916	−0.047312

Me_4BV (000 011 101 0)

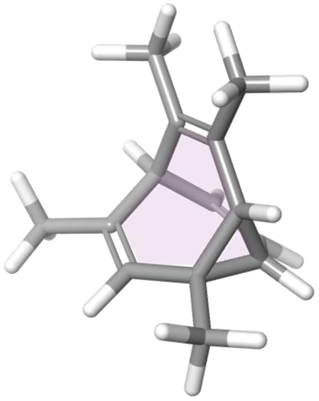

Table S82. Coordinates for the optimised geometry of **Me_4BV (000 011 101 0).**

	Coordinates / Å		
**Atom**	**x**	**y**	**z**
C	−0.866486	−1.407045	−1.226806
C	−0.762175	−0.314628	−2.204368
C	−0.320678	0.931133	−1.973807
C	0.137480	−1.600647	−0.114822
C	1.319252	−0.710693	0.103941
C	1.360604	0.627382	−0.134814
C	−1.290636	−1.194292	0.222304
C	−1.584451	0.173119	0.736276
C	−0.988623	1.335758	0.402627
C	0.134532	1.397564	−0.615562
C	2.519258	−1.448514	0.664847
C	2.582795	1.494989	0.032597
C	−2.168002	−2.272747	0.828086
C	−1.396199	2.641178	1.022057
H	−1.216322	−2.324001	−1.697860
H	−1.073925	−0.555704	−3.218490
H	−0.287859	1.649666	−2.786414
H	0.383263	−2.647094	0.067106
H	−2.381303	0.221615	1.477413
H	0.413590	2.453543	−0.739406
H	3.106991	−1.884028	−0.149279
H	3.166669	−0.812254	1.273920
H	2.201575	−2.260213	1.329413
H	3.509122	0.932042	0.164796
H	2.457240	2.158654	0.894079
H	2.730976	2.112768	−0.860386
H	−3.223471	−2.072196	0.613885
H	−2.034079	−2.309074	1.914768
H	−1.932656	−3.266783	0.431712
H	−0.546505	3.095565	1.541988
H	−2.205091	2.520064	1.750068
H	−1.744887	3.334873	0.250133

Me$_4$BV (000 011 110 0)

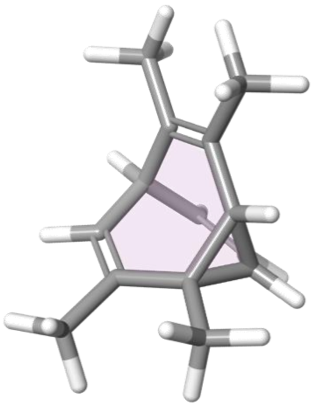

Table S83. Coordinates for the optimised geometry of **Me$_4$BV (000 011 110 0).**

	Coordinates / Å		
**Atom**	**x**	**y**	**z**
C	−0.686969	1.006249	1.425167
C	−0.116565	0.031833	2.365093
C	0.567072	−1.079218	2.054496
C	−0.019504	1.365314	0.119402
C	1.268815	0.771206	−0.351139
C	1.692238	−0.497121	−0.108867
C	−1.356739	0.629208	0.103666
C	−1.420076	−0.817637	−0.334843
C	−0.467850	−1.748021	−0.104174
C	0.819774	−1.497190	0.633771
C	2.098827	1.735482	−1.175738
C	3.028320	−1.056987	−0.526154
C	−2.553779	1.492576	−0.270928
C	−2.646686	−1.266878	−1.100403
H	−1.129176	1.844968	1.960793
H	−0.260376	0.252171	3.420834
H	0.950023	−1.717378	2.844053
H	−0.068822	2.430665	−0.108502
H	−0.601655	−2.764352	−0.464809
H	1.342242	−2.462869	0.679702
H	1.453533	2.385881	−1.777497
H	2.754294	1.233553	−1.892146
H	2.704026	2.367731	−0.518521
H	2.896782	−1.778522	−1.338762
H	3.741629	−0.297117	−0.852446
H	3.501268	−1.570877	0.318413
H	−2.439348	2.524778	0.080620
H	−3.472083	1.102895	0.180279
H	−2.676671	1.538351	−1.357915
H	−2.603430	−2.326203	−1.377548
H	−3.549044	−1.133386	−0.495430
H	−2.750073	−0.699103	−2.030467

Me_4BV (000 100 011 1)

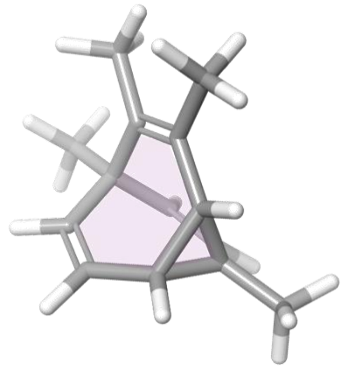

Table S84. Coordinates for the optimised geometry of **Me_4BV (000 100 011 1).**

Atom	Coordinates / Å		
	**x**	**y**	**z**
C	1.707927	0.693188	−0.928246
C	0.647993	1.488153	−1.556814
C	−0.639418	1.565376	−1.186473
C	1.826492	0.489016	0.575681
C	0.819397	1.066010	1.498481
C	−0.498085	1.221756	1.289894
C	1.445308	−0.668179	−0.334142
C	0.100681	−1.324051	−0.318235
C	−1.110099	−0.702947	−0.203436
C	−1.225153	0.828452	0.009198
C	3.232786	0.465369	1.141034
C	0.186416	−2.837178	−0.431134
C	−2.415721	−1.465279	−0.283795
C	−2.680912	1.339737	0.170192
H	2.645948	0.786963	−1.472763
H	0.948970	2.064586	−2.429519
H	−1.303846	2.189623	−1.778028
H	1.195905	1.391697	2.467285
H	−1.088878	1.649132	2.096314
H	2.242531	−1.382878	−0.538982
H	3.953263	0.034272	0.436976
H	3.266670	−0.133343	2.057863
H	3.567393	1.481567	1.376653
H	−0.013001	−3.152301	−1.460242
H	1.178276	−3.210110	−0.152909
H	−0.510089	−3.337597	0.249029
H	−2.936171	−1.434310	0.678416
H	−3.062078	−1.038269	−1.057036
H	−2.302607	−2.517095	−0.555586
H	−2.706359	2.423224	0.350024
H	−3.190196	0.866360	1.018372
H	−3.283342	1.159058	−0.728072

Me_4BV (000 100 101 1)

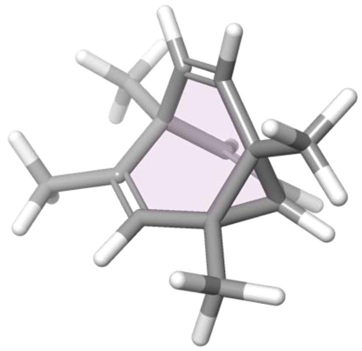

Table S85. Coordinates for the optimised geometry of **Me_4BV (000 100 101 1).**

Atom	Coordinates / Å		
	x	y	z
C	1.420147	−0.073977	1.119931
C	0.299926	−0.309826	2.042757
C	−0.994424	−0.494388	1.736559
C	1.537893	−0.749117	−0.238009
C	0.458341	−1.649988	−0.734881
C	−0.864615	−1.583162	−0.509173
C	1.282293	0.774477	−0.134547
C	−0.045362	1.347961	−0.525962
C	−1.294643	0.860282	−0.347590
C	−1.543905	−0.501934	0.317271
C	2.924650	−1.238753	−0.624198
C	2.430401	1.730668	−0.422235
C	−2.474335	1.662870	−0.831980
C	−3.042613	−0.876792	0.414872
H	2.350011	0.042888	1.674046
H	0.563590	−0.325511	3.098781
H	−1.695490	−0.641304	2.553995
H	0.789819	−2.472427	−1.368142
H	−1.496774	−2.335968	−0.973279
H	0.027184	2.314052	−1.025802
H	3.090588	−1.095472	−1.697378
H	3.725567	−0.725215	−0.084090
H	3.031952	−2.306160	−0.398810
H	3.368540	1.428418	0.052495
H	2.601796	1.800628	−1.501813
H	2.199417	2.733329	−0.044107
H	−2.172246	2.611871	−1.288730
H	−3.141650	1.909033	−0.000313
H	−3.034021	1.108243	−1.591370
H	−3.606817	−0.151992	1.014262
H	−3.515958	−0.935317	−0.572733
H	−3.179262	−1.857417	0.890171

Me_4BV (000 100 110 1)

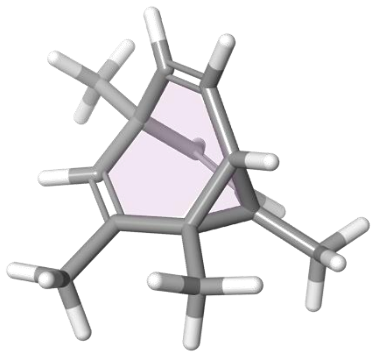

Table S86. Coordinates for the optimised geometry of **Me_4BV (000 100 110 1).**

Atom	Coordinates / Å x	y	z
C	1.066784	−0.501946	−1.146609
C	−0.076062	−0.545699	−2.071149
C	−1.380269	−0.442956	−1.771962
C	1.044895	−1.099582	0.252244
C	−0.203574	−1.724870	0.783260
C	−1.482704	−1.405725	0.522981
C	1.141919	0.440930	0.049361
C	−0.044234	1.319779	0.426574
C	−1.352475	1.036213	0.227038
C	−1.894716	−0.249692	−0.362816
C	2.290446	−1.863235	0.678657
C	2.506602	1.104168	0.226487
C	0.257577	2.634108	1.121997
C	−3.435380	−0.177766	−0.404071
H	1.993908	−0.641715	−1.701020
H	0.179621	−0.679182	−3.120810
H	−2.103329	−0.496137	−2.581012
H	−0.054546	−2.561972	1.464713
H	−2.266519	−1.986813	1.001219
H	−2.100091	1.756876	0.550887
H	3.176746	−1.600719	0.093928
H	2.145162	−2.942110	0.548896
H	2.507107	−1.670455	1.734864
H	2.734014	1.240113	1.288781
H	3.324139	0.522991	−0.210833
H	2.535776	2.077195	−0.275103
H	0.863767	2.465624	2.017829
H	0.788029	3.315190	0.449459
H	−0.648970	3.154550	1.451142
H	−3.782904	0.657354	−1.025556
H	−3.860447	−0.037804	0.598056
H	−3.870271	−1.096715	−0.817432

Me_4BV (000 100 111 0)

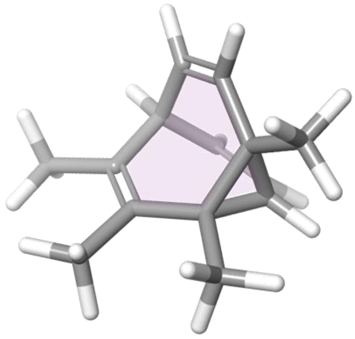

Table S87. Coordinates for the optimised geometry of **Me_4BV (000 100 111 0).**

	Coordinates / Å		
**Atom**	**x**	**y**	**z**
C	1.139553	−0.406893	1.254845
C	0.291659	−1.491905	1.766491
C	−0.718977	−2.098933	1.130305
C	1.596444	−0.274282	−0.189937
C	1.141046	−1.263229	−1.210018
C	−0.024829	−1.926511	−1.258247
C	0.601220	0.752199	0.421010
C	−0.850181	0.784169	−0.091920
C	−1.648313	−0.296501	−0.328482
C	−1.131296	−1.723916	−0.260500
C	3.039635	0.153175	−0.409107
C	1.145385	2.107337	0.880505
C	−1.388095	2.173286	−0.410437
C	−3.106296	−0.222586	−0.718310
H	1.892775	−0.155102	2.000555
H	0.516572	−1.828932	2.776547
H	−1.274592	−2.887731	1.626718
H	1.848386	−1.479271	−2.009705
H	−0.210043	−2.622913	−2.070170
H	−1.933068	−2.417254	−0.549496
H	3.679292	−0.721956	−0.572506
H	3.462693	0.691639	0.443800
H	3.111111	0.800719	−1.289679
H	0.423224	2.622859	1.522512
H	2.060238	2.018184	1.475498
H	1.381890	2.741327	0.019968
H	−2.232635	2.165726	−1.104476
H	−1.701345	2.681911	0.506426
H	−0.630608	2.780005	−0.916540
H	−3.579367	0.727445	−0.458190
H	−3.220069	−0.394195	−1.793167
H	−3.681410	−0.987869	−0.184292

Me_4BV (000 101 001 1)

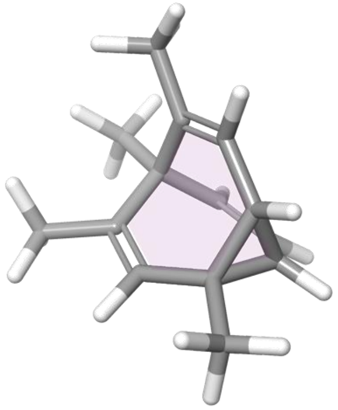

Table S88. Coordinates for the optimised geometry of **Me_4BV (000 101 001 1).**

	Coordinates / Å		
**Atom**	**x**	**y**	**z**
C	1.987718	−0.027817	−1.085887
C	1.054307	−0.914190	−1.787399
C	−0.245762	−1.110151	−1.516126
C	1.956664	0.167221	0.421095
C	0.927751	−0.512578	1.257398
C	−0.365528	−0.795330	0.977828
C	1.554824	1.297617	−0.512865
C	0.172552	1.795838	−0.607199
C	−0.987454	1.103531	−0.556206
C	−1.016831	−0.433553	−0.378131
C	3.303410	0.308883	1.104005
C	−1.204614	−1.464321	2.037601
C	−2.288185	1.860302	−0.643718
C	−2.460928	−0.996763	−0.493754
H	2.971265	−0.033160	−1.551559
H	1.474815	−1.467689	−2.624900
H	−0.787541	−1.808520	−2.149502
H	1.288830	−0.805890	2.243068
H	2.274934	2.106035	−0.626511
H	0.108312	2.876693	−0.724956
H	3.708590	−0.676605	1.358482
H	4.037529	0.814661	0.466865
H	3.205765	0.893659	2.025233
H	−0.644229	−1.640665	2.962587
H	−2.061877	−0.837816	2.302838
H	−1.561261	−2.439807	1.693586
H	−2.862450	1.547522	−1.520928
H	−2.888208	1.701467	0.257657
H	−2.134282	2.941398	−0.734945
H	−2.908368	−0.766116	−1.469154
H	−2.478571	−2.090050	−0.397909
H	−3.131179	−0.593809	0.273405

Me_4BV (000 101 010 1)

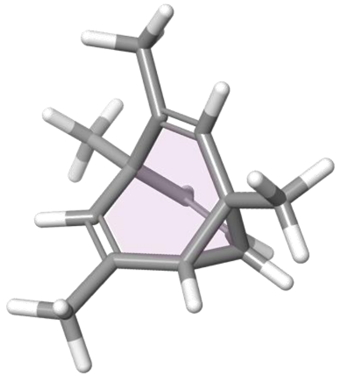

Table S89. Coordinates for the optimised geometry of **Me_4BV (000 101 010 1).**

	Coordinates / Å		
**Atom**	**x**	**y**	**z**
C	−1.517099	0.505412	1.174530
C	−0.411905	0.186664	2.088168
C	0.815131	−0.252557	1.763609
C	−1.297679	1.172248	−0.176331
C	0.080475	1.492142	−0.649984
C	1.235559	0.813097	−0.462661
C	−1.775836	−0.270518	−0.094455
C	−0.941174	−1.418230	−0.523167
C	0.391103	−1.538756	−0.343648
C	1.281043	−0.501752	0.334514
C	−2.336129	2.197010	−0.592023
C	2.511532	1.356062	−1.051680
C	−1.691683	−2.524051	−1.225925
C	2.700884	−1.113437	0.426524
H	−2.408647	0.807204	1.721085
H	−0.622990	0.331977	3.145913
H	1.523417	−0.429589	2.568661
H	0.144285	2.412978	−1.229467
H	−2.830735	−0.430413	−0.315615
H	0.887301	−2.430720	−0.718806
H	−2.387935	2.267475	−1.684028
H	−3.337450	1.940223	−0.228607
H	−2.081412	3.184129	−0.190961
H	2.352061	2.294059	−1.595049
H	3.242472	1.565145	−0.264578
H	2.941752	0.645372	−1.763964
H	−2.459278	−2.939262	−0.564277
H	−1.037872	−3.347165	−1.532912
H	−2.180852	−2.135874	−2.125624
H	2.696364	−2.049877	1.000385
H	3.405694	−0.438502	0.927143
H	3.109605	−1.350492	−0.563237

Me_4BV (000 101 011 0)

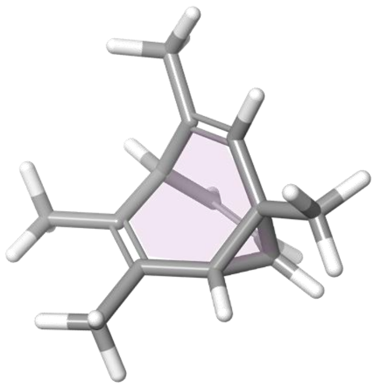

Table S90. Coordinates for the optimised geometry of **Me_4BV (000 101 011 0).**

	Coordinates / Å		
**Atom**	**x**	**y**	**z**
C	−1.249379	0.200114	1.623157
C	0.032826	0.261091	2.338780
C	1.258879	0.249192	1.794126
C	−1.497913	0.901333	0.292139
C	−0.409277	1.664307	−0.380950
C	0.910558	1.389487	−0.404897
C	−1.440985	−0.618304	0.368001
C	−0.360120	−1.433610	−0.267190
C	0.962681	−1.119184	−0.289305
C	1.490907	0.181903	0.307103
C	−2.873636	1.510908	0.103429
C	1.887546	2.258801	−1.142269
C	−0.883413	−2.687408	−0.940367
C	2.044831	−1.987595	−0.880349
H	−2.087029	0.182504	2.318223
H	−0.032730	0.316312	3.423401
H	2.133240	0.294977	2.435292
H	−0.738751	2.551268	−0.920723
H	−2.404460	−1.126864	0.327505
H	2.581538	0.208687	0.172533
H	−2.900126	2.528175	0.509115
H	−3.130287	1.553681	−0.960729
H	−3.654187	0.932018	0.609625
H	2.633106	2.663238	−0.450000
H	1.398916	3.105130	−1.636176
H	2.405076	1.678054	−1.912769
H	−0.952569	−3.503156	−0.213955
H	−1.881930	−2.516787	−1.359027
H	−0.267035	−3.009550	−1.783718
H	1.717992	−3.003197	−1.113144
H	2.872545	−2.093300	−0.169899
H	2.433188	−1.532224	−1.796962

Me_4BV (000 101 100 1)

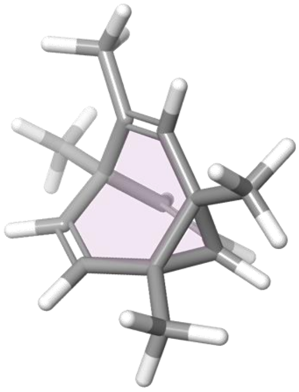

Table S91. Coordinates for the optimised geometry of **Me_4BV (000 101 100 1).**

	Coordinates / Å		
**Atom**	**x**	**y**	**z**
C	−1.437062	−0.090043	1.011116
C	−0.339954	−0.433257	1.928007
C	0.949896	−0.650246	1.624090
C	−1.247918	0.834086	−0.181696
C	0.107076	1.377126	−0.519124
C	1.333035	0.827375	−0.360544
C	−1.563605	−0.667289	−0.390473
C	−0.514785	−1.576716	−0.935270
C	0.806603	−1.579896	−0.691253
C	1.517850	−0.585441	0.213559
C	−2.352332	1.854034	−0.416638
C	2.550745	1.611149	−0.776883
C	−2.963686	−1.072222	−0.823812
C	2.998678	−1.027618	0.301872
H	−2.368869	0.027791	1.561751
H	−0.618356	−0.508704	2.977651
H	1.633266	−0.880419	2.437296
H	0.080525	2.376694	−0.953397
H	−0.870677	−2.340498	−1.626126
H	1.413991	−2.325420	−1.198215
H	−2.506041	2.003185	−1.490831
H	−3.308319	1.559691	0.026395
H	−2.086091	2.818704	0.030788
H	3.216127	1.773271	0.076673
H	2.293584	2.600279	−1.171811
H	3.097763	1.085959	−1.565857
H	−3.750401	−0.563373	−0.258960
H	−3.117183	−2.146987	−0.672174
H	−3.109104	−0.850617	−1.886604
H	3.583587	−0.368525	0.954870
H	3.482635	−1.039392	−0.682248
H	3.089021	−2.042679	0.711382

Me_4BV (000 101 101 0)

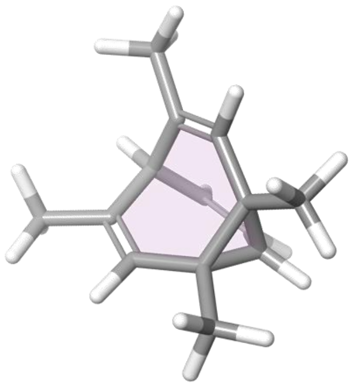

Table S92. Coordinates for the optimised geometry of **Me_4BV (000 101 101 0).**

Atom	Coordinates / Å		
	x	y	z
C	1.232793	−0.067350	1.341562
C	0.083079	0.269330	2.198725
C	−1.187859	0.468617	1.815302
C	1.127830	−0.998944	0.141201
C	−0.194363	−1.568710	−0.272316
C	−1.423587	−1.021514	−0.176052
C	1.423591	0.510105	−0.054824
C	0.389340	1.409492	−0.659184
C	−0.949006	1.399924	−0.490597
C	−1.639574	0.370889	0.381424
C	2.262956	−1.997099	−0.030043
C	−2.653510	−1.747263	−0.639425
C	2.838578	0.939878	−0.411551
C	−1.842169	2.392406	−1.177163
H	2.135588	−0.166287	1.942050
H	0.302263	0.364288	3.260432
H	−1.940382	0.712631	2.558368
H	−0.139894	−2.567150	−0.705405
H	0.789814	2.176461	−1.321600
H	−2.717878	0.582344	0.382326
H	3.185303	−1.686152	0.469362
H	2.482510	−2.140279	−1.093547
H	1.989045	−2.967942	0.399012
H	−3.351854	−1.879206	0.193392
H	−2.423267	−2.739823	−1.040363
H	−3.155081	−1.178013	−1.428829
H	3.602374	0.441850	0.192933
H	3.044016	0.724690	−1.465703
H	2.965928	2.016366	−0.248433
H	−1.279348	3.096740	−1.798521
H	−2.400474	2.974950	−0.437154
H	−2.556764	1.874771	−1.825380

Me_4BV (000 101 110 0)

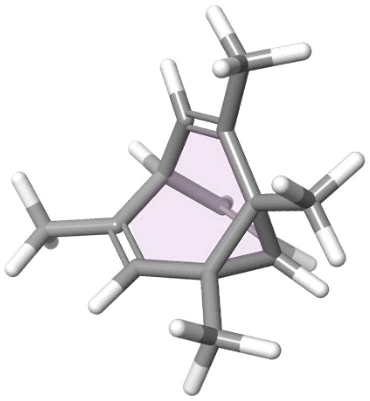

Table S93. Coordinates for the optimised geometry of **Me_4BV (000 101 110 0).**

	Coordinates / Å		
**Atom**	**x**	**y**	**z**
C	0.546144	−1.011947	−1.203261
C	−0.534632	−0.615975	−2.120279
C	−1.549339	0.223903	−1.867517
C	0.347772	−1.222766	0.291073
C	−0.986770	−0.967302	0.922780
C	−1.931464	−0.067553	0.581501
C	1.225345	−0.048097	−0.234183
C	0.727421	1.382990	−0.075187
C	−0.546683	1.805931	−0.234623
C	−1.717480	0.916064	−0.546078
C	1.037449	−2.438324	0.895270
C	−3.235243	0.036496	1.317924
C	2.743818	−0.201770	−0.173760
C	1.739897	2.436217	0.329858
H	1.200455	−1.730454	−1.695260
H	−0.497756	−1.065685	−3.110630
H	−2.286315	0.426362	−2.637881
H	−1.218716	−1.616339	1.766852
H	−0.794486	2.853952	−0.088601
H	−2.602210	1.561455	−0.631361
H	0.333464	−3.273182	0.991846
H	1.422298	−2.196133	1.891714
H	1.868715	−2.807240	0.287658
H	−4.071912	−0.131562	0.632048
H	−3.342330	1.031521	1.761949
H	−3.315044	−0.698527	2.125592
H	3.110207	−0.011210	0.840210
H	3.233828	0.488337	−0.868664
H	3.084062	−1.200972	−0.462126
H	1.277778	3.411542	0.520344
H	2.481636	2.584954	−0.460870
H	2.250091	2.145312	1.253663

Me_4BV (000 110 001 1)

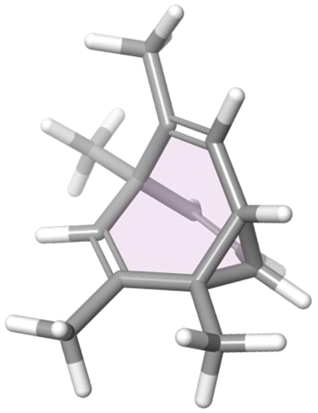

Table S94. Coordinates for the optimised geometry of **Me_4BV (000 110 001 1).**

	Coordinates / Å		
**Atom**	**x**	**y**	**z**
C	1.203167	−0.066233	−1.651668
C	0.150504	−1.041889	−1.961456
C	−0.972738	−1.280012	−1.265746
C	1.693642	0.229673	−0.237984
C	1.061882	−0.462383	0.949628
C	−0.241825	−0.803904	1.070286
C	0.897832	1.269134	−1.019590
C	−0.468867	1.689148	−0.664254
C	−1.495691	0.937676	−0.209812
C	−1.333214	−0.571408	0.032180
C	3.185018	0.517753	−0.131744
C	1.965468	−0.798345	2.119389
C	−2.820713	1.593016	0.076471
C	−2.622793	−1.243914	0.565475
H	1.958176	−0.043926	−2.435657
H	0.301833	−1.619933	−2.871153
H	−1.662042	−2.028112	−1.648130
H	−0.565874	−1.286744	1.989850
H	1.468230	2.106523	−1.418686
H	−0.648887	2.753951	−0.805434
H	3.395989	1.176288	0.717096
H	3.568705	1.020360	−1.027514
H	3.754618	−0.410475	−0.019282
H	2.765885	−1.475778	1.805467
H	1.430678	−1.296672	2.935733
H	2.407370	0.111076	2.538645
H	−3.609841	1.156699	−0.543552
H	−3.087995	1.481176	1.131710
H	−2.805899	2.667721	−0.136516
H	−3.456853	−1.145228	−0.139917
H	−2.474022	−2.319214	0.732526
H	−2.941744	−0.816024	1.523638

Me_4BV (000 110 010 1)

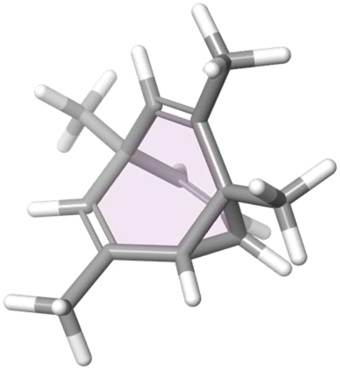

Table S95. Coordinates for the optimised geometry of **Me_4BV (000 110 010 1).**

	Coordinates / Å		
**Atom**	**x**	**y**	**z**
C	−0.687556	0.861134	−1.480164
C	0.226588	−0.020289	−2.220689
C	1.137276	−0.865053	−1.710689
C	−1.384154	0.458324	−0.182183
C	−1.112807	−0.896678	0.439451
C	0.068242	−1.557188	0.422434
C	−0.301103	1.534862	−0.185025
C	1.030592	1.366891	0.447228
C	1.776970	0.242856	0.432402
C	1.347066	−1.056399	−0.222233
C	−2.804470	0.988093	−0.034368
C	−2.265332	−1.580814	1.146979
C	1.560986	2.591726	1.153006
C	2.457714	−2.107921	−0.013910
H	−1.268601	1.478568	−2.163339
H	0.151996	0.033091	−3.305159
H	1.745364	−1.448641	−2.396037
H	0.132363	−2.528564	0.907460
H	−0.659556	2.559939	−0.091030
H	2.743560	0.244281	0.929701
H	−2.926560	1.968312	−0.510252
H	−3.524155	0.312934	−0.508601
H	−3.066574	1.112384	1.021346
H	−2.625886	−0.966947	1.978403
H	−3.090031	−1.764876	0.451241
H	−1.983531	−2.552076	1.568910
H	0.873017	2.896121	1.948908
H	1.663286	3.421281	0.445380
H	2.541814	2.422069	1.609577
H	3.405659	−1.786950	−0.464321
H	2.647094	−2.288919	1.051868
H	2.190729	−3.071552	−0.466296

Me$_4$BV (000 110 011 0)

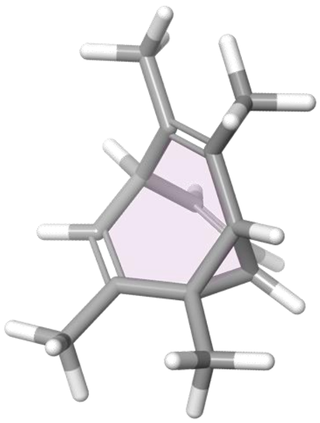

Table S96. Coordinates for the optimised geometry of **Me$_4$BV (000 110 011 0).**

	Coordinates / Å		
**Atom**	**x**	**y**	**z**
C	0.550913	1.568865	−0.866456
C	0.039715	1.027929	−2.133094
C	−0.533289	−0.169589	−2.322523
C	1.292081	0.738262	0.181345
C	1.496368	−0.746630	−0.026081
C	0.623194	−1.579699	−0.633633
C	−0.104701	1.285581	0.463725
C	−1.320347	0.432101	0.630799
C	−1.637598	−0.652293	−0.124236
C	−0.703852	−1.164526	−1.209858
C	2.419375	1.473415	0.893655
C	2.781021	−1.358332	0.491569
C	−2.206108	0.881672	1.776235
C	−2.905480	−1.457012	0.008406
H	0.900571	2.591825	−0.997553
H	0.130635	1.681607	−2.998111
H	−0.882837	−0.452026	−3.310119
H	0.857255	−2.636424	−0.730791
H	−0.142470	2.157724	1.117395
H	−1.140298	−2.066680	−1.660694
H	2.203492	2.542490	1.005872
H	3.354003	1.395281	0.328779
H	2.571464	1.072011	1.900868
H	2.862789	−1.224249	1.574812
H	3.648846	−0.900879	0.006104
H	2.839995	−2.435507	0.299041
H	−2.792153	0.067666	2.210698
H	−2.884856	1.671344	1.439097
H	−1.602277	1.278057	2.600738
H	−3.357292	−1.612542	−0.977865
H	−2.685628	−2.436397	0.445119
H	−3.672530	−0.973046	0.616755

Me$_4$BV (000 110 100 1)

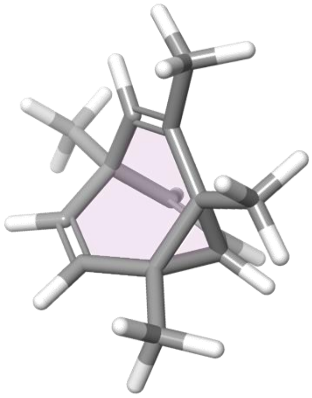

Table S97. Coordinates for the optimised geometry of **Me$_4$BV (000 110 100 1).**

Atom	Coordinates / Å		
	x	y	z
C	−0.902816	−0.801899	1.116499
C	0.362442	−1.094477	1.806648
C	1.610515	−0.908800	1.349634
C	−1.118410	0.433502	0.249142
C	0.031726	1.386360	−0.052305
C	1.343000	1.063383	−0.141555
C	−1.112987	−0.991298	−0.378290
C	0.017016	−1.441905	−1.245288
C	1.332134	−1.202281	−1.108119
C	1.916402	−0.333314	−0.015264
C	−2.467272	1.115117	0.471835
C	−0.318735	2.840282	−0.308137
C	−2.437055	−1.612405	−0.799551
C	3.447634	−0.270156	−0.193757
H	−1.741724	−1.092114	1.747747
H	0.261003	−1.512631	2.806568
H	2.443420	−1.180111	1.992180
H	2.061603	1.847951	−0.367889
H	−0.264493	−2.059601	−2.097657
H	2.012621	−1.626546	−1.841386
H	−2.844946	1.537448	−0.465002
H	−3.232601	0.433166	0.854708
H	−2.383320	1.912364	1.217874
H	−1.057772	2.922795	−1.111496
H	0.548284	3.434378	−0.618543
H	−0.716011	3.308069	0.597902
H	−2.800961	−1.137108	−1.716801
H	−3.215398	−1.524056	−0.036184
H	−2.316340	−2.684814	−0.992653
H	3.903011	−1.265869	−0.118573
H	3.723774	0.141747	−1.172845
H	3.916256	0.362821	0.570558

Me$_4$BV (000 110 101 0)

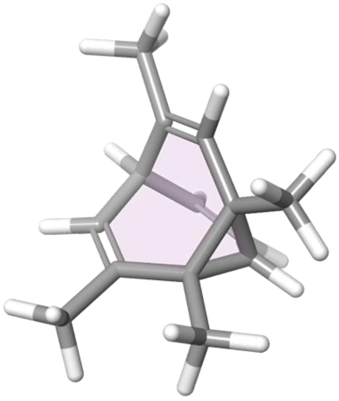

Table S98. Coordinates for the optimised geometry of **Me$_4$BV (000 110 101 0).**

	Coordinates / Å		
**Atom**	**x**	**y**	**z**
C	−0.884710	1.022067	0.961657
C	−0.118884	0.653578	2.163122
C	0.932111	−0.176554	2.235265
C	−1.240302	0.035481	−0.147231
C	−0.705904	−1.391136	−0.127206
C	0.468093	−1.795047	0.407795
C	−0.256601	1.213771	−0.411499
C	1.206874	0.966625	−0.617604
C	2.016433	0.084333	0.002589
C	1.487806	−0.885545	1.034393
C	−2.674778	0.168418	−0.654806
C	−1.545408	−2.463252	−0.793730
C	−0.747602	2.411328	−1.213411
C	3.480121	−0.013346	−0.315311
H	−1.660811	1.739026	1.226376
H	−0.449605	1.117079	3.090583
H	1.411032	−0.358182	3.191942
H	0.756517	−2.841748	0.359781
H	1.671880	1.606327	−1.367307
H	2.313402	−1.518023	1.387821
H	−3.342095	−0.517887	−0.122899
H	−3.093073	1.167166	−0.497137
H	−2.724581	−0.041583	−1.728170
H	−2.485955	−2.610185	−0.253979
H	−1.039688	−3.435118	−0.822400
H	−1.763128	−2.192450	−1.831807
H	−0.053296	3.253823	−1.113166
H	−1.723997	2.778121	−0.884154
H	−0.819764	2.149711	−2.274550
H	4.076157	0.175548	0.583437
H	3.788310	0.710871	−1.076548
H	3.721444	−1.013221	−0.690430

Me_4BV (000 110 110 0)

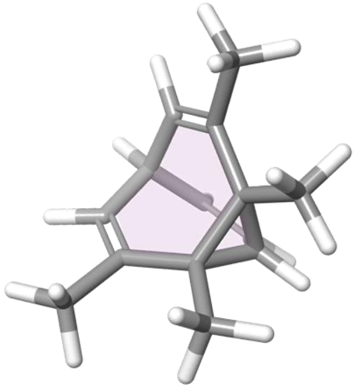

Table S99. Coordinates for the optimised geometry of **Me_4BV (000 110 110 0).**

	Coordinates / Å		
**Atom**	**x**	**y**	**z**
C	−0.213221	−0.021712	−1.544550
C	−0.347468	1.401540	−1.886464
C	−0.294938	2.446691	−1.049692
C	0.756710	−0.564145	−0.500150
C	1.587304	0.383952	0.357250
C	1.239705	1.636603	0.727110
C	−0.791265	−0.658495	−0.285128
C	−1.464630	0.197934	0.781183
C	−1.181490	1.489028	1.063432
C	−0.080448	2.279464	0.421033
C	1.501515	−1.824006	−0.941265
C	2.919654	−0.122560	0.874416
C	−1.472240	−2.005251	−0.528201
C	−2.536249	−0.455100	1.632268
H	−0.303252	−0.616076	−2.453507
H	−0.507170	1.618439	−2.940964
H	−0.409783	3.454786	−1.434083
H	1.909704	2.229762	1.343662
H	−1.724594	2.008244	1.848496
H	−0.078673	3.280133	0.872902
H	2.473308	−1.570954	−1.378149
H	0.973308	−2.387288	−1.715994
H	1.653926	−2.497226	−0.091423
H	3.625728	−0.267841	0.051131
H	3.389208	0.574747	1.577591
H	2.790509	−1.067972	1.411010
H	−1.306829	−2.677667	0.319842
H	−1.111036	−2.514344	−1.426448
H	−2.549102	−1.877052	−0.680555
H	−3.416822	−0.697082	1.029375
H	−2.876818	0.192827	2.447974
H	−2.154547	−1.369379	2.097897

Me_4BV (000 111 001 0)

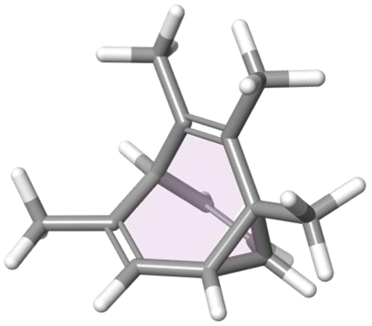

Table S100. Coordinates for the optimised geometry of **Me_4BV (000 111 001 0).**

	Coordinates / Å		
**Atom**	**x**	**y**	**z**
C	−0.259742	−1.629580	1.292648
C	0.490257	−0.747565	2.196136
C	1.169329	0.358172	1.857026
C	−1.103166	−1.114147	0.130422
C	−1.178407	0.389497	−0.162414
C	−0.143147	1.274184	−0.070226
C	0.180115	−1.899595	−0.126791
C	1.391639	−1.316195	−0.722834
C	1.910537	−0.095069	−0.497961
C	1.237064	0.878206	0.444090
C	−2.372496	−1.917730	−0.137924
C	−2.559056	0.895597	−0.556905
C	−0.215045	2.731675	−0.464783
C	3.163359	0.377999	−1.173749
H	−0.655558	−2.492278	1.825790
H	0.492842	−1.035260	3.245384
H	1.688121	0.925547	2.623142
H	0.055521	−2.931427	−0.452269
H	1.914664	−1.969349	−1.419092
H	1.863385	1.779800	0.501380
H	−3.171219	−1.629138	0.552721
H	−2.713329	−1.780875	−1.169283
H	−2.212437	−2.993372	0.003382
H	−3.323623	0.514812	0.127320
H	−2.801967	0.580525	−1.576516
H	−2.668208	1.980838	−0.504085
H	0.706907	3.035645	−0.974108
H	−0.338134	3.357754	0.424290
H	−1.016533	2.955270	−1.172244
H	2.958606	1.274036	−1.768623
H	3.581923	−0.378824	−1.845334
H	3.927795	0.620847	−0.428588

Me$_4$BV (000 111 010 0)

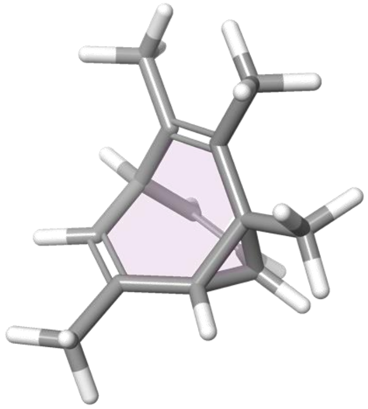

Table S101. Coordinates for the optimised geometry of **Me$_4$BV (000 111 010 0).**

	Coordinates / Å		
**Atom**	**x**	**y**	**z**
C	−1.251077	−0.430532	−1.374503
C	−0.813726	0.757562	−2.117780
C	−0.051227	1.758456	−1.656172
C	−0.366986	−1.184989	−0.382955
C	1.044399	−0.689078	−0.042925
C	1.450262	0.613796	−0.024845
C	−1.591741	−0.411227	0.097420
C	−1.509622	0.811417	0.930205
C	−0.599914	1.793866	0.781386
C	0.490594	1.772455	−0.256577
C	−0.553079	−2.699229	−0.434124
C	2.033549	−1.783383	0.333770
C	2.868716	1.075886	0.216866
C	−2.548114	0.923017	2.018554
H	−1.915565	−1.038777	−1.986483
H	−1.154305	0.823131	−3.149088
H	0.207122	2.587209	−2.307040
H	−2.454840	−1.020547	0.365332
H	−0.613327	2.656219	1.441465
H	1.032951	2.721207	−0.141848
H	0.156506	−3.158885	−1.129672
H	−0.430676	−3.141879	0.559510
H	−1.557382	−2.976175	−0.776554
H	2.334031	−2.346266	−0.555472
H	1.593715	−2.471922	1.061922
H	2.938769	−1.417625	0.822814
H	3.620441	0.311737	0.007533
H	3.117548	1.910176	−0.449302
H	2.983778	1.416381	1.250518
H	−2.439270	1.838817	2.609137
H	−2.468362	0.073650	2.705261
H	−3.553171	0.925532	1.583648

Me_4BV (000 111 100 0)

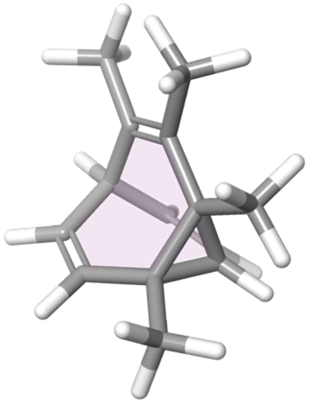

Table S102. Coordinates for the optimised geometry of **Me_4BV (000 111 100 0).**

Atom	Coordinates / Å x	y	z
C	−1.358586	0.192466	−1.075162
C	−0.685994	1.247159	−1.845355
C	0.371615	1.977236	−1.467133
C	−0.608892	−0.827176	−0.222599
C	0.906371	−0.723707	0.030294
C	1.655162	0.416158	0.001208
C	−1.557604	0.215874	0.432675
C	−1.008743	1.344079	1.240062
C	0.099512	2.065368	1.009748
C	1.035427	1.796932	−0.136034
C	−1.125889	−2.253863	−0.424902
C	1.585776	−2.034688	0.404705
C	3.159297	0.463247	0.137235
C	−2.910494	−0.252583	0.946279
H	−2.206171	−0.186174	−1.645302
H	−1.099306	1.445910	−2.832298
H	0.780022	2.725683	−2.138227
H	−1.586106	1.621337	2.121133
H	0.368395	2.864600	1.693435
H	1.823658	2.559505	−0.070849
H	−2.131150	−2.284079	−0.857868
H	−1.170549	−2.788257	0.529473
H	−0.486673	−2.807851	−1.120570
H	1.776492	−2.635943	−0.489492
H	2.531641	−1.899932	0.935908
H	0.967691	−2.611494	1.099908
H	3.582256	1.184728	−0.571396
H	3.647647	−0.486863	−0.092596
H	3.438523	0.771079	1.149651
H	−3.431167	−0.912166	0.246122
H	−3.573596	0.603123	1.118852
H	−2.788563	−0.789707	1.893096

Me_4BV (001 001 001 1)

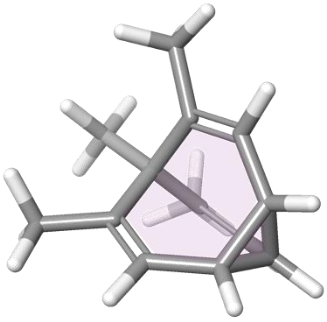

Table S103. Coordinates for the optimised geometry of **Me_4BV (001 001 001 1).**

	Coordinates / Å		
**Atom**	**x**	**y**	**z**
C	2.257814	0.506948	−1.089075
C	1.247567	1.548246	−0.869583
C	0.020799	1.438398	−0.312809
C	1.889634	−0.903606	−1.467513
C	0.496369	−1.329680	−1.641720
C	−0.601881	−0.947149	−0.952852
C	2.479391	−0.617240	−0.111556
C	1.699642	−0.745427	1.124824
C	0.395526	−0.462861	1.340372
C	−0.543475	0.083150	0.218818
C	−0.821082	2.687543	−0.210429
C	−1.927688	−1.551948	−1.347904
C	−0.155136	−0.691304	2.727525
C	−1.976357	0.302379	0.795735
H	3.155657	0.899028	−1.562391
H	1.559817	2.533354	−1.213434
H	2.558792	−1.387670	−2.175883
H	0.362476	−2.053795	−2.444156
H	3.514865	−0.923431	0.022301
H	2.280383	−1.122554	1.965470
H	−1.751798	2.577068	−0.774944
H	−0.311200	3.567924	−0.618092
H	−1.051943	2.916861	0.834192
H	−1.831777	−2.257517	−2.181096
H	−2.364295	−2.110858	−0.514775
H	−2.627221	−0.776764	−1.674792
H	−0.525123	0.243891	3.158376
H	−0.962029	−1.430023	2.709269
H	0.603866	−1.074904	3.418904
H	−1.984620	1.020628	1.623645
H	−2.672751	0.686515	0.041509
H	−2.414223	−0.625202	1.182064

Me_4BV (001 001 010 1)

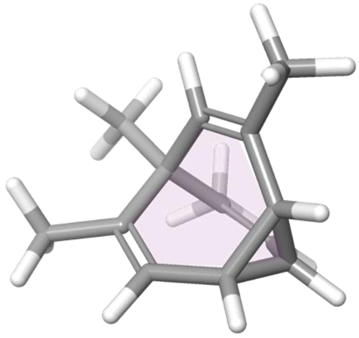

Table S104. Coordinates for the optimised geometry of **Me_4BV (001 001 010 1).**

	Coordinates / Å		
**Atom**	**x**	**y**	**z**
C	1.298782	0.136360	1.887423
C	0.319223	1.202445	1.628996
C	−0.570225	1.306260	0.616319
C	1.002214	−1.314195	1.592563
C	−0.284378	−1.749847	1.028873
C	−1.066654	−1.121832	0.122753
C	2.043938	−0.577935	0.787349
C	1.853097	−0.248752	−0.640109
C	0.685547	0.106804	−1.214944
C	−0.666961	0.238137	−0.500687
C	−1.507601	2.486411	0.592727
C	−2.375407	−1.758109	−0.270062
C	3.099950	−0.331483	−1.487186
C	−1.661685	0.668205	−1.615920
H	1.871814	0.321150	2.793881
H	0.325495	1.992171	2.378819
H	1.391693	−2.027194	2.316523
H	−0.634274	−2.702174	1.424586
H	3.074371	−0.837210	1.026435
H	0.689242	0.322797	−2.281220
H	−2.549223	2.152320	0.626254
H	−1.349850	3.091488	−0.305087
H	−1.360670	3.150389	1.451998
H	−3.213073	−1.094642	−0.033744
H	−2.389377	−1.992870	−1.338597
H	−2.556422	−2.698176	0.263143
H	3.859391	0.361665	−1.110081
H	3.510149	−1.346497	−1.457316
H	2.913330	−0.080015	−2.536533
H	−1.362687	1.615792	−2.082246
H	−2.682044	0.800322	−1.239620
H	−1.707707	−0.071785	−2.425289

Me$_4$BV (001 001 011 0)

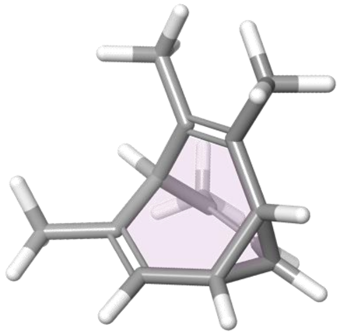

Table S105. Coordinates for the optimised geometry of **Me$_4$BV (001 001 011 0).**

Atom	Coordinates / Å		
	x	y	z
C	−0.590548	−2.219697	0.072839
C	0.509738	−1.920264	−0.856627
C	1.166989	−0.751775	−0.992474
C	−0.657752	−1.639999	1.468384
C	0.373063	−0.738275	2.004976
C	1.058085	0.208339	1.334388
C	−1.655646	−1.205866	0.420031
C	−1.690477	0.168261	−0.163730
C	−0.604600	0.949207	−0.409568
C	0.821233	0.463181	−0.144832
C	2.277005	−0.577305	−1.987354
C	2.090442	1.067042	2.004000
C	−3.092740	0.635952	−0.499229
C	−0.663507	2.359859	−0.940606
H	−0.937061	−3.246598	−0.023656
H	0.806250	−2.750491	−1.494779
H	−1.046414	−2.310521	2.232380
H	0.588318	−0.870626	3.063582
H	−2.652159	−1.623513	0.561573
H	1.508448	1.266366	−0.448110
H	2.026578	0.215505	−2.699707
H	2.467068	−1.491112	−2.559918
H	3.206038	−0.306311	−1.475341
H	2.203064	0.826295	3.066235
H	3.065954	0.929326	1.526406
H	1.811277	2.123107	1.930002
H	−3.119657	1.340649	−1.334528
H	−3.553870	1.100724	0.377841
H	−3.721514	−0.206410	−0.809597
H	−0.286661	2.390305	−1.967950
H	−0.041861	3.019910	−0.324996
H	−1.665083	2.794732	−0.929633

Me_4BV (001 001 100 1)

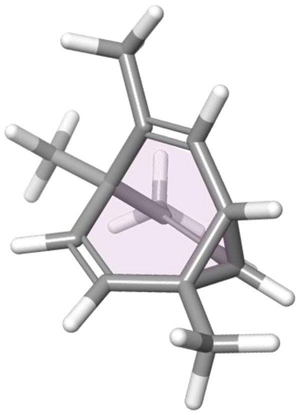

Table S106. Coordinates for the optimised geometry of **Me_4BV (001 001 100 1).**

Atom	Coordinates / Å		
	x	y	z
C	1.603766	0.911519	−0.898601
C	0.248091	1.371450	−1.243827
C	−0.942328	0.953044	−0.758471
C	1.899989	−0.529505	−0.565762
C	0.851400	−1.563444	−0.565949
C	−0.446268	−1.460124	−0.201098
C	2.025656	0.534647	0.511949
C	1.043547	0.588862	1.620742
C	−0.269738	0.309455	1.579860
C	−1.043143	−0.136916	0.335615
C	−2.206007	1.556009	−1.316728
C	−1.337819	−2.667434	−0.341238
C	3.434482	0.918011	0.917874
C	−2.506000	−0.324333	0.826123
H	2.356734	1.409780	−1.506709
H	0.234583	2.143068	−2.012376
H	2.834555	−0.914658	−0.969823
H	1.195095	−2.529509	−0.933146
H	1.440165	0.893886	2.588356
H	−0.826733	0.409481	2.508639
H	−2.839003	0.781615	−1.761001
H	−2.002963	2.289370	−2.105224
H	−2.766745	2.078157	−0.535694
H	−1.727073	−2.979559	0.632485
H	−0.806897	−3.529072	−0.761324
H	−2.174063	−2.453045	−1.013905
H	3.804954	0.243652	1.697705
H	4.134136	0.867392	0.076096
H	3.455732	1.942512	1.305291
H	−3.178494	−0.647841	0.024052
H	−2.572352	−1.072151	1.626820
H	−2.917261	0.605681	1.239267

Me_4BV (001 001 101 0)

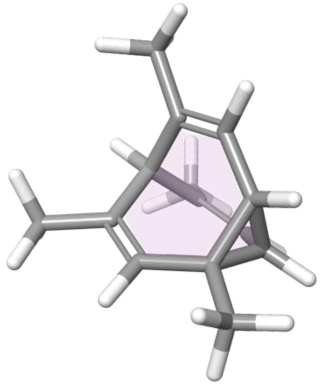

Table S107. Coordinates for the optimised geometry of **Me_4BV (001 001 101 0).**

	Coordinates / Å		
**Atom**	**x**	**y**	**z**
C	1.714959	0.575359	−0.946422
C	0.590643	1.510451	−1.136238
C	−0.684380	1.362308	−0.723469
C	1.536426	−0.924899	−0.975599
C	0.225001	−1.562159	−1.195993
C	−0.981559	−1.134978	−0.772035
C	1.915326	−0.234314	0.328432
C	0.933213	−0.139204	1.447487
C	−0.404664	0.021362	1.377757
C	−1.137147	0.134337	0.050583
C	−1.732558	2.401393	−0.994945
C	−2.242996	−1.887979	−1.078366
C	3.348580	−0.413837	0.789310
C	−1.257274	0.098840	2.611240
H	2.618935	0.942468	−1.428999
H	0.845378	2.417735	−1.681057
H	2.331248	−1.475048	−1.476018
H	0.262269	−2.482309	−1.776352
H	1.369503	−0.210484	2.443130
H	−2.207219	0.257395	0.270692
H	−2.142993	2.781077	−0.053627
H	−1.334243	3.255302	−1.552754
H	−2.549202	1.971140	−1.583730
H	−2.735349	−2.196572	−0.150435
H	−2.933215	−1.255913	−1.646507
H	−2.053572	−2.789477	−1.670301
H	3.659263	0.432761	1.411278
H	3.449142	−1.332887	1.376958
H	4.046762	−0.480561	−0.052540
H	−0.667391	0.010775	3.529511
H	−1.996502	−0.708802	2.612335
H	−1.786384	1.056719	2.646675

Me$_4$BV (001 001 110 0)

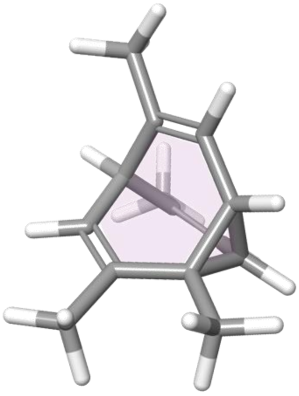

Table S108. Coordinates for the optimised geometry of **Me$_4$BV (001 001 110 0).**

	Coordinates / Å		
**Atom**	**x**	**y**	**z**
C	0.625917	−0.587530	−1.589524
C	−0.676159	−1.235258	−1.349237
C	−1.560970	−0.968511	−0.368433
C	0.867425	0.878670	−1.318735
C	−0.180782	1.772200	−0.793798
C	−1.159387	1.469526	0.081840
C	1.586830	−0.173279	−0.477016
C	1.202834	−0.378070	0.974316
C	−0.045513	−0.266093	1.481366
C	−1.277280	0.083834	0.685232
C	−2.866916	−1.697622	−0.252355
C	−2.178881	2.479480	0.519100
C	3.061251	−0.354632	−0.810065
C	2.306419	−0.739356	1.946303
H	1.085010	−0.968233	−2.500518
H	−0.935846	−2.008809	−2.069734
H	1.473453	1.390002	−2.064978
H	−0.145758	2.787861	−1.183851
H	−0.218034	−0.433413	2.541199
H	−2.113753	0.089946	1.398164
H	−2.998756	−2.439023	−1.047293
H	−2.924330	−2.223505	0.706166
H	−3.701651	−0.991843	−0.313939
H	−2.028257	3.452924	0.040872
H	−2.125065	2.628871	1.602336
H	−3.186493	2.135712	0.263679
H	3.677169	0.361320	−0.256206
H	3.262342	−0.191010	−1.875398
H	3.391677	−1.371921	−0.576302
H	2.780029	−1.683180	1.658280
H	1.938177	−0.867837	2.970378
H	3.065299	0.048778	1.978151

Me$_4$BV (001 010 010 1)

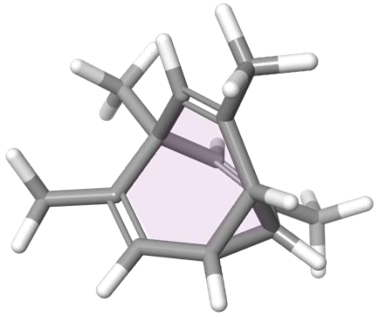

Table S109. Coordinates for the optimised geometry of **Me$_4$BV (001 010 010 1).**

	Coordinates / Å		
**Atom**	**x**	**y**	**z**
C	0.526976	−0.730819	−1.834974
C	0.107725	0.680515	−1.853366
C	−0.296860	1.452389	−0.820034
C	1.314969	−1.334446	−0.695764
C	1.732499	−0.543285	0.483983
C	1.013061	0.439000	1.066322
C	−0.114203	−1.762125	−0.935866
C	−1.221996	−1.427417	−0.012377
C	−1.355170	−0.269693	0.668457
C	−0.371177	0.895894	0.613573
C	−0.682565	2.885118	−1.076225
C	3.090857	−0.893877	1.040883
C	−2.273790	−2.499245	0.139613
C	−0.858206	1.944087	1.645455
H	0.799518	−1.084335	−2.827551
H	0.137923	1.134906	−2.842495
H	2.065036	−2.065069	−0.995947
H	1.438816	0.957425	1.922152
H	−0.224770	−2.750293	−1.380638
H	−2.226051	−0.139286	1.306450
H	−1.725622	3.061082	−0.795962
H	−0.586400	3.157194	−2.133255
H	−0.037783	3.566172	−0.512400
H	3.110810	−1.942868	1.354876
H	3.359778	−0.282694	1.908845
H	3.862201	−0.744505	0.277939
H	−3.074195	−2.208065	0.827935
H	−2.733234	−2.718174	−0.830102
H	−1.822254	−3.419090	0.526100
H	−0.901145	1.519452	2.657451
H	−1.865518	2.310208	1.412656
H	−0.189230	2.811845	1.694267

Me_4BV (001 010 011 0)

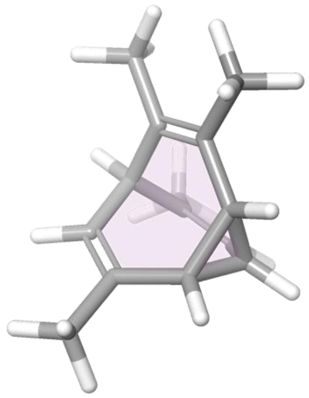

Table S1110. Coordinates for the optimised geometry of **Me_4BV (001 010 011 0).**

	Coordinates / Å		
**Atom**	**x**	**y**	**z**
C	0.130725	0.464819	−1.985999
C	−0.684153	−0.750605	−1.834528
C	−0.904069	−1.448769	−0.703604
C	1.416530	0.697008	−1.222083
C	1.962147	−0.283822	−0.255555
C	1.221611	−1.054717	0.565815
C	0.197058	1.539883	−0.925377
C	−0.552835	1.470812	0.364467
C	−0.808642	0.335785	1.068154
C	−0.287291	−1.025502	0.615783
C	−1.767555	−2.675175	−0.678439
C	3.465712	−0.392370	−0.222095
C	−1.008481	2.828766	0.859966
C	−1.628335	0.272276	2.332260
H	0.130592	0.824151	−3.013368
H	−1.153731	−1.102379	−2.751166
H	2.197024	1.205937	−1.786180
H	1.705782	−1.748477	1.246904
H	0.236782	2.552069	−1.327483
H	−0.568757	−1.771524	1.372775
H	−1.187271	−3.541127	−0.343782
H	−2.608971	−2.532518	0.007380
H	−2.178997	−2.911433	−1.665299
H	3.845059	−0.692501	−1.204584
H	3.816011	−1.128973	0.508660
H	3.908742	0.573849	0.041394
H	−0.278755	3.603412	0.597734
H	−1.105495	2.877629	1.947649
H	−1.966863	3.093572	0.402462
H	−2.191219	1.184897	2.539164
H	−0.980802	0.064835	3.190119
H	−2.371554	−0.529809	2.258856

Me_4BV (001 010 100 1)

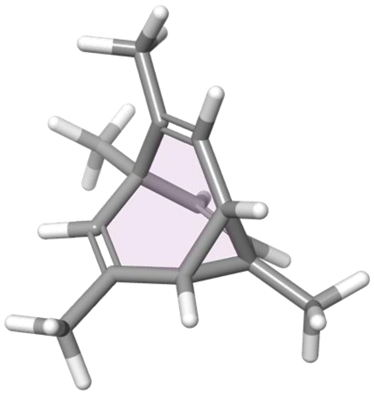

Table S111. Coordinates for the optimised geometry of **Me_4BV (001 010 100 1).**

Atom	Coordinates / Å		
	x	y	z
C	−1.046401	−1.084742	1.000895
C	0.383010	−1.181775	1.347908
C	1.442342	−0.590477	0.751630
C	−1.675985	0.186437	0.484832
C	−0.900327	1.435987	0.296723
C	0.384783	1.516581	−0.108192
C	−1.553961	−1.022376	−0.432925
C	−0.589767	−1.021956	−1.563282
C	0.630147	−0.460217	−1.613337
C	1.264582	0.324669	−0.472542
C	2.825765	−0.835402	1.293381
C	−1.653253	2.706352	0.611740
C	−2.837634	−1.774029	−0.725015
C	2.602022	0.894006	−1.006813
H	−1.659071	−1.650368	1.700915
H	0.580989	−1.820060	2.207825
H	−2.675693	0.383282	0.870994
H	0.843483	2.499188	−0.189488
H	−0.911848	−1.552529	−2.458428
H	1.205146	−0.576625	−2.528442
H	3.286657	0.103340	1.615648
H	3.459851	−1.307199	0.536530
H	2.818086	−1.501676	2.163198
H	−1.982013	2.701840	1.656383
H	−2.536340	2.790547	−0.030514
H	−1.045541	3.604447	0.458963
H	−2.622301	−2.821572	−0.962591
H	−3.525394	−1.762500	0.127912
H	−3.358675	−1.324038	−1.577152
H	3.117881	1.501472	−0.253319
H	3.287914	0.098880	−1.323398
H	2.441547	1.540516	−1.880041

Me$_4$BV (001 010 101 0)

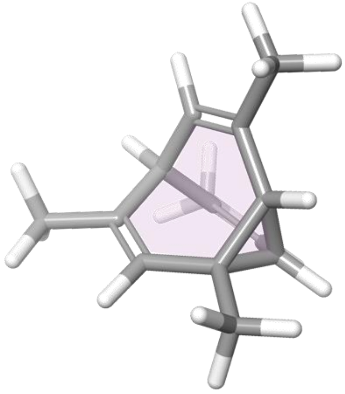

Table S112. Coordinates for the optimised geometry of **Me$_4$BV (001 010 101 0).**

	Coordinates / Å		
**Atom**	**x**	**y**	**z**
C	−0.999915	0.563938	1.380960
C	0.332742	1.182650	1.506728
C	1.373533	1.078523	0.656150
C	−1.697191	0.383396	0.051766
C	−1.097041	0.817987	−1.235180
C	0.215752	0.772201	−1.542030
C	−1.215949	−0.838905	0.825663
C	−0.059203	−1.645398	0.337550
C	1.055064	−1.217506	−0.291415
C	1.281952	0.248235	−0.610825
C	2.682640	1.765539	0.911509
C	−2.074872	1.351141	−2.252862
C	−2.291446	−1.667823	1.501135
C	2.140598	−2.164124	−0.714297
H	−1.648102	0.858271	2.204569
H	0.466075	1.790648	2.399713
H	−2.771362	0.564116	0.078868
H	0.561986	1.115842	−2.512406
H	−0.138707	−2.715583	0.524932
H	2.234436	0.339967	−1.151383
H	2.911796	2.460080	0.096827
H	3.490142	1.029394	0.980307
H	2.674093	2.338335	1.844683
H	−1.586217	1.654089	−3.184925
H	−2.817167	0.585095	−2.500851
H	−2.598131	2.225872	−1.852442
H	−3.126146	−1.051741	1.853739
H	−1.879663	−2.195355	2.368466
H	−2.697641	−2.408516	0.803657
H	1.912332	−3.201706	−0.449213
H	2.279941	−2.121117	−1.799373
H	3.085671	−1.897548	−0.230021

Me_4BV (001 010 110 0)

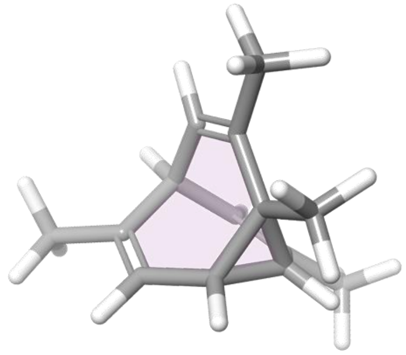

Table S113. Coordinates for the optimised geometry of **Me_4BV (001 010 110 0).**

**Atom**		**Coordinates / Å**	
	**x**	**y**	**z**
C	−0.043548	0.235068	−1.567327
C	1.316161	−0.315676	−1.425846
C	1.887038	−0.824806	−0.316686
C	−1.235477	−0.317856	−0.821225
C	−1.137714	−1.464611	0.116390
C	−0.088096	−1.731717	0.919394
C	−0.730121	1.089238	−0.502705
C	−0.030443	1.373450	0.811371
C	0.790099	0.521598	1.466279
C	1.140425	−0.865557	0.998663
C	3.285948	−1.365772	−0.313382
C	−2.331072	−2.387690	0.130881
C	−1.596562	2.214325	−1.052693
C	−0.262894	2.726368	1.450753
H	−0.239850	0.513256	−2.601734
H	1.905720	−0.305998	−2.340856
H	−2.150417	−0.364224	−1.411754
H	−0.101819	−2.601900	1.568887
H	1.249326	0.820859	2.404536
H	1.794302	−1.306766	1.763341
H	3.907705	−0.806220	0.392977
H	3.757913	−1.297331	−1.299062
H	3.284165	−2.419925	−0.017788
H	−2.213076	−3.214046	0.839782
H	−3.232953	−1.834320	0.413262
H	−2.485915	−2.821648	−0.862597
H	−2.066701	1.940062	−2.004475
H	−2.405685	2.459060	−0.356908
H	−0.997885	3.111520	−1.240661
H	0.077267	3.529219	0.789126
H	−1.324835	2.869778	1.673903
H	0.278994	2.842259	2.396155

Me_4BV (001 011 010 0)

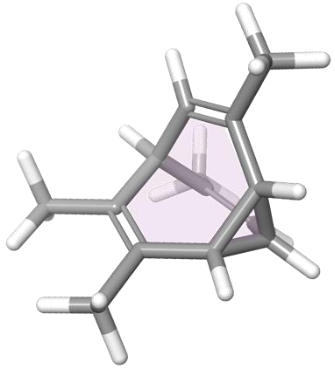

Table S114. Coordinates for the optimised geometry of **Me_4BV (001 011 010 0).**

	Coordinates / Å		
**Atom**	**x**	**y**	**z**
C	1.135291	1.084217	−1.308789
C	−0.283480	1.246051	−1.662520
C	−1.351288	0.796591	−0.975092
C	1.675626	−0.167665	−0.656170
C	0.827822	−1.348491	−0.313114
C	−0.464921	−1.296299	0.105204
C	1.641015	1.131035	0.116983
C	0.745892	1.344278	1.277666
C	−0.509148	0.864061	1.385303
C	−1.186454	0.030809	0.323661
C	−2.756892	1.020571	−1.448827
C	1.561309	−2.667295	−0.454261
C	−1.330789	−2.499993	0.379592
C	1.326545	2.165670	2.400949
H	1.789343	1.543047	−2.047908
H	−0.463926	1.789148	−2.588241
H	2.664204	−0.457179	−1.012095
H	2.609355	1.613765	0.242174
H	−1.096870	1.067623	2.275669
H	−2.190542	−0.183242	0.717106
H	−2.791711	1.574167	−2.392921
H	−3.321005	1.593714	−0.705942
H	−3.261217	0.061696	−1.606598
H	2.618862	−2.554462	−0.189319
H	1.504236	−3.020216	−1.488727
H	1.176783	−3.444846	0.210876
H	−1.507628	−2.598458	1.455365
H	−0.910027	−3.438674	0.013041
H	−2.299506	−2.388541	−0.120679
H	0.625652	2.291748	3.232905
H	2.227285	1.683591	2.795345
H	1.596183	3.163579	2.039365

Me_4BV (001 011 100 0)

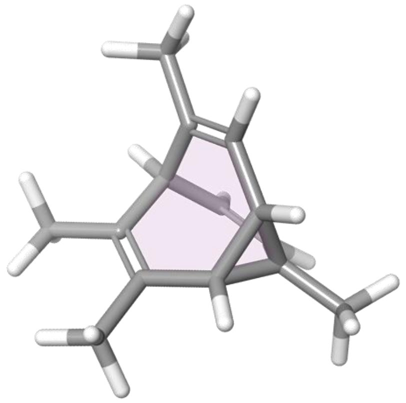

Table S115. Coordinates for the optimised geometry of **Me_4BV (001 011 100 0).**

	Coordinates / Å		
**Atom**	**x**	**y**	**z**
C	−0.271480	−1.327969	1.230603
C	1.128097	−0.889918	1.364526
C	1.825016	−0.099981	0.524880
C	−1.349266	−0.443162	0.651094
C	−1.100942	0.947002	0.158752
C	0.038226	1.385369	−0.440606
C	−0.916212	−1.694609	−0.101726
C	−0.145111	−1.580470	−1.367549
C	0.783117	−0.661376	−1.678315
C	1.201064	0.445747	−0.744272
C	3.250892	0.283736	0.789978
C	−2.284004	1.870407	0.374139
C	0.297413	2.810948	−0.858688
C	−1.885864	−2.859526	−0.080870
H	−0.577399	−1.924891	2.088265
H	1.633439	−1.253125	2.257559
H	−2.300740	−0.518810	1.177922
H	−0.361294	−2.329550	−2.127711
H	1.268039	−0.699384	−2.648799
H	1.973013	1.011297	−1.285279
H	3.627666	−0.145011	1.724435
H	3.895948	−0.068226	−0.021581
H	3.343313	1.372271	0.862372
H	−2.249172	2.297441	1.381290
H	−2.333393	2.681361	−0.357196
H	−3.227984	1.323780	0.266637
H	1.284815	3.132216	−0.508034
H	−0.415247	3.527844	−0.445370
H	0.275678	2.892763	−1.950044
H	−1.349967	−3.805795	−0.213174
H	−2.437097	−2.920303	0.864136
H	−2.620565	−2.760076	−0.887371

Me_4BV (001 100 010 1)

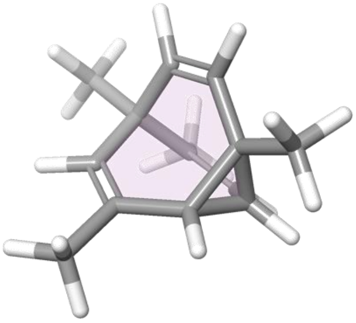

Table S116. Coordinates for the optimised geometry of **Me_4BV (001 100 010 1).**

	Coordinates / Å		
**Atom**	**x**	**y**	**z**
C	0.996238	−1.076156	1.059627
C	−0.444276	−1.383845	1.117022
C	−1.455162	−0.847734	0.397258
C	1.732157	−0.768719	−0.237004
C	0.989000	−0.733698	−1.523032
C	−0.269480	−0.316589	−1.743242
C	1.523605	0.316170	0.810812
C	0.626879	1.480353	0.613412
C	−0.566618	1.462341	−0.016826
C	−1.195556	0.233506	−0.666326
C	−2.864251	−1.321029	0.637675
C	3.138732	−1.320805	−0.358916
C	1.129841	2.778040	1.199074
C	−2.485324	0.707437	−1.380627
H	1.550045	−1.652676	1.798714
H	−0.698542	−2.147412	1.850762
H	1.532504	−1.101882	−2.392270
H	−0.654503	−0.379174	−2.757851
H	2.403651	0.576448	1.398231
H	−1.135923	2.386807	−0.078353
H	−2.915864	−2.089435	1.417067
H	−3.285153	−1.761021	−0.271572
H	−3.499904	−0.492574	0.964972
H	3.115475	−2.346399	−0.743323
H	3.659063	−1.340210	0.605195
H	3.732711	−0.705909	−1.043901
H	1.269005	2.675940	2.280551
H	2.090955	3.047730	0.748576
H	0.439438	3.611202	1.030514
H	−2.266439	1.476454	−2.133593
H	−3.203730	1.147144	−0.678183
H	−2.988574	−0.114304	−1.904440

Me_4BV (001 100 011 0)

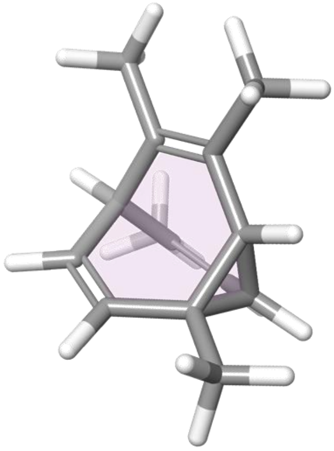

Table S117. Coordinates for the optimised geometry of **Me_4BV (001 100 011 0).**

	Coordinates / Å		
**Atom**	**x**	**y**	**z**
C	1.650446	0.175383	0.716571
C	0.906846	1.416290	0.991976
C	−0.252035	1.819506	0.435993
C	1.773696	−0.443013	−0.671810
C	1.090080	0.179115	−1.836094
C	−0.095999	0.808425	−1.846146
C	0.963471	−1.135479	0.416501
C	−0.522460	−1.297142	0.366566
C	−1.409265	−0.358886	−0.060461
C	−0.952673	0.985589	−0.618318
C	−0.914722	3.109313	0.820544
C	3.111949	−1.068073	−1.012497
C	−0.984415	−2.663068	0.835125
C	−2.908977	−0.514145	−0.028808
H	2.531156	0.098882	1.352169
H	1.362733	2.061396	1.740842
H	1.614204	0.114235	−2.788348
H	−0.476091	1.216168	−2.777655
H	1.445426	−1.993700	0.885279
H	−1.839140	1.541201	−0.955829
H	−1.916980	2.917042	1.217031
H	−1.004135	3.765003	−0.051625
H	−0.351677	3.650875	1.587802
H	3.795258	−0.312904	−1.416138
H	3.593228	−1.515480	−0.135692
H	2.985603	−1.858312	−1.760614
H	−0.257560	−3.435184	0.557673
H	−1.091828	−2.670440	1.924314
H	−1.927861	−2.975774	0.380088
H	−3.251889	−1.359099	0.572124
H	−3.370104	0.376276	0.413514
H	−3.296286	−0.633998	−1.045593

Me_4BV (001 100 100 1)

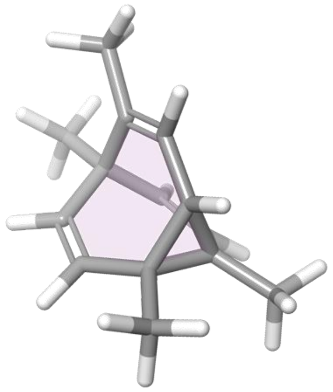

Table S118. Coordinates for the optimised geometry of **Me_4BV (001 100 100 1).**

	Coordinates / Å		
**Atom**	**x**	**y**	**z**
C	−1.040658	−1.155270	−0.090589
C	0.351175	−1.625707	−0.227991
C	1.500367	−0.914838	−0.200418
C	−1.538070	0.156719	−0.676806
C	−0.588027	1.071750	−1.373331
C	0.717521	1.275550	−1.128459
C	−1.453137	−0.040087	0.857363
C	−0.420416	0.683364	1.654306
C	0.853528	0.960394	1.328314
C	1.497065	0.609723	−0.004662
C	2.814603	−1.631656	−0.365128
C	−2.902229	0.118461	−1.347425
C	−2.736796	−0.264880	1.640826
C	2.915240	1.230190	−0.003580
H	−1.729630	−1.995795	−0.160270
H	0.432200	−2.702545	−0.370617
H	−1.008678	1.640016	−2.202444
H	1.249617	1.977319	−1.765723
H	−0.740580	1.018784	2.640353
H	1.461408	1.486559	2.059986
H	2.684235	−2.711617	−0.496455
H	3.346767	−1.265635	−1.248503
H	3.444596	−1.492347	0.518740
H	−2.794908	−0.008656	−2.430947
H	−3.527747	−0.708438	−0.998459
H	−3.440000	1.054609	−1.162556
H	−3.407969	−0.985986	1.165068
H	−2.514215	−0.659063	2.639211
H	−3.278372	0.680076	1.757018
H	2.876585	2.318618	0.138193
H	3.536864	0.827579	0.805331
H	3.439660	1.052811	−0.950347

Me_4BV (001 100 101 0)

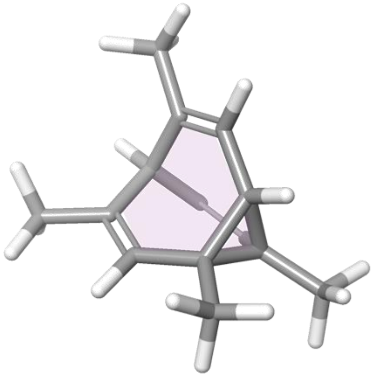

Table S119. Coordinates for the optimised geometry of **Me_4BV (001 100 101 0).**

	Coordinates / Å		
**Atom**	**x**	**y**	**z**
C	−0.835834	−1.077740	−0.773043
C	0.581609	−1.339758	−1.090659
C	1.654509	−0.580355	−0.792285
C	−1.458981	0.311488	−0.809854
C	−0.618862	1.510390	−1.108399
C	0.673746	1.717429	−0.807433
C	−1.290310	−0.443675	0.534366
C	−0.285331	0.018619	1.544723
C	0.951943	0.518301	1.346235
C	1.516818	0.730714	−0.043774
C	3.047177	−0.984738	−1.177865
C	−2.844202	0.408551	−1.429334
C	−2.517947	−1.062230	1.185818
C	1.840622	0.914172	2.489730
H	−1.460325	−1.886429	−1.149680
H	0.755855	−2.269301	−1.630001
H	−1.119332	2.315416	−1.645130
H	1.147307	2.649380	−1.100152
H	−0.609341	−0.078613	2.580568
H	2.514204	1.182110	0.050594
H	3.493630	−0.230069	−1.833446
H	3.673190	−1.086333	−0.285387
H	3.068106	−1.941732	−1.709570
H	−3.446755	1.149174	−0.892546
H	−2.775245	0.716426	−2.479149
H	−3.384884	−0.542444	−1.419513
H	−2.221993	−1.785772	1.954292
H	−3.125322	−0.284355	1.660939
H	−3.151545	−1.603735	0.477222
H	1.369522	0.736549	3.462076
H	2.086030	1.979412	2.428747
H	2.771939	0.339150	2.461912

Me_4BV (001 100 110 0)

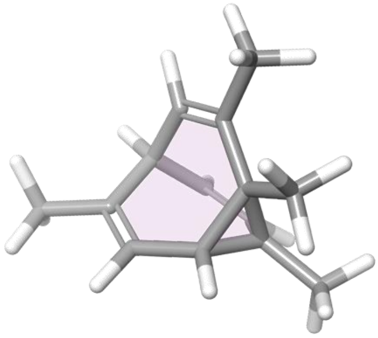

Table S120. Coordinates for the optimised geometry of **Me_4BV (001 100 110 0).**

	Coordinates / Å		
**Atom**	**x**	**y**	**z**
C	−0.175910	−0.627561	−1.219054
C	1.288301	−0.475201	−1.302078
C	2.145636	−0.145672	−0.317324
C	−0.902329	−1.210879	−0.015353
C	−0.134797	−1.611402	1.203122
C	0.992798	−1.071422	1.693362
C	−1.074727	0.290386	−0.396460
C	−0.475531	1.365632	0.501714
C	0.723779	1.307773	1.123170
C	1.657870	0.130526	1.085096
C	3.622979	−0.028960	−0.550052
C	−2.037472	−2.178850	−0.316417
C	−2.356949	0.706064	−1.114815
C	−1.302427	2.611778	0.750155
H	−0.567048	−0.927246	−2.190675
H	1.703878	−0.658741	−2.291527
H	−0.534971	−2.461283	1.754755
H	1.440839	−1.487860	2.590033
H	1.055473	2.135587	1.744267
H	2.519173	0.378572	1.720072
H	3.964879	0.984745	−0.317321
H	3.896199	−0.242016	−1.588787
H	4.164445	−0.735018	0.087871
H	−2.860657	−2.024890	0.389501
H	−2.432630	−2.075504	−1.331060
H	−1.695072	−3.216236	−0.224204
H	−2.219269	1.657061	−1.640078
H	−2.669576	−0.010624	−1.880262
H	−3.182544	0.805105	−0.402587
H	−2.289394	2.347323	1.142973
H	−0.841535	3.281787	1.484841
H	−1.423412	3.187023	−0.172879

Me$_4$BV (001 101 010 0)

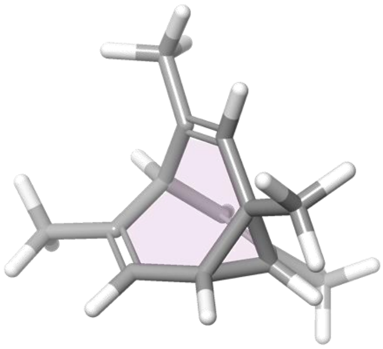

Table S121. Coordinates for the optimised geometry of **Me$_4$BV (001 101 010 0).**

	Coordinates / Å		
**Atom**	**x**	**y**	**z**
C	−0.804972	0.074032	1.603572
C	0.608089	0.452136	1.790364
C	1.558078	0.584856	0.843012
C	−1.259610	−0.966121	0.586526
C	−0.269082	−1.629646	−0.310879
C	0.845789	−1.105926	−0.861386
C	−1.616470	0.504757	0.402950
C	−1.056312	1.346863	−0.684416
C	0.210355	1.290582	−1.144921
C	1.256004	0.335196	−0.623077
C	2.967168	0.976719	1.177484
C	−2.390176	−1.878423	1.021942
C	1.750762	−1.915782	−1.743688
C	−2.019764	2.335610	−1.292874
H	−1.342230	0.085173	2.550324
H	0.892145	0.641831	2.823897
H	−0.486922	−2.672681	−0.537493
H	−2.648830	0.769888	0.629174
H	0.530097	1.951071	−1.945616
H	2.169588	0.533748	−1.200818
H	3.232967	1.908414	0.667677
H	3.109100	1.133056	2.251912
H	3.663901	0.194030	0.860210
H	−2.948197	−2.238208	0.150585
H	−1.995621	−2.744415	1.564624
H	−3.100026	−1.368616	1.682867
H	2.757197	−1.960567	−1.314930
H	1.396465	−2.944321	−1.868321
H	1.814865	−1.463338	−2.738596
H	−1.562466	2.929147	−2.091556
H	−2.381698	3.030694	−0.527920
H	−2.880195	1.810240	−1.720629

Me_4BV (001 101 100 0)

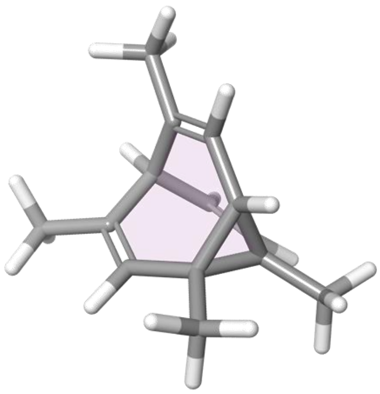

Table S122. Coordinates for the optimised geometry of **Me_4BV (001 101 100 0).**

	Coordinates / Å		
**Atom**	**x**	**y**	**z**
C	0.877532	−1.242472	−0.379397
C	−0.527619	−1.641445	−0.591639
C	−1.643829	−0.968741	−0.247576
C	1.360746	0.195595	−0.507356
C	0.390011	1.301546	−0.788543
C	−0.899781	1.417422	−0.410475
C	1.362597	−0.518329	0.869582
C	0.394156	−0.106853	1.930292
C	−0.887900	0.267642	1.787580
C	−1.578351	0.373732	0.453574
C	−3.015706	−1.502632	−0.538501
C	2.683979	0.394076	−1.231062
C	−1.739403	2.597709	−0.805449
C	2.685554	−0.993943	1.448883
H	1.547608	−2.004422	−0.774687
H	−0.650742	−2.599945	−1.093057
H	0.793657	2.113392	−1.393016
H	0.777468	−0.119787	2.949862
H	−1.466712	0.535421	2.666073
H	−2.604808	0.706695	0.660851
H	−3.565148	−0.806372	−1.180426
H	−3.576320	−1.635803	0.392416
H	−2.983448	−2.470842	−1.048908
H	3.314578	−0.499828	−1.222985
H	3.247033	1.212778	−0.770163
H	2.512327	0.644106	−2.284471
H	−1.186559	3.311375	−1.425113
H	−2.087956	3.130749	0.085089
H	−2.611919	2.266784	−1.378145
H	2.514321	−1.704177	2.266136
H	3.312972	−1.509483	0.715815
H	3.251662	−0.143944	1.844813

Me_4BV (001 110 010 0)

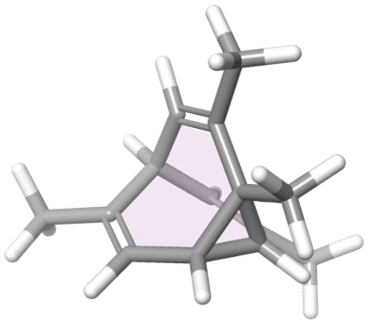

Table S123. Coordinates for the optimised geometry of **Me_4BV (001 110 010 0).**

Atom	Coordinates / Å x	y	z
C	−0.211463	−0.812456	−1.344940
C	−1.202869	−1.478588	−0.481503
C	−1.570632	−1.133543	0.767915
C	1.105667	−0.229950	−0.834777
C	1.433791	−0.273471	0.644264
C	0.540502	−0.155729	1.652241
C	−0.003995	0.684195	−1.354145
C	−0.783278	1.610716	−0.495074
C	−1.218789	1.341835	0.752280
C	−0.939604	0.048683	1.470327
C	−2.607649	−1.897655	1.536247
C	2.284688	−0.399023	−1.783647
C	2.883880	−0.468039	1.034919
C	−1.100349	2.949491	−1.114378
H	−0.175943	−1.285516	−2.325183
H	−1.686533	−2.343558	−0.931851
H	0.875068	−0.204303	2.684946
H	0.162641	1.106400	−2.345086
H	−1.791688	2.080838	1.304466
H	−1.377170	0.138969	2.473988
H	−2.176293	−2.301535	2.457896
H	−3.012045	−2.737664	0.961924
H	−3.443676	−1.241918	1.800229
H	3.018265	0.401002	−1.640413
H	2.772013	−1.367586	−1.631635
H	1.972580	−0.360969	−2.834018
H	3.027161	−0.484274	2.121227
H	3.500706	0.348038	0.645529
H	3.262532	−1.420248	0.650273
H	−1.690851	2.814104	−2.026667
H	−1.671951	3.597579	−0.441638
H	−0.174718	3.474176	−1.373717

Me_4BV (001 110 100 0)

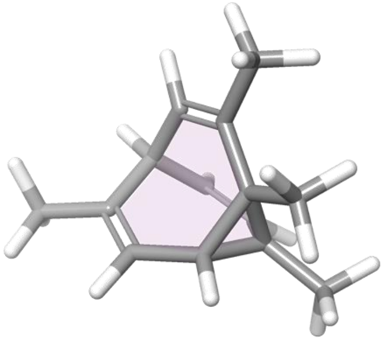

Table S124. Coordinates for the optimised geometry of **Me_4BV (001 110 100 0).**

	Coordinates / Å		
**Atom**	**x**	**y**	**z**
C	−0.179859	−0.313643	−1.334222
C	1.294266	−0.344754	−1.336771
C	2.125967	−0.358834	−0.277796
C	−1.004951	0.512336	−0.352315
C	−0.331231	1.272433	0.783581
C	0.812643	0.918355	1.411339
C	−1.043156	−1.045224	−0.316210
C	−0.404888	−1.804098	0.802240
C	0.749950	−1.542041	1.435534
C	1.593970	−0.335972	1.135512
C	3.617551	−0.393121	−0.433775
C	−2.180783	1.244607	−0.995832
C	−1.010302	2.532506	1.283017
C	−2.269820	−1.761474	−0.862506
H	−0.546869	−0.336236	−2.359717
H	1.741775	−0.359539	−2.329201
H	1.207116	1.536478	2.213444
H	−0.939465	−2.693124	1.134796
H	1.088868	−2.202375	2.227540
H	2.440226	−0.352118	1.835623
H	4.028390	−1.288695	0.043517
H	3.923311	−0.406434	−1.485128
H	4.068245	0.488912	0.032647
H	−2.534015	0.763131	−1.912727
H	−1.895211	2.260747	−1.287655
H	−3.028467	1.293988	−0.304690
H	−1.003989	3.308155	0.511093
H	−0.514186	2.955769	2.163822
H	−2.044009	2.322887	1.576026
H	−3.107916	−1.657972	−0.164982
H	−2.064923	−2.829980	−0.997502
H	−2.588238	−1.384671	−1.838702

Me_4BV (010 010 010 1)

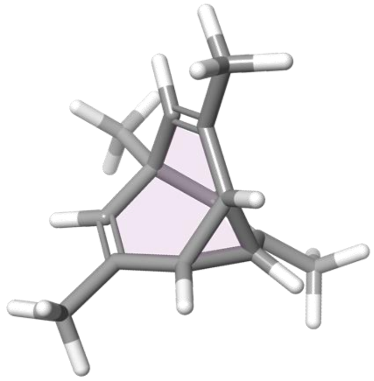

Table S125. Coordinates for the optimised geometry of **Me_4BV (010 010 010 1).**

	Coordinates / Å		
**Atom**	**x**	**y**	**z**
C	0.672882	−1.555928	0.418264
C	1.736602	−0.532645	0.279130
C	1.541626	0.765259	−0.036708
C	−0.688024	−1.251433	1.004574
C	−1.072439	0.095861	1.489330
C	−0.702048	1.267267	0.929918
C	−0.555469	−1.588921	−0.464123
C	−0.798829	−0.600747	−1.542202
C	−0.483506	0.710864	−1.491468
C	0.179727	1.384730	−0.301971
C	3.140345	−1.031403	0.520426
C	−1.953091	0.108224	2.714800
C	−1.456969	−1.154889	−2.782062
C	0.373957	2.881186	−0.628305
H	1.071244	−2.535710	0.679359
H	2.402241	1.425164	−0.110673
H	−1.107188	−2.048299	1.617878
H	−1.046984	2.196907	1.375332
H	−0.895007	−2.588522	−0.733096
H	−0.711013	1.341541	−2.347090
H	3.229794	−1.431384	1.535984
H	3.891492	−0.242641	0.406982
H	3.385569	−1.827974	−0.189984
H	−2.888620	−0.424170	2.513093
H	−1.445106	−0.385394	3.550046
H	−2.211190	1.122796	3.036152
H	−2.433268	−1.583487	−2.532084
H	−0.833974	−1.941323	−3.221086
H	−1.616758	−0.390600	−3.549896
H	1.006492	3.021786	−1.514099
H	0.851569	3.416220	0.202432
H	−0.584057	3.377662	−0.828852

Me_4BV (010 010 011 0)

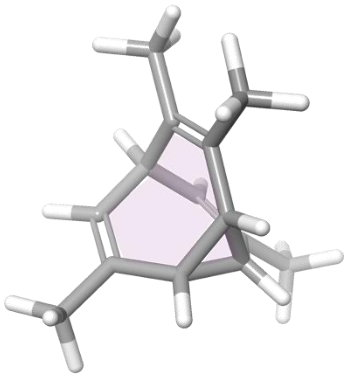

Table S126. Coordinates for the optimised geometry of **Me_4BV (010 010 011 0).**

Atom	Coordinates / Å x	y	z
C	1.202679	0.824734	0.962838
C	1.952777	0.165673	−0.131412
C	1.392356	−0.457035	−1.187468
C	−0.089927	1.581439	0.739324
C	−0.719515	1.736740	−0.592387
C	−0.734152	0.794755	−1.556169
C	−0.098878	0.276243	1.503480
C	−0.733033	−0.977866	0.997182
C	−0.767938	−1.373750	−0.303031
C	−0.096039	−0.560568	−1.402071
C	3.454791	0.215498	−0.008934
C	−1.387062	3.065530	−0.842443
C	−1.339110	−1.823862	2.099039
C	−1.464749	−2.610180	−0.811532
H	1.860567	1.260298	1.713920
H	2.017547	−0.921352	−1.944365
H	−0.206400	2.470997	1.357562
H	−1.224280	0.988488	−2.505576
H	−0.229935	0.399256	2.578527
H	−0.230626	−1.079607	−2.361404
H	3.775047	−0.272173	0.917829
H	3.798313	1.255189	0.008531
H	3.959387	−0.287336	−0.840704
H	−2.181904	3.233576	−0.108094
H	−1.836876	3.126935	−1.839043
H	−0.657072	3.877345	−0.756718
H	−2.355796	−1.482902	2.318218
H	−0.747152	−1.747324	3.018319
H	−1.363678	−2.889599	1.857631
H	−0.726709	−3.354984	−1.125939
H	−2.092212	−2.360490	−1.674655
H	−2.130422	−3.073670	−0.080454

Me_4BV (010 010 100 1)

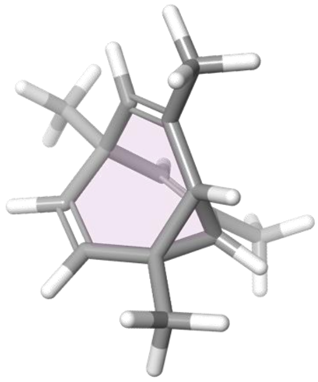

Table S127. Coordinates for the optimised geometry of **Me_4BV (010 010 100 1).**

Atom	Coordinates / Å		
	x	y	z
C	0.410225	1.415389	0.345606
C	−1.070059	1.323106	0.282309
C	−1.779390	0.240798	−0.102331
C	1.288639	0.254901	0.751989
C	0.746155	−1.076324	1.122547
C	−0.329860	−1.674200	0.568268
C	1.334139	0.771138	−0.681701
C	0.776499	−0.041353	−1.796528
C	−0.298400	−0.848441	−1.777848
C	−1.161129	−1.071795	−0.550841
C	−1.805307	2.574763	0.697058
C	1.495650	−1.786184	2.224186
C	2.565297	1.567819	−1.067854
C	−2.292779	−2.055819	−0.914759
H	0.737677	2.381397	0.729309
H	−2.865072	0.295256	−0.104635
H	2.137310	0.532316	1.376826
H	−0.632476	−2.654264	0.928235
H	1.308582	0.029498	−2.744321
H	−0.566321	−1.371318	−2.691877
H	−1.517342	3.412578	0.053215
H	−1.559661	2.831598	1.732941
H	−2.892872	2.464589	0.631402
H	2.538333	−1.945441	1.929498
H	1.065242	−2.764538	2.462550
H	1.480720	−1.185101	3.139519
H	2.955294	2.156435	−0.229978
H	2.331042	2.263708	−1.880870
H	3.364864	0.897907	−1.402583
H	−2.948081	−2.251253	−0.056379
H	−2.922314	−1.664721	−1.724201
H	−1.894602	−3.022446	−1.248753

Me_4BV (010 010 101 0)

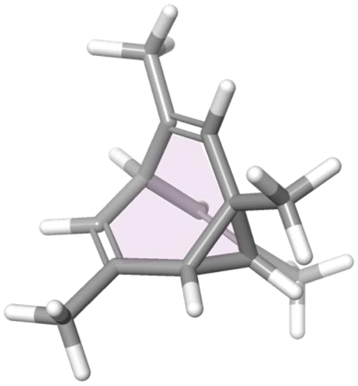

Table S128. Coordinates for the optimised geometry of **Me_4BV (010 010 101 0).**

Atom	Coordinates / Å		
	x	y	z
C	−0.467530	−1.422816	0.129204
C	−1.486650	−0.803999	−0.756206
C	−1.455322	0.462097	−1.220285
C	1.009591	−1.113366	0.028646
C	1.572131	−0.163201	−0.964443
C	0.977508	0.971760	−1.385909
C	0.225772	−0.668542	1.259122
C	−0.060415	0.776053	1.500671
C	−0.324134	1.740647	0.595206
C	−0.363649	1.446659	−0.889868
C	−2.634589	−1.703509	−1.142404
C	2.923116	−0.539201	−1.520763
C	0.477257	−1.455539	2.531402
C	−0.593922	3.160225	1.000598
H	−0.693534	−2.469179	0.333101
H	−2.249350	0.829451	−1.863653
H	1.659282	−1.976274	0.172930
H	1.467705	1.608152	−2.116706
H	−0.051412	1.073621	2.548573
H	−0.594873	2.374828	−1.430082
H	−3.166608	−2.042530	−0.247226
H	−2.262211	−2.582237	−1.679491
H	−3.360883	−1.201278	−1.790145
H	3.657611	−0.612900	−0.711805
H	3.297389	0.193593	−2.243425
H	2.866598	−1.507780	−2.028653
H	1.338816	−1.046218	3.070193
H	0.684804	−2.512145	2.328646
H	−0.398158	−1.410099	3.188447
H	−1.591108	3.466543	0.668245
H	0.145405	3.830327	0.550011
H	−0.548638	3.296858	2.086070

Me_4BV (010 010 110 0)

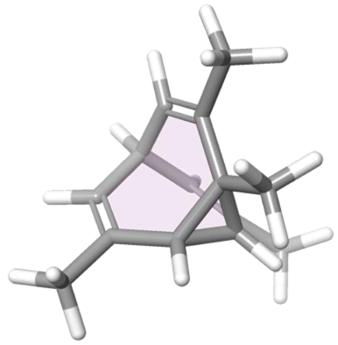

Table S129. Coordinates for the optimised geometry of **Me_4BV (010 010 110 0).**

	Coordinates / Å		
**Atom**	**x**	**y**	**z**
C	0.648693	−0.556572	−0.996405
C	1.375818	−1.338455	0.034744
C	1.294749	−1.142857	1.366081
C	0.359772	0.921200	−0.863337
C	0.776277	1.728088	0.310876
C	0.819032	1.290367	1.585183
C	−0.796253	−0.079602	−0.844836
C	−1.560584	−0.344476	0.437194
C	−1.031353	−0.353019	1.681129
C	0.416066	−0.098206	1.993963
C	2.273690	−2.428011	−0.497263
C	1.183466	3.148261	0.004860
C	−1.616385	−0.124432	−2.127701
C	−3.044533	−0.624407	0.324018
H	0.935646	−0.865451	−2.001667
H	1.868118	−1.762444	2.049013
H	0.476497	1.482986	−1.790198
H	1.141950	1.951806	2.383469
H	−1.662966	−0.554029	2.542080
H	0.539122	−0.172175	3.082597
H	1.688753	−3.149837	−1.077057
H	3.042781	−1.999969	−1.148860
H	2.783025	−2.978242	0.300957
H	1.476805	3.702720	0.902556
H	2.034410	3.157696	−0.684417
H	0.352009	3.687351	−0.461408
H	−2.406823	0.632965	−2.111874
H	−2.065501	−1.112866	−2.269067
H	−1.001612	0.075491	−3.013258
H	−3.223286	−1.517248	−0.283377
H	−3.513057	−0.803752	1.298288
H	−3.564324	0.227119	−0.126282

Me_4BV (010 011 100 0)

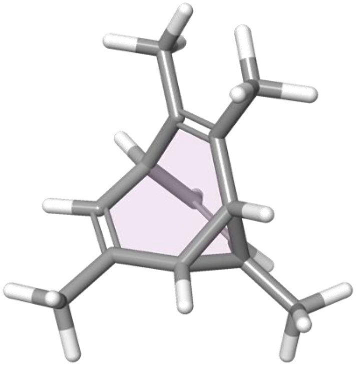

Table S130. Coordinates for the optimised geometry of **Me_4BV (010 011 100 0).**

	Coordinates / Å		
**Atom**	**x**	**y**	**z**
C	−1.325926	0.210546	0.762635
C	−0.877595	1.598688	0.495732
C	0.094500	1.951105	−0.368704
C	−0.381028	−0.968216	0.772312
C	1.087426	−0.861315	0.509682
C	1.677983	0.010653	−0.350242
C	−1.443239	−0.863287	−0.315315
C	−1.052933	−0.574661	−1.720708
C	−0.052842	0.214413	−2.144874
C	0.857739	0.963112	−1.209355
C	−1.590016	2.669707	1.283756
C	1.903994	−1.865701	1.299056
C	3.165609	0.152053	−0.550812
C	−2.622705	−1.808383	−0.194281
H	−2.091233	0.178542	1.537995
H	0.363666	2.995473	−0.495494
H	−0.593868	−1.684496	1.566342
H	−1.645686	−1.064903	−2.491434
H	0.123568	0.329973	−3.209626
H	1.537082	1.548413	−1.844460
H	−1.448430	2.506759	2.357396
H	−2.663262	2.647256	1.067041
H	−1.224852	3.675181	1.049231
H	1.353933	−2.806439	1.416999
H	2.123562	−1.470914	2.295987
H	2.841608	−2.137647	0.807428
H	3.452624	1.208779	−0.505895
H	3.763115	−0.353574	0.210690
H	3.451942	−0.241472	−1.531326
H	−2.888321	−2.005055	0.850395
H	−2.391615	−2.770686	−0.664083
H	−3.504797	−1.383906	−0.686067

Me_4BV (010 100 011 0)

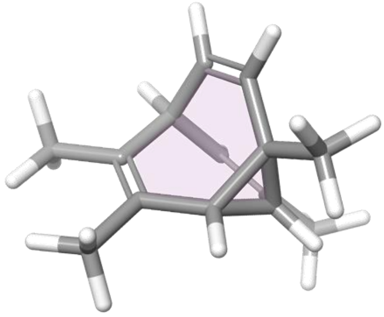

Table S131. Coordinates for the optimised geometry of **Me_4BV (010 100 011 0).**

	Coordinates / Å		
**Atom**	**x**	**y**	**z**
C	1.399231	−0.403685	−0.513050
C	1.394262	1.067624	−0.698527
C	0.497455	1.917907	−0.160781
C	1.044665	−1.092471	0.801753
C	0.614408	−0.298802	1.983170
C	−0.119472	0.825533	1.988335
C	0.132782	−1.221227	−0.412382
C	−1.242917	−0.638578	−0.481147
C	−1.613404	0.579069	−0.002995
C	−0.631226	1.479735	0.733334
C	2.496002	1.611452	−1.573757
C	1.863413	−2.317765	1.158546
C	−2.246072	−1.566699	−1.137621
C	−2.989158	1.179861	−0.143018
H	2.194700	−0.877532	−1.088029
H	0.566007	2.983763	−0.356955
H	0.928665	−0.678685	2.954068
H	−0.366408	1.299718	2.932975
H	0.190806	−2.172493	−0.942071
H	−1.152407	2.392723	1.053123
H	2.449816	2.700725	−1.677075
H	3.473994	1.359223	−1.150391
H	2.429653	1.179295	−2.577818
H	2.165932	−2.884633	0.271010
H	2.774791	−2.026523	1.692107
H	1.284578	−2.991092	1.800204
H	−2.214985	−1.445096	−2.224903
H	−3.269786	−1.407913	−0.788957
H	−2.018126	−2.613001	−0.903609
H	−2.914364	2.210342	−0.508503
H	−3.493766	1.194442	0.828277
H	−3.629070	0.654783	−0.855314

Me_4BV (010 100 100 1)

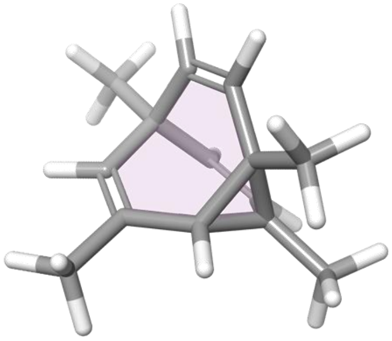

Table S132. Coordinates for the optimised geometry of **Me_4BV (010 100 100 1).**

	Coordinates / Å		
**Atom**	**x**	**y**	**z**
C	−0.974114	0.676883	−0.432580
C	0.292041	1.368033	−0.790931
C	1.540531	0.946823	−0.498073
C	−1.136509	−0.837024	−0.450774
C	0.025141	−1.722978	−0.761729
C	1.326842	−1.531877	−0.486659
C	−1.185814	−0.053154	0.886830
C	−0.072021	−0.178284	1.874174
C	1.248269	−0.282710	1.644957
C	1.860275	−0.327392	0.260426
C	0.116547	2.659613	−1.554281
C	−2.435755	−1.363618	−1.040909
C	−2.531702	0.161815	1.562089
C	3.391111	−0.460221	0.394693
H	−1.836127	1.238115	−0.793255
H	2.387834	1.547651	−0.818930
H	−0.217594	−2.645702	−1.287923
H	2.036427	−2.293728	−0.797844
H	−0.372756	−0.178926	2.921445
H	1.914884	−0.361446	2.499466
H	1.071303	3.136986	−1.798846
H	−0.468089	3.371690	−0.962191
H	−0.411592	2.473198	−2.495422
H	−3.245287	−0.628107	−1.019177
H	−2.767193	−2.251523	−0.491777
H	−2.295690	−1.641120	−2.092122
H	−3.314780	0.476702	0.866082
H	−2.860887	−0.761828	2.050243
H	−2.458461	0.946751	2.323789
H	3.879167	−0.503516	−0.587307
H	3.822957	0.390135	0.937650
H	3.671043	−1.371243	0.938887

Me_4BV (010 100 101 0)

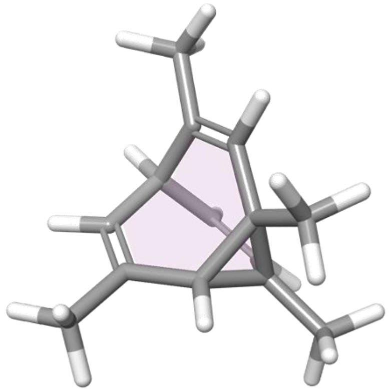

Table S133. Coordinates for the optimised geometry of **Me_4BV (010 100 101 0).**

Atom	Coordinates / Å x	y	z
C	−0.910256	0.824299	0.373628
C	0.241321	1.743569	0.174741
C	1.412963	1.424845	−0.412076
C	−1.333383	−0.227796	−0.644692
C	−0.541299	−0.445103	−1.892986
C	0.783472	−0.315119	−2.072015
C	−0.752012	−0.648073	0.731358
C	0.606223	−1.273630	0.823088
C	1.721378	−0.991065	0.118873
C	1.729791	0.062005	−0.966717
C	0.038417	3.146307	0.693969
C	−2.829418	−0.364673	−0.882614
C	−1.700789	−1.181613	1.794684
C	3.015085	−1.715404	0.352868
H	−1.730678	1.336765	0.875967
H	2.197668	2.169747	−0.506528
H	−1.114633	−0.743852	−2.769726
H	1.213521	−0.510374	−3.049340
H	0.691577	−2.060266	1.572312
H	2.734973	0.117208	−1.405946
H	−0.816275	3.614226	0.194061
H	−0.156950	3.125885	1.771362
H	0.911553	3.785899	0.526988
H	−3.132236	0.210029	−1.765614
H	−3.090546	−1.415240	−1.049614
H	−3.431616	0.007441	−0.048544
H	−1.985505	−2.213571	1.562817
H	−2.615602	−0.589180	1.888947
H	−1.219761	−1.169703	2.779641
H	2.932657	−2.465607	1.146188
H	3.332427	−2.230108	−0.559856
H	3.797932	−1.007848	0.644774

Me_4BV (010 100 110 0)

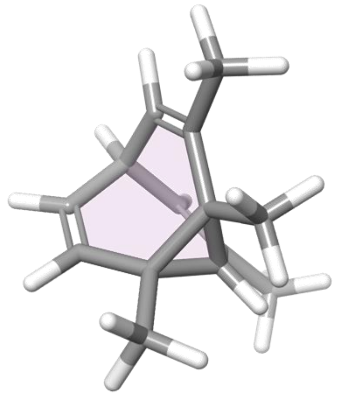

Table S134. Coordinates for the optimised geometry of **Me_4BV (010 100 110 0).**

	Coordinates / Å		
**Atom**	**x**	**y**	**z**
C	−0.971160	0.468397	0.296866
C	−1.776140	−0.673717	−0.195148
C	−1.329855	−1.628444	−1.027294
C	0.064023	1.224621	−0.527970
C	0.389978	0.793637	−1.907064
C	0.383536	−0.450183	−2.405870
C	0.499293	0.385784	0.699575
C	1.288950	−0.875149	0.509327
C	1.095500	−1.783210	−0.466555
C	0.076065	−1.666070	−1.571423
C	−3.193522	−0.716263	0.314934
C	0.077259	2.737321	−0.395030
C	0.855945	1.152748	1.961707
C	2.408577	−1.128338	1.492009
H	−1.575183	1.111781	0.946454
H	−2.000285	−2.438976	−1.333492
H	0.655923	1.601704	−2.599642
H	0.642705	−0.605874	−3.458859
H	1.743903	−2.665058	−0.509891
H	0.166885	−2.554095	−2.214478
H	−3.745581	−1.586039	−0.075765
H	−3.742574	0.196322	0.016101
H	−3.208447	−0.773138	1.419556
H	1.112340	3.122561	−0.423989
H	−0.482472	3.196090	−1.228189
H	−0.393711	3.089658	0.534812
H	0.793038	0.496080	2.844675
H	1.875626	1.570879	1.904523
H	0.165586	1.988000	2.148946
H	2.021960	−1.281566	2.515479
H	2.984134	−2.027445	1.221540
H	3.117704	−0.282019	1.514156

Me$_4$BV (010 101 100 0)

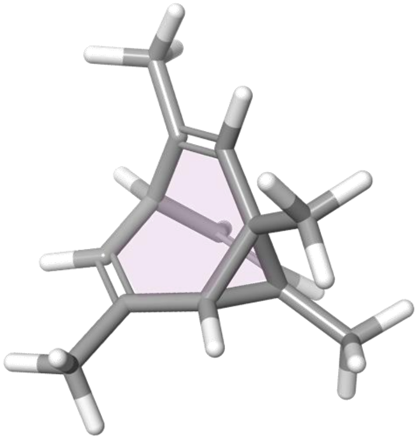

Table S135. Coordinates for the optimised geometry of **Me$_4$BV (010 101 100 0).**

	Coordinates / Å		
**Atom**	**x**	**y**	**z**
C	−1.092417	0.519674	0.429183
C	−0.195900	1.705837	0.424912
C	1.031402	1.765088	−0.130880
C	−0.587751	−0.908608	0.591086
C	0.879619	−1.198914	0.675268
C	1.908047	−0.558645	0.082204
C	−1.222045	−0.441996	−0.746105
C	−0.372114	−0.278889	−1.964248
C	0.884592	0.187878	−2.046600
C	1.684240	0.613481	−0.847079
C	−0.747216	2.928092	1.118155
C	−1.401931	−1.802810	1.514676
C	3.333680	−0.980019	0.289837
C	−2.633418	−0.895092	−1.086974
H	−2.024581	0.742515	0.948288
H	1.612414	2.681103	−0.076990
H	1.137073	−2.043599	1.313737
H	−0.834620	−0.573851	−2.905376
H	1.371873	0.246157	−3.014790
H	2.655426	0.970421	−1.215525
H	−1.678459	3.246887	0.638038
H	−0.053076	3.774709	1.091071
H	−0.956638	2.704015	2.169484
H	−0.961378	−1.821090	2.518293
H	−1.420551	−2.827426	1.127923
H	−2.435656	−1.465885	1.635659
H	3.418557	−1.836175	0.967148
H	3.788208	−1.264766	−0.664634
H	3.913043	−0.156926	0.720595
H	−3.046829	−0.286110	−1.899318
H	−3.327456	−0.803594	−0.246304
H	−2.626137	−1.941464	−1.410734

Me_4BV (010 110 100 0)

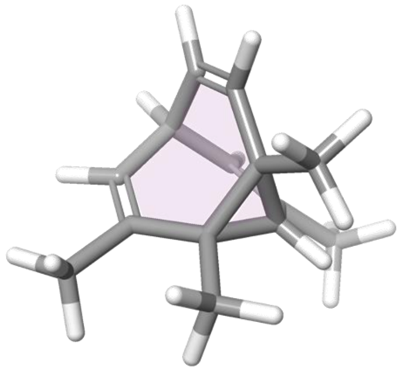

Table S136. Coordinates for the optimised geometry of **Me_4BV (010 110 100 0).**

	Coordinates / Å		
**Atom**	**x**	**y**	**z**
C	0.551839	0.205292	−0.963631
C	1.563259	1.025935	−0.249503
C	1.773861	1.034183	1.081258
C	−0.858091	−0.050930	−0.436955
C	−1.277293	0.424993	0.948857
C	−0.469095	0.556923	2.024428
C	0.170156	−1.215560	−0.569578
C	0.770643	−1.853078	0.641636
C	1.160990	−1.267727	1.785220
C	0.987883	0.198584	2.047341
C	2.408278	1.913870	−1.130690
C	−1.946082	0.070459	−1.502783
C	−2.740618	0.758629	1.163095
C	0.007594	−2.210075	−1.710767
H	0.620581	0.375901	−2.038423
H	2.540615	1.671131	1.512077
H	−0.873488	0.902469	2.972017
H	0.921530	−2.930341	0.582521
H	1.591747	−1.870773	2.578157
H	1.369270	0.405302	3.055866
H	3.135677	2.501122	−0.560266
H	1.772353	2.616297	−1.679865
H	2.964578	1.308861	−1.854474
H	−1.575382	−0.113972	−2.515613
H	−2.362614	1.083078	−1.521429
H	−2.751738	−0.646480	−1.314097
H	−3.373997	−0.090792	0.888087
H	−3.029810	1.631799	0.570230
H	−2.968669	0.994396	2.208815
H	0.914896	−2.814151	−1.828370
H	−0.830272	−2.884975	−1.505378
H	−0.168602	−1.730372	−2.677782

Me_4BV (011 100 100 0)

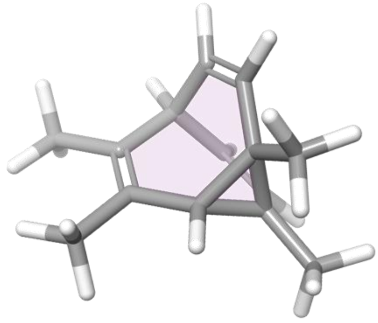

Table S137. Coordinates for the optimised geometry of **Me_4BV (011 100 100 0).**

	Coordinates / Å		
**Atom**	**x**	**y**	**z**
C	−0.589028	0.850208	−0.124115
C	0.896417	1.022056	−0.212022
C	1.825382	0.043039	−0.047860
C	−1.335415	−0.351578	−0.685584
C	−0.584741	−1.495406	−1.281462
C	0.623093	−1.965775	−0.932545
C	−1.289170	−0.092545	0.844977
C	−0.497530	−0.983187	1.743065
C	0.693239	−1.554722	1.505376
C	1.428218	−1.403131	0.205034
C	1.305815	2.445191	−0.542338
C	3.318285	0.255738	−0.075421
C	−2.619190	−0.049127	−1.443063
C	−2.532518	0.453126	1.531910
H	−1.093794	1.807934	−0.254603
H	−1.075586	−2.004057	−2.110223
H	1.046181	−2.806954	−1.472689
H	−0.938922	−1.185844	2.718106
H	1.152334	−2.175295	2.268410
H	2.334728	−2.019475	0.278501
H	1.350969	3.044637	0.372314
H	2.267077	2.507785	−1.058692
H	0.582090	2.910553	−1.221402
H	3.742064	−0.171889	−0.989635
H	3.615935	1.304668	−0.015448
H	3.785037	−0.237700	0.784708
H	−3.362651	−0.830635	−1.252619
H	−3.060488	0.914224	−1.171126
H	−2.429062	−0.010342	−2.521978
H	−3.001924	1.273570	0.981259
H	−2.281873	0.847590	2.523527
H	−3.274972	−0.342657	1.655637

Me_4BV (100 100 100 1)

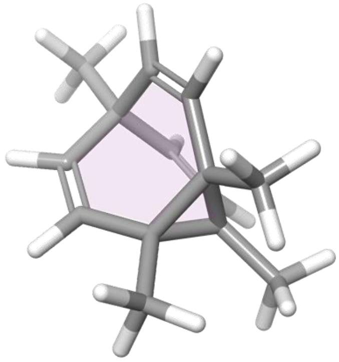

Table S138. Coordinates for the optimised geometry of **Me_4BV (100 100 100 1).**

	Coordinates / Å		
**Atom**	**x**	**y**	**z**
C	−0.905237	0.062286	0.880084
C	0.303454	0.113593	1.769294
C	1.607677	0.085557	1.448503
C	−0.880991	0.748860	−0.510826
C	0.351321	1.469048	−0.976656
C	1.646303	1.179368	−0.767386
C	−0.889461	−0.799160	−0.409981
C	0.334599	−1.587074	−0.777579
C	1.632810	−1.286821	−0.606740
C	2.124702	−0.009520	0.032339
C	−2.177150	0.123989	1.720277
C	−2.129588	1.470772	−1.008160
C	−2.146203	−1.565850	−0.810331
C	3.666137	−0.016425	0.055799
H	0.090048	0.184248	2.836061
H	2.336234	0.133998	2.253422
H	0.166939	2.361592	−1.574900
H	2.396393	1.837562	−1.197715
H	0.140078	−2.547617	−1.255118
H	2.375378	−2.003407	−0.947524
H	−3.108148	0.092231	1.154657
H	−2.194222	1.051746	2.304220
H	−2.203918	−0.719910	2.419655
H	−3.075545	1.015443	−0.715698
H	−2.143129	2.498754	−0.627260
H	−2.125056	1.512869	−2.103707
H	−2.170662	−2.535521	−0.299298
H	−3.086934	−1.066154	−0.580070
H	−2.142918	−1.749764	−1.891154
H	4.056602	−0.867170	0.628667
H	4.084235	−0.084670	−0.956595
H	4.066256	0.897149	0.513721

Me_4BV (100 100 101 0)

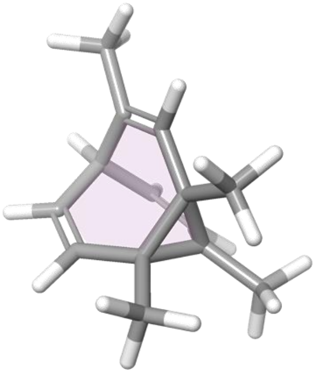

Table S139. Coordinates for the optimised geometry of **Me_4BV (100 100 101 0).**

Atom	Coordinates / Å		
	x	y	z
C	−1.089162	−0.696279	0.259238
C	−0.257782	−1.943282	0.161886
C	1.004288	−2.092144	−0.270403
C	−0.952216	0.423895	−0.807391
C	0.013106	0.272487	−1.947968
C	1.222111	−0.310441	−1.966939
C	−0.425269	0.655636	0.633948
C	1.055003	0.729911	0.902008
C	2.074881	0.059698	0.329094
C	1.845936	−0.953899	−0.764781
C	−2.469422	−1.030214	0.816771
C	−2.200966	1.165669	−1.274153
C	−1.170470	1.620833	1.551922
C	3.501732	0.278675	0.742254
H	−0.749185	−2.858320	0.492311
H	1.456778	−3.078940	−0.268500
H	−0.314187	0.699796	−2.895722
H	1.793354	−0.325883	−2.889957
H	1.331389	1.439243	1.682434
H	2.810771	−1.372513	−1.080530
H	−3.144327	−0.181198	0.923062
H	−2.371260	−1.481630	1.811059
H	−2.971593	−1.752372	0.162304
H	−2.960520	1.322267	−0.508531
H	−1.926427	2.156957	−1.653625
H	−2.682737	0.610411	−2.087541
H	−0.976175	1.364319	2.599996
H	−0.819568	2.645247	1.380303
H	−2.253116	1.635437	1.428248
H	3.593771	1.025149	1.538025
H	3.937227	−0.655618	1.111285
H	4.094035	0.627103	−0.110107

Me_4BV (100 100 110 0)

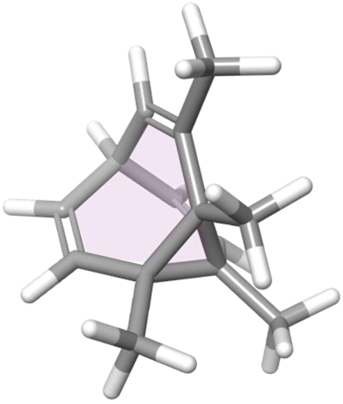

Table S140. Coordinates for the optimised geometry of **Me_4BV (100 100 110 0).**

	Coordinates / Å		
**Atom**	**x**	**y**	**z**
C	−0.628239	−0.406912	0.940204
C	0.060398	−1.717763	1.190373
C	0.913384	−2.394527	0.406746
C	−1.118914	−0.072691	−0.493540
C	−0.844121	−0.979517	−1.657398
C	0.203697	−1.786833	−1.878265
C	0.051665	0.743072	0.136286
C	1.502646	0.553229	−0.333802
C	2.061043	−0.585832	−0.800210
C	1.354942	−1.898686	−0.932777
C	−1.488741	−0.041741	2.148386
C	−2.499239	0.563033	−0.656116
C	−0.259563	2.193752	0.519862
C	2.433441	1.750768	−0.252836
H	−0.173634	−2.193633	2.142701
H	1.315635	−3.343924	0.745957
H	−1.605845	−0.982702	−2.437102
H	0.242223	−2.380158	−2.786369
H	3.106432	−0.599063	−1.097364
H	2.064092	−2.627074	−1.346703
H	−0.872225	−0.026339	3.055165
H	−1.978355	0.930332	2.095011
H	−2.276994	−0.791323	2.284760
H	−3.252764	−0.214665	−0.830117
H	−2.850476	1.136005	0.201990
H	−2.499211	1.236759	−1.520744
H	0.342367	2.498978	1.382349
H	−0.050908	2.864721	−0.320349
H	−1.297884	2.384822	0.790996
H	2.089992	2.554898	−0.910767
H	3.457576	1.510392	−0.560839
H	2.497581	2.122620	0.774511

A Selection of 3−D and Common Ring Systems

(x =) denotes the ranking in the 100 most frequently used ring systems from small molecule drugs listed in the FDA Orange Book before January 2020, sorted by descending frequency (f) and then ascending molecular weight when f is equal. (x_n =) denotes the ranking in the top most frequently used ring systems in U.S. clinical trial compounds in January 2020 that were not present in drugs, sorted by descending frequency (f).[33b]

1,4−dimethyladamantane
(x = 63, f = 5)

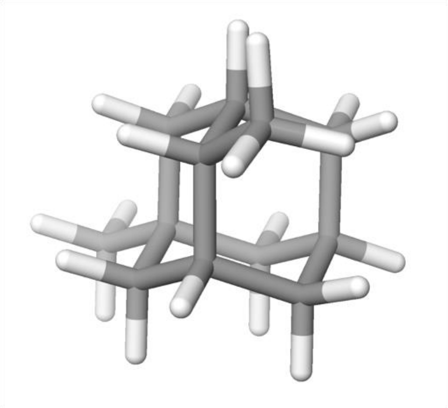

Table S141. Coordinates for the optimised geometry of **1,4−dimethyladamantane**

Atom	Coordinates / Å x	y	z
C	−0.640902	−0.783378	1.101290
C	−1.336293	−1.004576	−0.263862
C	−0.744643	−0.015415	−1.297130
C	0.759192	−0.319582	−1.458289
C	1.497052	−0.140058	−0.109598
C	0.862832	−1.086806	0.937811
C	1.304632	1.318789	0.365839
C	−0.795844	0.675166	1.570620
C	−0.192158	1.633363	0.531307
C	−0.899241	1.440583	−0.819850
C	−2.861537	−0.921030	−0.171128
C	2.986847	−0.452296	−0.274020
H	−1.065251	−1.459002	1.853146
H	−1.109717	−2.026226	−0.600794
H	−1.243390	−0.140273	−2.265370
H	0.890756	−1.344248	−1.829327
H	1.190072	0.345818	−2.217680
H	0.997307	−2.133073	0.634235
H	1.368428	−0.974488	1.905720
H	1.750732	2.015672	−0.355430
H	1.823199	1.479247	1.319893
H	−0.291038	0.809169	2.535730
H	−1.850700	0.917988	1.739377
H	−0.314590	2.668842	0.868159
H	−0.468380	2.121997	−1.564336
H	−1.957654	1.709715	−0.733279
H	−3.241137	−1.666876	0.535464
H	−3.313921	−1.128195	−1.146876
H	−3.218615	0.057935	0.157783

H	3.139456	−1.483578	−0.610832
H	3.450824	0.212035	−1.011366
H	3.523682	−0.327220	0.672792

1,2−dimethylcubane
(x = N/A, f = N/A)

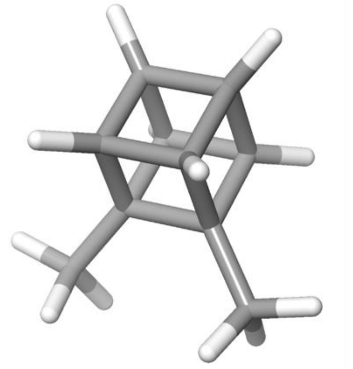

Table S142. Coordinates for the optimised geometry of **1,2−dimethylcubane**

	Coordinates / Å		
**Atom**	**x**	**y**	**z**
C	−0.730636	0.408936	0.166836
C	0.803663	0.285214	−0.040939
C	0.554435	−0.580387	−1.293555
C	0.570258	−1.845964	−0.426376
C	0.808245	−0.991417	0.825442
C	−0.706930	−0.869238	1.030627
C	−0.945784	−1.723713	−0.221075
C	−0.960740	−0.458205	−1.088370
C	1.799995	1.385947	0.053240
C	−1.480569	1.650479	0.497493
H	1.063697	−0.470890	−2.242703
H	1.095896	−2.761072	−0.666842
H	1.524595	−1.217284	1.605215
H	−1.218021	−0.996128	1.976621
H	−1.653500	−2.539363	−0.294522
H	−1.678917	−0.249723	−1.871298
H	1.586780	2.174571	−0.675163
H	2.813012	1.018883	−0.139303
H	1.793309	1.840106	1.049059
H	−1.346101	2.411062	−0.278000
H	−1.139565	2.076605	1.446223
H	−2.553122	1.451580	0.587389

1,4−dimethylcubane
(x = N/A, f = N/A)

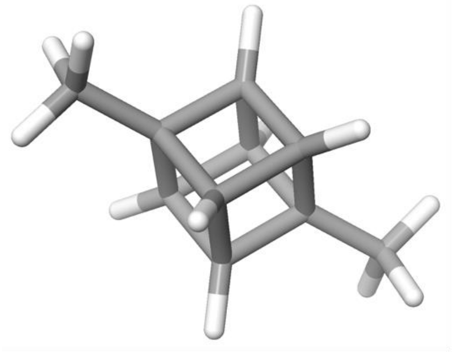

Table S143. Coordinates for the optimised geometry of **1,4−dimethylcubane**

Atom	Coordinates / Å		
	x	y	z
C	−0.345815	−1.248554	0.441061
C	−1.334456	−0.087772	0.203399
C	−0.521107	0.334732	−1.038079
C	0.345814	1.248575	−0.162631
C	−0.451712	0.827108	1.078085
C	0.521105	−0.334715	1.316512
C	1.334456	0.087794	0.075035
C	0.451714	−0.827087	−0.799650
C	−2.816427	−0.185288	0.274703
C	2.816430	0.185272	0.003735
H	−0.621747	−2.266211	0.686806
H	−0.939946	0.608039	−1.998305
H	0.621735	2.266219	−0.408449
H	−0.813963	1.501873	1.843346
H	0.939938	−0.607980	2.276755
H	0.813970	−1.501895	−1.564873
H	−3.199818	−0.907510	−0.452891
H	−3.285988	0.780952	0.065267
H	−3.143346	−0.506961	1.268495
H	3.143360	0.506906	−0.990061
H	3.199841	0.907483	0.731322
H	3.285963	−0.780980	0.213183

***p*−xylene**
(x = 1, f = 727) – regiochemistry not listed

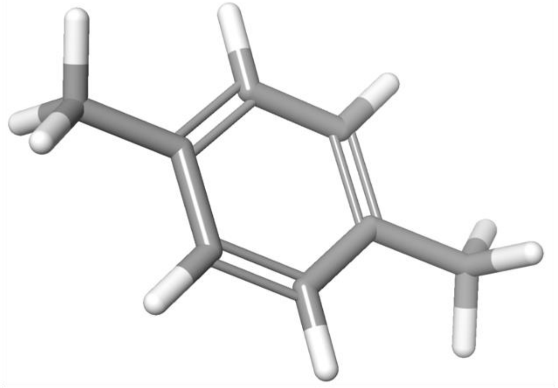

Table S144. Coordinates for the optimised geometry of ***p*−xylene**

Atom	Coordinates / Å x	y	z
C	0.695728	−1.016929	0.650407
C	−0.699689	−0.975858	0.706646
C	−1.407457	0.035379	0.047845
C	−0.695728	1.016924	−0.650416
C	0.699688	0.975853	−0.706655
C	1.407456	−0.035383	−0.047851
C	2.903131	−0.098547	−0.136240
C	−2.903131	0.098552	0.136246
H	1.223706	−1.819901	1.159573
H	−1.230785	−1.742360	1.266276
H	−1.223706	1.819896	−1.159581
H	1.230785	1.742355	−1.266283
H	3.201794	−0.691514	−1.006133
H	3.331913	0.904969	−0.227516
H	3.329014	−0.552308	0.764845
H	−3.331918	−0.904961	0.227553
H	−3.329021	0.552297	−0.764844
H	−3.201780	0.691537	1.006130

***o*−xylene**
(x = 1, f = 727) – regiochemistry not listed

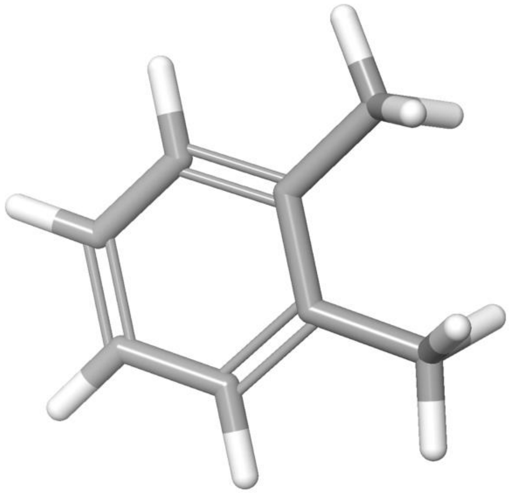

Table S145. Coordinates for the optimised geometry of ***o*−xylene**

Atom	Coordinates / Å x	y	z
C	0.701148	−1.445854	0.569158
C	2.017267	−0.990609	0.495058
C	2.281155	0.299637	0.047991
C	1.229113	1.135564	−0.325294
C	−0.100990	0.691718	−0.256259
C	−0.368502	−0.616248	0.196944
C	−1.774670	−1.141837	0.290243
C	−1.209490	1.621541	−0.667252
H	0.514645	−2.458056	0.921286
H	2.835233	−1.643802	0.786464
H	3.305732	0.656641	−0.010634
H	1.455346	2.141379	−0.672402
H	−2.248800	−1.136972	−0.696278
H	−2.363589	−0.531867	0.982457
H	−1.795200	−2.172455	0.660325
H	−1.771502	1.196581	−1.504866
H	−1.886333	1.801703	0.173860
H	−0.820563	2.592936	−0.990801

***m*−xylene**
(x = 1, f = 727) – regiochemistry not listed

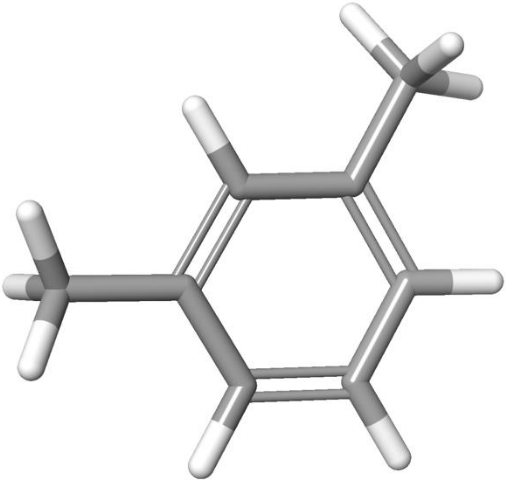

Table S146. Coordinates for the optimised geometry of ***m*−xylene**

Atom	Coordinates / Å x	y	z
C	0.005666	−0.009673	−0.000849
C	0.005103	−0.040497	1.392175
C	1.207498	−0.036700	2.097037
C	2.429408	0.001839	1.415835
C	2.407852	0.028339	0.016710
C	1.212322	0.021362	−0.709903
C	1.225157	0.011446	−2.213759
C	3.732605	0.049600	2.164881
H	−0.943406	−0.011816	−0.547528
H	−0.944643	−0.067826	1.935893
H	1.198713	−0.061956	3.191930
H	3.359113	0.055639	−0.527942
H	0.338323	0.519256	−2.629278
H	2.124177	0.508716	−2.615345
H	1.221588	−1.024252	−2.602497
H	3.651881	−0.440597	3.149756
H	4.048194	1.094733	2.343499
H	4.542451	−0.444527	1.601813

1,4−dimethylpiperazine
(x = 4, f = 65)

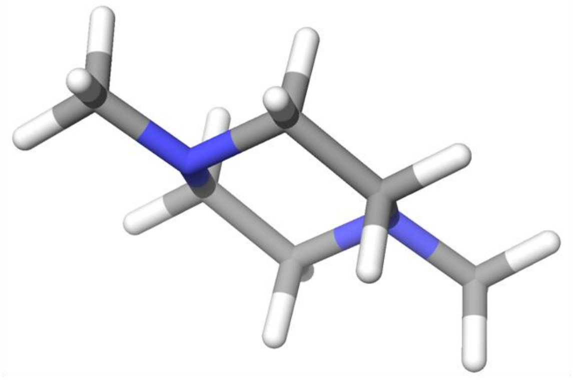

Table S147. Coordinates for the optimised geometry of **1,4−dimethylpiperazine**

	Coordinates / Å		
**Atom**	**x**	**y**	**z**
C	0.903737	0.869965	0.727689
C	−0.626497	0.913159	0.945763
N	−1.372247	0.472637	−0.249752
C	−0.903737	−0.869961	−0.646471
C	0.626499	−0.913158	−0.864537
N	1.372246	−0.472635	0.330979
C	2.809212	−0.463164	0.070169
C	−2.809212	0.463160	0.011063
H	1.177711	1.614832	−0.031939
H	1.391825	1.164065	1.664756
H	−0.908580	1.944034	1.191821
H	−0.881836	0.288149	1.812417
H	−1.391824	−1.164050	−1.583543
H	−1.177716	−1.614834	0.113149
H	0.908582	−1.944034	−1.110593
H	0.881843	−0.288147	−1.731189
H	3.359915	−0.165197	0.969217
H	3.081339	0.220107	−0.742446
H	3.157394	−1.467843	−0.193913
H	−3.157389	1.467829	0.275196
H	−3.359925	0.165240	−0.887998
H	−3.081339	−0.220153	0.823645

(1*S*,2*S*)−1,2−dimethylcyclobutane
(x = 53, f = 5) – regiochemistry/stereochemistry not listed

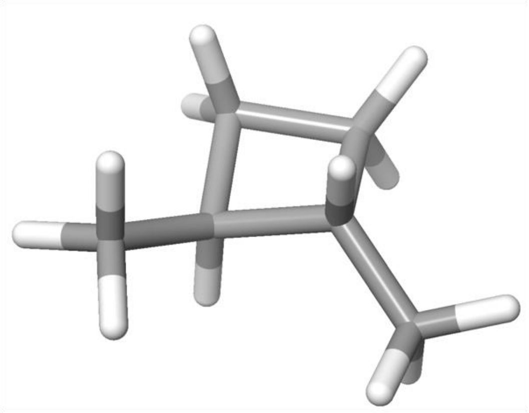

Table S148. Coordinates for the optimised geometry of **(1*S*,2*S*)−1,2−dimethylcyclobutane**

	Coordinates / Å		
**Atom**	**x**	**y**	**z**
C	0.916944	1.253670	0.375995
C	0.715594	−0.271969	0.185562
C	−0.740272	0.062563	−0.261840
C	−0.608870	1.360548	0.576223
C	−1.821238	−0.909441	0.165296
C	1.602548	−0.946293	−0.841240
H	1.527573	1.548099	1.233483
H	1.278433	1.780137	−0.515203
H	0.753348	−0.810735	1.143544
H	−0.816133	0.277970	−1.337603
H	−0.920109	1.260488	1.622738
H	−1.079978	2.250051	0.149978
H	−1.767489	−1.129480	1.236508
H	−1.725407	−1.854278	−0.379084
H	−2.813516	−0.498452	−0.046152
H	2.639439	−0.978703	−0.491893
H	1.274054	−1.975761	−1.015992
H	1.585079	−0.418414	−1.800322

(1*R*,3*R*)−1,3−dimethylcyclobutane
(x = 53, f = 5) – regiochemistry/stereochemistry not listed

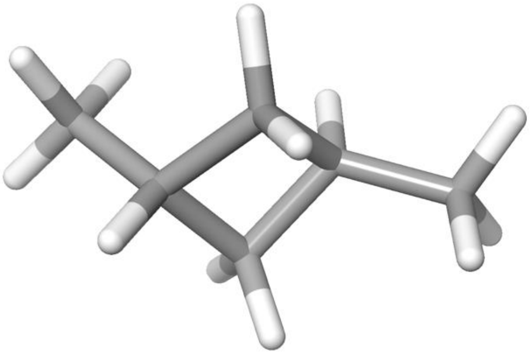

Table S149. Coordinates for the optimised geometry of **(1*R*,3*R*)−1,3−dimethylcyclobutane**

	Coordinates / Å		
**Atom**	**x**	**y**	**z**
C	−0.786766	0.398338	0.291260
C	−0.279218	−1.063620	0.225455
C	1.016202	−0.531820	−0.436856
C	0.207560	0.722755	−0.851248
C	2.192075	−0.289298	0.497129
C	−2.255692	0.618434	−0.007679
H	−0.506611	0.889920	1.233512
H	−0.159050	−1.563292	1.190909
H	−0.864759	−1.715457	−0.434151
H	1.339422	−1.136262	−1.293569
H	−0.221093	0.646664	−1.857874
H	0.725356	1.682294	−0.765307
H	2.996117	0.235346	−0.028983
H	2.593934	−1.240573	0.860604
H	1.909224	0.312093	1.367022
H	−2.877029	0.211233	0.796322
H	−2.474747	1.687518	−0.093486
H	−2.554924	0.135728	−0.943822

(1*R*,2*S*)−1,2−dimethylcyclobutane
(x = 53, f = 5) – regiochemistry/stereochemistry not listed

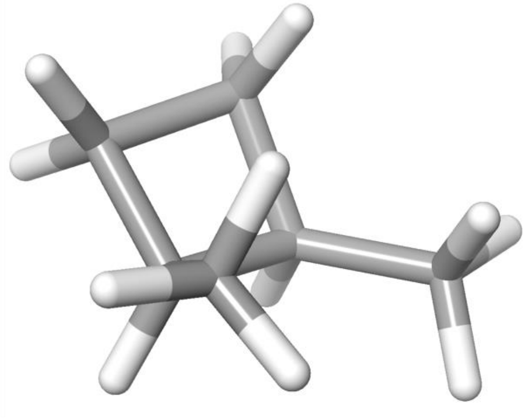

Table S150. Coordinates for the optimised geometry of **(1*R*,2*S*)−1,2−dimethylcyclobutane**

	Coordinates / Å		
**Atom**	**x**	**y**	**z**
C	0.968200	1.136831	0.751119
C	0.568977	−0.360218	0.685250
C	−0.878364	0.126199	0.350971
C	−0.243127	1.446783	−0.150795
C	−1.713208	−0.645565	−0.651346
C	1.326541	−1.183325	−0.346753
H	0.884792	1.566602	1.756454
H	1.944767	1.401892	0.337040
H	0.629304	−0.859653	1.661046
H	−1.462814	0.285570	1.269945
H	−0.001627	1.459457	−1.220001
H	−0.792525	2.359306	0.095689
H	−1.964175	−1.636659	−0.260342
H	−1.191823	−0.777878	−1.604414
H	−2.650768	−0.118182	−0.855170
H	0.886555	−2.181299	−0.440404
H	1.318031	−0.712766	−1.335059
H	2.371264	−1.307095	−0.043231

(1*S*,3*S*)−1,3−dimethylcyclobutane
(x = 53, f = 5) – regiochemistry/stereochemistry not listed

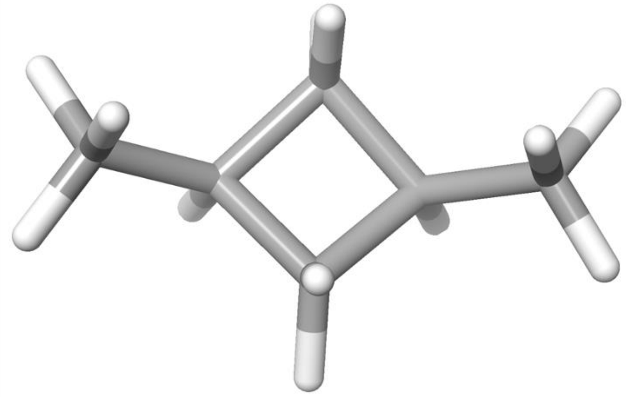

Table S151. Coordinates for the optimised geometry of **(1*S*,3*S*)−1,3−dimethylcyclobutane**

Atom	Coordinates / Å x	y	z
C	−1.086066	−0.161162	−0.549925
C	−0.132902	−0.933459	0.398418
C	1.038486	−0.425301	−0.481323
C	0.118613	0.757333	−0.880733
C	2.323423	−0.072041	0.238271
C	−2.288593	0.501357	0.089348
H	−1.388705	−0.769864	−1.414021
H	−0.268486	−2.018268	0.420460
H	−0.119575	−0.558608	1.428971
H	1.237297	−1.096349	−1.329226
H	0.207102	1.637452	−0.232436
H	0.192318	1.079447	−1.923081
H	2.812204	−0.974780	0.618286
H	3.020726	0.426952	−0.442192
H	2.141736	0.596478	1.086189
H	−2.003141	1.111793	0.952355
H	−3.006478	−0.251359	0.430395
H	−2.797960	1.150378	−0.630076

(1*R*,4*R*,6*R*)−2,6−dimethylbicyclo[2.2.1]hept−2−ene
(x = N/A, f = N/A)

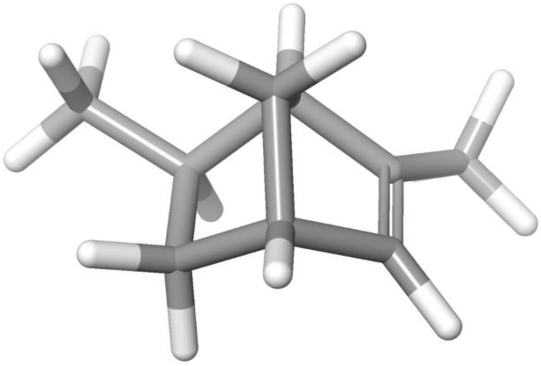

Table S152. Coordinates for the optimised geometry of **(1*R*,4*R*,6*R*)−2,6−dimethylbicyclo[2.2.1]hept−2−ene**

	Coordinates / Å		
**Atom**	**x**	**y**	**z**
C	1.434707	−0.257624	−0.103102
C	1.000604	−1.296389	−0.835715
C	−0.446494	−1.525765	−0.465566
C	−1.283445	−0.354143	−1.025049
C	−0.755653	0.870972	−0.212978
C	0.270002	0.212389	0.748013
C	−0.375016	−1.153032	1.017565
C	−1.871976	1.605140	0.520024
C	2.757227	0.408735	−0.103281
H	1.556345	−1.845259	−1.580564
H	−0.822594	−2.527018	−0.677370
H	−1.132215	−0.214569	−2.101205
H	−2.351384	−0.523541	−0.846116
H	−0.260475	1.583015	−0.886033
H	0.542695	0.779896	1.639137
H	0.261956	−1.826123	1.606146
H	−1.357807	−1.091997	1.496204
H	−2.586977	2.024721	−0.195343
H	−2.424382	0.947508	1.198156
H	−1.462470	2.430284	1.111819
H	2.654364	1.461375	−0.383904
H	3.443206	−0.065352	−0.812436
H	3.209782	0.356780	0.891597

1,3−dimethylazetidine
(x = 79, f = 3) – regiochemistry not listed

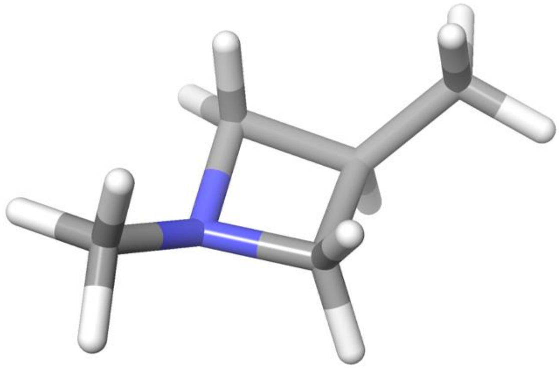

Table S153. Coordinates for the optimised geometry of **1,3−dimethylazetidine**

	Coordinates / Å		
**Atom**	**x**	**y**	**z**
C	−0.122260	−1.062935	0.190609
C	1.030199	−0.226432	−0.417957
C	−0.082494	0.806274	−0.724032
N	−1.046973	−0.322889	−0.705391
C	2.135041	0.209302	0.520564
C	−2.263322	0.051483	0.006810
H	−0.066006	−2.138265	−0.003878
H	−0.273167	−0.909461	1.267120
H	1.441297	−0.681837	−1.330775
H	−0.220481	1.567053	0.055311
H	0.007356	1.310067	−1.691215
H	1.735956	0.664274	1.433053
H	2.751897	−0.646738	0.811891
H	2.785696	0.942894	0.034072
H	−2.884011	−0.835189	0.174819
H	−2.078435	0.523408	0.979328
H	−2.850295	0.748989	−0.600329

1,2−dimethylazetidine
(x = 79, f = 3) – regiochemistry not listed

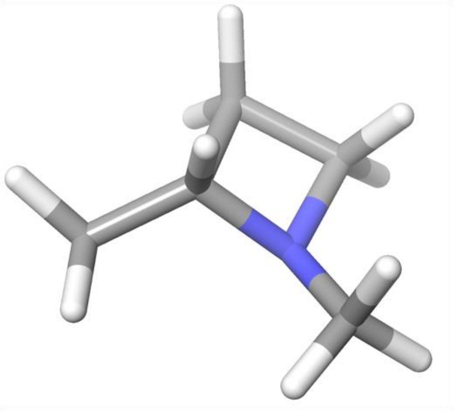

Table S154. Coordinates for the optimised geometry of **1,2−dimethylazetidine**

	Coordinates / Å		
**Atom**	**x**	**y**	**z**
C	−1.224481	−0.942037	−0.283553
C	0.175512	−1.538017	−0.042527
C	0.608462	−0.068807	0.180075
N	−0.551607	0.324515	−0.670074
C	−1.248121	1.470329	−0.094969
C	1.933682	0.343363	−0.430271
H	−1.844110	−0.901577	0.621451
H	−1.800128	−1.415934	−1.083882
H	0.271259	−2.204577	0.818115
H	0.610696	−2.021375	−0.925386
H	0.536126	0.230485	1.235749
H	−0.648157	2.376779	−0.229177
H	−2.197523	1.628842	−0.617615
H	−1.465066	1.364408	0.974718
H	2.011738	0.049049	−1.482692
H	2.763543	−0.124038	0.109902
H	2.068173	1.428592	−0.370711

2,5−dimethylpyridine
(x = 2, f = 89) – regiochemistry not listed

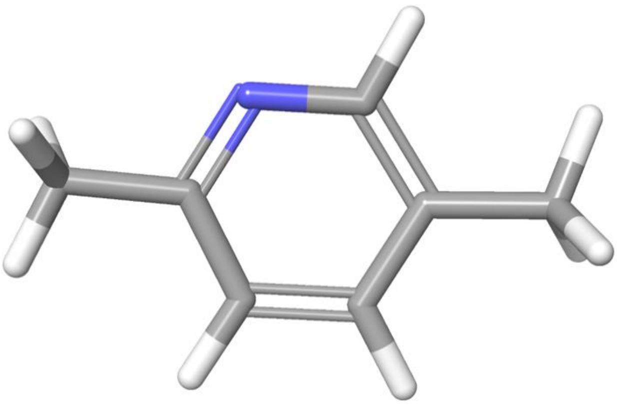

Table S155. Coordinates for the optimised geometry of **2,5−dimethylpyridine**

	Coordinates / Å		
**Atom**	**x**	**y**	**z**
C	0.607063	1.185478	0.319087
C	1.331710	0.034474	−0.002074
C	0.575514	−1.109147	−0.286266
C	−0.810400	−1.045089	−0.250640
C	−1.434166	0.170523	0.070853
N	−0.720136	1.259414	0.353397
C	−2.930715	0.289145	0.115923
C	2.830472	0.037209	−0.069781
H	1.146563	2.109926	0.568649
H	1.076736	−2.051813	−0.534073
H	−1.414114	−1.931633	−0.468527
H	−3.361559	−0.365462	0.894991
H	−3.206309	1.330489	0.340163
H	−3.386893	−0.002475	−0.846857
H	3.248666	0.910908	0.578352
H	3.262137	−0.985080	0.318364
H	3.183431	0.187858	−1.184368

2,3−dimethylpyridine
(x = 2, f = 89) – regiochemistry not listed

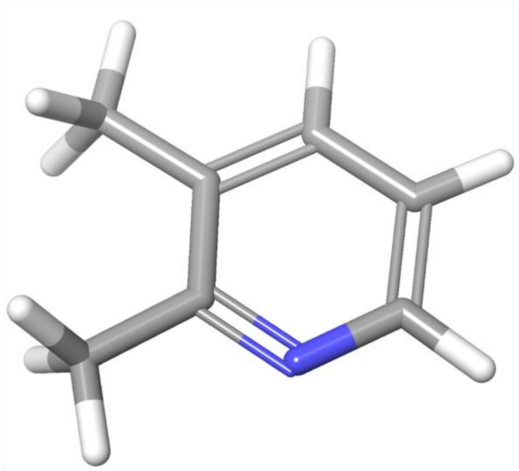

Table S156. Coordinates for the optimised geometry of **2,3−dimethylpyridine**

	Coordinates / Å		
**Atom**	**x**	**y**	**z**
C	2.026479	−1.132988	0.050331
C	2.289290	0.214047	−0.108098
C	1.210899	1.089006	−0.164919
C	−0.095517	0.597490	−0.062446
C	−0.262898	−0.784283	0.096298
N	0.783109	−1.647127	0.152974
C	−1.626301	−1.402272	0.214839
C	−1.261218	1.538915	−0.123389
H	2.832320	−1.859159	0.100779
H	3.308418	0.574439	−0.185492
H	1.394599	2.153235	−0.289204
H	−2.150362	−1.006608	1.090153
H	−2.215866	−1.202626	−0.685115
H	−1.551690	−2.488570	0.332031
H	−1.841402	1.488188	0.803383
H	−0.932978	2.576148	−0.250014
H	−1.906881	1.292164	−0.972110

2,4−dimethylpyridine
(x = 2, f = 89) – regiochemistry not listed

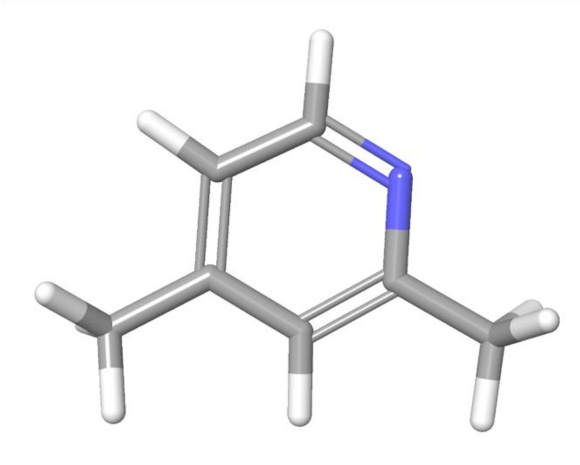

Table S157. Coordinates for the optimised geometry of **2,4−dimethylpyridine**

Atom	Coordinates / Å		
	x	y	z
C	0.053592	1.802621	−0.843692
C	−1.144846	1.192697	−0.482560
C	−1.106896	−0.072542	0.113505
C	0.153572	−0.638697	0.309204
C	1.307601	0.047636	−0.089846
N	1.250386	1.254800	−0.656887
C	2.669339	−0.560556	0.087794
C	−2.362827	−0.797010	0.503357
H	0.043428	2.797772	−1.307633
H	−2.097064	1.702603	−0.658173
H	0.243076	−1.622861	0.781330
H	2.892942	−1.261218	−0.737999
H	3.431377	0.233353	0.070090
H	2.747274	−1.125479	1.031862
H	−3.133481	−0.098443	0.868155
H	−2.170858	−1.545924	1.289695
H	−2.789850	−1.330565	−0.365811

3,4−dimethylpyridine
(x = 2, f = 89) – regiochemistry not listed

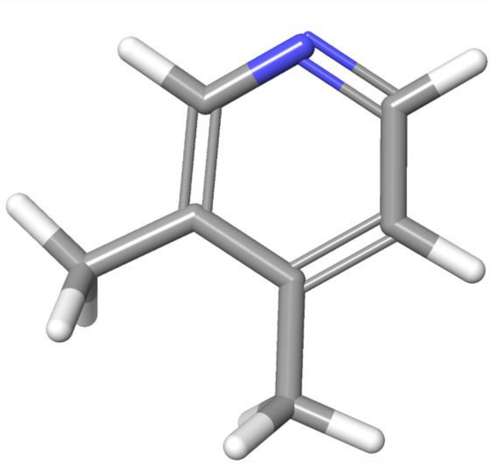

Table S158. Coordinates for the optimised geometry of **3,4−dimethylpyridine**

Atom	Coordinates / Å x	y	z
C	2.052237	−1.230017	−0.208132
C	1.748296	0.082244	0.117208
C	0.407163	0.479617	0.147301
C	−0.582684	−0.470355	−0.154497
C	−0.164132	−1.762667	−0.468358
N	1.126022	−2.163370	−0.502110
C	0.058330	1.898072	0.497858
C	−2.043711	−0.135082	−0.147165
H	3.082142	−1.573131	−0.241135
H	2.549658	0.778169	0.342470
H	−0.880544	−2.543509	−0.711099
H	−0.567253	1.925261	1.395638
H	−0.472648	2.375440	−0.331772
H	0.954800	2.493244	0.702062
H	−2.257986	0.650246	−0.878907
H	−2.352593	0.200232	0.847952
H	−2.657097	−1.004395	−0.407315

1,4−dimethylpiperidine
(x = 3, f = 76) – regiochemistry not listed

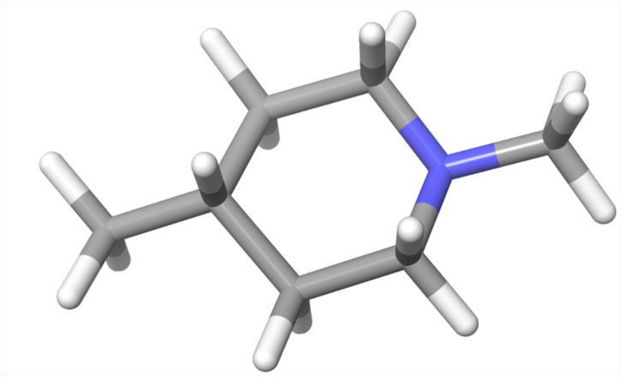

Table S159. Coordinates for the optimised geometry of **1,4−dimethylpiperidine**

	Coordinates / Å		
**Atom**	**x**	**y**	**z**
N	1.473133	0.115400	0.326515
C	0.873361	0.932488	−0.736870
C	−0.601354	1.219666	−0.446967
C	−1.394205	−0.077434	−0.253778
C	−0.708121	−0.951040	0.801923
C	0.770124	−1.166446	0.470727
C	2.894222	−0.107309	0.060911
C	−2.841000	0.216394	0.133239
H	1.399506	1.892232	−0.808569
H	0.966975	0.436236	−1.711920
H	−0.679992	1.845314	0.451979
H	−1.028070	1.798714	−1.274685
H	−1.401707	−0.624818	−1.205832
H	−1.211139	−1.923292	0.866726
H	−0.794475	−0.482254	1.791117
H	1.220514	−1.746898	1.285161
H	0.858623	−1.766694	−0.444490
H	3.354148	−0.674270	0.877957
H	3.428984	0.847240	0.002565
H	3.060313	−0.653710	−0.874584
H	−3.408722	−0.713253	0.245389
H	−2.897793	0.763971	1.080115
H	−3.333322	0.819762	−0.636628

2,6−dimethyl−2−azaspiro[3.3]heptane
(x = N/A, f =N/A)

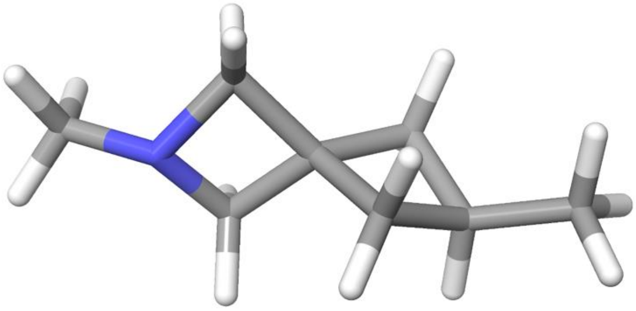

Table S160. Coordinates for the optimised geometry of
2,6−dimethyl−2−azaspiro[3.3]heptane

Atom	Coordinates / Å		
	x	y	z
C	0.604791	0.612061	−0.576342
C	1.786818	−0.358672	−0.275138
C	1.066740	−0.726726	1.057661
C	−0.192529	−0.187751	0.427443
C	−1.235706	−1.150509	−0.075688
N	−2.276021	−0.326964	0.594295
C	−1.233235	0.489073	1.268961
C	3.160895	0.266772	−0.151248
C	−2.991682	0.483310	−0.391771
H	0.250925	0.604625	−1.611052
H	0.790307	1.650264	−0.276727
H	1.805934	−1.206444	−0.975188
H	1.076454	−1.790037	1.312789
H	1.412049	−0.154205	1.926638
H	−1.323551	−1.252799	−1.163408
H	−1.166414	−2.160023	0.346855
H	−1.318345	1.575889	1.156001
H	−1.159389	0.271074	2.341124
H	3.508041	0.628515	−1.124302
H	3.158225	1.113322	0.543143
H	3.886308	−0.467458	0.213277
H	−2.332235	1.040171	−1.067939
H	−3.639616	1.203891	0.119002
H	−3.638765	−0.157375	−1.000653

2,5−dimethyloctahydrocyclopenta[*c*]pyrrole
(x = N/A, f = N/A)

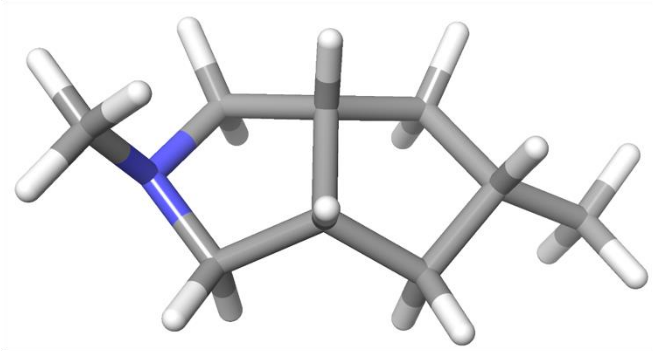

Table S161. Coordinates for the optimised geometry of **2,5−dimethyloctahydrocyclopenta[*c*]pyrrole**

	Coordinates / Å		
**Atom**	**x**	**y**	**z**
C	−0.268551	−0.646190	0.752670
C	−1.358368	−1.298924	−0.089632
N	−2.276579	−0.207731	−0.461287
C	−1.395790	0.952118	−0.686906
C	−0.293200	0.836874	0.359155
C	1.116178	1.173349	−0.123719
C	1.993205	0.141485	0.587357
C	1.154529	−1.133817	0.488481
C	−3.257836	0.055337	0.591653
C	3.371278	−0.000766	−0.035076
H	−0.505773	−0.737964	1.820566
H	−0.945461	−1.727649	−1.010769
H	−1.857998	−2.117726	0.440281
H	−1.924955	1.909946	−0.628377
H	−0.988828	0.880526	−1.702803
H	−0.541933	1.438467	1.243076
H	1.202827	1.069472	−1.211952
H	1.398873	2.198912	0.134761
H	2.102875	0.423405	1.642958
H	1.466787	−1.887066	1.218972
H	1.246882	−1.580363	−0.508824
H	−3.893738	−0.823961	0.743019
H	−3.921949	0.873833	0.292546
H	−2.805025	0.318148	1.553801
H	3.951326	−0.770284	0.484589
H	3.922852	0.942733	0.030053
H	3.308372	−0.282162	−1.091644

3,6−dimethyl−3−azabicyclo[3.1.0]hexane
(x = 83, f = 3) – regiochemistry not listed

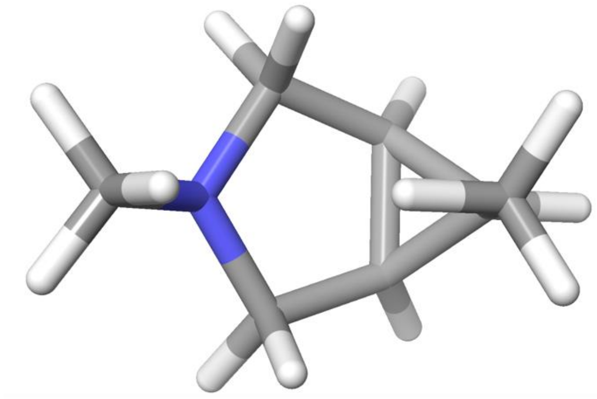

Table S162. Coordinates for the optimised geometry of **3,6−dimethyl−3−azabicyclo[3.1.0]hexane**

	Coordinates / Å		
**Atom**	**x**	**y**	**z**
C	0.377589	−0.671634	1.327468
C	1.717099	−0.422727	0.670750
C	0.672323	−1.281246	−0.006675
C	−0.357796	−0.770660	−0.967672
N	−1.431595	−0.316029	−0.073599
C	−0.828239	0.202381	1.161842
C	2.121626	0.913057	0.149749
C	−2.309688	0.652038	−0.709924
H	0.319421	−1.326750	2.186275
H	2.572515	−0.939564	1.095886
H	0.815230	−2.352249	−0.058046
H	0.051362	0.034206	−1.588046
H	−0.718805	−1.569628	−1.623101
H	−0.539588	1.256494	1.086946
H	−1.519764	0.087035	2.002528
H	1.297034	1.479095	−0.291060
H	2.544102	1.515264	0.960406
H	2.892796	0.794032	−0.617999
H	−2.775500	0.218569	−1.601655
H	−3.122235	0.935711	−0.032166
H	−1.777885	1.562605	−1.008499

1,5−dimethylazocane
(x = 58, f = 5) – regiochemistry not listed

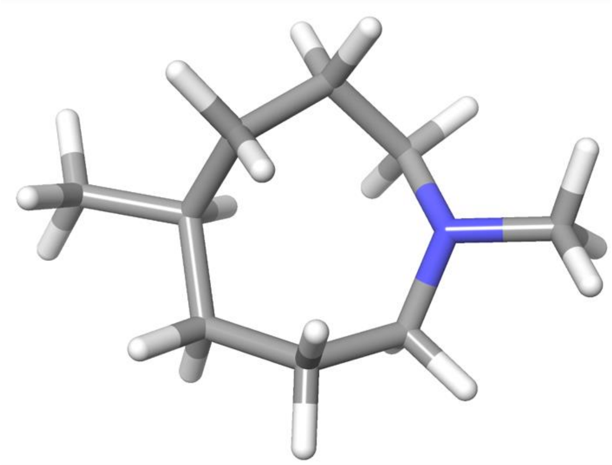

Table S163. Coordinates for the optimised geometry of **1,5−dimethylazocane**

	Coordinates / Å		
**Atom**	**x**	**y**	**z**
C	−1.442563	1.281611	−0.021493
C	−1.606317	−0.210498	0.308770
C	−1.014506	−1.128276	−0.771094
C	0.267004	−1.847000	−0.345718
C	−0.098562	1.750612	−0.582604
C	1.149847	1.380338	0.220080
C	1.318282	−1.056323	0.440216
N	1.809611	0.145021	−0.251177
C	−3.096493	−0.516691	0.512641
C	3.260187	0.264946	−0.045673
H	−2.212992	1.577187	−0.746665
H	−1.658435	1.858283	0.888045
H	−1.129180	−0.403150	1.275397
H	−0.832502	−0.580674	−1.701930
H	−1.738006	−1.907078	−1.047030
H	−0.019682	−2.708284	0.272698
H	0.734671	−2.271310	−1.243934
H	−0.148365	2.847580	−0.631130
H	0.014672	1.435621	−1.627054
H	0.949469	1.367199	1.298752
H	1.841408	2.221153	0.067683
H	2.145956	−1.764756	0.590314
H	0.974868	−0.832688	1.457205
H	−3.665586	−0.363306	−0.410725
H	−3.524195	0.129200	1.286975
H	−3.237858	−1.555364	0.830404
H	3.780465	−0.617767	−0.435144
H	3.517238	0.384033	1.013270
H	3.661565	1.120383	−0.601077

2,6−dimethyl−2,6−diazaspiro[3.3]heptane
(x = N/A, f = N/A)

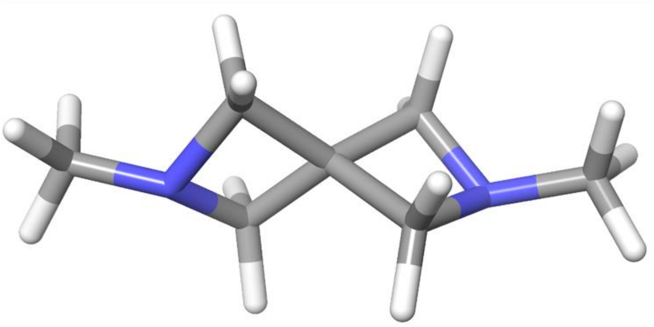

Table S164. Coordinates for the optimised geometry of **2,6−dimethyl−2,6−diazaspiro[3.3]heptane**

	Coordinates / Å		
**Atom**	**x**	**y**	**z**
C	−1.156108	0.796759	0.569577
N	−2.010012	0.340816	−0.568608
C	−0.984638	−0.681150	−0.925982
C	−0.006550	0.108810	−0.111879
C	1.059789	−0.567582	0.693648
N	1.954944	0.574081	0.348936
C	1.051730	0.937554	−0.784462
C	−3.222452	−0.301108	−0.070296
C	3.235276	0.088056	−0.155965
H	−1.415102	0.382294	1.552103
H	−1.086229	1.885020	0.663464
H	−1.171118	−1.694347	−0.547973
H	−0.777929	−0.746923	−1.998748
H	0.863209	−0.670175	1.765428
H	1.366175	−1.546624	0.303985
H	1.357876	0.568618	−1.771567
H	0.851774	2.010601	−0.865713
H	−3.034193	−1.058147	0.700531
H	−3.753825	−0.785045	−0.896767
H	−3.895675	0.452231	0.352619
H	3.812993	0.921578	−0.569594
H	3.138636	−0.677063	−0.935692
H	3.821428	−0.338254	0.665188

1,6−dimethyl−1,6−diazaspiro[3.3]heptane
(x = N/A, f = N/A)

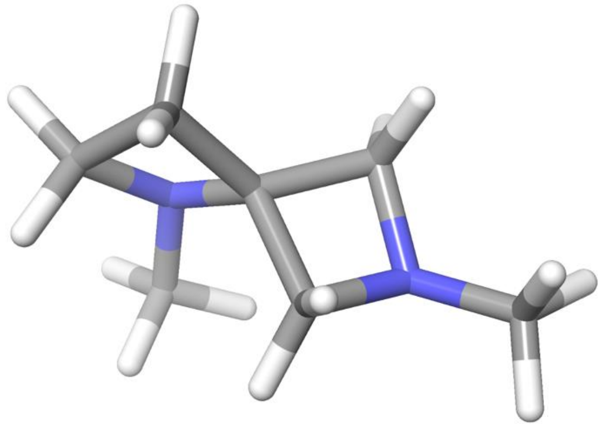

Table S165. Coordinates for the optimised geometry of **1,6−dimethyl−1,6−diazaspiro[3.3]heptane**

Atom	Coordinates / Å		
	x	y	z
C	1.017722	−0.017294	1.034134
N	1.404142	1.015572	0.024786
C	0.528127	0.357271	−0.986944
C	−0.159844	−0.360932	0.150663
N	−1.468010	0.015060	0.680582
C	−2.028027	−1.210809	0.048043
C	−0.591238	−1.791792	−0.003171
C	2.809707	0.872170	−0.338674
C	−2.054234	1.211913	0.097600
H	0.787181	0.395802	2.021835
H	1.734377	−0.838153	1.166376
H	−0.048199	1.057713	−1.596069
H	1.048257	−0.317632	−1.680618
H	−2.476332	−1.076803	−0.944651
H	−2.729282	−1.766043	0.678201
H	−0.322873	−2.299792	−0.932936
H	−0.347565	−2.439953	0.846497
H	3.039093	1.511116	−1.198175
H	3.095178	−0.155224	−0.594985
H	3.444425	1.202509	0.490554
H	−3.063829	1.352922	0.499384
H	−1.475410	2.098440	0.377575
H	−2.143365	1.183940	−0.993989

2,5−dimethyl−2,5−diazaspiro[3.4]octane
(x_n = 48, f = 1) – regiochemistry not listed

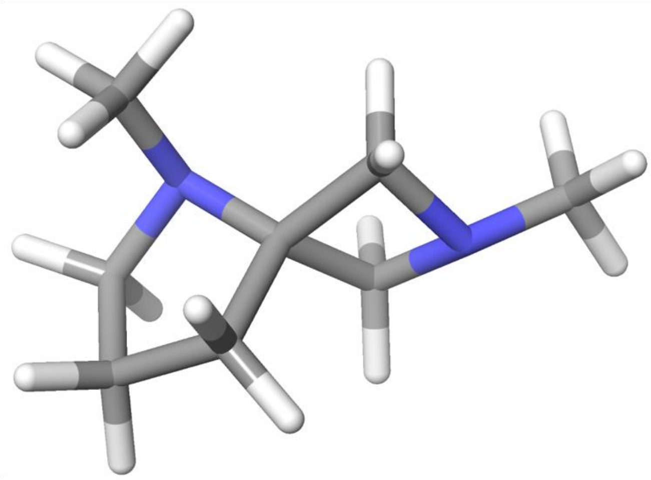

Table S166. Coordinates for the optimised geometry of **2,5−dimethyl−2,5−diazaspiro[3.4]octane**

Atom	Coordinates / Å x	y	z
C	1.298411	−0.876830	−0.170778
N	2.111016	0.372561	−0.191299
C	0.963601	1.080786	0.446297
C	0.030294	0.018795	−0.222594
C	3.226686	0.271301	0.742989
N	−1.024072	−0.361157	0.751570
C	−2.110580	0.581559	0.421591
C	−2.025530	0.877710	−1.075728
C	−0.640360	0.420274	−1.519210
C	−1.473972	−1.739973	0.525668
H	1.411571	−1.488305	0.734334
H	1.449430	−1.521876	−1.042289
H	0.962816	1.080787	1.545384
H	0.831337	2.113646	0.107229
H	3.700607	1.251406	0.863150
H	2.940844	−0.089409	1.738404
H	3.985430	−0.409817	0.342961
H	−1.939839	1.510666	0.980183
H	−3.103158	0.222973	0.717192
H	−2.172792	1.944872	−1.272751
H	−2.794449	0.327400	−1.629520
H	−0.730578	−0.436795	−2.196659
H	−0.103731	1.208850	−2.056541
H	−2.313128	−1.973095	1.191180
H	−0.684058	−2.456110	0.773482
H	−1.795798	−1.930218	−0.504245

1,7−dimethyl−diazaspiro[4.4]nonane
(x = N/A, f = N/A)

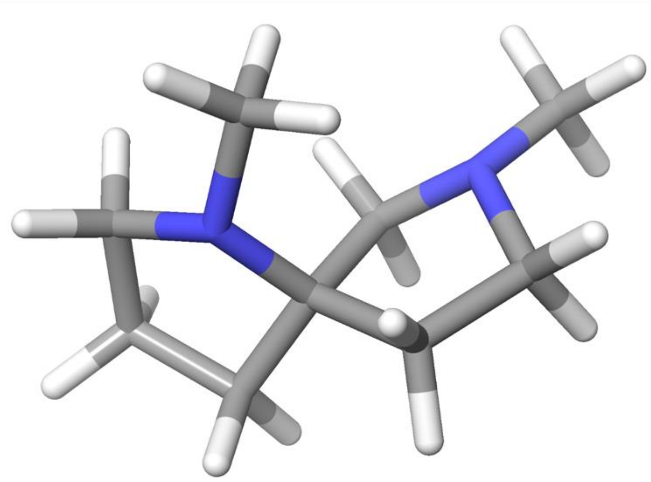

Table S167. Coordinates for the optimised geometry of
1,7−dimethyl−diazaspiro[4.4]nonane

	Coordinates / Å		
**Atom**	**x**	**y**	**z**
C	0.934013	−1.475018	0.032281
C	2.190433	−0.619178	0.070800
N	1.806864	0.519634	0.909462
C	0.358992	0.747247	0.737116
C	−0.170964	−0.430000	−0.126895
N	−1.537508	−0.924693	0.098158
C	−2.441663	0.069673	−0.481328
C	−1.710442	0.569132	−1.719304
C	−0.297974	0.006407	−1.602640
C	2.608201	1.704567	0.648969
C	−1.893635	−1.250424	1.470496
H	0.962562	−2.208363	−0.780158
H	0.837644	−2.024320	0.976436
H	2.463570	−0.307654	−0.945162
H	3.035466	−1.165217	0.501618
H	0.126658	1.718424	0.283003
H	−0.080722	0.750042	1.738619
H	−2.618894	0.909147	0.202871
H	−3.409900	−0.370395	−0.741292
H	−1.694119	1.664349	−1.740111
H	−2.186991	0.220299	−2.641673
H	0.448387	0.746263	−1.911393
H	−0.203640	−0.854774	−2.277460
H	3.669346	1.495597	0.822898
H	2.492283	2.065312	−0.379318
H	2.326677	2.513931	1.331329
H	−1.214990	−1.996952	1.893579
H	−1.904639	−0.377742	2.131076
H	−2.895012	−1.695295	1.498022

2,5−dimethyl−diazabicyclo[2.2.1]heptane
(x_n = 34, f = 1)

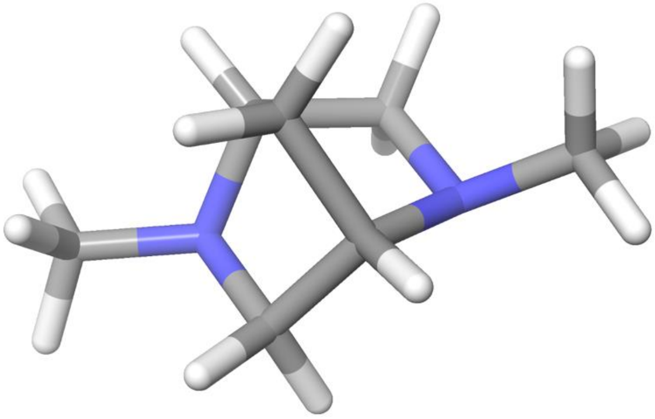

Table S168. Coordinates for the optimised geometry of **2,5−dimethyl−diazabicyclo[2.2.1]heptane**

	Coordinates / Å		
**Atom**	**x**	**y**	**z**
C	0.730451	−0.455436	−1.178405
C	−0.467393	0.445247	−0.766308
N	−1.504792	−0.424357	−0.144420
C	−0.883990	−0.799663	1.159219
C	0.508373	−0.110239	1.115616
N	1.350622	−0.835882	0.124045
C	0.145154	1.186562	0.410810
C	2.758188	−0.468258	0.195445
C	−2.784981	0.249243	0.025480
H	0.408040	−1.345717	−1.729028
H	1.421645	0.113161	−1.812978
H	−0.835053	1.066209	−1.587503
H	−1.459316	−0.421113	2.013287
H	−0.803639	−1.888149	1.251279
H	0.964332	−0.009408	2.104079
H	−0.563443	1.803487	0.972690
H	1.004957	1.805661	0.135364
H	3.329880	−1.020327	−0.558573
H	3.171779	−0.745108	1.171235
H	2.936200	0.601101	0.041442
H	−2.721354	1.155127	0.637249
H	−3.198601	0.525875	−0.950357
H	−3.507060	−0.428015	0.494606

1,3−dimethylcyclohexane
(x = 5, f= 47) – regiochemistry not listed

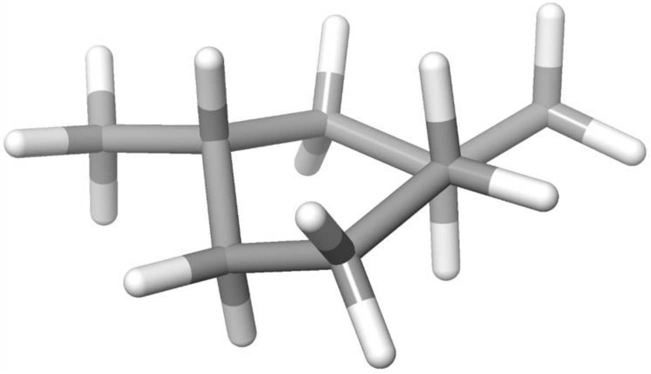

Table S169. Coordinates for the optimised geometry of **1,3−dimethylcyclohexane**

	**Coordinates / Å**		
**Atom**	**x**	**y**	**z**
C	1.202135	1.114388	−0.400719
C	1.247501	−0.221843	0.342544
C	0.017739	−1.087185	0.009082
C	−1.239506	−0.268109	−0.338450
C	−1.237956	1.081116	0.382381
C	−0.031566	1.934629	−0.016160
C	2.540581	−0.978248	0.045532
C	−2.507305	−1.061227	−0.028497
H	1.189931	0.930021	−1.483010
H	2.104092	1.704157	−0.198134
H	1.232023	−0.009309	1.420414
H	−0.176432	−1.745935	0.865722
H	0.233529	−1.753466	−0.836490
H	−1.230676	−0.073214	−1.419724
H	−1.220039	0.915345	1.467597
H	−2.158618	1.637682	0.170219
H	−0.298159	2.582556	−0.860056
H	0.213518	2.604914	0.816723
H	3.412952	−0.387232	0.343661
H	2.571837	−1.924113	0.596508
H	2.634210	−1.203076	−1.022329
H	−2.593838	−1.271134	1.042995
H	−3.398412	−0.504043	−0.336208
H	−2.507542	−2.016673	−0.563599

1,2−dimethylcyclopropane
(x = 6, f= 39) – regiochemistry not listed

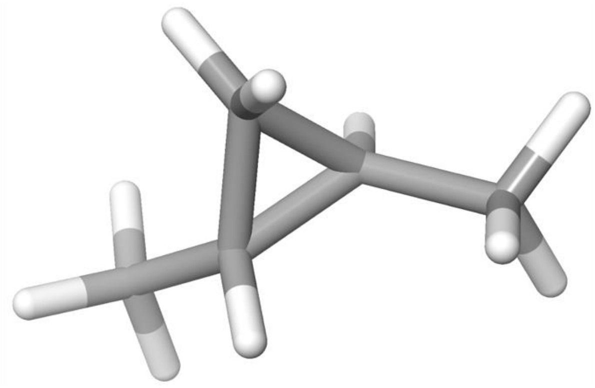

Table S170. Coordinates for the optimised geometry of **1,2−dimethylcyclopropane**

Atom	Coordinates / Å x	y	z
C	−0.556655	−0.283546	−0.733606
C	0.089953	−1.081982	0.369818
C	0.553240	0.324595	0.086889
C	−1.955442	0.208772	−0.583138
C	1.891080	0.565414	−0.524512
H	−0.343502	−0.574950	−1.756253
H	−0.468967	−1.255720	1.282691
H	0.732021	−1.908411	0.085773
H	0.300501	1.092161	0.810161
H	−2.181935	0.498804	0.448155
H	−2.662885	−0.571478	−0.880577
H	−2.118288	1.079840	−1.225361
H	2.661892	0.583398	0.252189
H	1.901151	1.532007	−1.037665
H	2.157836	−0.208903	−1.251324

1,4−dimethylcyclohexane
(x = 5, f= 47) – regiochemistry not listed

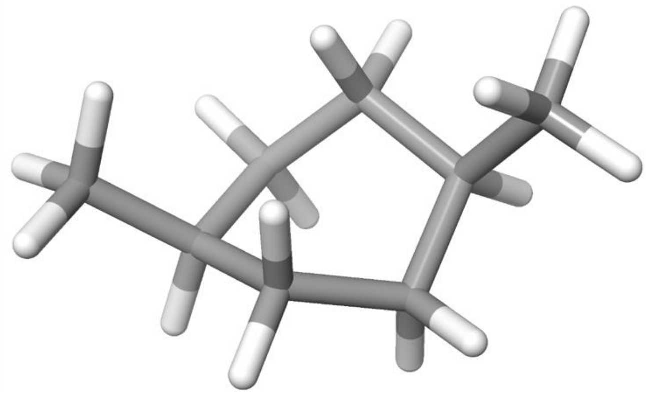

Table S171. Coordinates for the optimised geometry of **1,4−dimethylcyclohexane**

	**Coordinates / Å**		
**Atom**	**x**	**y**	**z**
C	−0.562064	−1.210340	−0.390149
C	0.860360	−0.971853	−0.884857
C	1.553416	0.171824	−0.121359
C	0.553629	1.193989	0.448262
C	−0.666076	1.348433	−0.453608
C	−1.449141	0.030292	−0.597013
C	−2.645245	−0.014805	0.355315
C	2.449078	−0.365425	0.996127
H	−1.002001	−2.062875	−0.921466
H	−0.529436	−1.490235	0.670633
H	0.820120	−0.714644	−1.951537
H	1.442606	−1.898104	−0.816438
H	2.206563	0.690776	−0.835745
H	1.053619	2.162927	0.565855
H	0.221572	0.893500	1.450314
H	−1.312838	2.149633	−0.077562
H	−0.326826	1.670794	−1.446872
H	−1.850248	−0.000129	−1.618995
H	−3.206875	−0.946494	0.229101
H	−3.330130	0.816753	0.158352
H	−2.325177	0.050978	1.400652
H	1.870328	−0.932614	1.732908
H	3.221436	−1.027433	0.590471
H	2.953330	0.455054	1.517610

7. References

S1. (a) S. Grimme, C. Bannwarth and P. Shushkov, *J. Chem. Theory Comput.*, 2017, **13**, 1989–2009; (b) C. Bannwarth, S. Ehlert and S. Grimme, *J. Chem. Theory. Comput.*, 2019, **15**, 1652–1671.

S2. C. Bannwarth, E. Caldeweyher, S. Ehlert, A. Hansen, P. Pracht, J. Seibert, S. Spicher and S. Grimme, *Wiley Interdiscip. Rev. Comp. Mol. Sci.*, 2021, **11**, e1493.

S3. F. Weigend, *Phys. Chem. Chem. Phys.,* 2006, **8**, 1057–1065.

S4. (a) O. Yahiaoui, H. D. Patel, K. S. Chinner, L. F. Pašteka and T. Fallon, *Org. Lett.*, 2021, **23**, 1157–1162.
